# Earth-Abundant Manganese Catalysts for Dehydrogenative Coupling of Alcohols and Silanols with Hydrosilanes

**DOI:** 10.1021/acs.inorgchem.6c04822

**Published:** 2026-10-15

**Authors:** Marcin Gruszczyński, Dariusz Lewandowski, Miłosz Makowski, Maciej Kubicki, Grzegorz Hreczycho

**Affiliations:** Faculty of Chemistry, Adam Mickiewicz University in Poznan, Uniwersytetu Poznańskiego St. 8, 61-614 Poznań, Poland

## Abstract

The development of catalysts and/or simple systems based on earth-abundant metals that enable the selective synthesis of siloxanes and alkoxysilanes while generating valuable hydrogen as the sole byproduct remains a major challenge in catalysis. Herein, we report a mild, scalable, and chemoselective cross-dehydrogenative functionalization protocol for the construction of Si–O–Si and Si–O–C motifs. The method relies on the dehydrogenative coupling of hydrosilanes with silanols or alcohols catalyzed by manganese complexes and simple manganese chloride, enabling efficient Si–O bond formation under operationally straightforward conditions. This transformation expands the synthetic tool box of organosilicon chemistry by introducing an earth-abundant metal catalyst bearing aminophosphine-type ligands and provides a practical approach to the synthesis of siloxanes and silyl ethers with broad functional group tolerance.

## Graphical abstract

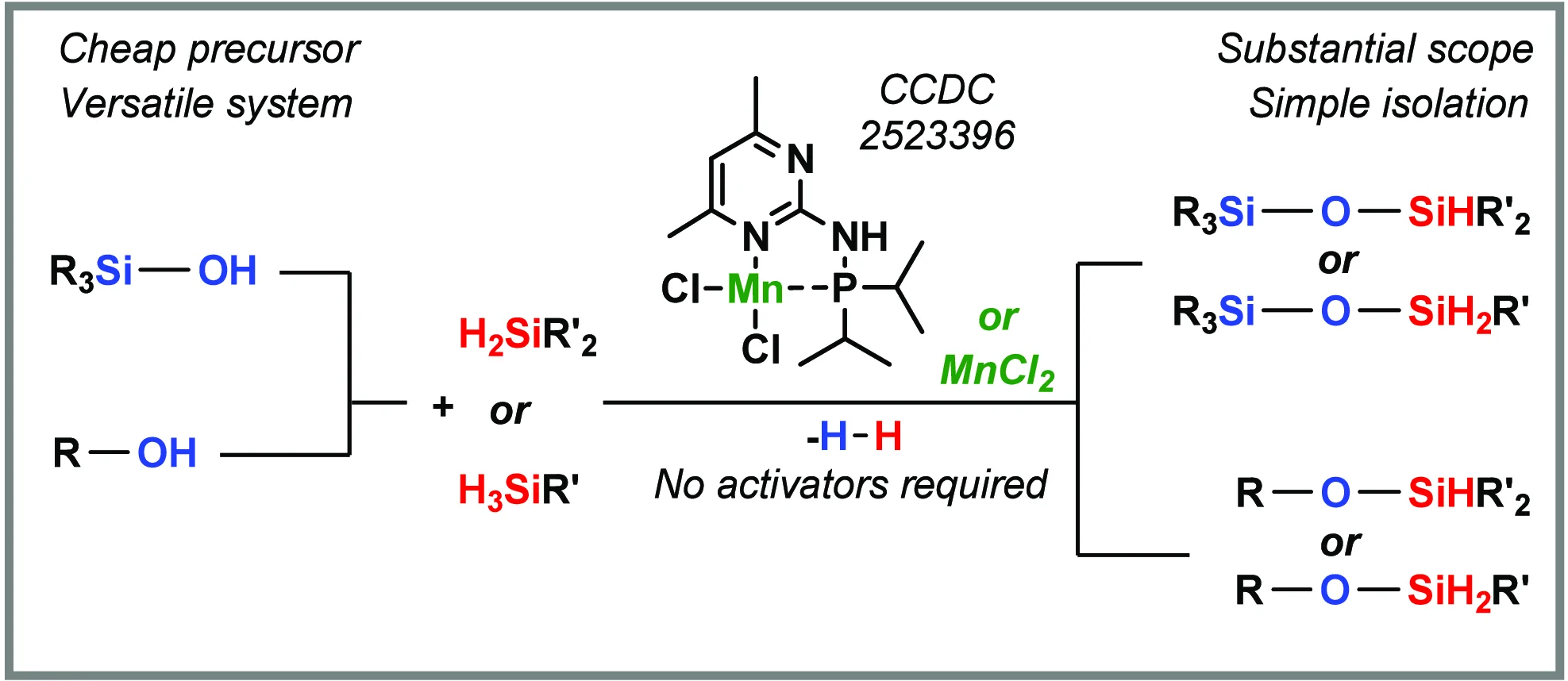

## Introduction

Siloxane motifs are omnipresent building blocks in both chemical synthesis and industrial applications.^1,2^ Owing to their unique physical and chemical properties, such as high thermal and oxidative stability, low surface energy, flexibility of the Si–O backbone, and excellent biocompatibility, siloxanes have become indispensable in a wide range of fields. These include medicine and biomedical devices, cosmetology, the textile and coatings industries, electronics, sealants, and elastomers.^3–6^ Traditionally, siloxanes are accessed through well-established stoichiometric routes, most commonly via the reaction of chlorosilanes with oxygen-based nucleophiles.^7^ However, these methods suffer from significant drawbacks. The generation of corrosive byproducts necessitates the use of stoichiometric amounts of Brønsted bases, leading to the formation of salts that make isolation and purification processes much more complicated. Moreover, such conditions limit functional group compatibility and impede the efficient synthesis of more advanced silicon-containing molecules.^1^ Beyond the fundamental need to construct Si–O bonds, more emphasis has been placed on the chemoselectivity and sustainability of siloxane-forming processes. In response, a considerable amount of effort has been devoted in recent years to the development of catalytic cross-coupling strategies involving silanols and a variety of silylating agents, including hydrosilanes, vinylsilanes, allylsilanes, and ethynylsilanes.^8–17^ From the perspective of atom economy, O–H/Si–H dehydrocoupling reactions are particularly attractive, as molecular hydrogen is the sole byproduct. Of special interest is the direct and selective synthesis of SiH- and SiH_2_-containing siloxanes, which represent valuable yet synthetically challenging motifs with significant potential in further functionalization and applications.

While catalytic routes to monohydrosiloxanes have been established, the selective construction of dihydrosiloxane analogues remains considerably more challenging because of their intrinsically higher reactivity (Figure 1). As a result, a vast majority of reported dehydrocoupling methodologies for the synthesis of hydrosiloxanes rely either on expensive 4d/5d transition metal catalysts or on strong base-mediated conditions.^18,19^ Notable recent advances include a gold-catalyzed protocol developed by Shimada, which employs phosphine ligands to enable Si–O bond formation.^20^ Despite these important contributions, the need for precious metals or stoichiometric additives continues to limit the practicality and sustainability of these methods.

**Figure 1:**
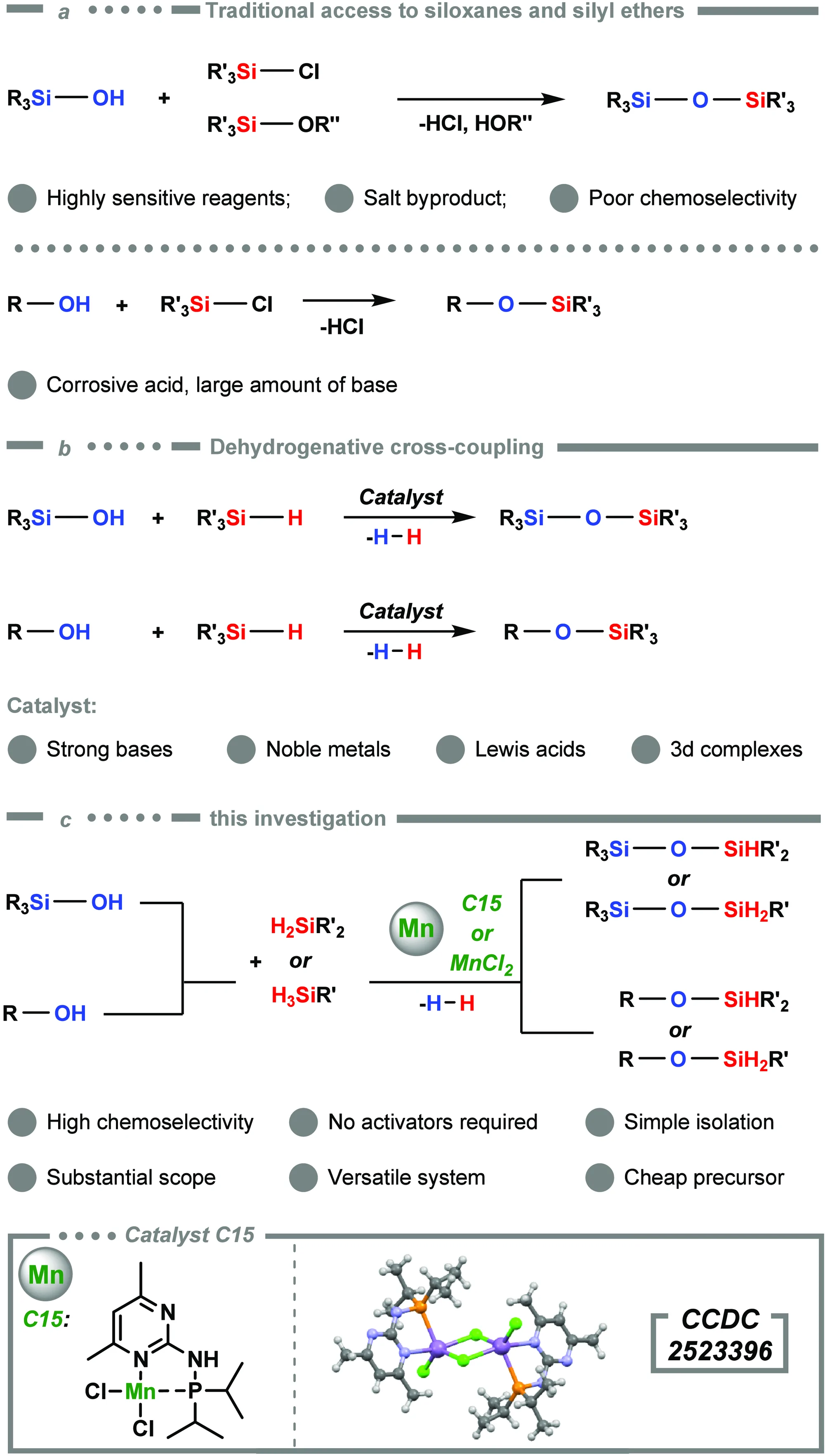
Context of this investigation. (a) Traditional synthetic pathways. (b) Dehydrogenative cross-coupling mediated by conventional catalyst categories. (c) Manganese-catalyzed dehydrogenative coupling of silanols and alcohols using hydrosilanes.

The use of earth-abundant 3d transition metals as catalysts has attracted a significant amount of attention in recent years as a sustainable alternative to precious metal systems.^21,22^ Among the various ligand scaffolds that have been explored, pincer-type complexes have emerged as particularly robust and versatile catalysts, often displaying high chemo- and regioselectivity.^23–27^ Recent significant contributions by Beller,^28,29^ Kempe,^30–33^ and Kirchner^34,35^ have demonstrated the exceptional potential of such pincer-supported metal complexes, transforming them into powerful and reliable tools for modern synthetic chemistry. In the context of dehydrogenative silylation reactions, pincer-supported 3d metal complexes have also demonstrated a considerable amount of potential. Nakajima reported iron-based catalytic systems enabling efficient Si–O bond formation that require sodium triethylborohydride as an external activator.^36^ Gunanathan disclosed an elegant approach based on an NNN-type Co­(II) complex in combination with potassium *tert*-butoxide as an activator.^37^ More recently, a cobalt-catalyzed methodology was proposed by our group, employing PNP-type aminophospine ligands, which enabled Si–O–Si and Si–O–C bond formation.^38,39^ Later, Iglesias and co-workers reported Mn­(I) complexes bearing hemilabile P,N 1,2,3-triazole-phosphane ligands as catalysts for the dehydrogenative coupling of hydrosilanes with hydroxyl compounds, with in situ generation of a manganese hydride species markedly accelerating hydrosilane methanolysis and affording turnover frequencies exceeding 45 000 h^–1^.^40^

On the other hand, related transformations have been reported using simple 3d metal salts, which display comparable catalytic activity. Dolgov and co-workers demonstrated that CoCl_2_, ZnCl_2_, and NiCl_2_ can promote the formation of silyl ethers; however, these reactions require very harsh conditions.^41^ Hilal’s group reported the use of dimanganese decacarbonyl [Mn_2_(CO)_10_] as a catalyst for the dehydrogenative coupling of monohydrosilanes and alcohols, although the method was limited to simple, nonbulky substrates.^42^ Later, using the same catalyst, as well as Re_2_(CO)_10_, Barton and Kelly demonstrated synthesis of silyl ethers at room temperature with use of phenylsilane.^43^ MnBr­(CO)_5_ was also used in dehydrogenative coupling providing unsymmetrical bisalkoxysilane carried out by Scott’s group.^44^ Leitner and co-workers reported the dehydrogenative coupling of hydrosilanes and water using 1 mol % MnBr­(CO)_5_. Notably, they found no activity with thye use of manganese halides (Mn­(II)) and other manganese­(III) and manganese(0) species under analogous conditions.^45^ The Salen-Mn­(V) catalyst was reported by Du as a catalyst in dehydrogenative coupling of alcohols and hydrosilanes.^46^ Recently, Meng and co-workers detailed the extensive utility of dimanganese decacarbonyl Mn_2_(CO)_10_ in facilitating various Si–O bond-forming reactions. While highly effective, these carbonyl systems often rely on energy-intensive activation, such as temperatures of ≤140 °C for reactions with water, or continuous UV irradiation for the dehydrocoupling of silanols.^47^ While Si–O bond formations have been predominantly enabled by Mn_2_(CO)_10_, the capacity for simple, noncarbonyl manganese salts to mediate such transformations remains scarcely explored. This represents a significant opportunity, as simple manganese halides not only are vastly less expensive but also can efficiently drive these couplings under mild, thermal conditions without the need for extreme heat or photochemical assistance.^48^

Considering recent successes within our group in cobalt-catalyzed activation of hydrosilanes/hydroboranes (proceeding via H_2_ liberation),^49,50^ we hypothesized that the use of manganese, an Earth-abundant, inexpensive, environmentally benign 3d metal, constitutes a compelling alternative to cobalt, whose broader application is increasingly limited by toxicity concerns and regulatory pressure.^51^ The superior air stability and operational robustness of manganese complexes further motivated their use in the selective assembly of unsymmetrical siloxanes and silyl ethers. Considering the demonstrated robustness of 3d pincer complexes as well as the operational simplicity of simple manganese salts, we herein report a manganese-catalyzed dehydrogenative cross-coupling reaction between silanols/alcohols and hydrosilanes, delivering a practical and sustainable method for Si–O bond formation under mild, activator-free conditions. Notably, we demonstrate the first example of Si–O bond formation catalyzed by simple, commercially available manganese chloride without the need for any additives or thermal or photochemical activation.

## Results and Discussion

We began our research with the synthesis of 22 complexes (for structures, see Tables S1 and S2) with P,N- and PNP-donor ligands (**C1–C22**) via a convenient, one-step synthetic procedure (Scheme 1). Ligands required for this process were obtained according to a known procedure.^52^ To the best of our knowledge, among all of the obtained complexes, 21 of them were previously unknown and the remaining one has been reported in the literature (see the Supporting Information).

**Scheme 1:**
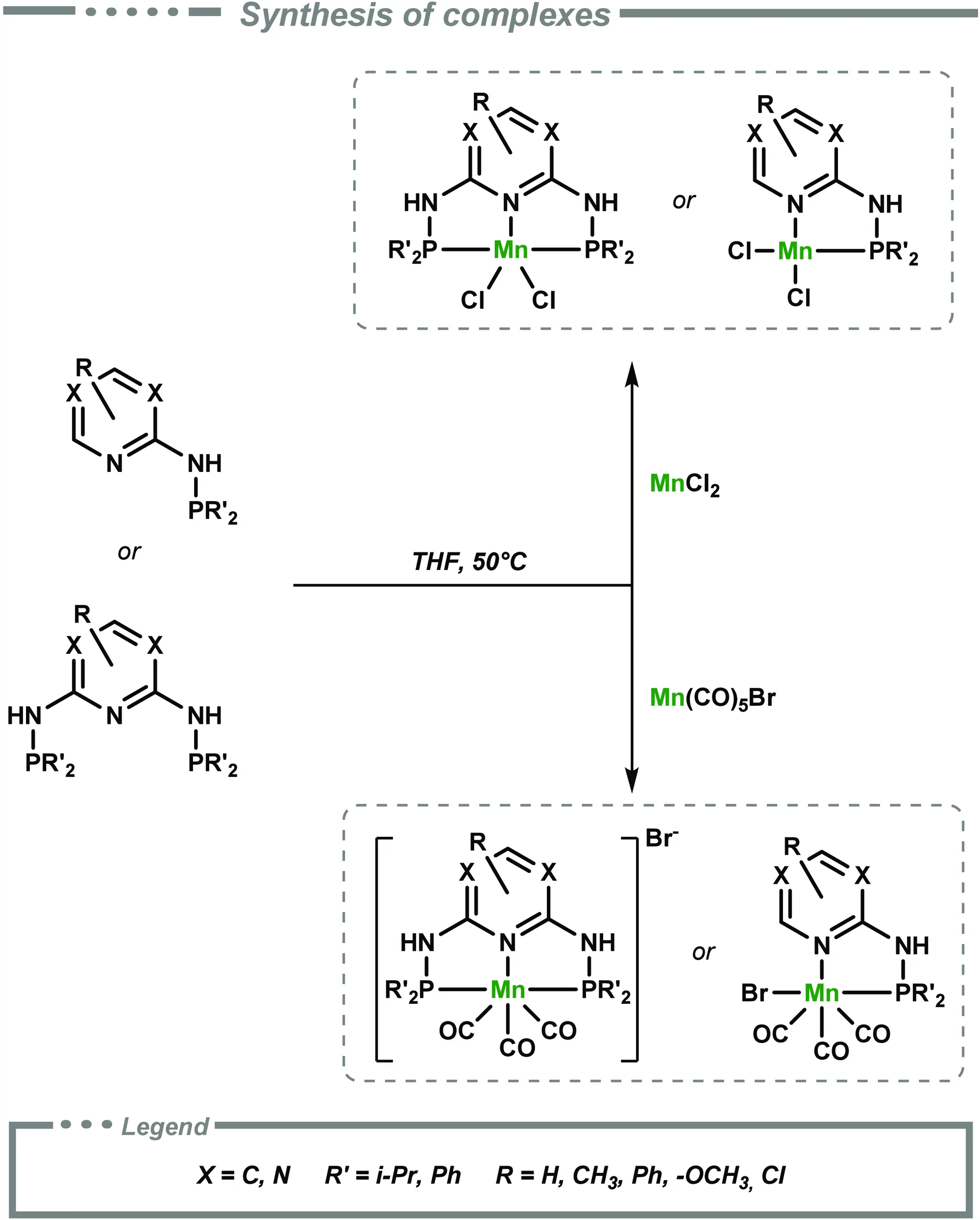
Synthesis of Aminophosphine-Based Manganese Chloride Complexes (top) and Manganese Tricarbonyl Complexes (bottom)

Subsequently, the complexes were thoroughly characterized by NMR spectroscopy; however, all complexes obtained from manganese­(II) chloride exhibited paramagnetism, which hindered their characterization by the NMR methods. In contrast, the bromide-derived complexes could be characterized by NMR spectroscopy because of their oxidation state (I). In addition, all compounds were further recrystallized and examined by X-ray diffraction when suitable single crystals were obtained.

The structures of compounds **C1**, **C2**, **C4**, **C6**, **C7**, **C13**, **C15**, **C17**, and **C20–C22** were confirmed unequivocally by X-ray single-crystal diffraction (more details in X-ray Crystallography in the Supporting Information). Figures 1 and 2 show the structure of complex **C15**, the most active and selective complex. It should be noted that in the crystal structure, our catalyst is present in a dimeric form.

**Figure 2:**
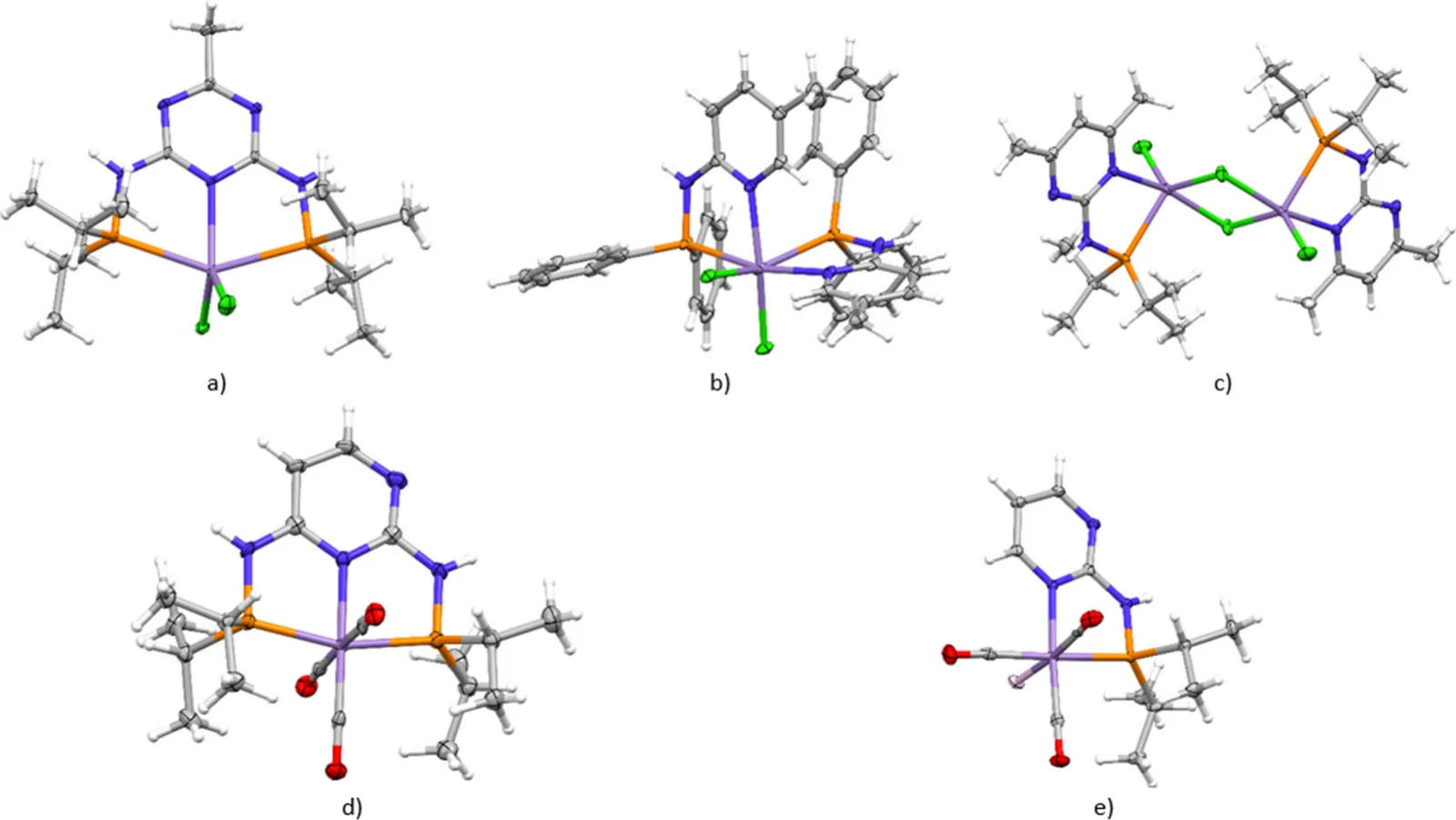
Perspective views of the chosen structures of (a) **C1**, (b) **C13**, (c) **C15**, (d) **C20**, and (e) **C22**. Ellipsoids are drawn at the 50% probability level, and hydrogen atoms are represented by spheres of arbitrary radii.

In principle, there are five different types of complex structures in the set: five five-coordinated MnCl_2_(PNP) complexes (**C1**, **C2**, **C4**, **C6**, and **C7**), one six-coordinated MnCl_2_(PN)_2_ derivative (**C13**), one cationic six-coordinated [Mn­(CO)_3_(PNP)]Br (**C20**), two two-centered, di-Cl-bridged, *C*_*i*_-symmetrical five-coordinated [Mn­(μCl)­Cl­(PN)]_2_ (**C15** and **C17**), and two hexacoordinate neutral complexes of the MnBr­(CO)­3­(PN) type (**C21** and **C22**).

The perspective views of the representative examples of each family (**C1**, **C13**, **C15**, **C20**, and **C22**) are presented in Figure 2. The conformations of ligands are similar in all complexes, with P atoms in *cis* position with respect to the N1 ring atom (see Table S10 and the values of appropriate torsion angles). In the crystal structures, N–H···N hydrogen bonds, accompanied in some ligands (with two P atoms) by N–H···P ones, join the molecules into rings or chains (see Table S12 contains the hydrogen bond data). Interestingly, in all of the Cl complexes, the structures contain solvent THF molecules, which act as the acceptor for hydrogen bonds (see Table S12). It is important to distinguish between the solid-state structures of the isolated precatalysts and the proposed active species in solution. While X-ray crystallography reveals more coordinated or dimeric solid-state structures, such as six-coordinated **C13** and pentacoordinated dimer **C15**, these complexes are proposed to undergo dissociation under homogeneous catalytic conditions. This *in situ* dissociation generates less coordinated, monomeric active species (e.g., tetracoordinated), thereby providing the open coordination sites necessary to facilitate the catalytic cycle.

Next, we investigated the catalytic activity of all previously synthesized manganese complexes in the dehydrocoupling reaction between silanols and silanes. For the purpose of the optimization process, we selected *tert*-butyldimethylsilanol and phenylsilane. The outcomes of our optimization study are presented in Table 1 (for more details see Tables S3–S5). The initial reaction conditions were established using 1 mol % catalysts (**C1–C22**) in THF at room temperature for 24 h under an argon atmosphere. We conducted three series of experiments using different substrate ratios (1:1, 2:1, and 1:2 silanol:silane), as an excess of either substrate was found to influence product selectivity. All investigated aminophosphine manganese complexes exhibited high catalytic activity, leading to nearly complete conversion of silanol and silane (up to 99%). Notably, only two complexes, **C11** and **C15**, enabled the nearly exclusive formation of desired mono- and dehydrocoupling products **2** (Figure 3) and **14** (Figure 4) with satisfying conversions. Because the solid-state structure of **C15** was unambiguously confirmed via single-crystal X-ray diffraction, it was selected as the model catalyst for all subsequent detailed studies.

**Table 1:**
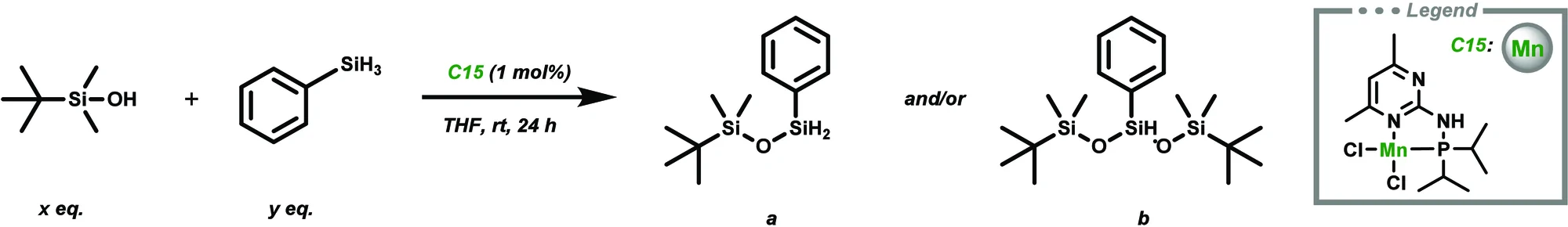
Optimization of the Dehydrogenative Coupling between Silanols and Silanes

	initial conditions	*X* equiv	*Y* equiv	conversion^a^ (%)	selectivity (a:b)
1	no change	2	1	100^b^	4:96
2		1	2	100	90:10
3		1	1	99	53:47
4	neat	1	1	99	34:66
5	toluene	1	1	99	90:10
6	toluene, 60 °C	1	1	99	8:92
7	1 h	2	1	13^b^	99:1
8	1 h, 60 °C	2	1	94^b^	1:99
9	air	2	1	99^b^	10:90
10	no catalyst	2	1	0^b^	–
11	MnCl_2_, 60 °C	2	1	99^b^	5:95
12	MnCl_2_	1	2	99	73:27
13	MnCl_2_	1	1	99	50:50
14	MnSO_4_	1	1	46	53:47
15	Mn(OAc)_2_	1	1	51	54:46
16	MnI_2_	1	1	99	53:47

a Conversion (%) of silanol determined by GC with *n*-dodecane as the internal standard.

b Conversion of silane determined by GC with *n*-dodecane as the internal standard.

**Figure 3:**
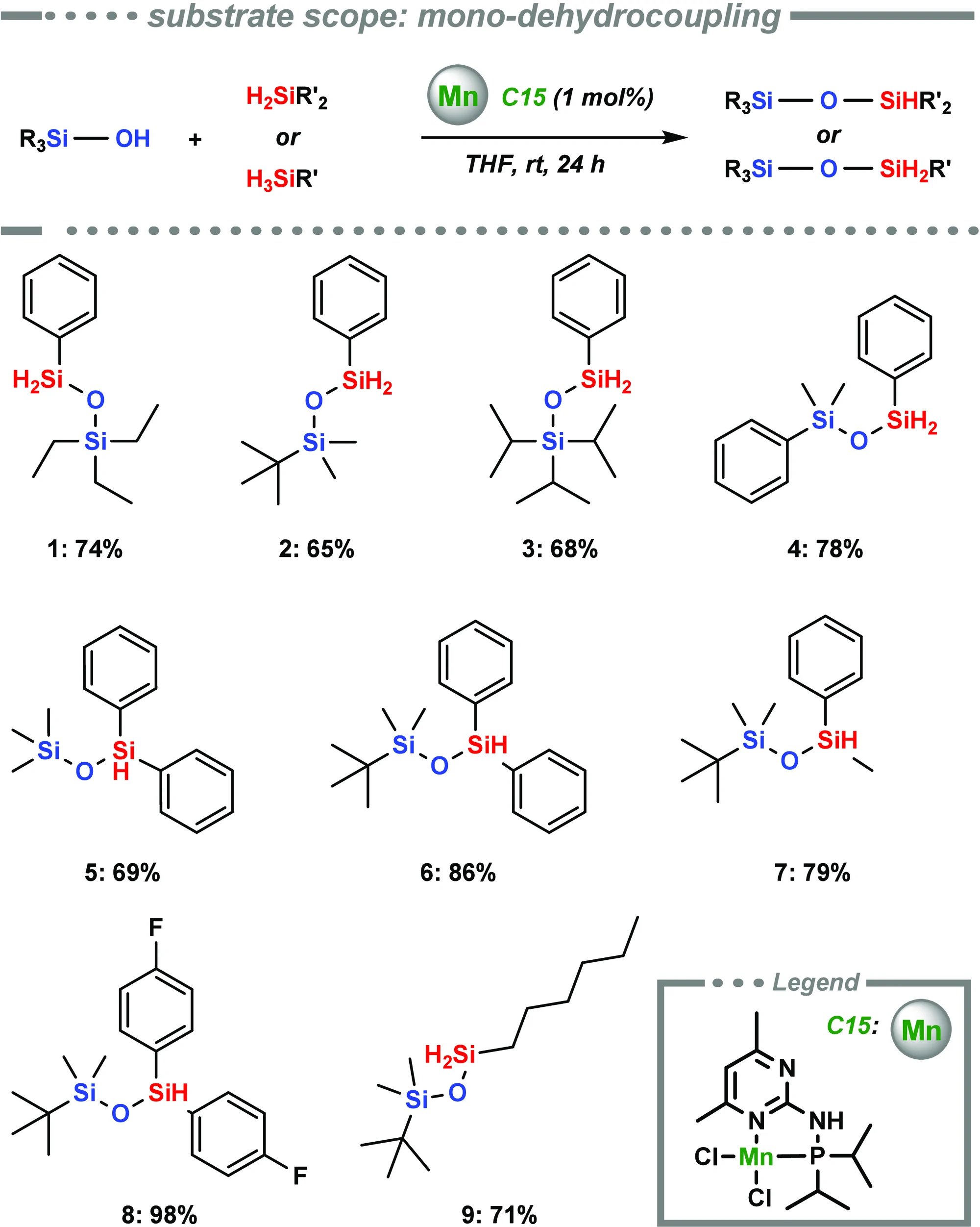
Substrate scope for monoalkoxysilanes with isolated yields.

**Figure 4:**
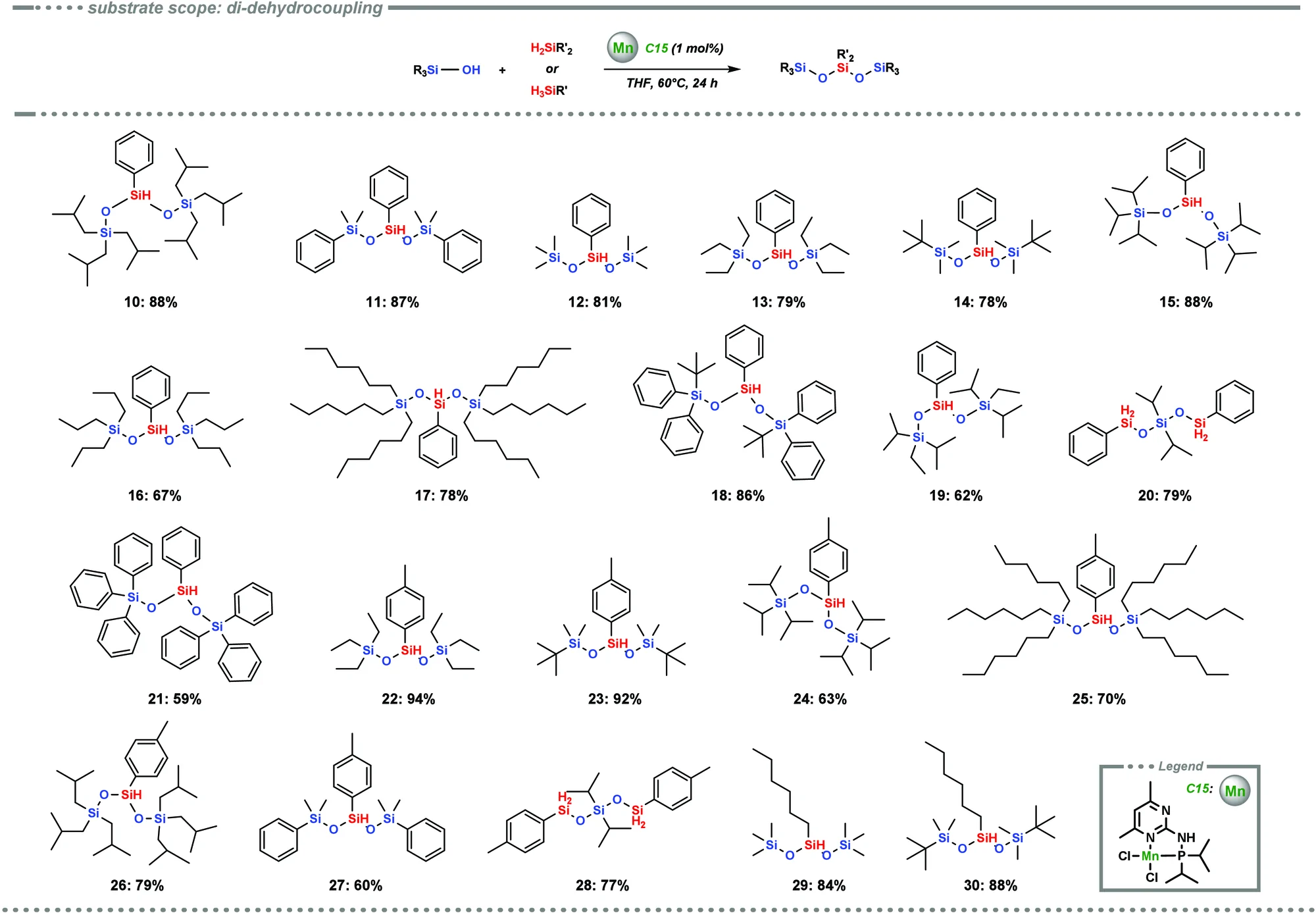
Substrate scope for dialkoxysilanes with isolated yields.

In the next step, we evaluated the effect of temperature over a range from 0 to 110 °C. Surprisingly, decreasing the temperature to 0 °C did not significantly alter the final conversion or reaction selectivity after 24 h (see Table S5). In contrast, an increased temperature (60 and 110 °C) significantly increased the reaction rate, enabling nearly complete conversion within 1 h.

Furthermore, evaluation of the catalytic system under air demonstrated that the catalyst is air stable. The catalyst-free attempt was also carried out and proved the essential role of 3d metal catalysis.

Subsequently, a simple manganese chloride was also examined in this process (Table 1, entry 11) and surprisingly exhibited similar activity. Therefore, we also evaluated the activity of other simple manganese salts; however, none of them provided satisfactory results. Consequently, we decided to carry out a series of further experiments with manganese chloride to confirm that it is also an effective catalyst for the studied processes. Importantly, the free ligand (with or without the activator) proved to be catalytically inactive, confirming that the intact complex is required for the reaction.

The structural diversity of the evaluated complexes highlighted a clear dependence on the metal’s oxidation state. The Mn­(II) precatalysts (**C1–C17**) proved to be highly efficient and selective. In contrast, the Mn­(I) variants (**C18–C22**) demonstrated inferior catalytic performance, characterized by reduced overall conversions (down to 63% for **C18**) and poorer chemoselectivity. Consequently, the Mn­(II) oxidation state seems to be essential for maximizing the efficiency and product selectivity of this transformation.

Having established optimized conditions (for didehydrocoupling, a 2:1 silanol:silane ratio, 1 mol % **C15**, THF, 60 °C, 24 h (silane as the limiting substrate, absolute concentration of 0.25 M); for monodehydrocoupling, a 1:2 silanol:silane ratio, 1 mol % **C15**, THF, room temperature, 24 h (silanol as the limiting substrate, absolute concentration of 0.5 M)), we extended our protocol to encompass variously substituted silanols.

The methodology tolerates both aryl- and alkyl-substituted silanols and silanes, including phenyl-, hexyl-, and fluoroaryl-derived substrates (Figure 3).

We have shown that the proper choice of the substrate ratio enables the selective formation of SiH_2_-containing siloxanes (**1–4** and **9**). Our protocol has shown that even silanols with ethyl substituents can selectively lead to dihydrosiloxanes. Intrigued by the high efficiency and chemoselectivity of the transformation, we next pursued the development of further applications for our catalytic system. Encouragingly, this strategy also enabled the dehydrocoupling of secondary silanes with silanols (**5–8**), providing easy access to SiH-containing siloxanes.

Subsequently, by changing the substrate ratio to 2.0 equiv of silanol and 1.0 equiv of silane (2:1 silanol:silane), we were able to shift the chemoselectivity toward dehydrocoupling, affording the corresponding SiH-containing siloxanes in good yields. Therefore, phenylsilane and hexylsilane were readily converted into the corresponding disiloxanes in commendable yields (Figure 4). Our system proved to be working even with very bulky substrates in dehydrocoupling reactions (**18**, **21**, and **25**). In summary, our manganese catalyst enables the synthesis of several monohydrosiloxanes as well as dihydrosiloxanes with high chemoselectivity and excellent efficiency. Since monosilanols were successfully used by us, we subsequently turned our attention to silanediols. As shown below, two cross-coupling products derived from diisopropylsilanediol were afforded (**20** and **28**).

Our research aimed to explore the versatility of this approach, leading us to investigate alcohols in the dehydrogenative coupling process with hydrosilanes. In this way, we obtained a wide variety of silyl ethers in very good yields with low catalyst concentrations under mild reaction conditions (Figure 5). We were able to obtain monosubstituted alkoxysilanes as well as di- and trisubstituted derivatives. To our delight, secondary and tertiary alcohols were readily adopted in this approach, which is quite challenging, particularly for commonly used base-catalyzed reactions. Interestingly, selective formation of monoalkoxylated products was readily achieved, whereas a comparable cobalt-catalyzed protocol resulted in a mixture of mono- and disubstituted products.^38^ Notably, no reduction processes or hydrosilylation products were observed under manganese-catalyzed conditions.

**Figure 5:**
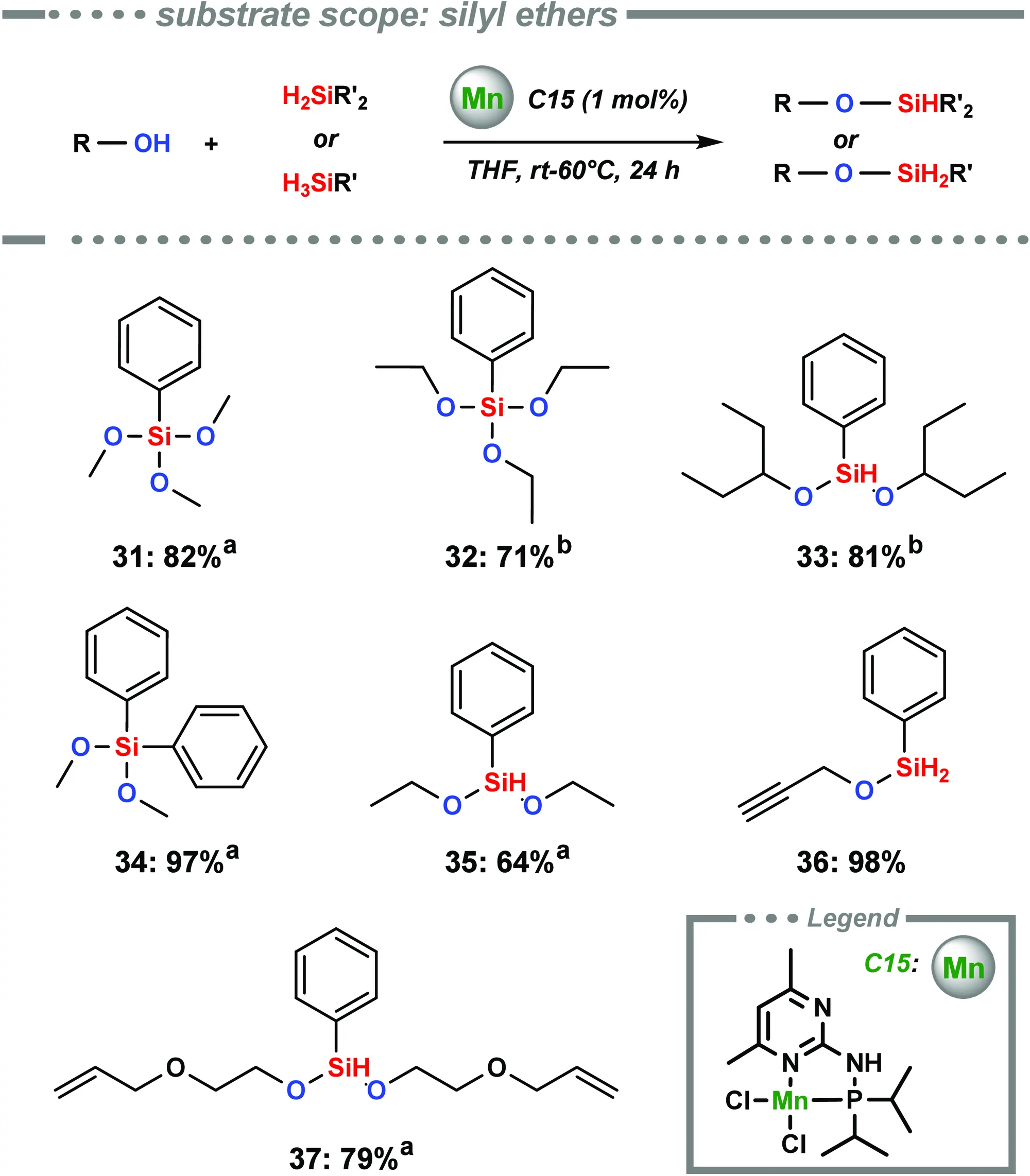
Substrate scope for silyl ethers with isolated yields at (a) 60 and (b) 110 °C with 5 equiv of alcohol.

In parallel with the investigations involving manganese complexes, we evaluated the catalytic activity of MnCl_2_ in the formation of Si–O bonds. To our delight, manganese chloride exhibited activity comparable to that of designated complex **C15**. To substantiate this observation, several reactions were conducted twice under identical conditions to assess whether similar activity and selectivity could be achieved by using a simple manganese salt. Although our complex proved to be a highly efficient catalyst for a wide range of substrates, simple manganese chloride can be successfully employed in the case of selected silanols. The results are summarized in Scheme 2.

**Scheme 2:**
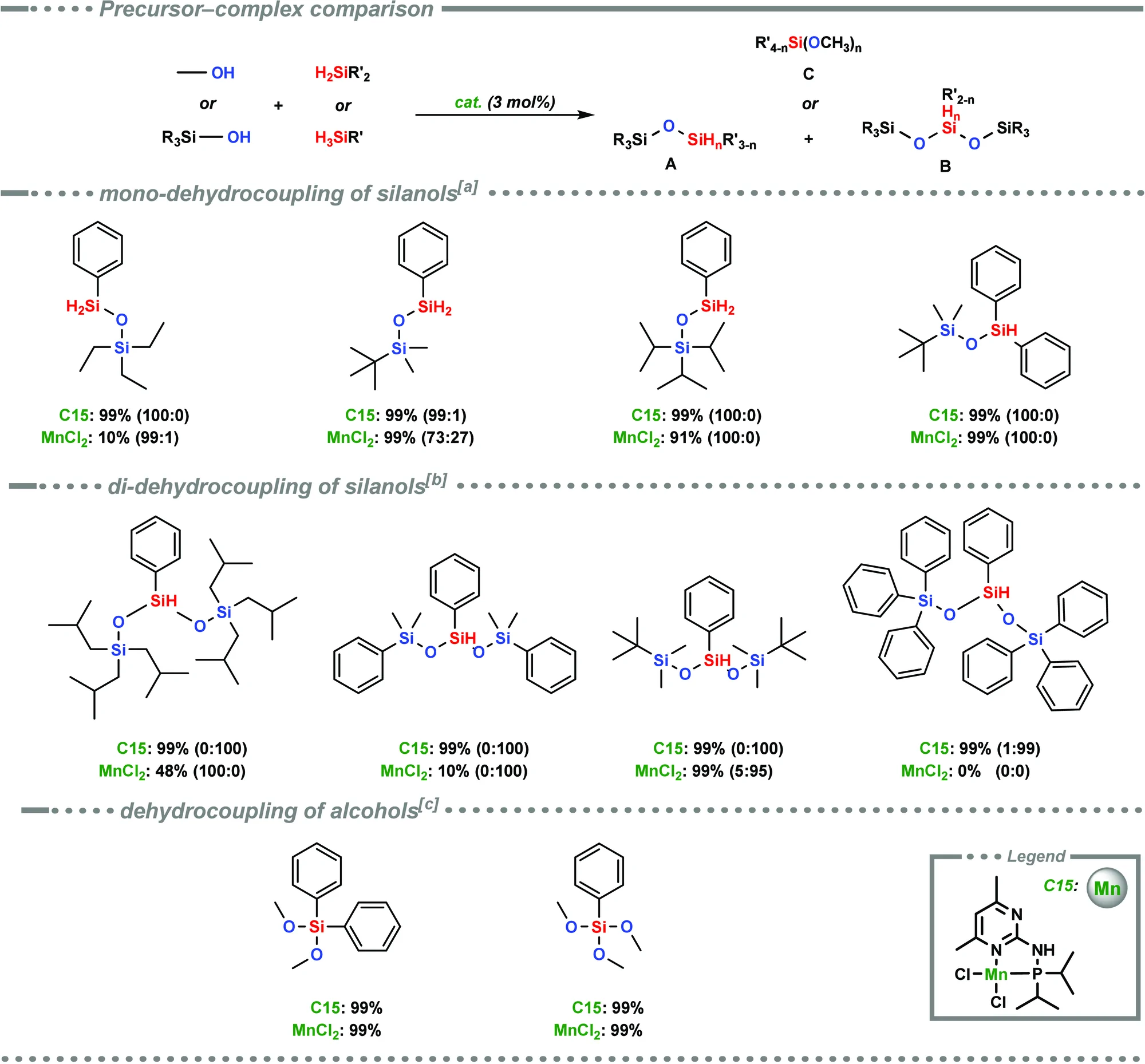
Comparative Studies of Manganese Chloride^a^

a Silanol conversions are reported. Reaction conditions: 2:1 silane:silanol, 3 mol % catalyst, rt, THF, 24 h.
b Silane conversions are reported. Reaction conditions: 1:2 silane:silanol, 3 mol % catalyst, 60 °C, THF, 24 h.
c Silane conversions are reported. Reaction conditions: 3 mol % catalyst, 60 °C, methanol as a solvent, 24 h.
d Values in parentheses correspond to reaction selectivity.

To contextualize the catalytic efficiency of the developed system, the turnover numbers (TON) and turnover frequencies (TOF) of well-defined manganese complex **C15** and simple MnCl_2_ were evaluated against established literature benchmarks for both precious and earth-abundant metal catalysts (see Table S6). In the current state of the art, noble metal systems typically achieve moderate to high efficiencies. Concurrently, reported earth-abundant counterparts generally display lower to moderate performance, exemplified by prior manganese systems (Table S6, entry Mn-1; TON = 1131; TOF = 6653 h^–1^),^53^ with the rare exception of highly active zinc-based networks (Table S6, entry Zn-1; TON = 10^7^; TOF = 10^7^ h^–1^).^54^

Remarkably, our well-defined manganese complex **C15** exhibited exceptional catalytic proficiency that significantly bridges, and in several aspects outperforms, these noble metal standards. Under the optimized neat conditions at ultralow catalyst loadings (0.001 mol %), **C15** achieved an outstanding TON of 99 000 and a TOF of 99 000 h^–1^ for silylether synthesis within 1 h (Table 2, entry 6). For monocoupling transformations, **C15** similarly demonstrated satisfactory efficacy, delivering a TON of 1360 and a TOF of 75.6 h^–1^ (for more data see Table S7).

**Table 2:**
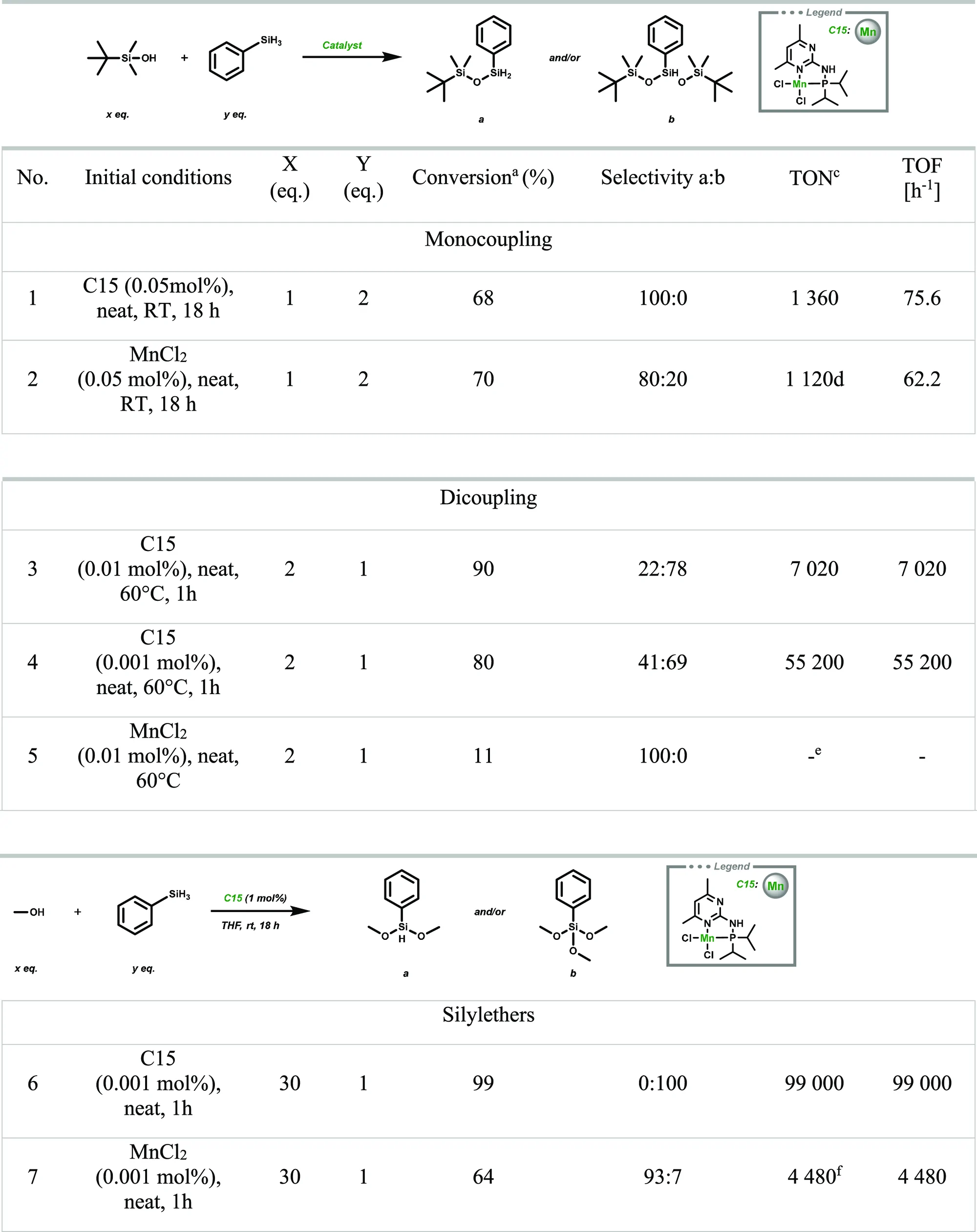
Comparative Studies of the Turnover Number (TON) and Turnover Frequency (TOF) between **C15** and MnCl_2_

a Conversion (%) of silanol determined by GC with *n*-dodecane as the internal standard.

b Conversion of silane determined by GC with *n*-dodecane as the internal standard.

c TON calculated for the product with the highest selectivity.

d TON calculated for the monocoupled product.

e TON calculated for the dicoupled product.

f TON calculated for the tricoupled product.

To ascertain the role of the ligand architecture, control experiments utilizing simple MnCl_2_ were conducted. While MnCl_2_ proved to be a competent precatalyst under neat conditions for monocoupling (TON = 1120), its performance dramatically lagged behind that of **C15** in dicoupling and silyl ether synthesis, yielding a significantly lower TON of 4480 and a TOF of 4480 h^–1^ (Table 2, entry 7). This sharp contrast underscores the pivotal role of the custom-tailored ligand coordination sphere in **C15**, which not only stabilizes the base metal center under neat reaction conditions but also drastically enhances its activity.

To demonstrate the practical utility of this Mn-catalyzed dehydrogenative coupling reaction, the model reaction was conducted on a gram scale using a Schlenk line. The reaction of *tert*-butyldimethylsilanol and phenylsilane worked well and afforded the corresponding product in 97% isolated yield, as illustrated in Scheme 3. The obtained product (**14**) was subsequently used in a typical hydrosilylation reaction with 1-decene using the Karstedt catalyst, affording addition product **38**. To demonstrate that the products obtained using our protocol are suitable for further transformations, we repeated the reaction using a different disiloxane (**7**) and trimethoxyvinylsilane, again affording desired addition product **39**. These results indicate that the protocol can be readily scaled and holds promise for industrial applications.

**Scheme 3:**
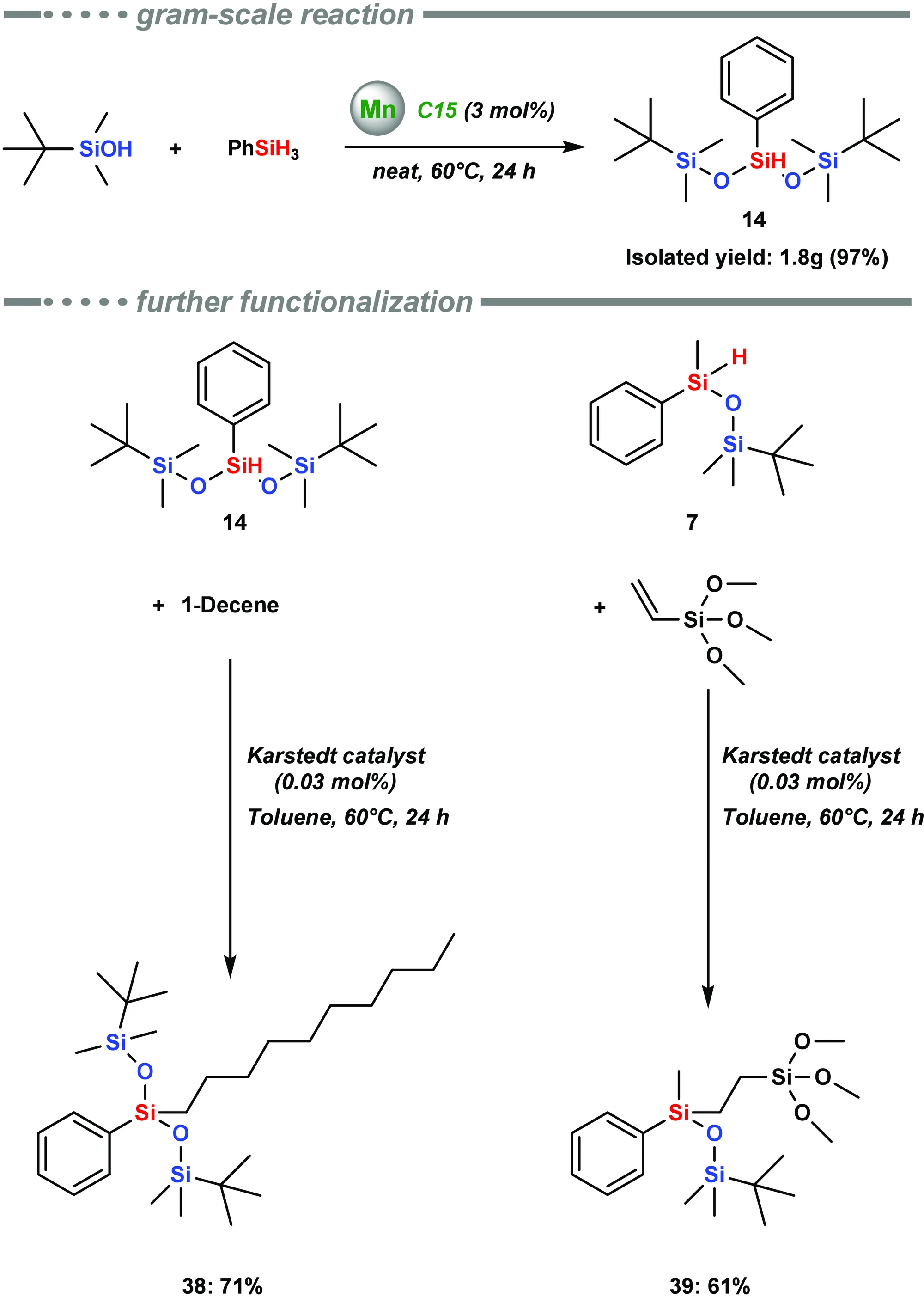
Gram-Scale Reaction and Functionalization

## Mechanistic Studies

To gain insight into the mechanism of the reaction, we carried out a series of NMR, IR, and GC-MS experiments (for details see Mechanistic Studies in the Supporting Information). Stoichiometric reactions of the manganese catalyst with either phenylsilane or *tert*-butyldimethylsilanol yielded no observable NMR resonances, consistent with a strongly paramagnetic species and suggesting that manganese most likely remains in the +II oxidation state under these conditions. GC-MS analysis of the reaction mixture revealed only trace amounts of disiloxane PhH_2_Si–O–SiH_2_Ph, which likely arises from minor hydrolysis of chlorosilane intermediates potentially formed via formal H/Cl exchange between PhSiH_3_ and Mn–Cl, followed by reaction with residual water in the solvent. The very low level of disiloxane indicates that chloride exchange is, at most, a negligible side process and does not contribute meaningfully to the catalytic cycle.^39,50^

We have conducted the reaction under the standard conditions to obtain disiloxane and measured the concentration of each substrate as well as the mono- and didehydrocoupling products, based on a known amount of the internal standard (Figure 6). Tracking the normalized relative concentrations over an 18 h period via GC-MS revealed a sequential kinetic profile (see Tables S14–S16 for data). The primary substrates, silane and silanol, exhibited a rapid and continuous depletion, achieving complete consumption by the 18 h mark. Concurrently, the monodehydrocoupling intermediate (monosiloxane) formed rapidly, reaching its peak concentration between 2 and 4 h before its levels began to decrease as it was consumed in the subsequent reaction step. Meanwhile, the final didehydrocoupling product (disiloxane) accumulated steadily throughout the observed time frame, ultimately emerging as the dominant chemical species.

**Figure 6:**
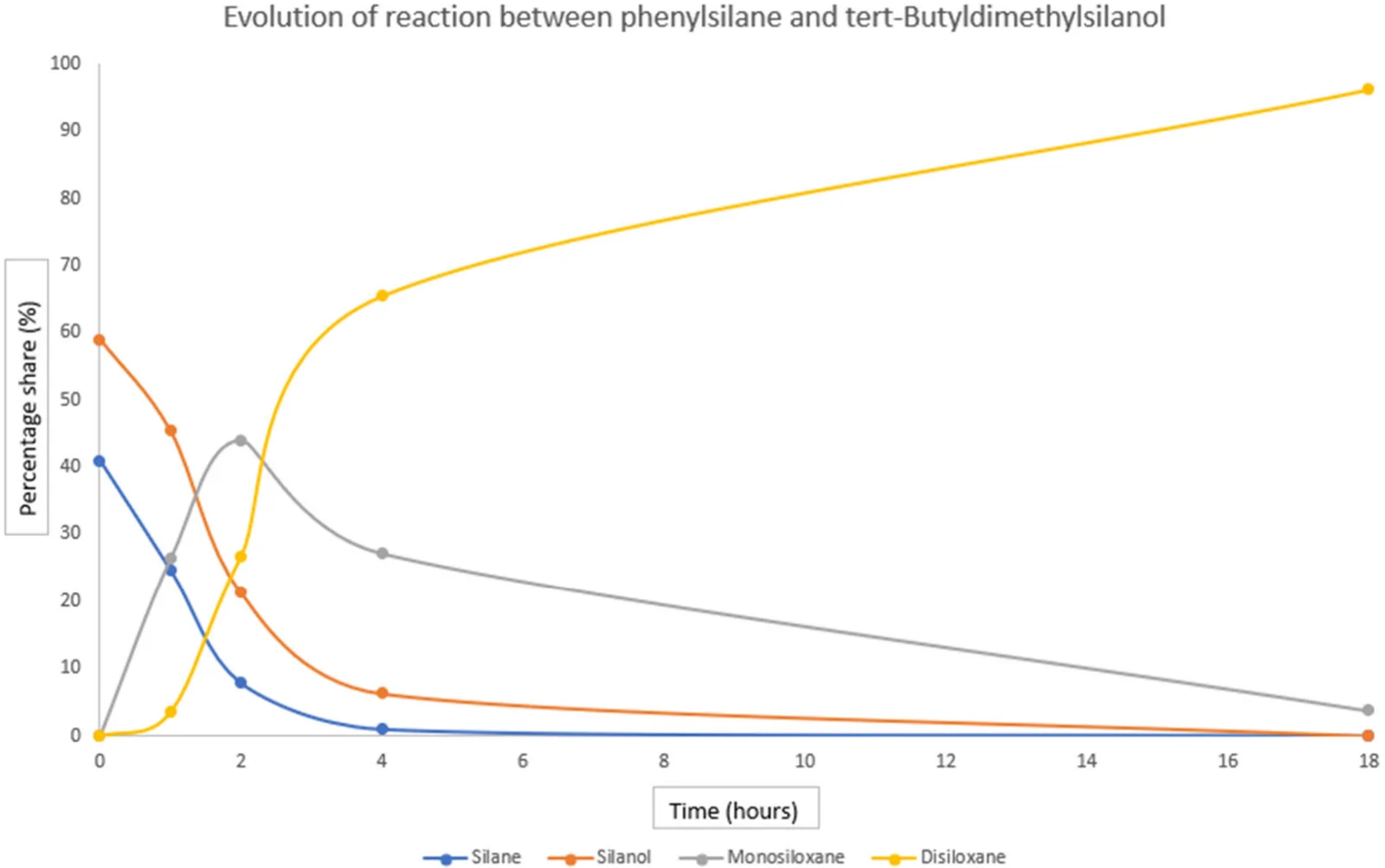
Speciation diagram of the reaction between phenylsilane (1 equiv) and *tert*-butyldimethylsilanol (2 equiv) under the standard reaction conditions.

Real-time FT-IR monitoring of the mixture of phenylsilane and the Mn catalyst showed no detectable shifts or intensity changes of the characteristic Si–H bands upon addition of the catalyst or the appearance of bands that can be attributed to Mn–H species.^55,56^ These observations suggest that classical oxidative addition of the Si–H bond to manganese does not occur to an appreciable extent (see the Supporting Information for more data).

Considering that manganese can access multiple oxidation states and in some systems can mediate radical pathways, we performed a radical-trapping experiment using TEMPO. The reaction proceeded with full conversion and without inhibition, indicating that a radical mechanism is unlikely. Additional control experiments employing QuadraPure and the mercury drop test further support a homogeneous reaction pathway.^57–60^

Based on the above experimental results and the literature,^42,43,45,46,48,61–64^ we propose a theoretical homogeneous mechanism in which manganese remains in the +II oxidation state throughout the catalytic cycle. In the first step, the silane coordinates to the manganese center via a σ-donation interaction, forming a σ-complex, in a manner analogous to previously reported mechanisms.^62,64^ To probe this interaction, detailed kinetic and kinetic isotope effect (KIE) studies were performed (see Tables S17–S24; for KIE, see Tables S25 and S26). Rate–concentration analysis yielded the empirical rate law *v* = *k*_obs_[Mn]^1/3^[silane]^2^[silanol]^0^. While solid-state structural characterization indicates the precatalyst exists as a chloride-bridged dimer, the observed one-third fractional order suggests dynamic solution-state speciation, likely involving a trimeric aggregate that must dissociate into the catalytically active mononuclear species. Crucially, isotopic labeling utilizing deuterated diphenylsilane (Ph_2_SiD_2_) yielded a primary KIE of 3.0. This substantial value, coupled with the zero-order dependence on silanol, confirms that the rate-determining step constitutes the cleavage of the Si–H bond by the active monomeric manganese center. Furthermore, the unusual second-order dependence on silane implies a highly complex transition state during this bond cleavage, potentially involving a bis­(silane) intermediate or electrophilic assistance from a second silane molecule. At this stage, the exact nature of this transition state and the macroscopic cycle remains a working hypothesis.

Furthermore, the reaction was also performed in the presence of a strong bidentate ligand, 1,10-phenanthroline. Under these conditions, only 51% conversion was observed, suggesting that the phenanthroline ligand occupies coordination sites at the manganese center, thereby hindering σ-donation from the silane and reducing its activation. Consequently, it could be possible that the activated silane reacts with the silanol to form the siloxane product, with the release of hydrogen being observed, thereby enabling regeneration of the manganese complex and completing the catalytic cycle.

## Experimental Section

Air- and moisture-sensitive reactions were carried out under an argon atmosphere using standard Schlenk techniques or a glovebox. Solvents used for all experiments were purchased from Honeywell or Sigma-Aldrich (Merck), dried over calcium hydride (CaH_2_), and purified by distillation. Toluene was additionally dried over sodium. Ligands and Mn complexes were prepared in accordance with previously reported methods^65^ using reagents purchased from Sigma-Aldrich (Merck) or ABCR GmBH. Silanes, silanols, and alcohols were purchased from Sigma-Aldrich, ABCR GmBH, Ambeed, Apollo Scientific, or Acros Organics and used as received. Non-commercially available silanols (e.g., triisobutylsilanol, trihexylsilanol, tripropylsilanol, diisopropyl­(ethyl)­silanol, diisopropylsilanediol, and *tert*-butyldiphenylsilanol) were synthesized from corresponding chlorosilanes by well-known procedures using water and triethylamine,^66^ while non-commercially available silanes (e.g., *p*-tolylsilane) were synthesized from corresponding chlorosilanes by well-known procedures using LiAlH_4_. The progress of the reaction was monitored by GC chromatography using Agilent 8860 GC and 5977B GC/MSD instruments with an Agilent 8860 GC system by determining the conversion of the silanol using *n*-dodecane as the internal standard. The structures of the products were determined by NMR spectroscopy and mass spectrometry. The ^1^H NMR (400 MHz), ^13^C NMR (101 MHz), and ^29^Si NMR (80 MHz) spectra were recorded on a Bruker Avance III HD NanoBay spectrometer, using chloroform-*d*_1_ (CDCl_3_) or benzene-*d*_6_ (C_6_D_6_) as the solvent. Deuterated solvents were purchased from Deutero GmbH (CDCl_3_, 99.6 atom % D) or Sigma-Aldrich (Merck) (C_6_D_6_, 99.8 atom % D; CDCl_3_, 99.8 atom % D) and used as received.

### Dehydrogenative Coupling of Silanols/Alcohols and Silanes

To a 12 mL vial equipped with a magnetic stirring bar under an inert atmosphere (glovebox), the catalyst (**C15**, 1 mol %; MnCl_2_, 1 mol %), dry THF (500 uL), silanol/alcohol (0.25 mmol), and silane (depending on the desired product, 0.125 mmol for monoalkoxysilane and 0.50 mmol for dialkoxysilane) were added. The reaction mixture was stirred at room temperature (or 60 °C) for 24 h. After this time, the solvent and residue were evaporated under reduced pressure. The obtained products were dissolved in hexane and isolated via filtration. The pure products were identified by ^1^H and ^13^C NMR spectroscopies and MS.

## Conclusions

In conclusion, we have developed and structurally characterized a series of new manganese­(II) and manganese­(I) complexes with P,N- and P,N,P-donor ligands that enable selective dehydrocoupling between silanols/alcohols and silanes under mild conditions with low catalyst loadings. The methodology affords a broad range of siloxanes and disiloxanes as well as silyl ethers, demonstrating the versatility of the system and scalability. Notably, the air stability of the complexes enables gram-scale reactions to be performed under ambient conditions in air at room temperature. Although manganese chloride exhibits good activity and selectivity, there remains a strong demand for catalysts bearing tunable ligands as the simple salt is insufficient for more demanding substrates. Nevertheless, demonstrating the catalytic competence of manganese chloride is an important finding, providing a practical, economical, and operationally simple alternative to less challenging transformations.

## Supplementary Material



## Notes

Tables, NMR spectra, X-ray analysis data, further experimental details, and additional references (PDF)
