# Supplementary material for: Earth-Abundant Manganese Catalysts for Dehydrogenative Coupling of Alcohols and Silanols with Hydrosilanes
Source: Inorg Chem. 2026 Oct 15;65(43):25577–90. doi: 10.1021/acs.inorgchem.6c04822 (PMC13733269; doi:10.1021/acs.inorgchem.6c04822)
Supplement: Supplementary file 1 [file ic6c04822_si_001.pdf]

Supporting Information

Earth-Abundant Manganese Catalysts for Dehydrogenative Coupling of Alcohols and Silanols with Hydrosilanes

Marcin Gruszczyński,[a] Dariusz Lewandowski,[a] Miłosz Makowski,[a] Maciej Kubicki,[a] and Grzegorz Hreczycho*[a]

[a] Faculty of Chemistry, Adam Mickiewicz University in Poznan
Uniwersytetu Poznańskiego St. 8, 61-614 Poznań (Poland)
E-mail: g.h@amu.edu.pl

CONTENT:

GENERAL INFORMATION 6

OPTIMIZATION STUDIES 7

TURNOVER NUMBER (TON) AND TURNOVER FREQUENCY (TOF) 13

State of the Art 13

Present Work: Manganese-Catalyzed Dehydrogenative Coupling 16

EXPERIMENTAL PROCEDURES 19

Synthesis of ligands 19

Synthesis of Mn-based complexes 19

Dehydrogenative coupling of silanols/alcohols and silanes 19

Gram-scale reaction 19

Hydrosilylation reaction (further functionalization) 19

X-RAY CRYSTALLOGRAPHY 21

PXRD Analysis 26

MECHANISTIC STUDIES 44

NMR studies 44

IR studies 45

Poisoning tests 46

Kinetic profile 47

CHARACTERIZATION DATA FOR ALL PRODUCTS 58

Elemental analysis of obtained manganese complexes 58

$Mn(CO)_3Br(L1)$ (C18) 63

$Mn(CO)_3Br(L16)$ (C19) 65

Mn(CO)3Br(L9) (C21) 65

$Mn(CO)_3Br(L14)$ (C22) 65

1,1,1-triethyl-3-phenyldisiloxan (1) 67

1-(tert-butyl)-1,1-dimethyl-3-phenyldisiloxane (2) 67

1,1,1-triisopropyl-3-phenyldisiloxane (3) 67

1,1-dimethyl-1,3-diphenyldisiloxane (4) 68

1,1,1-trimethyl-3,3-diphenyldisiloxane (5) 68

1-(tert-butyl)-1,1-dimethyl-3,3-diphenyldisiloxane (6) 68

1-(tert-butyl)-1,1,3-trimethyl-3-phenyldisiloxane (7) 69

1-(tert-butyl)-3,3-bis(4-fluorophenyl)-1,1-dimethyldisiloxane (8) 69

1-(tert-butyl)-3-hexyl-1,1-dimethyldisiloxanez (9) 69

1,1,1,5,5,5-hexaisobutyl-3-phenyltrisiloxane (10) 70

1,1,5,5-tetramethyl-1,3,5-triphenyltrisiloxane (11) 70

1,1,1,5,5,5-hexamethyl-3-phenyltrisiloxane (12) 70
1,1,1,5,5,5-hexaethyl-3-phenyltrisiloxane (13) 71
1,5-di-tert-butyl-1,1,5,5-tetramethyl-3-phenyltrisiloxane (14) 71
1,1,1,5,5,5-hexaisopropyl-3-phenyltrisiloxane (15) 71
3-phenyl-1,1,1,5,5,5-hexapropyltrisiloxane (16) 72
1,1,1,5,5,5-hexahexyl-3-phenyltrisiloxane (17) 72
1,5-di-tert-butyl-1,1,3,5,5-pentaphenyltrisiloxane (18) 73
1,5-diethyl-1,1,5,5-tetraisopropyl-3-phenyltrisiloxane (19) 73
3,3-diisopropyl-1,5-diphenyltrisiloxane (20) 74
1,1,1,3,5,5,5-heptaphenyltrisiloxane (21) 74
1,1,1,5,5,5-hexaethyl-3-(p-tolyl)trisiloxane (22) 74
1,5-di-tert-butyl-1,1,5,5-tetramethyl-3-(p-tolyl)trisiloxane (23) 75
1,1,1,5,5,5-hexaisopropyl-3-(p-tolyl)trisiloxane (24) 75
1,1,1,5,5,5-hexahexyl-3-(p-tolyl)trisiloxane (25) 76
1,1,1,5,5,5-hexaisobutyl-3-(p-tolyl)trisiloxane (26) 76
1,1,5,5-tetramethyl-1,5-diphenyl-3-(p-tolyl)trisiloxane (27) 77
3,3-diisopropyl-1,5-di-p-tolyltrisiloxane (28) 77
3-hexyl-1,1,1,5,5,5-hexamethyltrisiloxane (29) 77
1,5-di-tert-butyl-3-hexyl-1,1,5,5-tetramethyltrisiloxane (30) 78
trimethoxy(phenyl)silane (31) 78
triethoxy(phenyl)silane (32) 78
bis(pentan-3-yloxy)(phenyl)silane (33) 79
dimethoxydiphenylsilane (34) 79
diethoxy(phenyl)silane (35) 79
phenyl(prop-2-yn-1-yloxy)silane (36) 80
8-phenyl-4,7,9,12-tetraoxa-8-silapentadeca-1,14-diene (37) 80
1,5-di-tert-butyl-3-decyl-1,1,5,5-tetramethyl-3-phenyltrisiloxane (38) 80
1-(tert-butyl)-1,1,3-trimethyl-3-phenyl-3-(2-(trimethoxysilyl)ethyl)disiloxane (39) 81
SPECTRA DATA FOR ALL PRODUCTS 82
$Mn(CO)_3Br(L1)$ (C18) 82
$Mn(CO)_3Br(L16)$ (C19) 84
$Mn(CO)_3Br(L9)$ (C21) 86
$Mn(CO)_3Br(L14)$ (C22) 88
1,1,1-triethyl-3-phenyldisiloxane (1) 90
1-(tert-butyl)-1,1-dimethyl-3-phenyldisiloxane (2) 92
1,1,1-triisopropyl-3-phenyldisiloxane (3) 94

1,1-dimethyl-1,3-diphenyldisiloxane (4) 96
1,1,1-trimethyl-3,3-diphenyldisiloxane (5) 97
1-(tert-butyl)-1,1-dimethyl-3,3-diphenyldisiloxane (6) 99
1-(tert-butyl)-1,1,3-trimethyl-3-phenyldisiloxane (7)..101
1-(tert-butyl)-3,3-bis(4-fluorophenyl)-1,1-dimethyldisiloxane (8) 103
1-(tert-butyl)-3-hexyl-1,1-dimethyldisiloxane (9)...105
1,1,1,5,5,5-hexaisobutyl-3-phenyltrisiloxane (10)..107
1,1,5,5-tetramethyl-1,3,5-triphenyltrisiloxane (11) 109
1,1,1,5,5,5-hexamethyl-3-phenyltrisiloxane (12) 111
1,1,1,5,5,5-hexaethyl-3-phenyltrisiloxane (13)..113
1,5-di-tert-butyl-1,1,5,5-tetramethyl-3-phenyltrisiloxane (14) 115
1,1,1,5,5,5-hexaisopropyl-3-phenyltrisiloxane (15)...116
3-phenyl-1,1,1,5,5,5-hexapropyltrisiloxane (16) 117
1,1,1,5,5,5-hexahexyl-3-phenyltrisiloxane (17) 118
1,5-di-tert-butyl-1,1,3,5,5-pentaphenyltrisiloxane (18) 119
1,5-diethyl-1,1,5,5-tetraisopropyl-3-phenyltrisiloxane (19) 121
3,3-diisopropyl-1,5-diphenyltrisiloxane (20) 123
1,1,1,3,5,5,5-heptaphenyltrisiloxane (21) 125
1,1,1,5,5,5-hexaethyl-3-(p-tolyl)trisiloxane (22)..127
1,5-di-tert-butyl-1,1,5,5-tetramethyl-3-(p-tolyl)trisiloxane (23) 129
1,1,1,5,5,5-hexaisopropyl-3-(p-tolyl)trisiloxane (24)...131
1,1,1,5,5,5-hexahexyl-3-(p-tolyl)trisiloxane (25) 133
1,1,1,5,5,5-hexaisobutyl-3-(p-tolyl)trisiloxane (26) 135
1,1,5,5-tetramethyl-1,5-diphenyl-3-(p-tolyl)trisiloxane (27)...137
3,3-diisopropyl-1,5-di-p-tolyltrisiloxane (28)...139
3-hexyl-1,1,1,5,5,5-hexamethyltrisiloxane (29)..141
1,5-di-tert-butyl-3-hexyl-1,1,5,5-tetramethyltrisiloxane (30) 143
trimethoxy(phenyl)silane (31) 145
triethoxy(phenyl)silane (32) 147
bis(pentan-3-yloxy)(phenyl)silane (33)...149
dimethoxydiphenylsilane (34) 151
diethoxy(phenyl)silane (35)..153
phenyl(prop-2-yn-1-yloxy)silane (36) 155
8-phenyl-4,7,9,12-tetraoxa-8-silapentadeca-1,14-diene (37) 157
1,5-di-tert-butyl-3-decyl-1,1,5,5-tetramethyl-3-phenyltrisiloxane (38) 159
1-(tert-butyl)-1,1,3-trimethyl-3-phenyl-3-(2-(trimethoxysilyl)ethyl)disiloxanea (39) 161

REFERENCES 163

GENERAL INFORMATION

Air- and moisture sensitive reactions were carried out under an argon atmosphere using standard Schlenk techniques or a glove box. Solvents used for all experiments were purchased from Honeywell or Sigma Aldrich (Merck), dried over calcium hydride (CaH_2), and purified by distillation. Toluene was additionally dried over sodium. Ligands and Mn-complexes were prepared in accordance with previously reported methods[1,2], using reagents purchased from Sigma Aldrich (Merck) or ABCR GmBH. Silanes, silanols and alcohols were purchased from Sigma-Aldrich, ABCR GmBH, Ambeed, Apollo Scientific or Acros Organics and used as received. Non-commercially available silanols (e.g. triisobutylsilanol, trihexylsilanol, tripropylsilanol, diisopropyl(ethyl)silanol, diisopropylsilanediol, tert-butyldiphenylsilanol) were synthesized from corresponding chlorosilanes by well-known procedures using water and triethylamine while non-commercially available silanes (e.g. p-tolylsilane) were synthesized from corresponding chlorosilanes by well-known procedures using $LiAlH_4$. Reaction progress was monitored by GC chromatography using Agilent 8860 GC and Agilent 5977B GC/MSD with Agilent 8860 GC System by determining the conversion of the limiting reagent using n-dodecane as the internal standard. The structures of products were determined by NMR spectroscopy and mass spectrometry. The ^{1}H NMR (400 MHz), ^{13}C NMR (101 MHz) and ^{29}Si NMR (80 MHz) spectra were recorded on a Bruker Avance III HD NanoBay spectrometer, using chloroform-d1 ($CDCl_3$) or benzene-d6 (C_6D_6) as a solvent. Deuterated solvents were purchased from Deutero GmbH ($CDCl_3$ 99.6 atom% D) or Sigma Aldrich (Merck) (C_6D_6 99.8 atom% D, $CDCl_3$ 99.8 atom% D) and used as received.

OPTIMIZATION STUDIES

Table S1. Structures of obtained ligands.

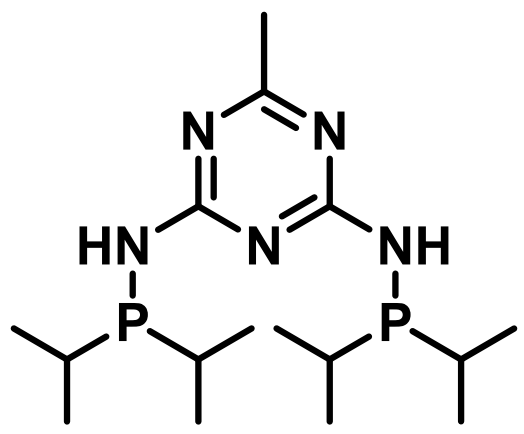 **L1**	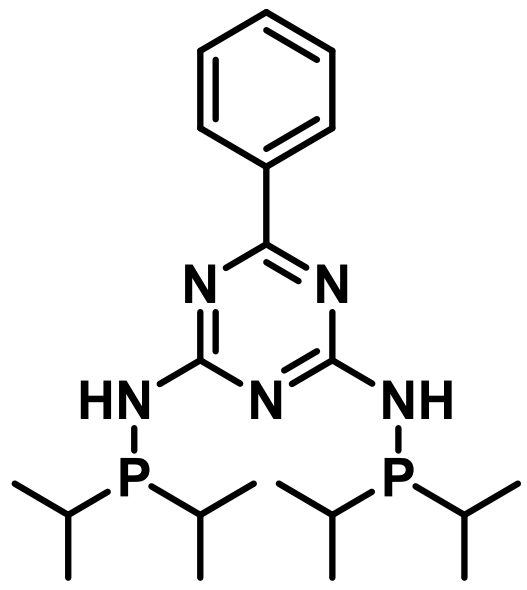 **L2**	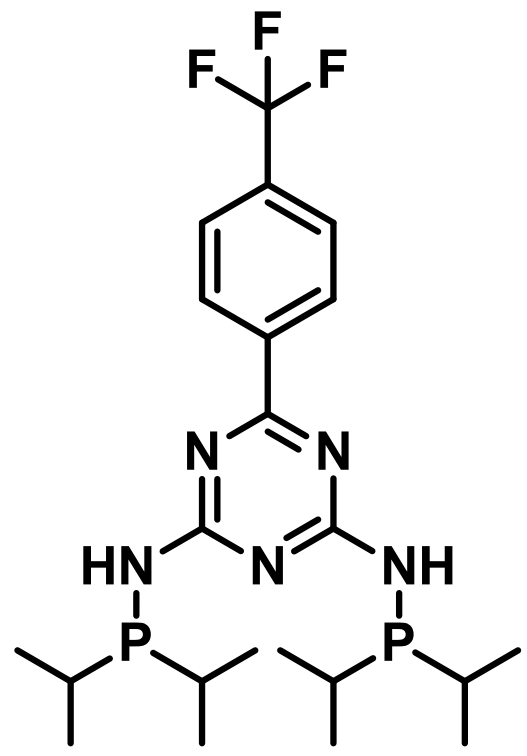 **L3**	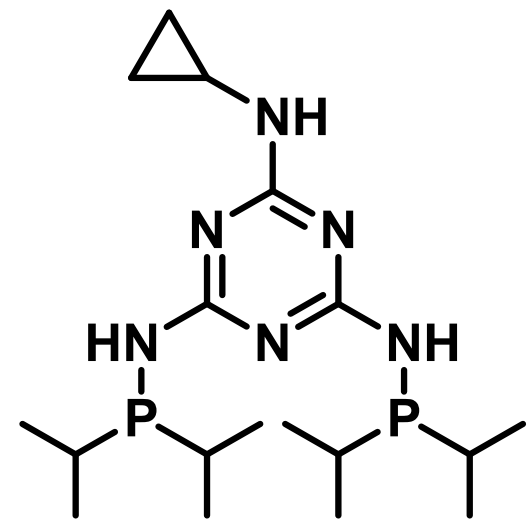 **L4**
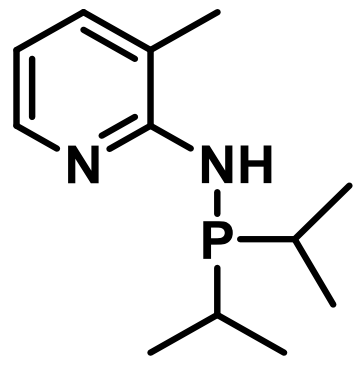 **L5**	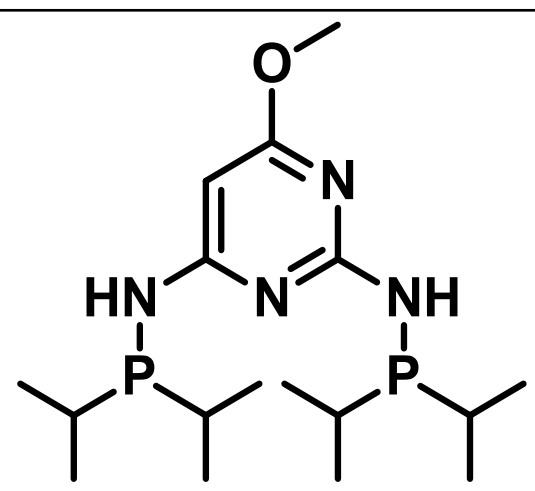 **L6**	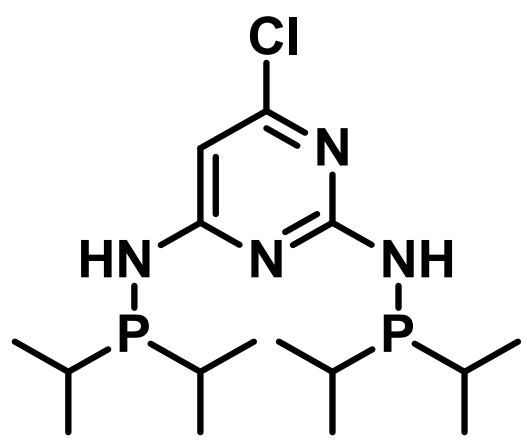 **L7**	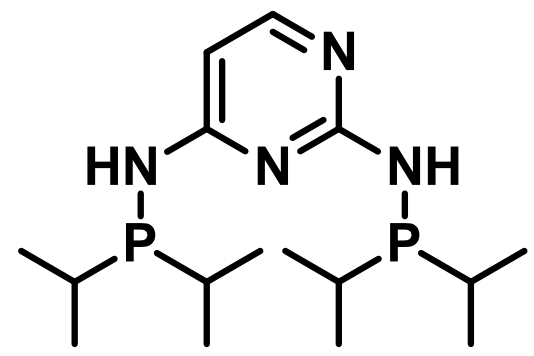 **L8**
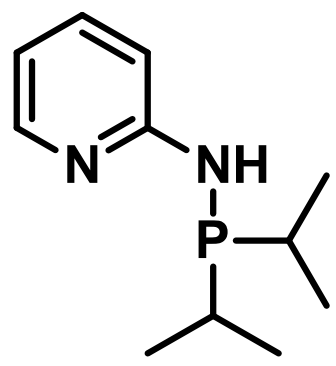 **L9**	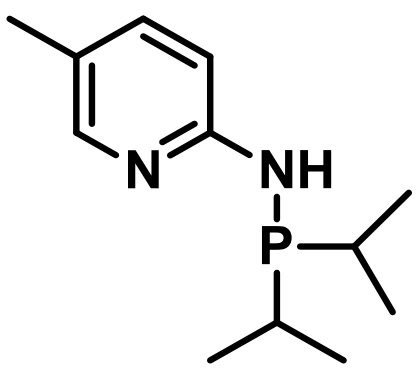 **L10**	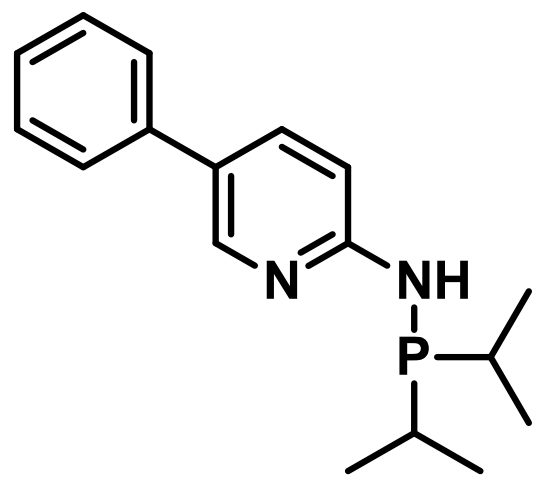 **L11**	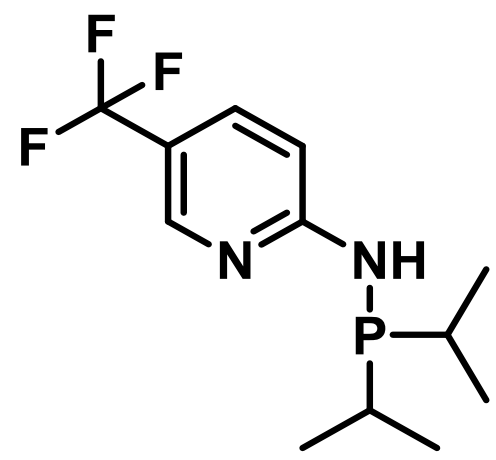 **L12**
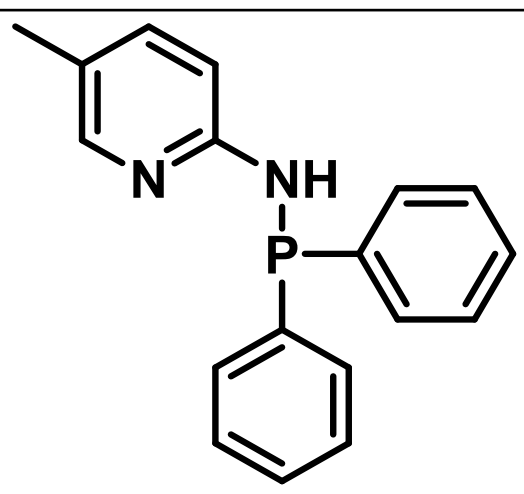 **L13**	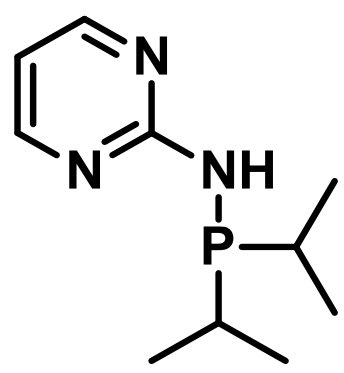 **L14**	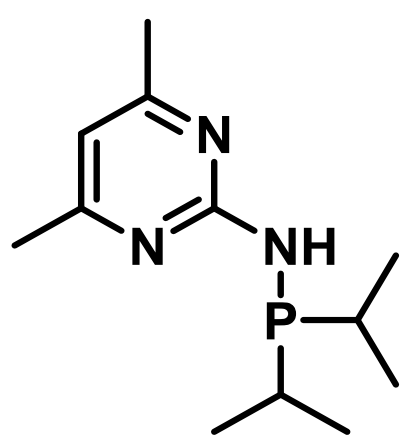 **L15**	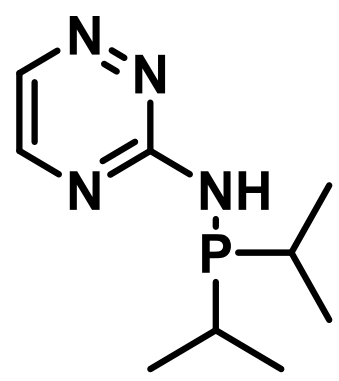 **L16**

Table S2. Structures of obtained complexes.

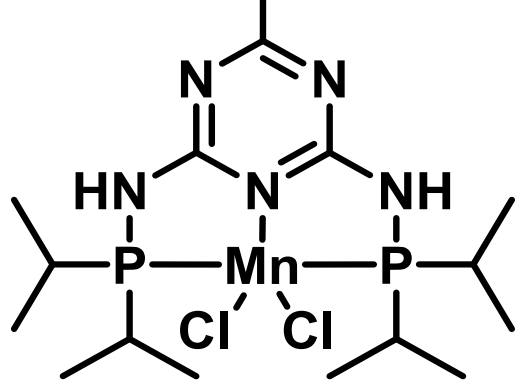 C1	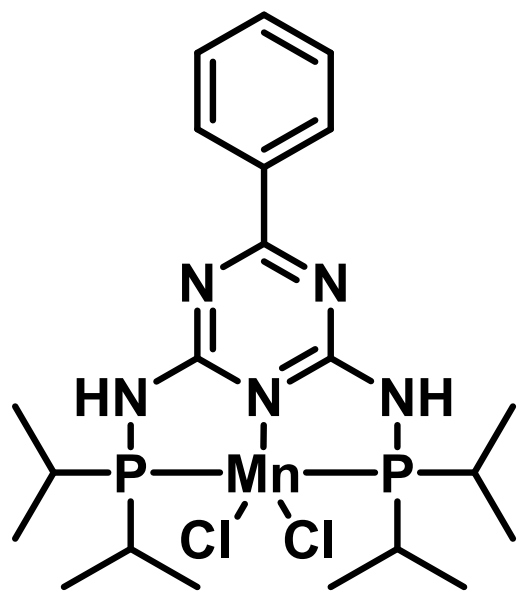 C2	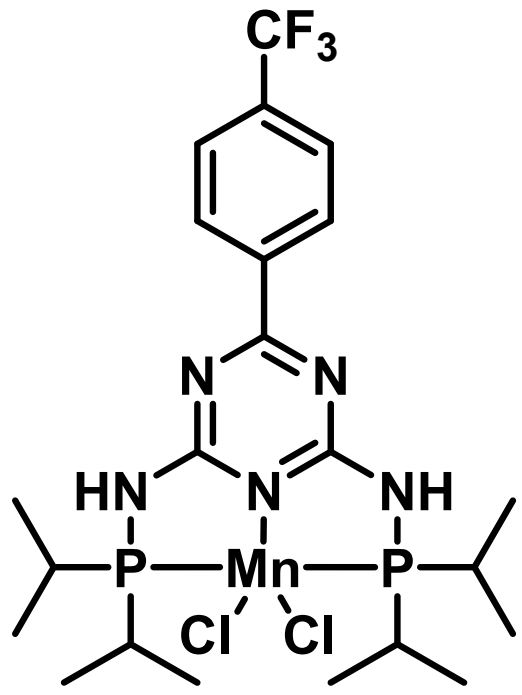 C3	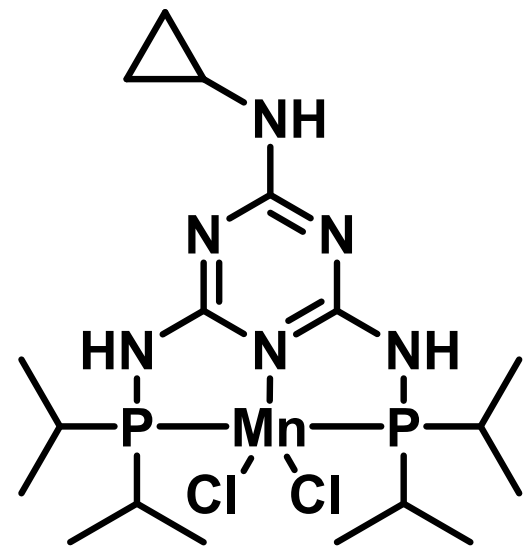 C4
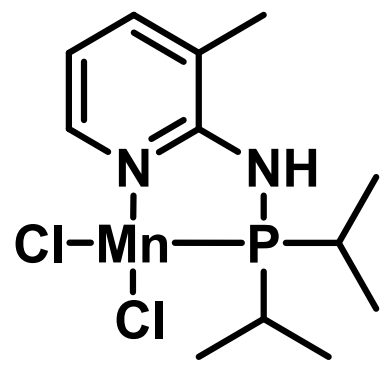 C5	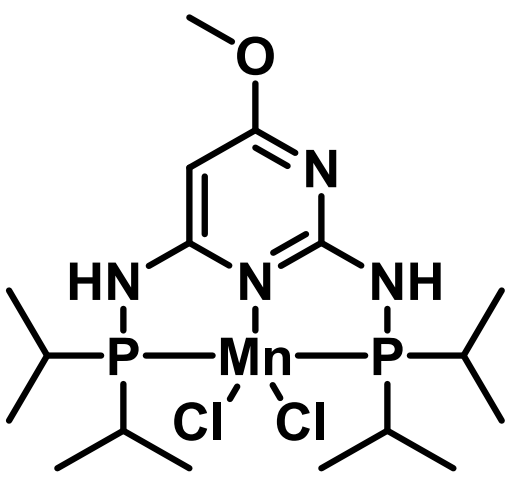 C6	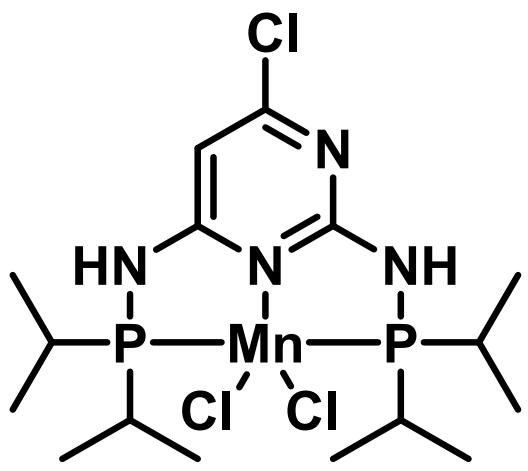 C7	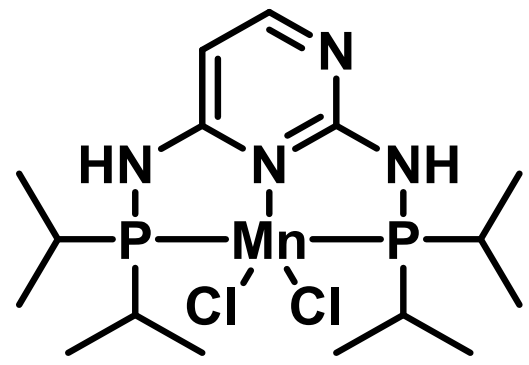 C8
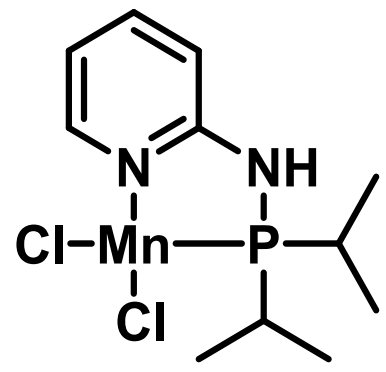 C9	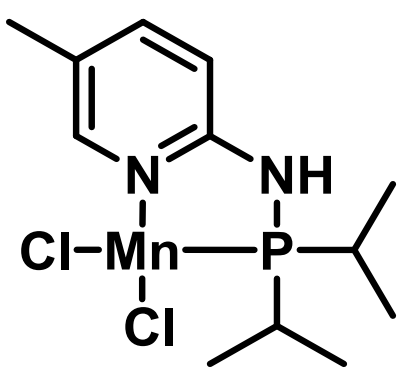 C10	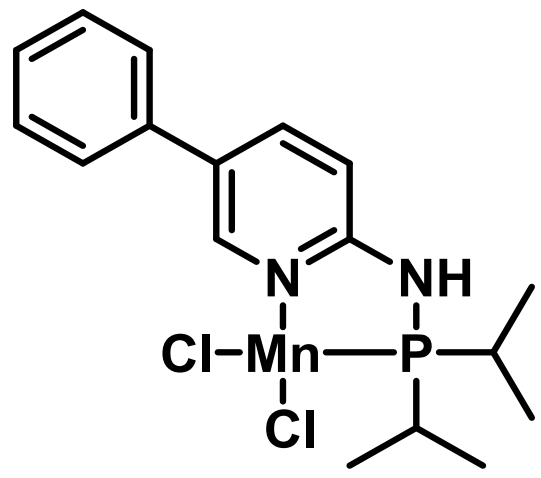 C11	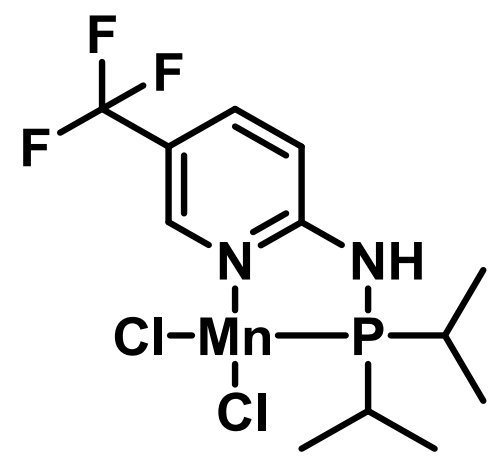 C12
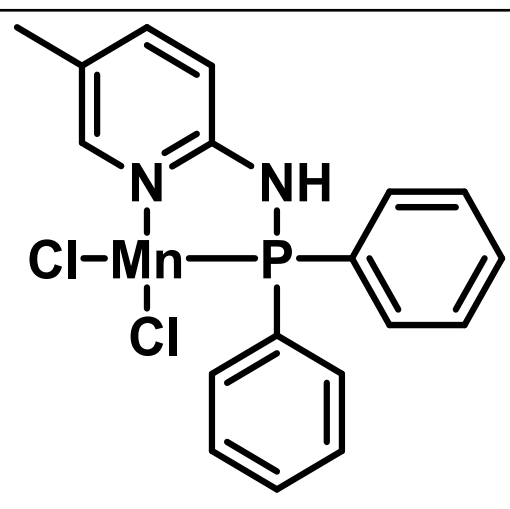 C13	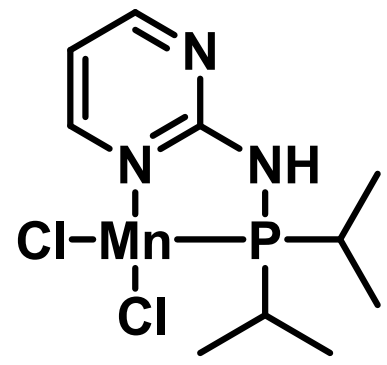 C14	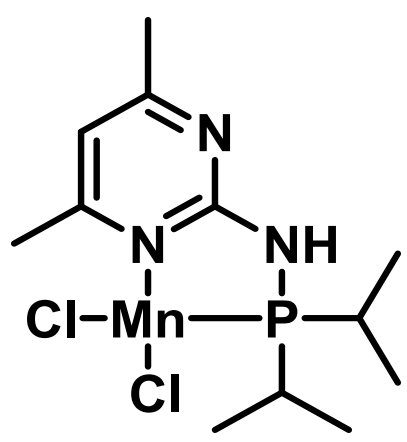 C15	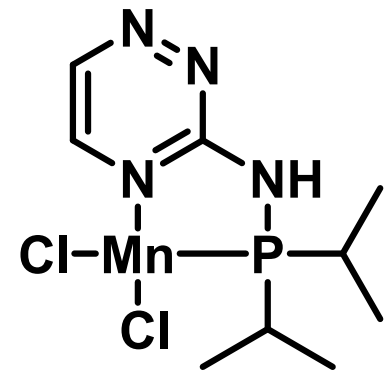 C16
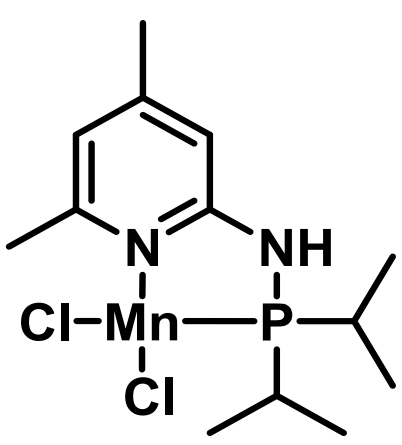 C17	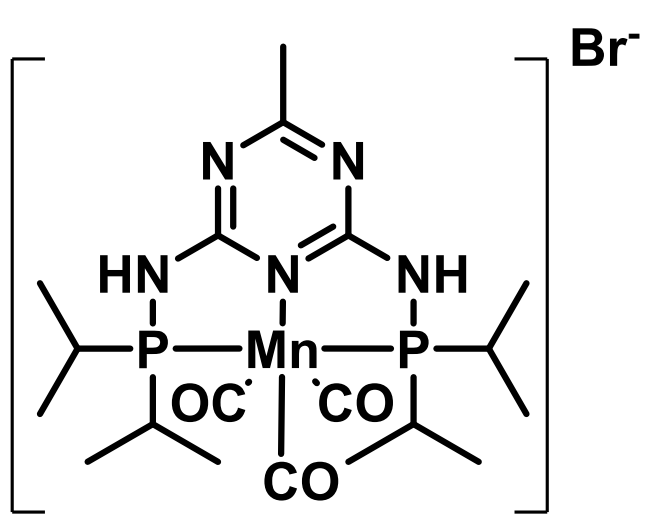 C18	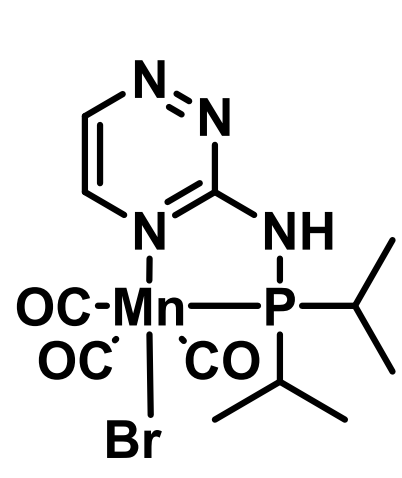 C19	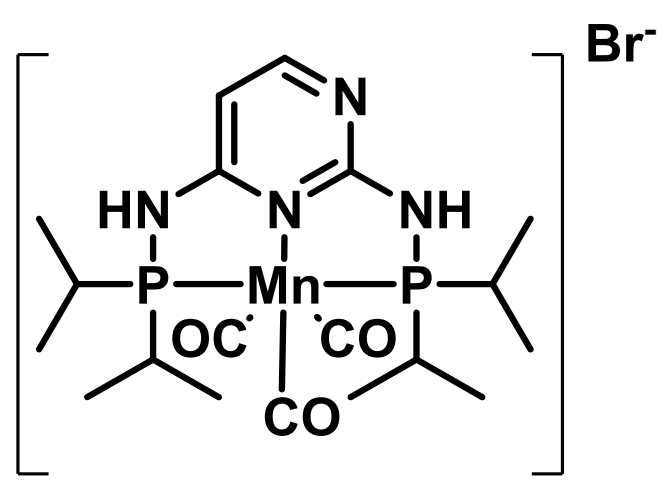 C20
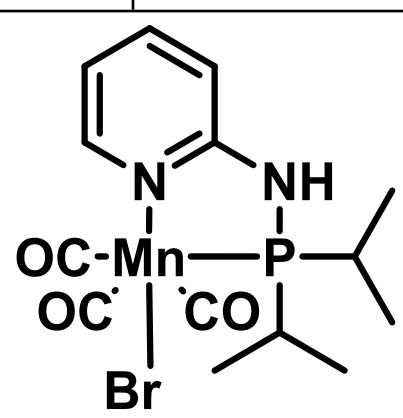 C21		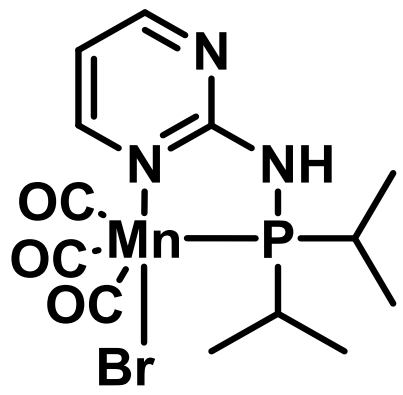 C22	

Table S3. Optimization for di-dehydrogenative coupling.

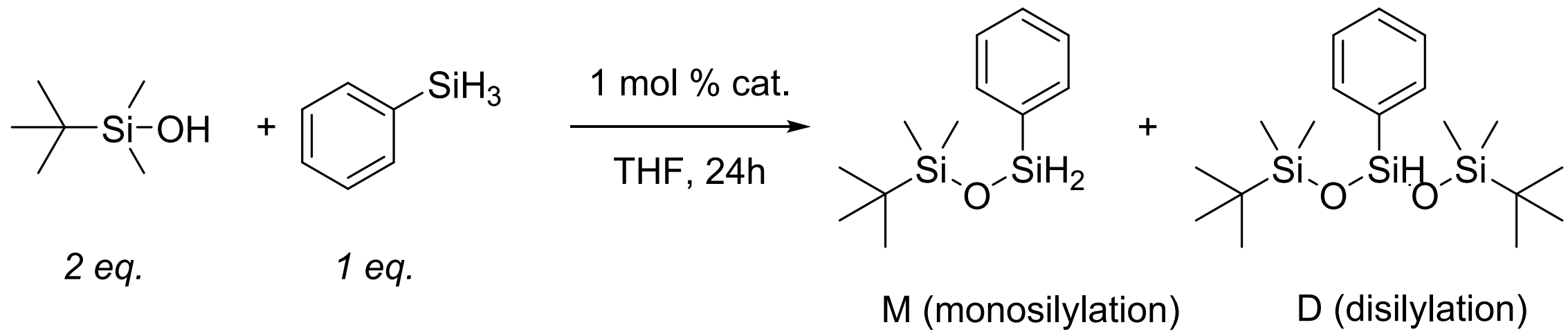

No.	Catalyst	Temperature	Conversion of silane [%][a]	Ratio M/D[b]
1	C1	60°C	99	7:93
2	C2	60°C	100	3:97
3	C3	60°C	100	0:100
4	C4	60°C	99	9:91
5	C5	60°C	100	0:100
6	C6	60°C	100	2:98
7	C7	60°C	100	0:100
8	C8	60°C	100	3:97
9	C9	60°C	100	0:100
10	C10	60°C	100	1:99
11	C11	60°C	100	0:100
12	C12	60°C	100	0:100
13	C13	60°C	100	0:100
14	C14	60°C	100	0:100
15	C15	60°C	100	0:100
16	C16	60°C	100	0:100
17	C17	60°C	100	0:100
18	C18	60°C	94	18:85
19	C19	60°C	100	0:100
20	C20	60°C	98	7:93
21	C21	60°C	100	0:100
22	C22	60°C	100	0:100
23	C1	RT	97	17:83
24	C2	RT	99	12:88
25	C3	RT	100	9:91
26	C4	RT	93	20:80
27	C5	RT	100	0:100
28	C6	RT	98	14:86
29	C7	RT	99	7:93
30	C8	RT	98	15:85
31	C9	RT	100	2:98
32	C10	RT	100	1:99
34	C11	RT	100	1:99
35	C12	RT	100	5:95
36	C13	RT	100	4:96
37	C14	RT	99	3:97
38	C15	RT	100	4:96
39	C16	RT	100	7:93

40	C17	RT	97	16:84
41	C18	RT	63	52:48
42	C19	RT	100	14:86
43	C20	RT	86	26:74
44	C21	RT	89	19:81
45	C22	RT	87	20:80

[a] Conversion of phenylsilane determined by GC with n-dodecane as the internal standard. [b] Ratio between M and D determined by GC.

Table S4. Optimization for mono-dehydrogenative coupling.

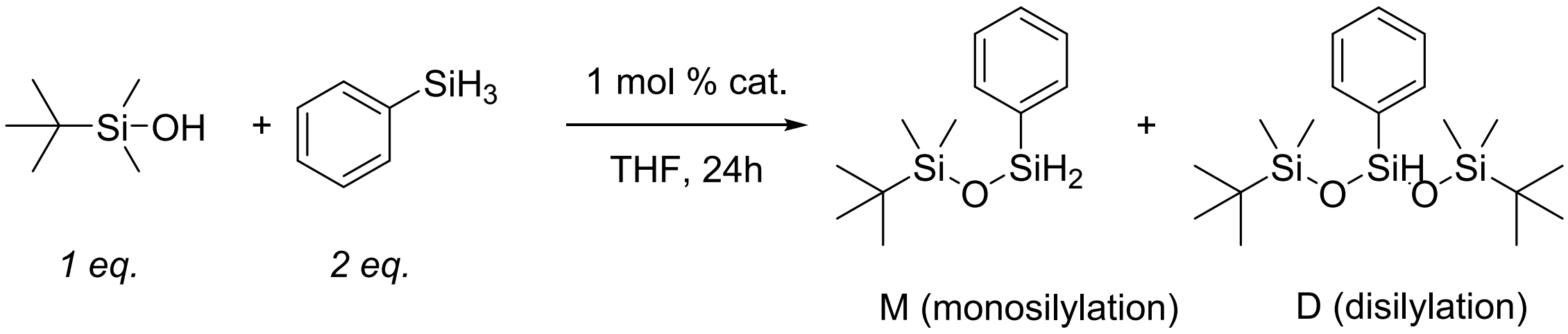

No.	Catalyst	Temperature	Conversion of silanol [%][a]	Ratio M/D[b]
1	C5	60°C	100	74:26
2	C9	60°C	50	78:22
3	C10	60°C	100	82:18
4	C11	60°C	100	90:10
5	C12	60°C	100	88:12
6	C13	60°C	100	86:14
7	C14	60°C	100	85:15
8	C15	60°C	100	90:10
9	C18	60°C	12	100:0

[a] Conversion of tert-butyldimethylsilanol determined by GC with n-dodecane as the internal standard. [b] Ratio between M and D determined by GC.

Table S5. Optimization for mono-dehydrogenative coupling.

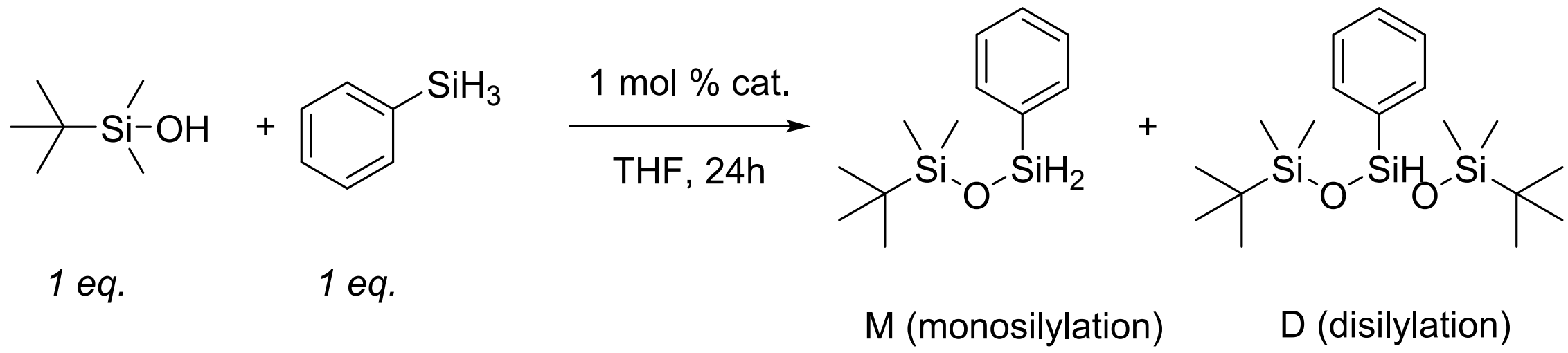

No.	Catalyst	Temperature	Conversion of silanol [%][a]	Ratio M/D[b]
1	C5	RT	100	44:56
2	C9	RT	100	47:53
3	C10	RT	100	44:56
4	C11	RT	100	31:69
5	C12	RT	100	58:42
6	C13	RT	100	58:42
7	C14	RT	100	43:57
8	C15	RT	100	53:47
9	C18	RT	24	90:10
10	C5	0°C	100	50:50
11	C9	0°C	100	39:61
12	C10	0°C	100	41:59
13	C11	0°C	100	56:44
14	C12	0°C	100	52:48
15	C13	0°C	100	50:50
16	C14	0°C	100	32:68
17	C15	0°C	100	52:48
18	C18	0°C	61	72:28

[a] Conversion of tert-butyldimethylsilanol determined by GC with n-dodecane as the internal standard. [b] Ratio between M and D determined by GC.

TURNOVER NUMBER (TON) AND TURNOVER FREQUENCY (TOF)

State of the Art

Table S6. Comparison of TON and TOF values of selected catalytic systems.

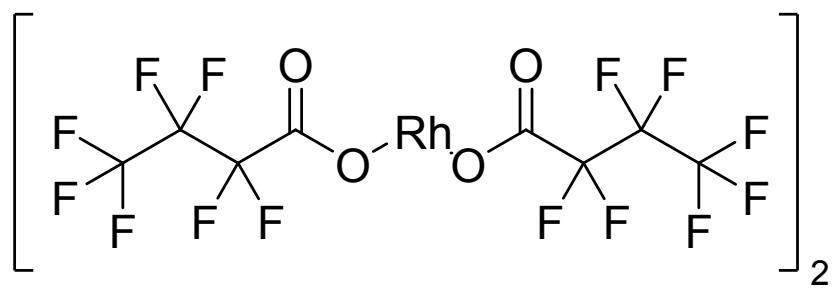

[Rh-1]

$PdCl_2$

[Pd-1]

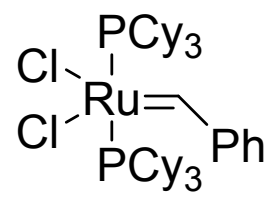

[Ru-1]

$Ru_3(CO)_{12}$

[Ru-2]

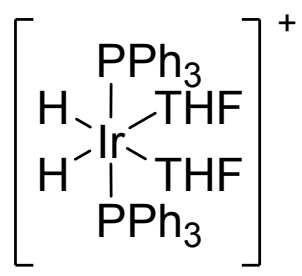

SbF_6^-

[Ir-1]

$AuCl(PPh_3)$

[Au-1]

AuCl(xantphos)

[Au-2]

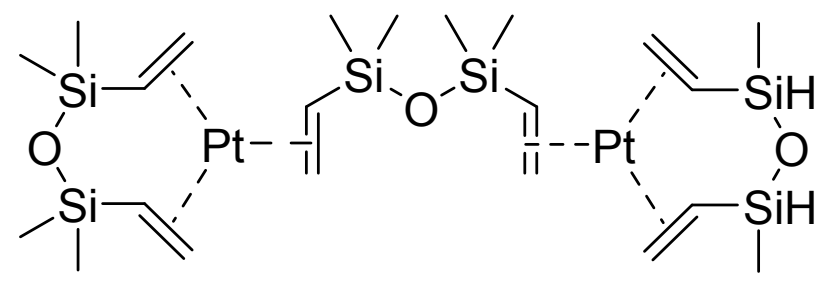

[Pt-1]

$HReO_4$

[Re-1]

$[Mn(CO)_4Br]_2$

[Mn-1]

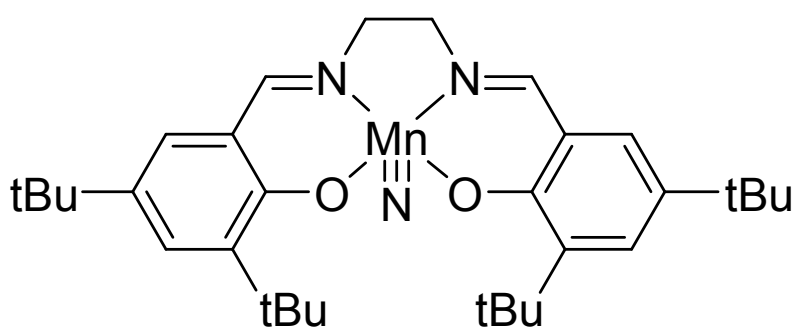

[Mn-2]

$[Mn(CO)_5]_2$

[Mn-3]

$[CpFe(CO)_2]_2$

[Fe-1]

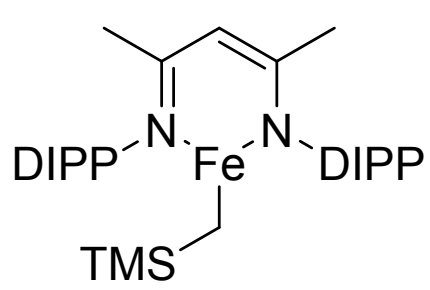

[Fe-2]

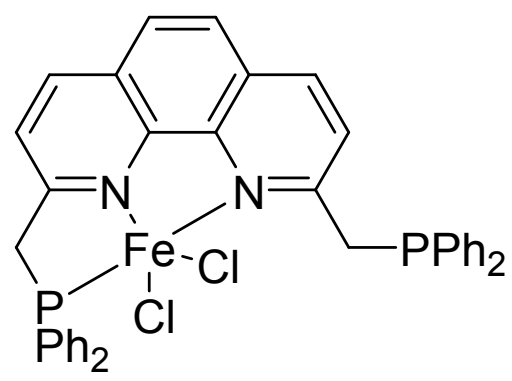

[Fe-3]

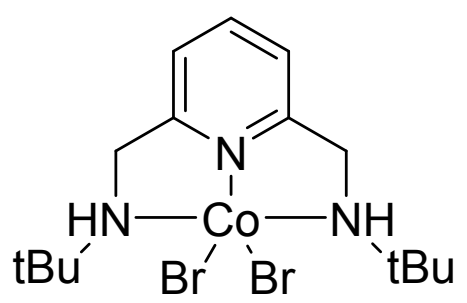

[Co-1]

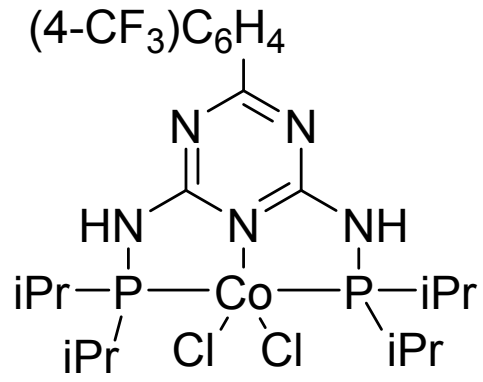

[Co-2]

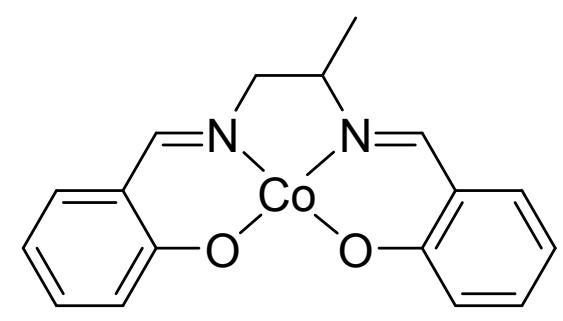

[Co-3]

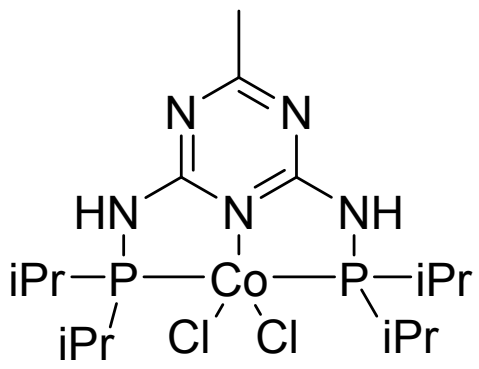

[Co-4]

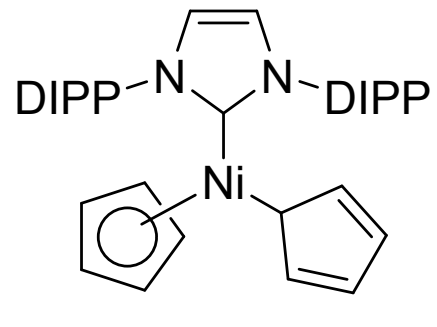

[Ni-1]

$Cu(OTf)_2$

[Cu-1]

$Cu(SbF_6)_2$

[Cu-2]

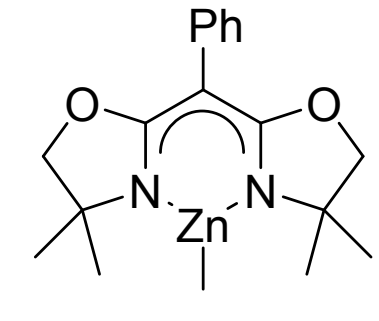

[Zn-1]

Catalyst	Type of Hydrosilane	Type of O–Nucleophile	Solvent, T, time	Cat. loading, additive	Yields	Highest TON	Highest TOF [h^{-1}]
			**Selected Precious metal examples**				

[Rh-1][3]	3°	1° and 2° alcohols	CH_2Cl_2, r.t., 0.5-22h	1-5 mol%	88-99%	99	184
[Pd-1][4]	3°	1°, 2°, and 3° alcohols	ethanol, r.t, 1-4h	0.2-0.4 mol%	65-92%	395	395
[Ru-1][5]	2° and 3°	1° and 2° alcohols	neat, r.t.-55°C, 0.25-8h	0.5 mol%	75->95%	198	792
[Ru-2][6]	2° and 3°	POSS mono- and disilanols	toluene, 120°C, 24h	4-8 mol%	84-98%	24.5	1
[Ir-1][7]	1°, 2°, and 3°	1° and 2° alcohols	CH_2Cl_2, r.t., 15s-11min	0.2-0.4 mol%	>90%	500	130000
[Au-1][8]	1° and 2°	silanols	THF, r.t-70°C, 13h	1-2.5 mol% cat., 2.5-7.5 mol% PPh_3/$PnBu_3$	75-95%	95	7.3
[Au-2][9]	3°	1°, 2°, and 3° alcohols	EDC, 50-80°C, 0.5-95h	1-5 mol%	80-99%	99	98
[Pt-1][10]	3°	1° alcohols	neat, r.t-80°C, 0.5-10	0.015 mol%	50-99%	6600	13 200
[Re-1][11]	3°	1° alcohols	neat, r.t., 24h	5 mol%	93-96%	19.2	0.8
**Selected Earth-abundant metal examples**							
[Mn-1][12]	3°	1°, 2°, and 3° alcohols	C_6H_6, r.t., 0.17-3h	0.084 mol%	36->95%	1131	6653
[Mn-2][13]	2° and 3°	1°, 2°, and 3° alcohols	CD_3CN, 80°C, 0.67-8.5h	0.5 mol%	50-99%	198	295.5
[Mn-3][14]	2° and 3°	2° alcohols	toluene, 140°C, 0.5-24h	5 mol% cat., 7.5 mol% tri(2-furyl)phosphine oxide	58-99%	20	38.4
[Fe-1][15]	1° and 2°	1° and 2° alcohols	C_6D_6, r.t., 24h, hv	2 mol%	91-99%	50	2.1
[Fe-2][16]	1° and 2°	1°, 2°, and 3° alcohols	C_6D_6, r.t., 24h	5 mol%	75-99%	20	0.8
[Fe-3][17]	1° and 2°	silanols	THF, r.t., 12h	0.1 mol% cat., 0.2 mol% $NaHBEt_3$	92-99%	990	82.5
[Co-1][18]	1° and 2°	silanols	THF, r.t., 1h, 4 mol% tBuOK	2 mol%	73-88%	44	44
[Co-2][19]	1° and 2°	silanols and POSS mono- and disilanols	THF, r.t., 20h	0.5-4 mol%	60-98%	196	9.8

[Co-3][20]	1° and 2°	silanols, 1°, 2°, and 3° alcohols	toluene, 60°C, 20h	0.5 mol%	71-98%	190	9.5
[Co-4][21]	1° and 2°	1°, 2°, and 3° alcohols	THF, 40°C, 22h	0.5-2 mol%	68-91%	182	8.3
[Ni-1][22]	3°	1° and 2° alcohols	toluene, 110°C, 1-24h	1-4 mol%	56-99%	99	99
[Cu-1][23]	1°, 2°, and 3°	POSS mono- and disilanols	toluene, 80°C, 24h	4-8 mol%	80-98%	24.5	1
[Cu-2][24]	1°	1°, 2°, and 3° alcohols	CH_2Cl_2, 0°C, 0.75h	1.5-5 mol%	71-95%	63.3	84.4
[Zn-1][25]	1° and 2°	1°, 2°, and 3° alcohols	benzene/neat, r.t. <10min-24h	0.00001-10mol%	63-99%	$1x10^7$	$1x10^7$

Present Work: Manganese-Catalyzed Dehydrogenative Coupling

Table S7. Optimization of TON and TOF for dehydrogenative coupling with use of C15 between *t*-$BuMe_2SiOH$/MeOH and $PhSiH_3$.

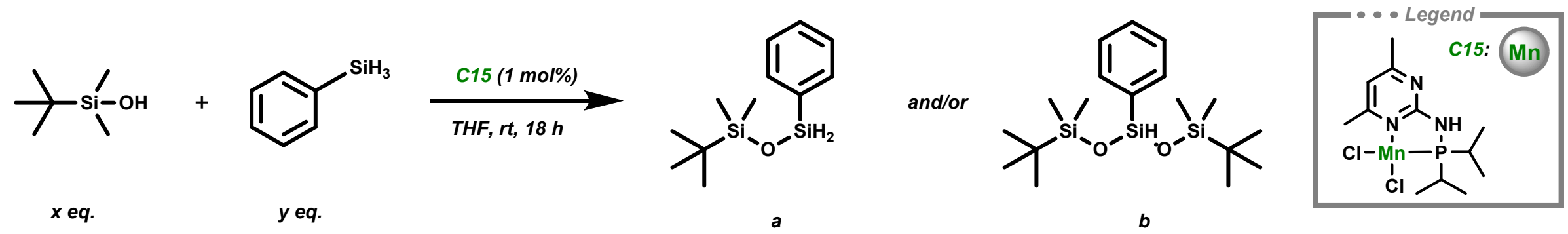

No.	Initial conditions	X (eq.)	Y (eq.)	Conversion[a] (%)	Selectivity a:b	TON[c]	TOF [h^{-1}]
				**Monocoupling**			
1	No change	1	2	99	90:10	89	4.9
2	0.5mol%	1	2	99	90:10	178	9.9
3	0.1mol%	1	2	73	91:9	664	36.9
4	0.5mol%, neat	1	2	99	69:31	137	7.6
5	0.1mol%, neat	1	2	99	71:29	703	39.1
**6**	**0.05mol%, neat**	**1**	**2**	**68**	**100:0**	**1 360**	**75.6**

No.	Initial conditions	X (eq.)	Y (eq.)	Conversion[b] (%)	Selectivity a:b	TON[c]	TOF [h^{-1}]
				**Dicoupling**			
7	No change	2	1	99	4:96	95	5.3
8	1 h, 60°C	2	1	94	1:99	94	94
9	0.5 mol%, 60°C	2	1	99	4:96	190	10.6
10	0.1mol%, 60°C	2	1	98	0:100	980	54.4
11	0.5 mol%, neat, 60°C	2	1	99	0:100	198	11
12	0.1 mol%, neat, 60°C	2	1	99	0:100	990	55
13	0.01 mol%, neat, 60°C, 1h	2	1	90	22:78	7 020	7 020

**14**	**0.001 mol%, neat, 60°C, 1h**	**2**	**1**	**80**	**41:69**	**55 200**	**55 200**

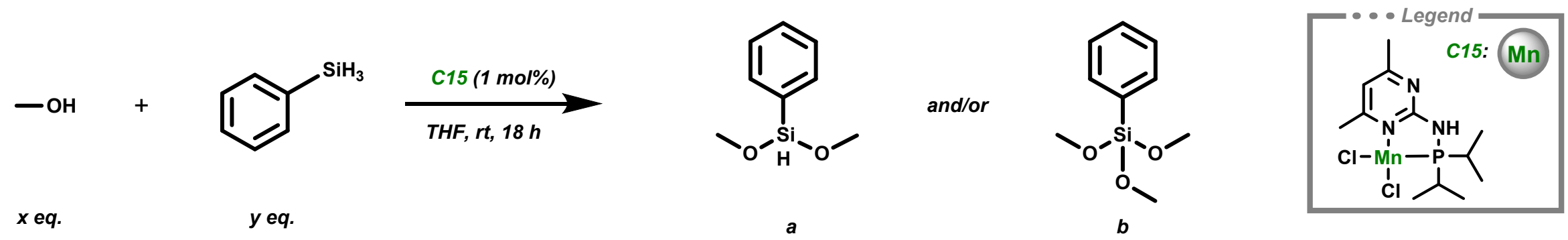

No.	Initial conditions	X (eq.)	Y (eq.)	Conversion[b] (%)	Selectivity a:b	TON[c]	TOF [h^{-1}]
				**Silylether**			
15	neat	30	1	99	0:100	99	5.5
16	neat, 1h	30	1	99	0:100	99	99
17	0.01 mol%, neat, 1h	30	1	99	0:100	9 900	9 900
18	0.01 mol%, neat, 0.25h	30	1	99	0:100	9 900	39 600
19	0.001 mol%, neat, 1h	30	1	99	0:100	99 000	99 000
**20**	**0.001 mol%, neat, 1h**	**30**	**1**	**99**	**0:100**	**99 000**	**99 000**
**21**	**0.00001 mol% neat, 1h**	**30**	**1**	**0**	**-**	**-**	**-**

a - conversion (%) of silanol determined by GC with n-dodecane as the internal standard; b - conversion of silane determined by GC with n-dodecane as the internal standard; c - TON calculated for the product with highest selectivity; d - TON calculated for the monocoupled product; e - TON calculated for the dicoupled product; f - TON calculated for the tricoupled product;

Table S8. Optimization of TON and TOF for dehydrogenative coupling with use of $MnCl_2$ between *t*-$BuMe_2SiOH$/MeOH and $PhSiH_3$.

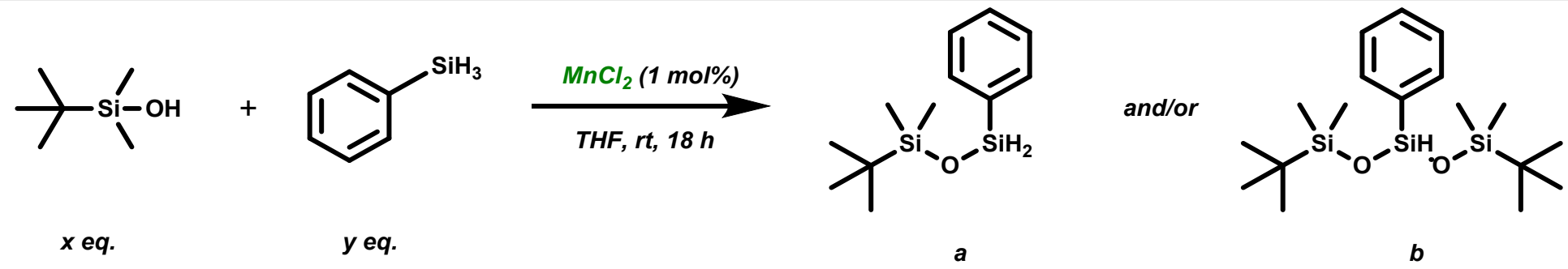

No.	Initial conditions	X (eq.)	Y (eq.)	Conversion[b] (%)	Selectivity a:b	TON	TOF [h^{-1}]
				Mono- and dicoupling			
21	0.05 mol%, neat	1	2	70	80:20	1 120[d]	62.2
22	0.01 mol%, neat, 60°C	2	1	11	100:0	-[e]	-

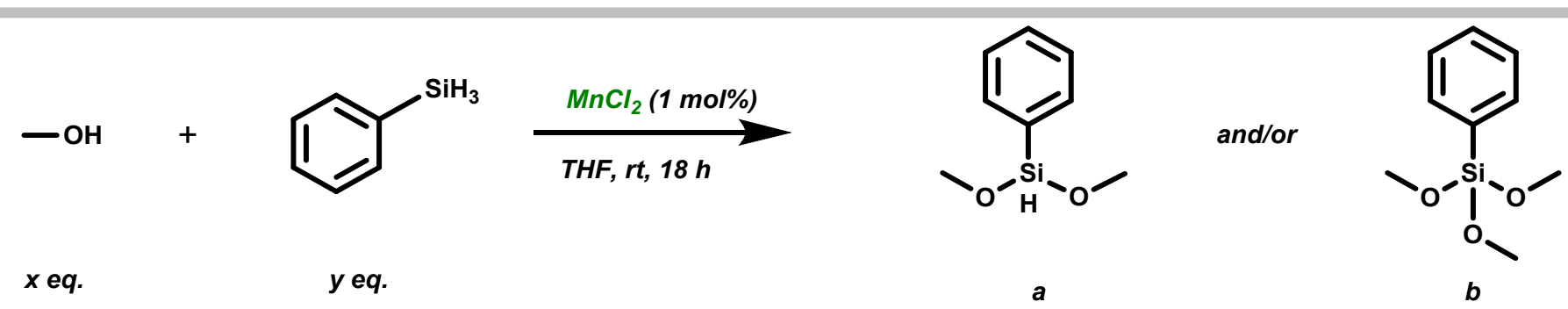

No.	Initial conditions	X (eq.)	Y (eq.)	Conversion[b] (%)	Selectivity a:b	TON[c]	TOF [h^{-1}]
				Silylether			
23	0.001 mol%, neat, 1h	30	1	64	93:7	4 480[f]	4 480
24	0.001 mol%, neat, 0.25h	30	1	36	100:0	-[f]	-
25	**0.00001 mol% neat, 0.25h**	**30**	**1**	**0**	**-**	**-**	**-**

a - conversion (%) of silanol determined by GC with n-dodecane as the internal standard; b - conversion of silane determined by GC with n-dodecane as the internal standard; c - TON calculated for the product with highest selectivity; d - TON calculated for the monocoupled product; e - TON calculated for the dicoupled product; f - TON calculated for the tricoupled product;

EXPERIMENTAL PROCEDURES

Synthesis of ligands

The reaction was performed in accordance with previously reported methods.[2] To a solution of 2-aminopirydine (3-methylpyridin-2-amine, 4-methylpyridin-2-amine etc., 1 eq., 0.02 mol) in dry THF was added Et_3N (2-4 eq., 0.04-0.08 mol) under an argon atmosphere (Schlenk technique). The resulting mixture was cooled to 0°C, and $P(iPr)_2Cl$ (1.2-2.4 eq., 0.024-0.048 mol) was added dropwise with vigorous stirring. The reaction mixture was heated at 50°C for 24 hours. After this time, the solution was filtered from the resulted $Et_3N{\cdot}HCl$, and the solvent was removed under vacuum. The remaining crude product was recrystallized from hot toluene.

Synthesis of Mn-based complexes

To oven-dried Schlenk flask $MnCl_2$ or $Mn(CO)_5Br$ (1 eq., 1 mmol), aminophospine (**L1**-**L16**, 1 eq., 1 mmol) and THF (8 mL) were added under argon atmosphere (glovebox). The reaction mixture was heated at 50°C and vigorously stirred for 24 hours. After this time volatiles were evaporated under vacuum. Obtained compounds were then washed with cold dry hexane (2x 5 mL) and afterwards dried under vacuum again. The complexes that yielded crystals suitable for X-ray diffraction were obtained by crystallization from dry THF.

Dehydrogenative coupling of silanols/alcohols and silanes

To a 12 mL vial equipped with a magnetic stirring bar under an inert atmosphere (glovebox) the catalyst (**C15**, 1 mol%; or $MnCl_2$ 1 mol%), dry THF (500 uL), silanol/alcohol (0.25 mmol) and silane (depending on desired product: 0.50 mmol for monoalkoxysilane; 0.125 mmol for dialkoxysilane) were added. The reaction mixture was stirred at room temperature (or 60°C) for 24 hours. After this time, solvent and residue were evaporated under reduced pressure. Obtained products were dissolved in hexane and isolated *via* filtration. The pure products were identified by ^1H and ^{13}C NMR spectroscopies and MS.

Gram-scale reaction

To a 50 mL Schlenk vessel equipped with a magnetic stirring bar under an inert atmosphere (glovebox) the catalyst (**C15**, 1 mol%), silanol (10 mmol) and silane (5 mmol) were added. The reaction mixture was stirred at 60°C for 24 hours. After this time, solvent and residue were evaporated under reduced pressure. Obtained product was dissolved in hexane and isolated *via* filtration. The pure product was identified by ^1H and ^{13}C NMR spectroscopies and MS.

Hydrosilylation reaction (further functionalization)

The reaction was performed using a standard, well-established procedure of hydrosilylation with use of Karstedt catalyst. To a 12 mL vial equipped with a magnetic stirring bar **14** (0.25 mmol), decene (0.25 mmol), dry toluene (500 uL) and Karstedt catalyst (0.03 mol%) were added. The reaction mixture was stirred at 60°C for 24 hours. After this time, solvent and residue were evaporated under reduced

pressure. Obtained product was dissolved in hexane and isolated *via* filtration. The pure product was identified by ^{1}H and ^{13}C NMR spectroscopies and MS.

X-RAY CRYSTALLOGRAPHY

Diffraction data were collected by the ω-scan technique, for C07 and C15 using graphite-monochromated MoKα radiation (λ=0.71073 Å), at 130(1) K on Rigaku XCalibur four-circle diffractometer with EOS CCD detector, for the other compounds at 100(1) K, on a Bruker D8 QUEST KAPPA diffractometer with a microfocus sealed tube using a multilayer mirror as monochromator (CuKα radiation, λ=1.54178 Å) and a Bruker PHOTON III CPAD detector. The data were corrected for Lorentz-polarization as well as for absorption effects. The structures were solved with SHELXT[26] and refined with the full-matrix least-squares procedure on F2 by SHELXL[27]. All non-hydrogen atoms were refined anisotropically. Hydrogen atoms were placed in idealized positions and refined as 'riding model' with isotropic displacement parameters set at 1.2 times Ueq of appropriate carrier atoms.

Crystallographic data for the structural analysis has been deposited with the Cambridge Crystallographic Data Centre. Copies of this information may be obtained free of charge from: The Director, CCDC, 12 Union Road, Cambridge, CB2 1EZ, UK; e-mail: deposit@ccdc.cam.ac.uk, or www: www.ccdc.cam.ac.uk.

Table S9. Crystal data, data collection and structure refinement

Compound	**1 (C1)**	**2 (C2)**	**3 (C4)**	**4 (C6)**
Formula	$C_{16}H_{33}Cl_2MnN_5P_2 \cdot 2(C_4H_8O)$	$2(C_{21}H_{35}Cl_2MnN_5P_2) \cdot 3(C_4H_8O)$	$C_{18}H_{36}Cl_2MnN_6P_2 \cdot C_4H_8O$	$C_{17}H_{34}Cl_2MnN_4OP_2 \cdot C_4H_8O$
Formula weight	627.46	1306.95	596.41	570.36
Crystal system	triclinic	triclinic	orthorhombic	monoclinic
Space group	P-1	P-1	Pbca	$P2_1/n$
a(Å)	10.6919(4)	13.7175(3)	17.2938(3)	9.1753(2)
b(Å)	16.5669(5)	15.4943(3)	16.3570(2)	14.5562(3)
c(Å)	19.2279(7)	16.7930(4)	20.9271(3)	21.3154(5)
α(º)	83.769(2)	102.3670(10)	90	90
β(º)	85.996(2)	95.4320(10)	90	93.1260(10)
γ(º)	71.987(2)	101.3340(10)	90	90
V(Å^3)	3217.4(2)	3383.39(13)	5919.75(15)	2842.60(11)
Z	4	2	8	4
D_x(g cm^{-3})	1.295	1.283	1.338	1.333
F(000)	1332	1380	2520	1204
μ(mm^{-1})	6.030	5.748	6.512	6.762
Reflections:				
collected	44295	52096	44994	25461
unique (R_{int})	11610 (0.0370)	12224 (0.0412)	5384 (0.0265)	5157 (0.0409)
with I>2σ(I)	10351	11064	5036	4767
R(F) [I>2σ(I)]	0.0515	0.0634	0.0313	0.0419
wR(F^2) [I>2σ(I)]	0.1210	0.1614	0.0361	0.0991
R(F) [all data]	0.0622	0.0720	0.0715	0.0465
wR(F^2) [all data]	0.1313	0.1706	0.0755	0.1019
Goodness of fit	1.056	1.075	1.161	1.057
max/min Δρ (e·Å^{-3})	0.93/-0.69	1.08/-1.20	0.31/-0.32	0.61/-0.51
CCDC deposition number	2523390	2523391	2523392	2523393

Compound	**5 (C7)**	**6 (C13)**	**7 (C15)**	**8 (C17)**
Formula	$C_{16}H_{31}Cl_3MnN_4P_2 \cdot C_4H_8O$	$C_{36}H_{34}Cl_2MnN_4P_2 \cdot C_4H_8O$	$C_{24}H_{44}Cl_4Mn_2N_6P_2 \cdot 2(C_4H_8O)$	$C_{26}H_{46}Cl_4Mn_2N_6P_2 \cdot 2(C_4H_8O)$
Formula weight	574.78	782.55	874.48	872.49
Crystal system	monoclinic	monoclinic	monoclinic	monoclinic
Space group	$P2_1/n$	$P2_1/c$	$P2_1/c$	$P2_1/n$
a(Å)	9.0463(2)	10.3573(7)	9.3661(3)	11.4863(3)
b(Å)	14.7750(5)	14.3723(10)	9.9660(3)	13.9020(4)
c(Å)	21.1146(6)	26.1044(17)	22.9068(7)	13.4915(4)
α (º)	90	90	90	90
β(º)	93.267(2)	99.801(3)	101.223(3)	100.7730(10)
γ (º)α	90	90	90	90
V(Å^3)	2817.57(14)	3829.1(4)	2097.29(11)	2116.39(10)
Z	4	4	2	2
D_x(g cm^{-3})	1.355	1.357	1.385	1.369
F(000)	1204	1628	916	916

μ(mm^{-1})	0.886	5.167	0.969	8.57
Reflections:				
collected	27407	30244	17410	21088
unique (R_{int})	6377 (0.0358)	7005 (0.0406)	4730 (0.0268)	3858 (0.0390)
with I>2σ(I)	5369	6457	4198	3661
R(F) [I>2σ(I)]	0.0374	0.0393	0.0286	0.0703
wR(F^2) [I>2σ(I)]	0.0744	0.1039	0.0632	0.1607
R(F) [all data]	0.0494	0.0458	0.0355	0.0734
wR(F^2) [all data]	0.0796	0.1075	0.0662	0.1629
Goodness of fit	1.042	1.044	1.043	1.039
max/min Δρ (e·Å^{-3})	0.45/-0.29	0.60/-0.46	0.39/-0.31	1.34/-0.71
CCDC deposition number	2523394	2523395	2523396	2523397

Compound	**9 (C20)**	**10 (C21)**	**11 (C22)**
Formula	$C_{19}H_{32}MnN_4O_3P_2^+ \cdot Br^- \cdot CH_4O$	$C_{14}H_{19}BrMnN_2O_3P$	$C_{13}H_{18}BrMnN_3O_3P$
Formula weight	593.32	429.13	430.12
Crystal system	triclinic	orthorhombic	monoclinic
Space group	P-1	Pbcn	P2$_1$/c
a(Å)	13.9682(9)	29.129(2)	14.9781(7)
b(Å)	13.9771(7)	9.9682(7)	9.3621(5)
c(Å)	15.0580(9)	12.0524(10)	12.0458(6)
α(º)	70.648(2)	90	90
β(º)	89.890(2)	90	97.303(2)
γ(º)	69.295(2)	90	90
V(Å^3)	2572.4(3)	3499.6(5)	1675.44(15)
Z	4	8	4
D_x(g cm^{-3})	1.532	1.629	1.705
F(000)	1224	1728	864
μ(mm^{-1})	2.224	3.141	3.282
Reflections:			
collected	46534	29794	21667
unique (R_{int})	9298 (0.0366)	3183 (0.0360)	3068 (0.0329)
with I>2σ(I)	8298	2899	2966
R(F) [I>2σ(I)]	0.0570	0.0291	0.0288
wR(F^2) [I>2σ(I)]	0.1219	0.0726	0.0725
R(F) [all data]	0.0681	0.0341	0.0302
wR(F^2) [all data]	0.1314	0.0764	0.0736
Goodness of fit	1.065	1.076	1.090
max/min Δρ (e·Å^{-3})	0.82/-1.93	0.51/-0.36	0.59/-0.82
CCDC deposition number	2523398	2523399	2523400

Table S10. The values of N1-C-N-P and C-N-P-C torsion angles (°) with s.u’s in parentheses

	**C1_A**	**C1_B**	**C2_A**	**C2_B**	**C4**	**C6**	**C7**
N1-C-N-P	1.2(4)	--7.1(5)	4.4(5)	-1.6(5)	2.2(3)	-1.2(3)	-2.7(2)
	4.0(4)	-2.9(5)	-11.4(5)	2.0(5)	3.5(3)	0.4(3)	-0.2(2)
C-N-P-C	119.2(3)	129.0(3)	116.6(4)	139.0(3)	-140.24(17)	142.8(2)	141.54(16)
	-128.4(3)	-121.1(3)	-134.7(4)	-111.6(3)	110.53(17)	-107.8(2)	-108.77(17)
	130.4(3)	140.5(3)	-96.5	110.8(3)	116.5(2)	-55.17(16)	118.49(14)
	-119.9(3)	-111.1(3)	154.4(3)	-138.7(3)	-135.4(2)	51.81(16)	-132.79(16)

	**C13**	**C15**	**C20_A**	**C20_B**	**C21**	**C22**
N1-C-N-P	-2.2(3)	-4.3(2)	-2.9(6)	-1.0(6)	-7.6(3)	-12.3(3)
	5.3(3)	-4.3(2)	-4.5(6)	-2.0(6)		
C-N-P-C	-135.30(18)	-123.35(13)	133.7(4)	123.3(4)	138.0(2)	-104.0(2)
	120.90(19)	126.91(13)	-116.1(4)	-118.7(4)	-112.3(2)	147.9(2)
	-123.93(17)	-123.35(13)	-123.6(4)	-129.8(4)		
	128.53(18)	126.91(13)	126.2(4)	121.4(4)		

Table S11. Geometrical parameters describing the coordination of Mn centers (Å, °) with s.u.’s in parentheses

	**C1_A**	**C1_B**	**C2_A**	**C2_B**	**C4**	**C6**	**C7**
Mn-N1	2.326(3)	2.351(3)	2.336(3)	2.339(3)	1.2.3491(16)	2.354(2)	2.4095(16)
Mn-Cl	2.3318(9)<br>2.3695(9)	2.3474(10)<br>2.3564(10)	2.3264(12)<br>2.3909(11)	2.3353(11)<br>2.3857(11)	2.3632(6)<br>2.3651(6)	2.3344(8)<br>2.3891(8)	2.3254(6)<br>2.3980(6)
Mn-P	2.6184(10)<br>2.6184(10)	2.6171(9)<br>2.6305(9)	2.6115(12)<br>2.6407(11)	2.5999(11)<br>2.6001(11)	2.6224(6)<br>2.6442(6)	2.6113(7)<br>2.6439(7)	2.5909(6)<br>2.6225(6)
Tau	0.17	0.31	0.016	0.048	0.25	0.21	0.19

	**C13**	**C15**	**C17**
Mn-N1	2.3665(17)<br>2.3443(17)	2.3284(13)	2.309(4)
Mn-Cl	2.4007(6)<br>2.4480(6)	2.3319(5)<br>2.4328(5)<br>2.5670(4)	2.3464(14)<br>2.4015(12)<br>2.6692(13)
Mn-P	2.7033(6)<br>2.8188(6)	2.5730(4)	2.5524(14)
Tau		0.59	0.65
X-Mn-Y	160.97(4)<br>165.75(4)<br>159.92(2)		

	**C20_A**	**C20_B**	**C21**	**C22**
Mn-N1	2.047(4)	2.051(4)	2.090(2)	2.074(2)
Mn-C	1.791(5)<br>1.838(5)<br>1.851(6)	1.799(5)<br>1.838(5)<br>1.863(5)	1.790(3)<br>1.797(3)<br>1.834(3)	1.794(3)<br>1.839(4)<br>1.844(3)
Mn-P	2.2602(13)<br>2.2660(14)	2.2724(14)<br>2.2766(14)	2.3058(8)	2.2974(7)
Mn-Br			2.5222(5)	2.5219(5)
X-Mn-Y	178.19(18)<br>177.1(2)	176.49(19)<br>175.2(2)	173.84(10)<br>172.40(8)	175.40(10)<br>175.78(9)

	162.99(5)	162.36(6)	176.72(8)	175.49(8)

Table S12. Hydrogen bond data (Å, °)

D	H	A	D-H	H···A	D···A	D-H···A
**C1**						
N2A	H2A	O1E	0.88	2.08	2.930(4)	162
N6A	H6A	O1D	0.88	2.00	2.834(4)	157
N2B	H2B	O1F	0.88	2.19	2.904(4)	138
N6B	H6B	Cl2A	0.88	2.34	3.181(3)	161
**C2**						
N2A	H2A	Cl2B	0.88	2.38	3.231(3)	163
N6A	H6A	O1D	0.88	2.06	2.923(4)	168
N2B	H2B	O1E	0.88	1.97	2.849(5)	178
N6B	H6B	Cl1A^{i}	0.88	2.50	3.309(3)	154
**C4**						
N2	H2	O1A	0.84	2.06	2.897(2))	174
N4	H4	Cl1^{ii}	0.81	2.48	3.2801(18)	170
N6	H6	Cl2^{ii}	0.88	2.71	3.4895(17)	148
**C6**						
N2	H2	O1B	0.88	2.01	2.889(3)	174
N6	H6	Cl1^{iii}	0.88	2.49	3.192(2)	137
**C7**						
N2	H2	O1A	0.82	2.03	2.843(2)	174
N6	H6	Cl1^{iv}	0.88	2.44	3.2319(17)	149
**C13**						
N2A	H2A	Cl2^{v}	0.88	2.48	3.3398(18)	168
N2B	H2B	O1C	0.80	2.16	2.960(2)	174
**C15**						
N2	H2	O1A^{vi}	0.82	2.09	2.9039(18)	175
**C17**						
N2	H2	Cl2^{iii}	0.88	2.45	3.293(4)	160
**C20**						
N2A	H2A	O1C	0.88	1.97	2.845(5)	175
N6A	H6A	Br1A^{vii}	0.88	2.46	3.314(4)	164
N2B	H2B	O1D	0.88	1.96	2.818(6)	165
N6B	H6B	Br1B^{vii}	0.88	2.45	3.289(4)	161
O1C	H1C	Br1A	0.84	2.41	3.251(4)	178
O1D	H1D	Br1B^{viii}	0.84	2.40	3.241(4)	175
**C21**						
N2	H2	Br1^{ix}	0.88	2.53	3.333(2)	153
**C22**						
N2	H2	Br1^{x}	0.88	2.59	3.440(2)	163

^{I} -1+x, y, z; ^{ii} -x+1/2, y-1/2, z; ^{iii} -x+3/2, y+1/2, -z+3/2; ^{iv} -x+1/2, y+1/2, -z+1/2; ^{v} -x+1, y-1/2, -z+1/2; ^{vi} -1+x, y, z; ^{vii} -x+1, -y+1, -z+1; ^{viii} x, y, -1+z; ^{ix} x, -1+y, z-1/2; ^{x} x, -y+1/2, z+1/2.

PXRD Analysis

Powder X-ray diffraction (PXRD) data were collected on a Bruker AXS D8 Advance diffractometer using a standard PMMA sample holder or, where indicated, a zero-background sample holder. High-angle scans were performed over the range of 1–10° in 2θ, with a step size of 0.02° in 2θ and a counting time of 3 s per step. The diffraction patterns obtained are presented below.

Importantly, during the preparation and analysis of all complexes, the analyst observed that the samples became increasingly sticky. Although each complex was stored under an argon atmosphere and exposed to the ambient atmosphere only immediately prior to sample preparation, the observed changes suggest that the complexes may have absorbed moisture to varying extents and undergone partial decomposition. Consequently, some degree of alteration of the samples during preparation and analysis cannot be excluded.

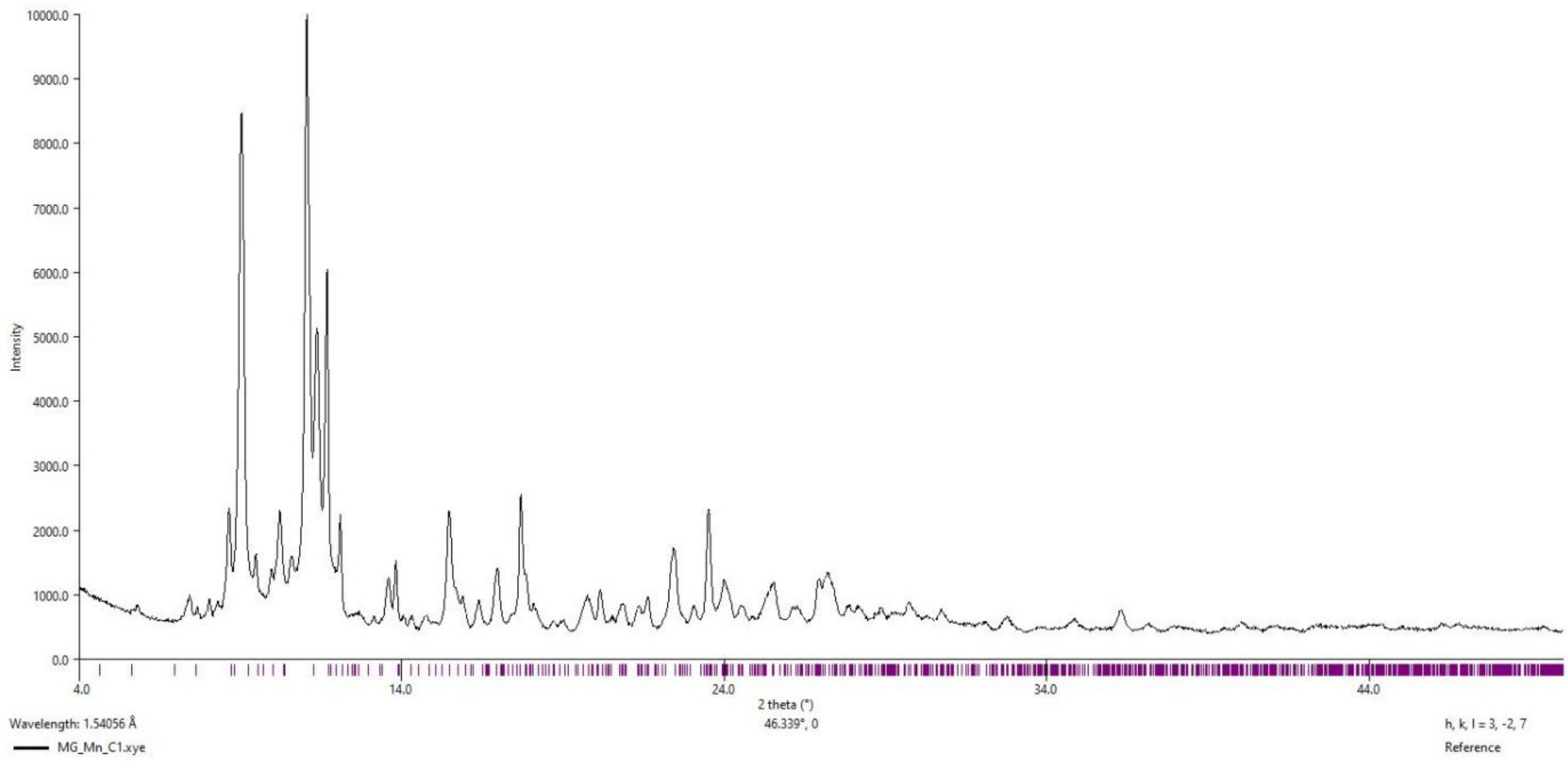

Figure S1. Powder X-ray diffraction (PXRD) pattern of complex C1.

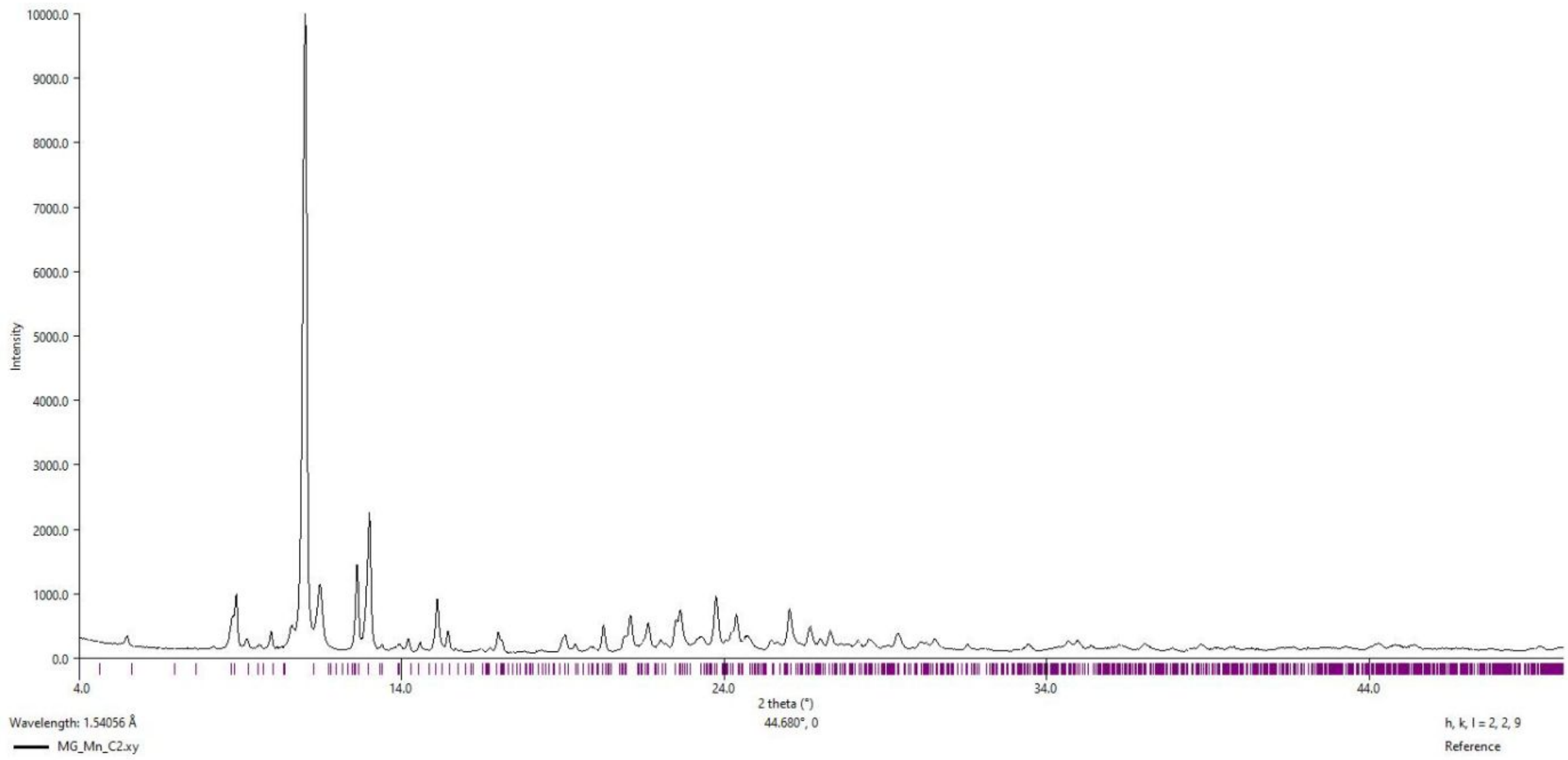

Figure S2. Powder X-ray diffraction (PXRD) pattern of complex C2.

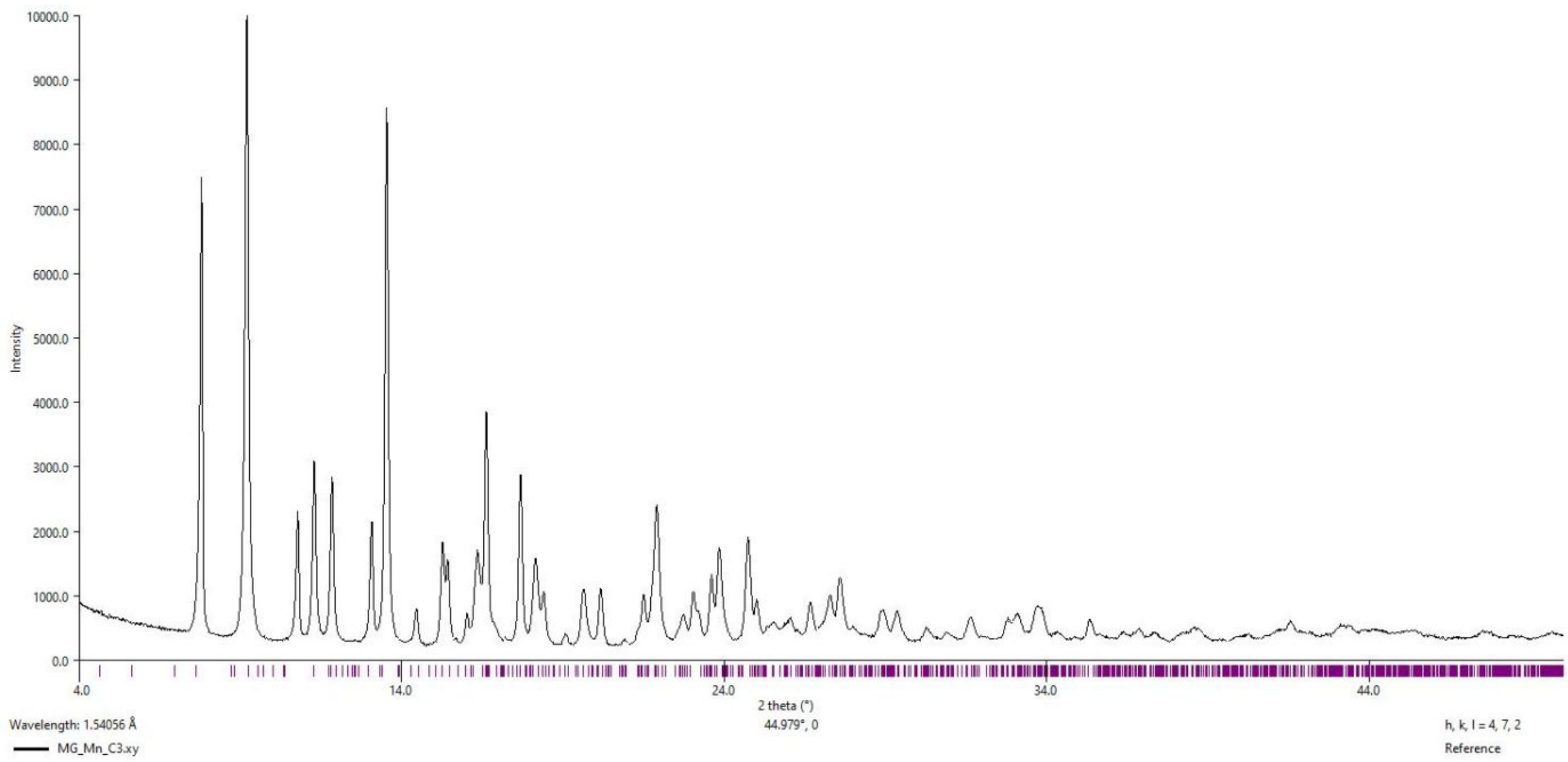

Figure S3. Powder X-ray diffraction (PXRD) pattern of complex C3.

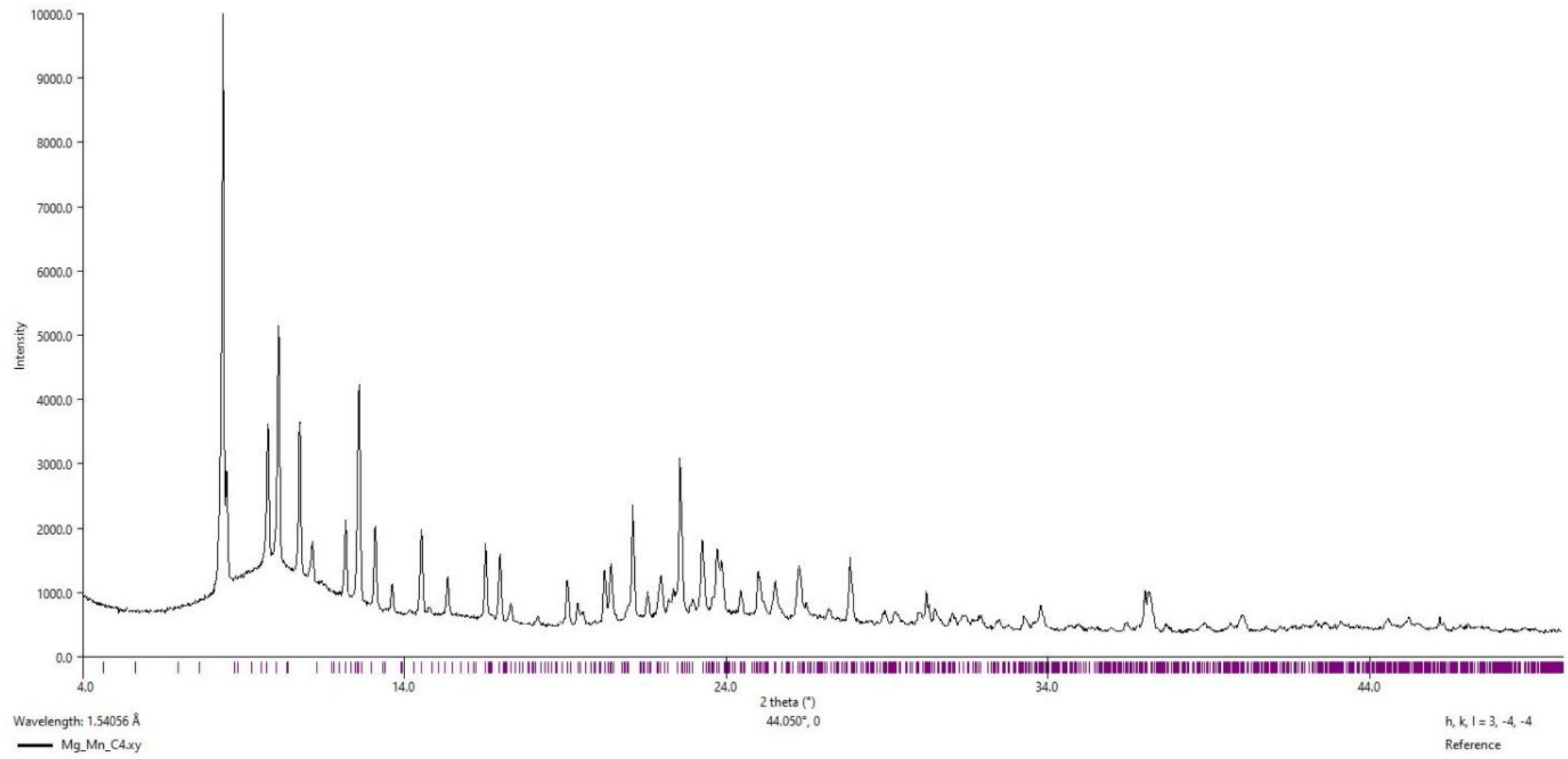

Figure S4. Powder X-ray diffraction (PXRD) pattern of complex C4.

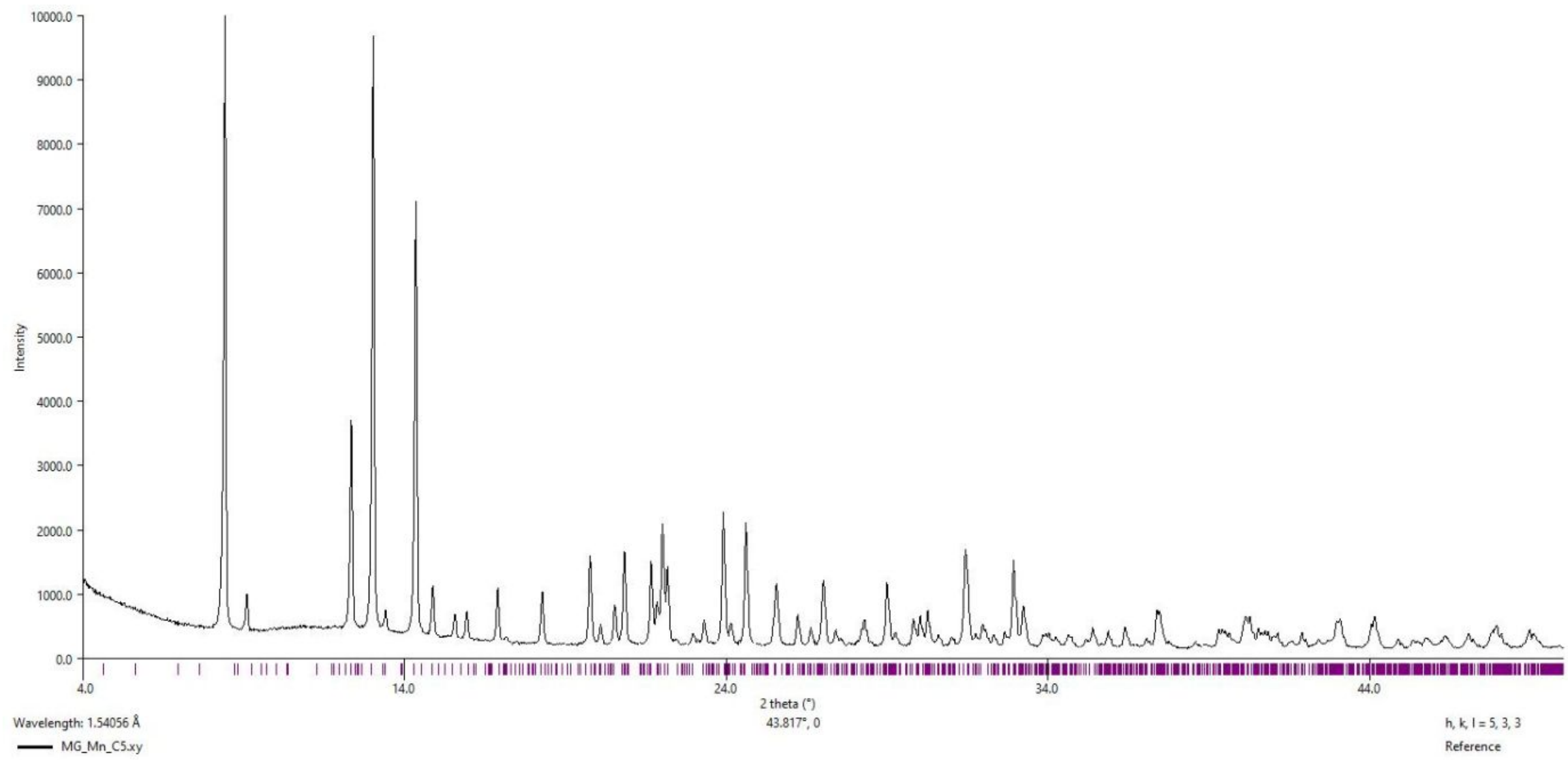

Figure S5. Powder X-ray diffraction (PXRD) pattern of complex C5.

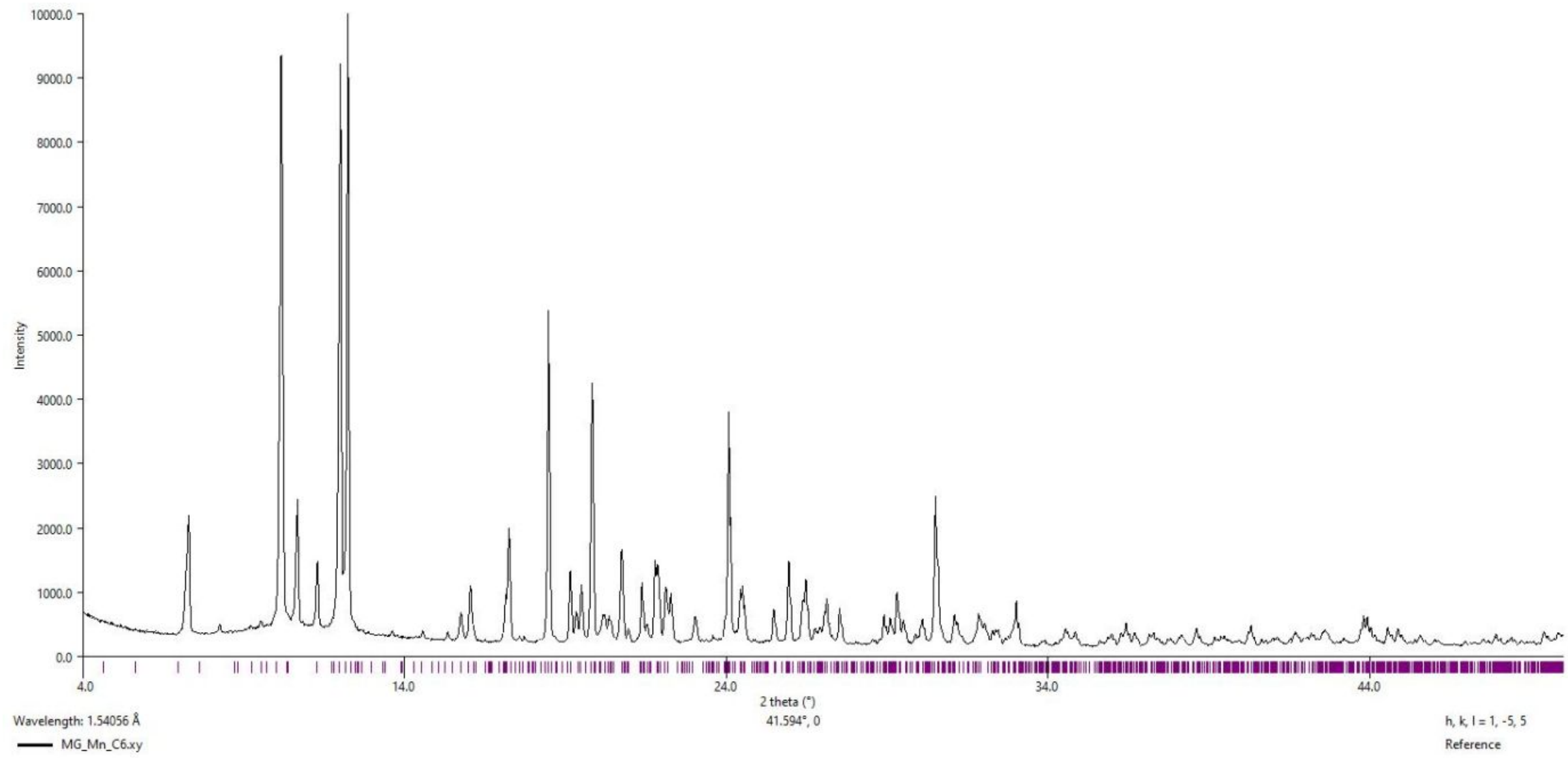

Figure S6. Powder X-ray diffraction (PXRD) pattern of complex C6.

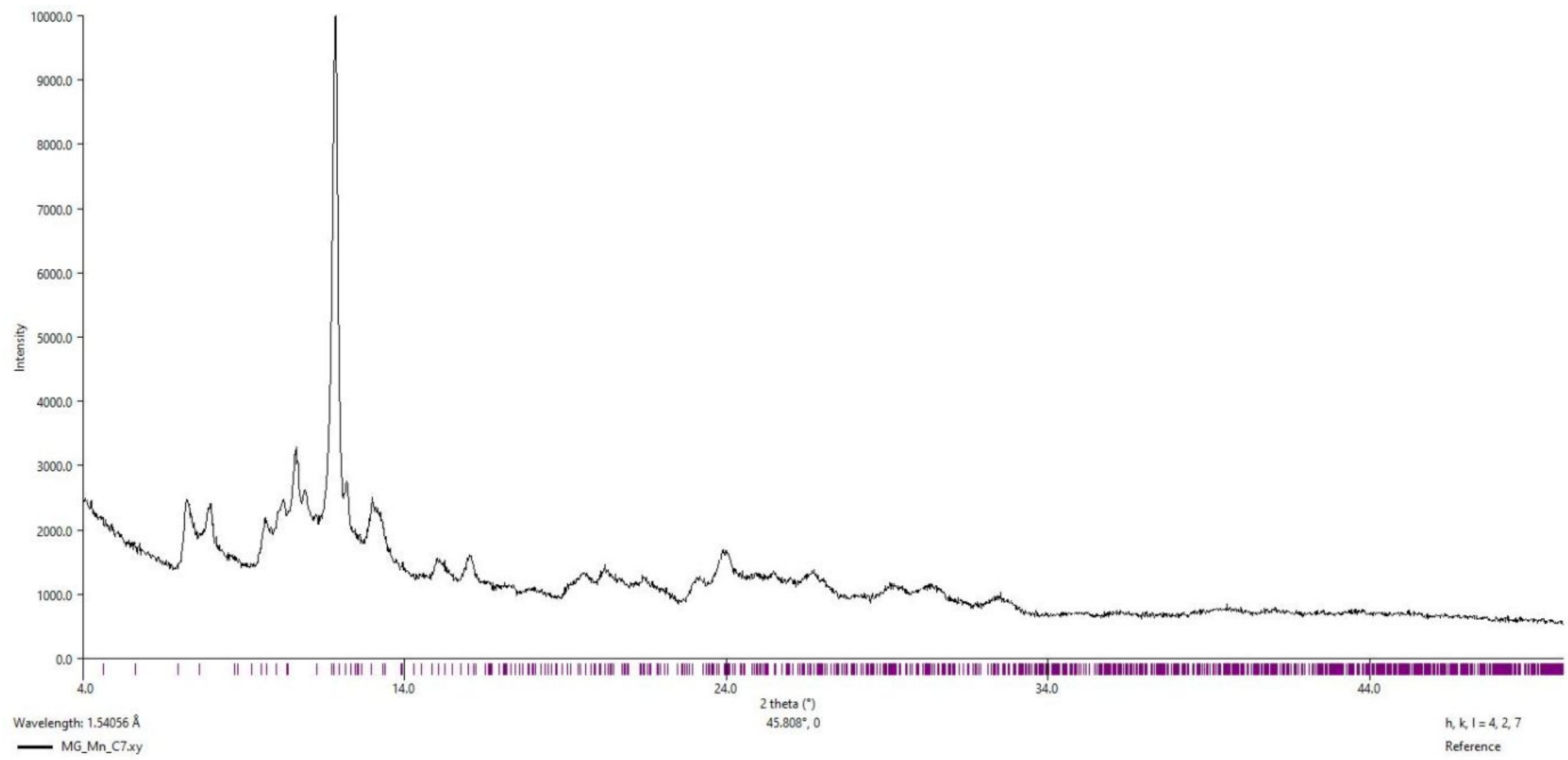

Figure S7. Powder X-ray diffraction (PXRD) pattern of complex C7.

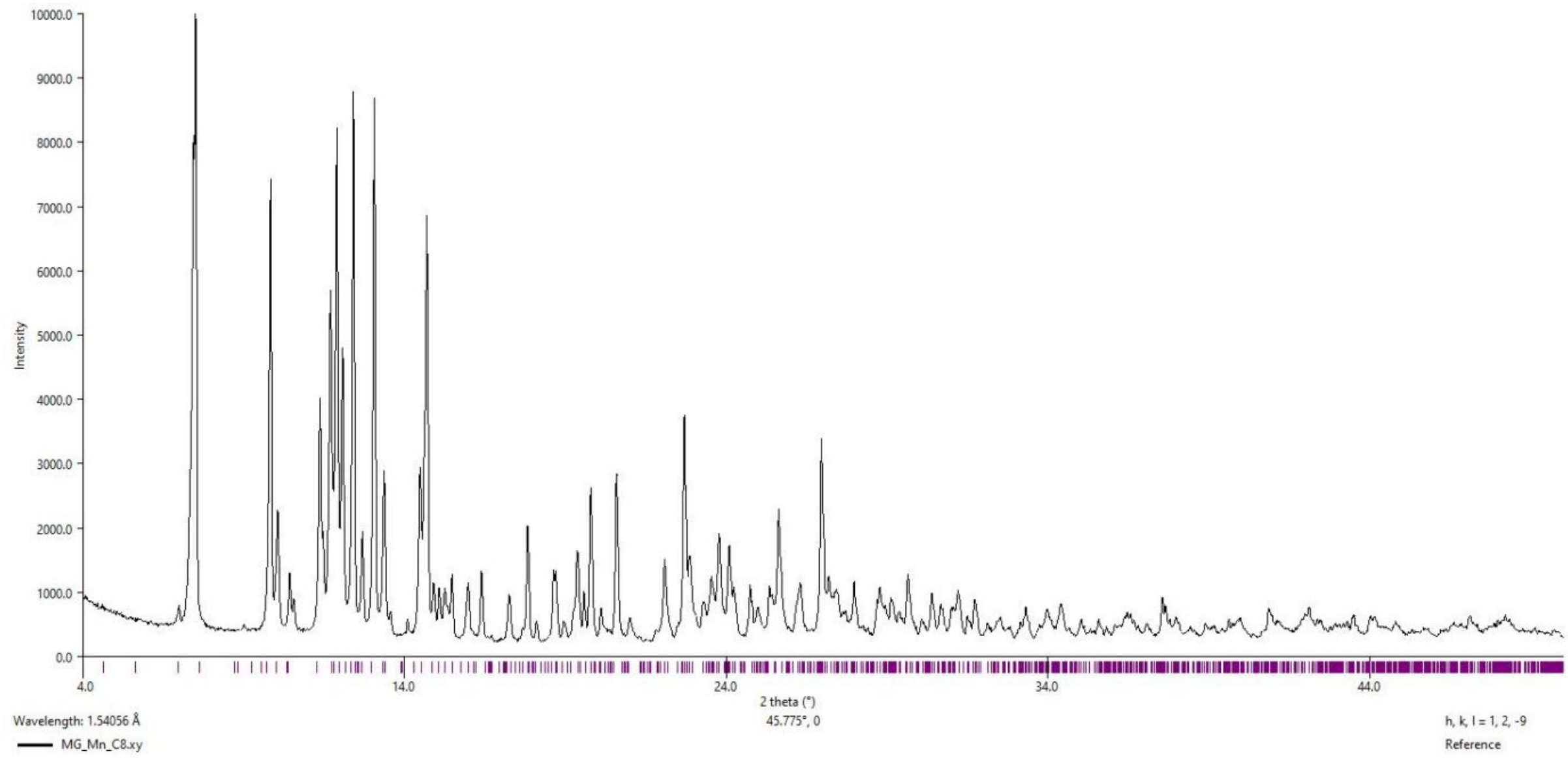

Figure S8. Powder X-ray diffraction (PXRD) pattern of complex C8.

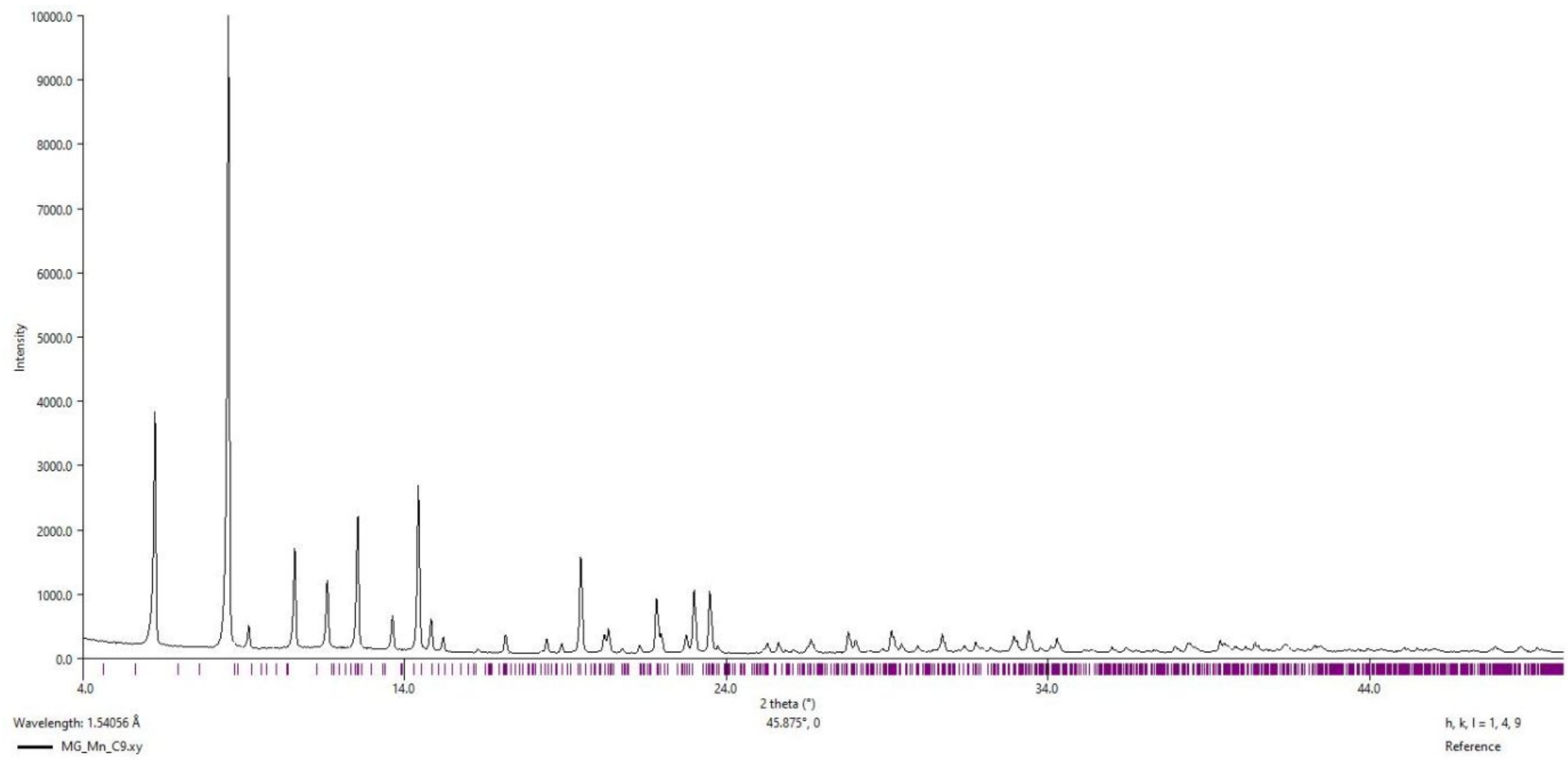

Figure S9. Powder X-ray diffraction (PXRD) pattern of complex C9.

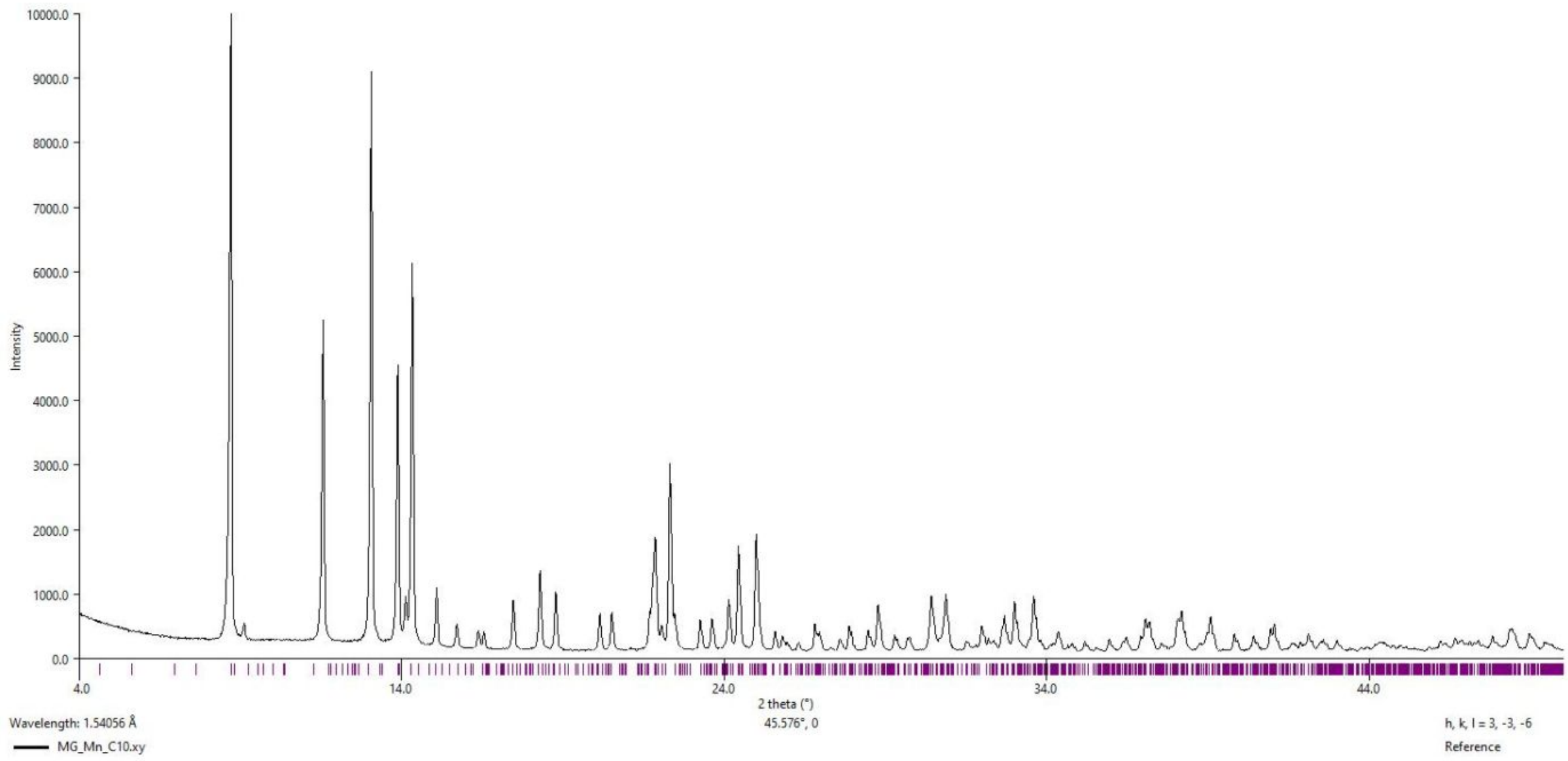

Figure S10. Powder X-ray diffraction (PXRD) pattern of complex C10.

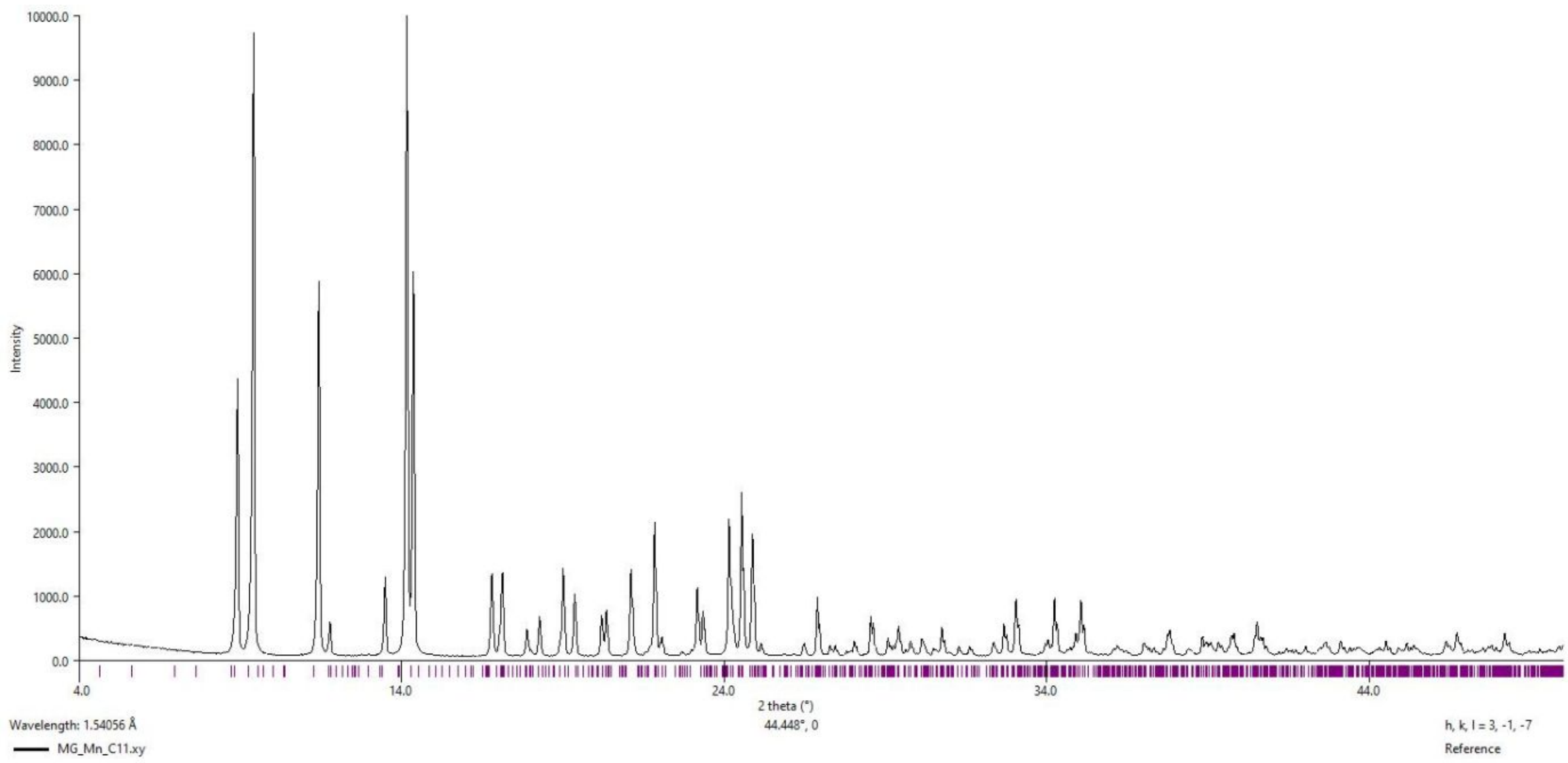

Figure S11. Powder X-ray diffraction (PXRD) pattern of complex C11.

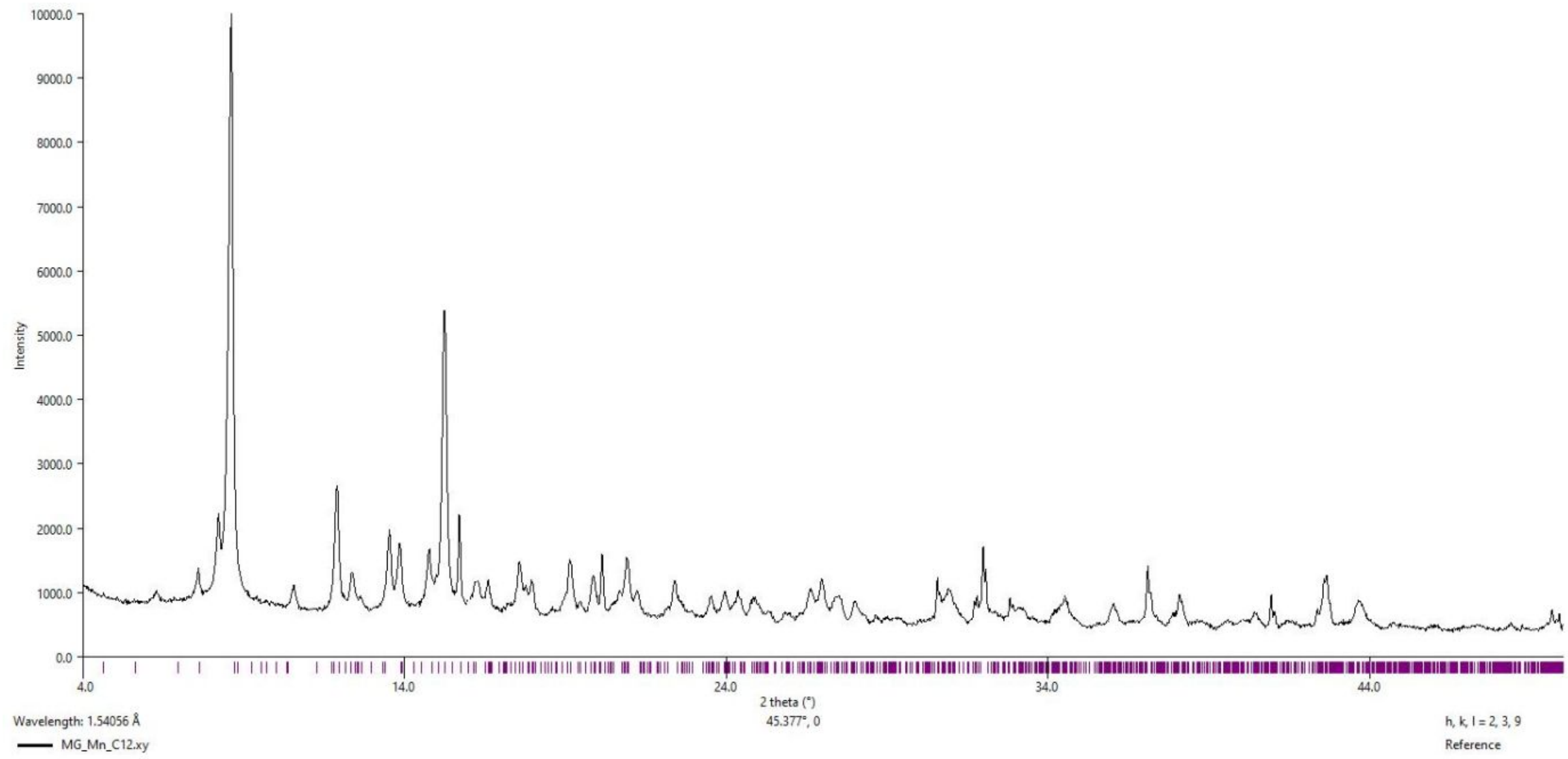

Figure S12. Powder X-ray diffraction (PXRD) pattern of complex C12.

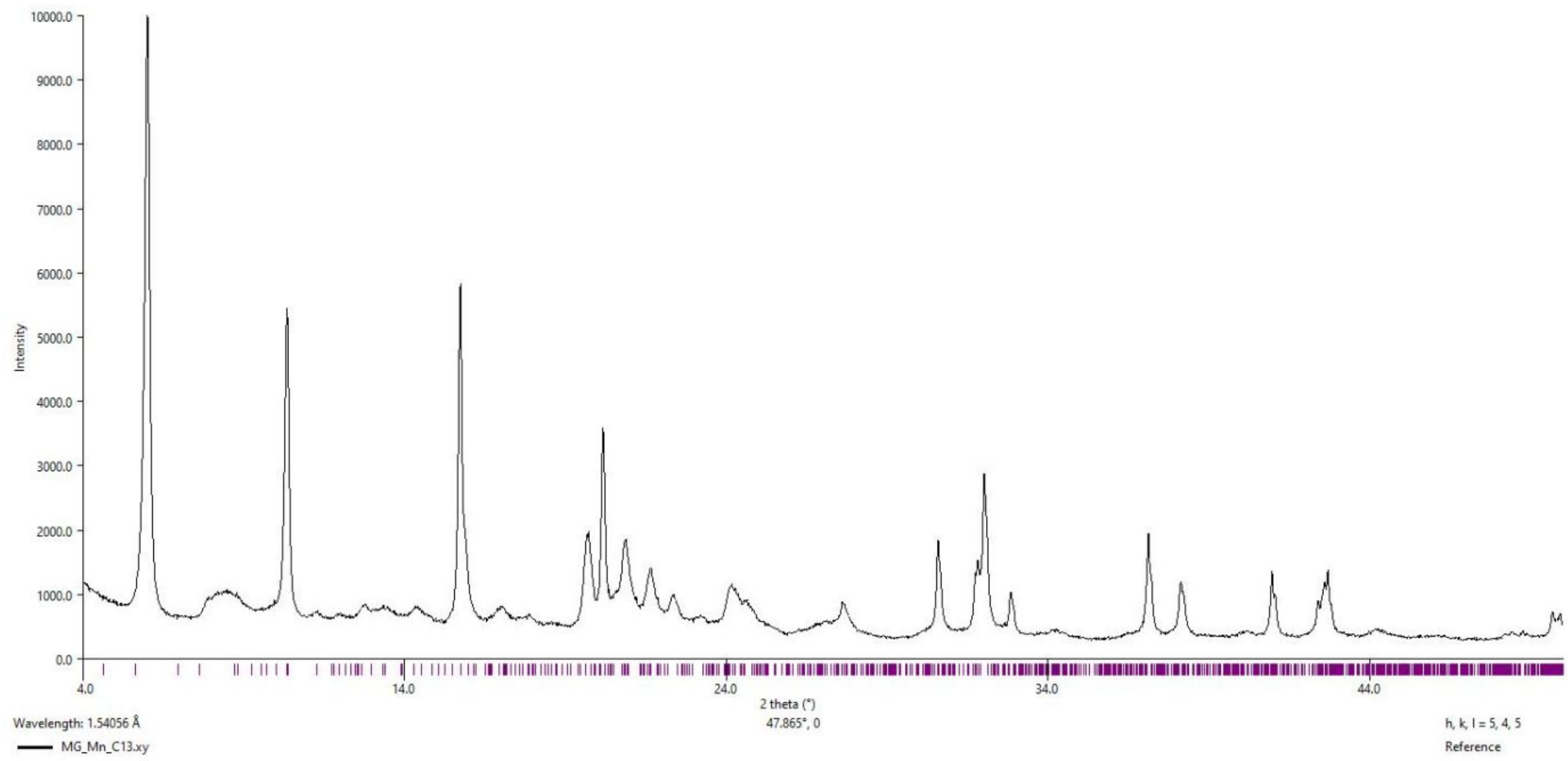

Figure S13. Powder X-ray diffraction (PXRD) pattern of complex C13.

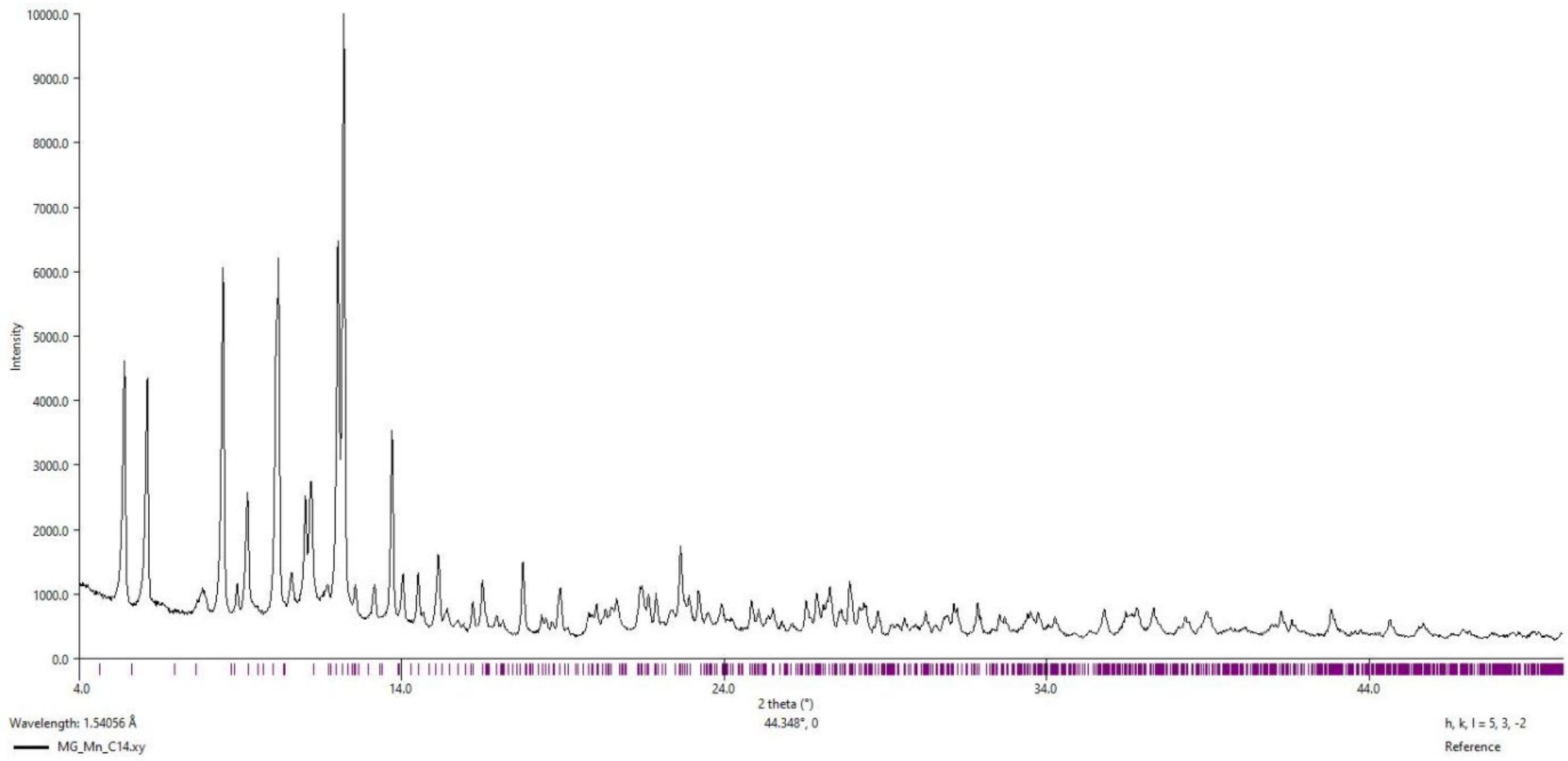

Figure S14. Powder X-ray diffraction (PXRD) pattern of complex C14.

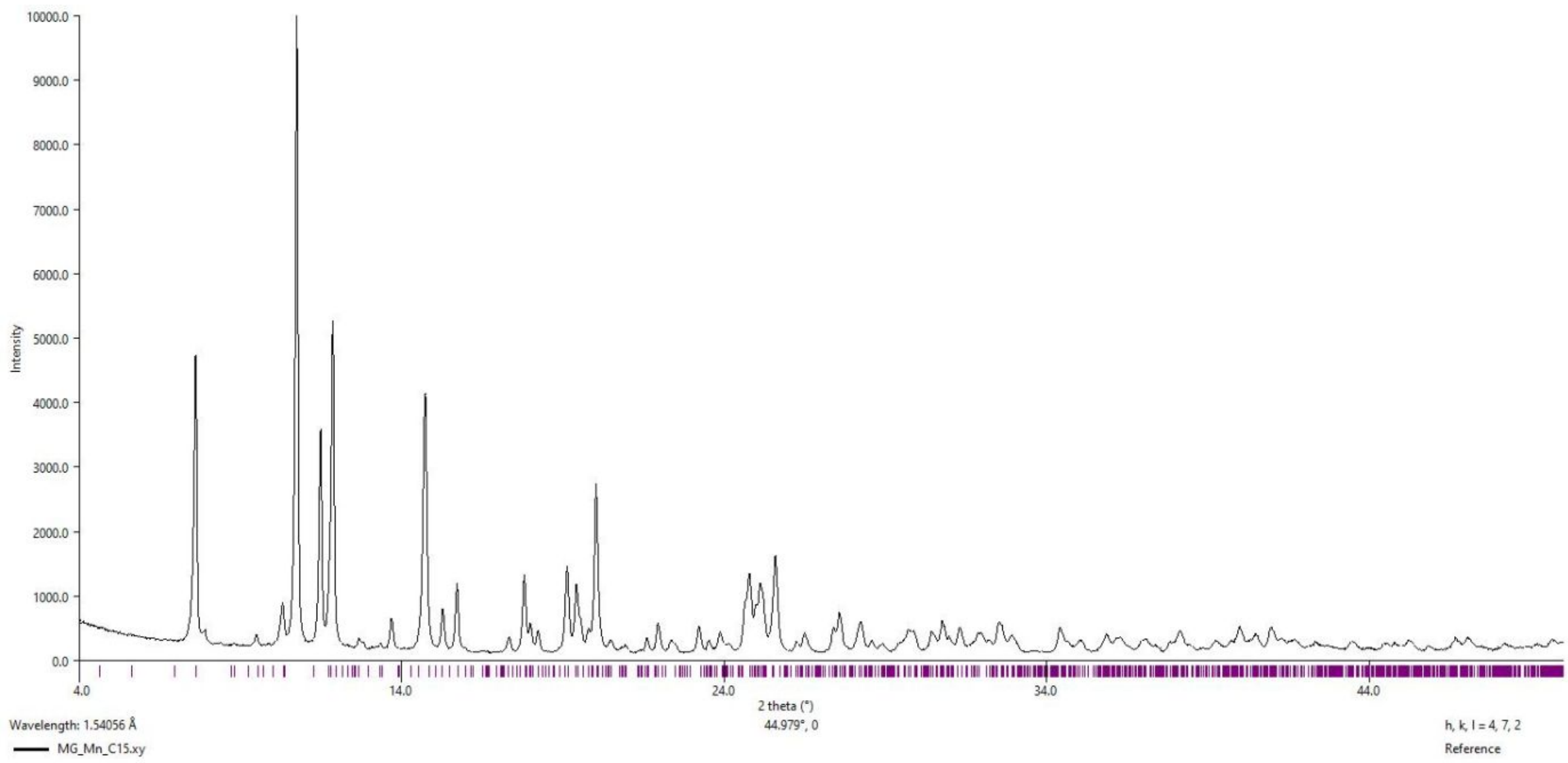

Figure S15. Powder X-ray diffraction (PXRD) pattern of complex C15.

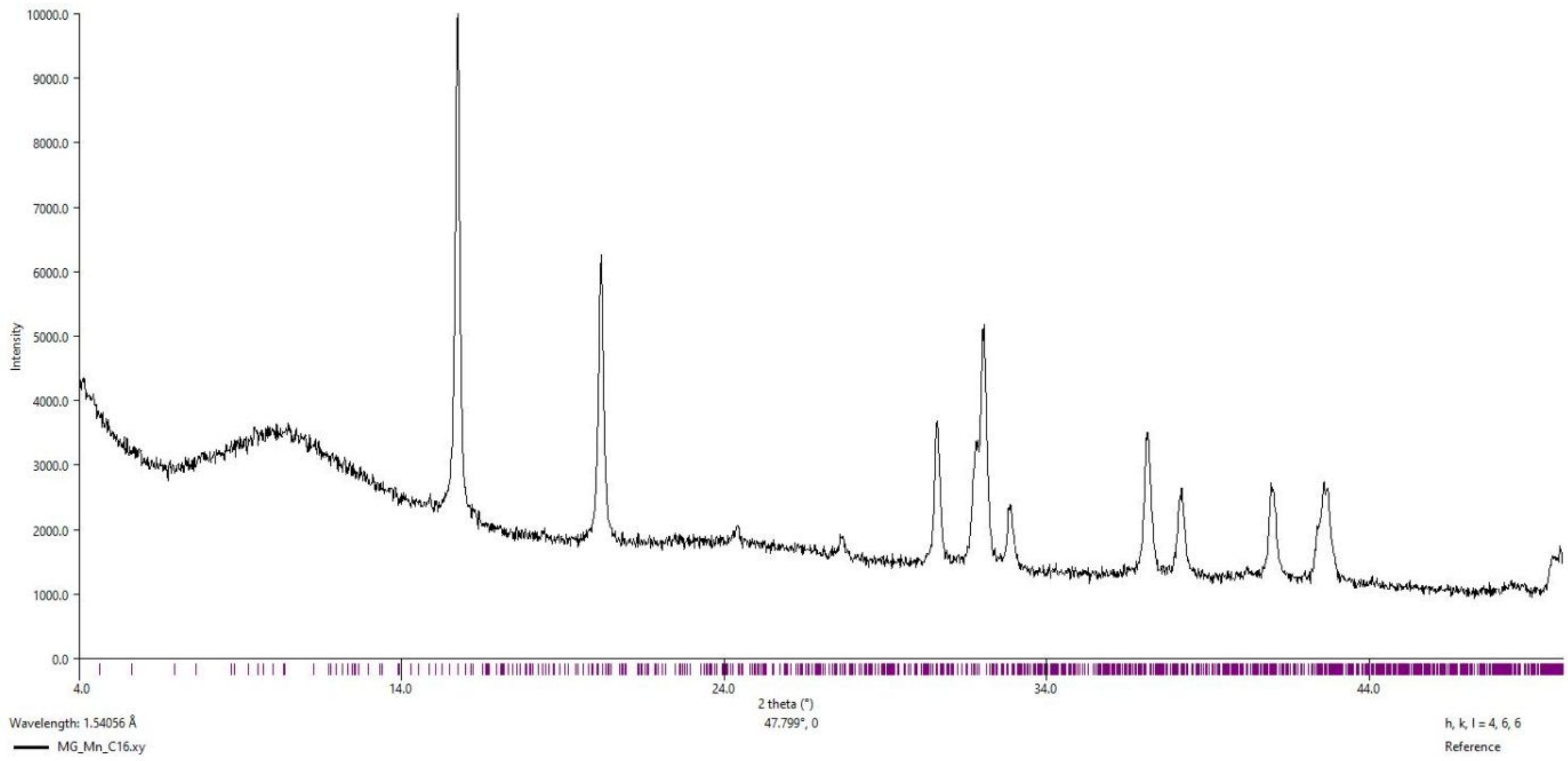

Figure S16. Powder X-ray diffraction (PXRD) pattern of complex C16.

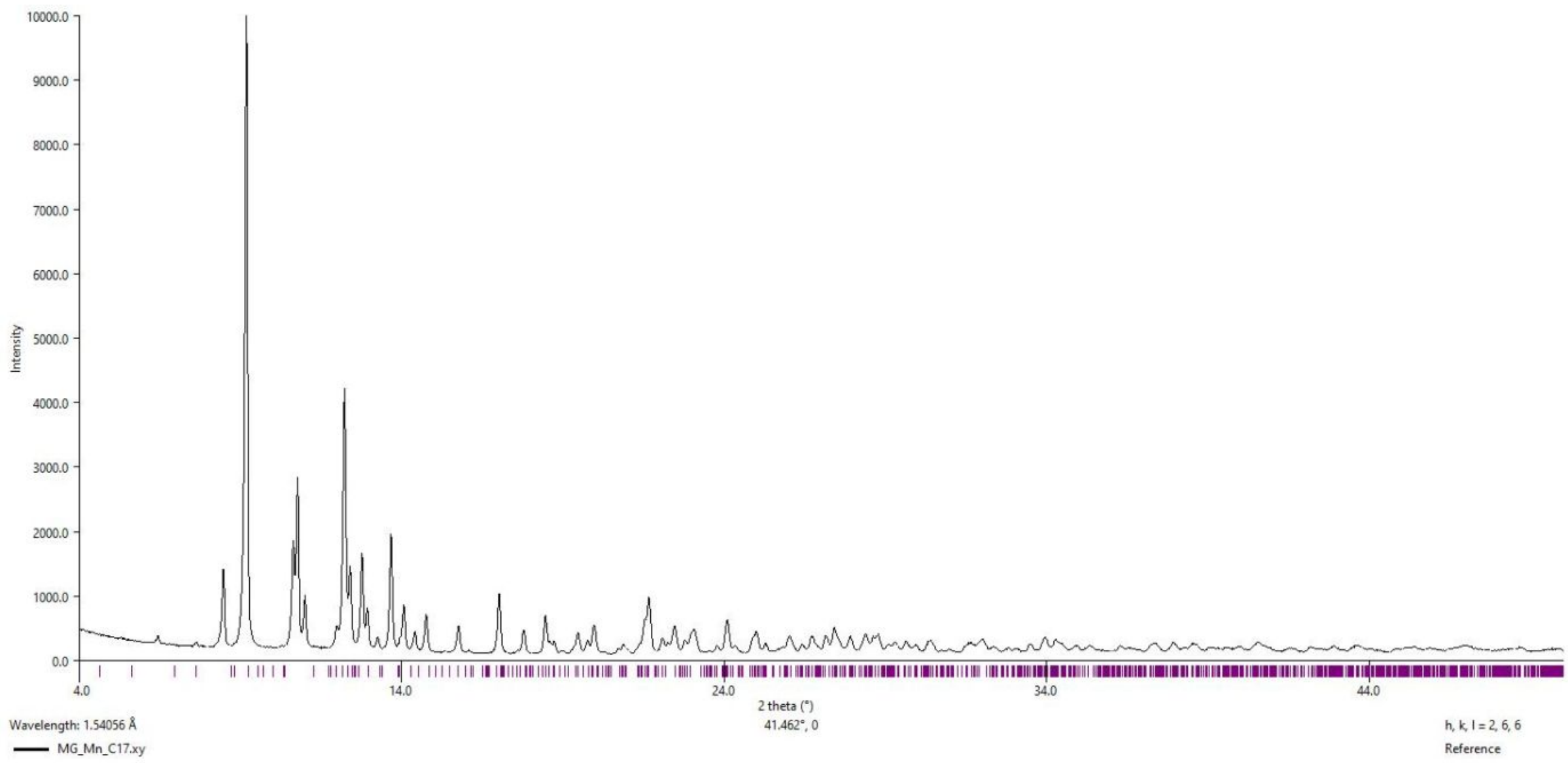

Figure S17. Powder X-ray diffraction (PXRD) pattern of complex C17.

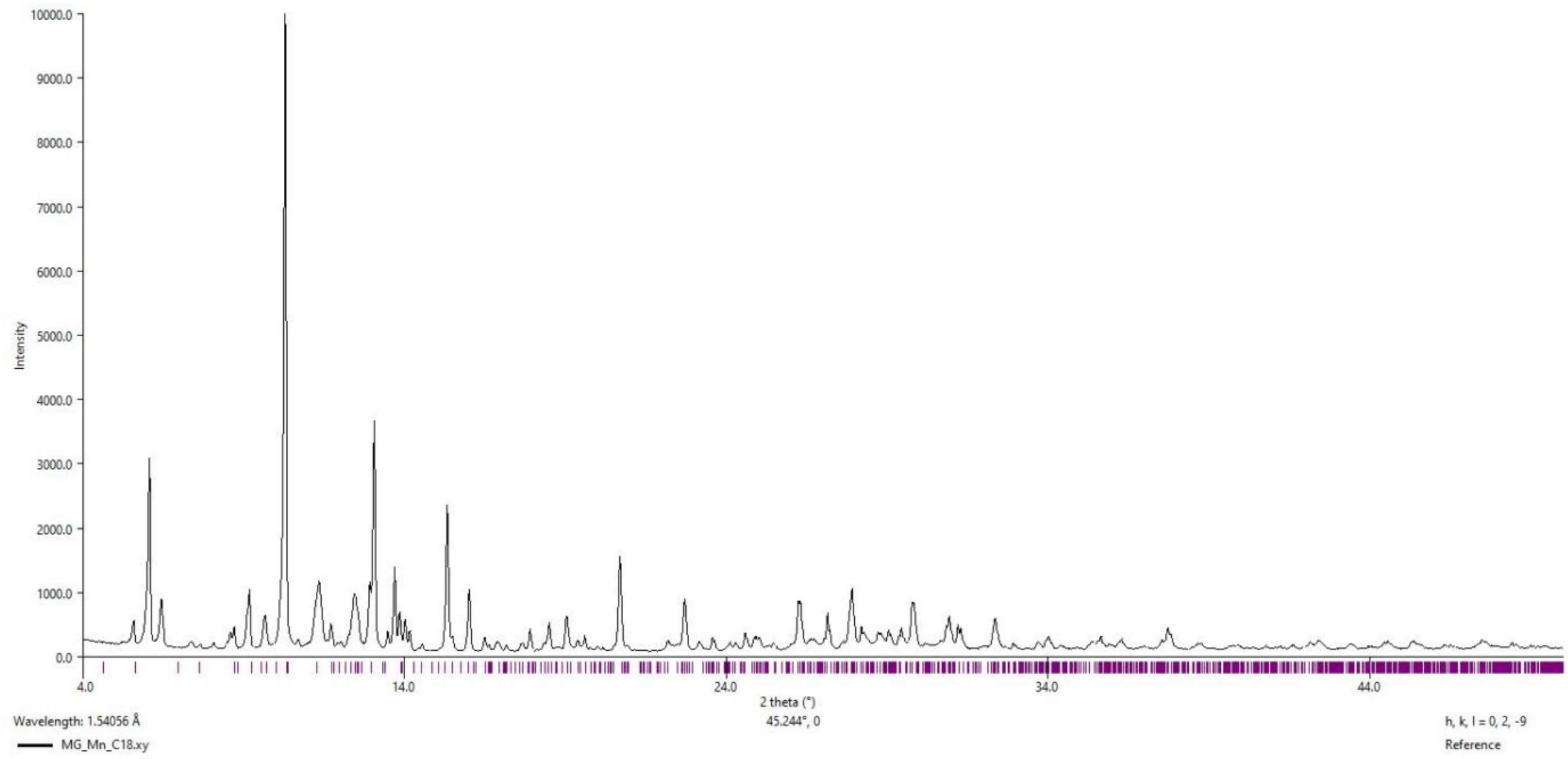

Figure S18. Powder X-ray diffraction (PXRD) pattern of complex C18.

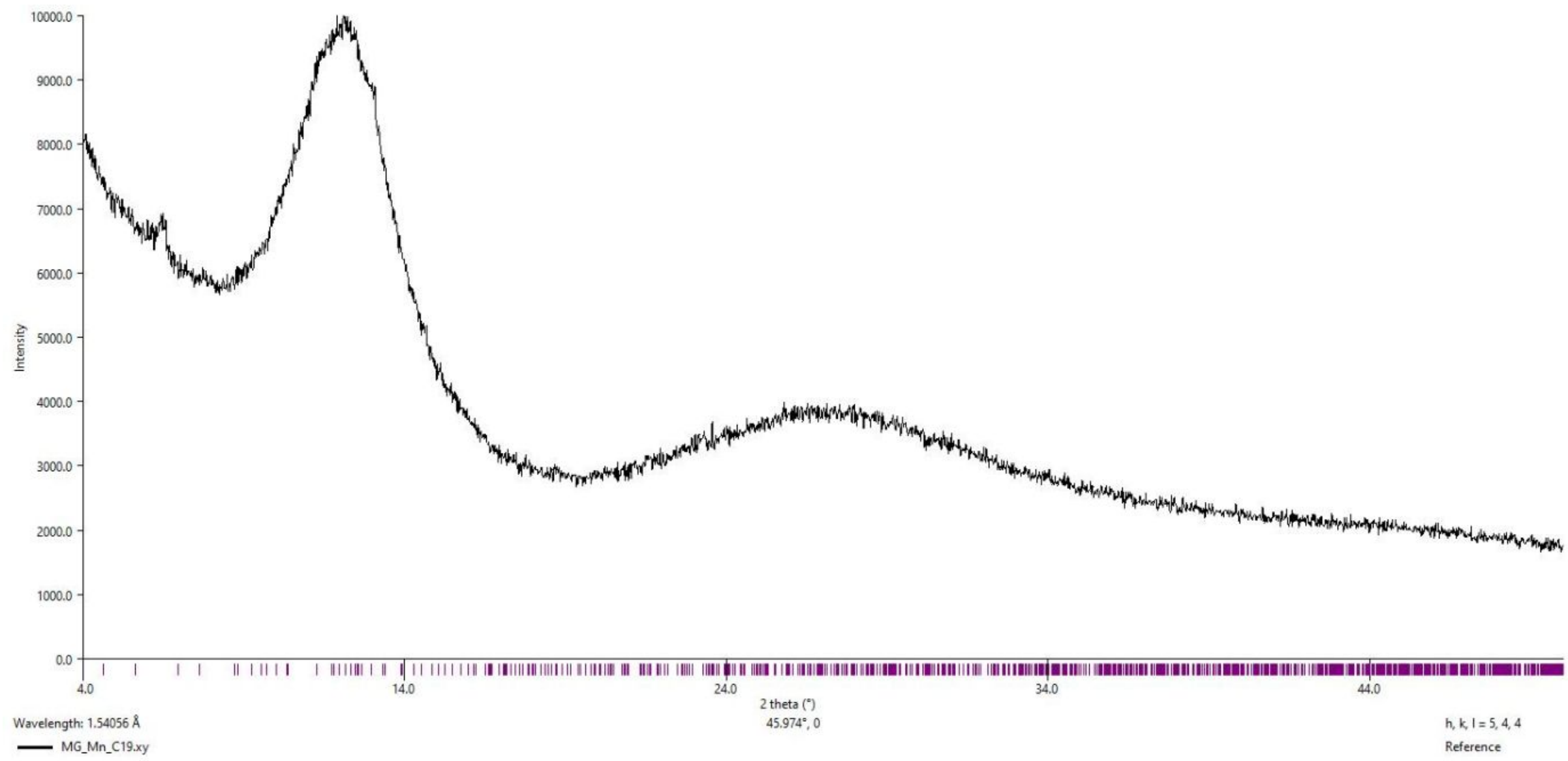

Figure S19. Powder X-ray diffraction (PXRD) pattern of complex C19.

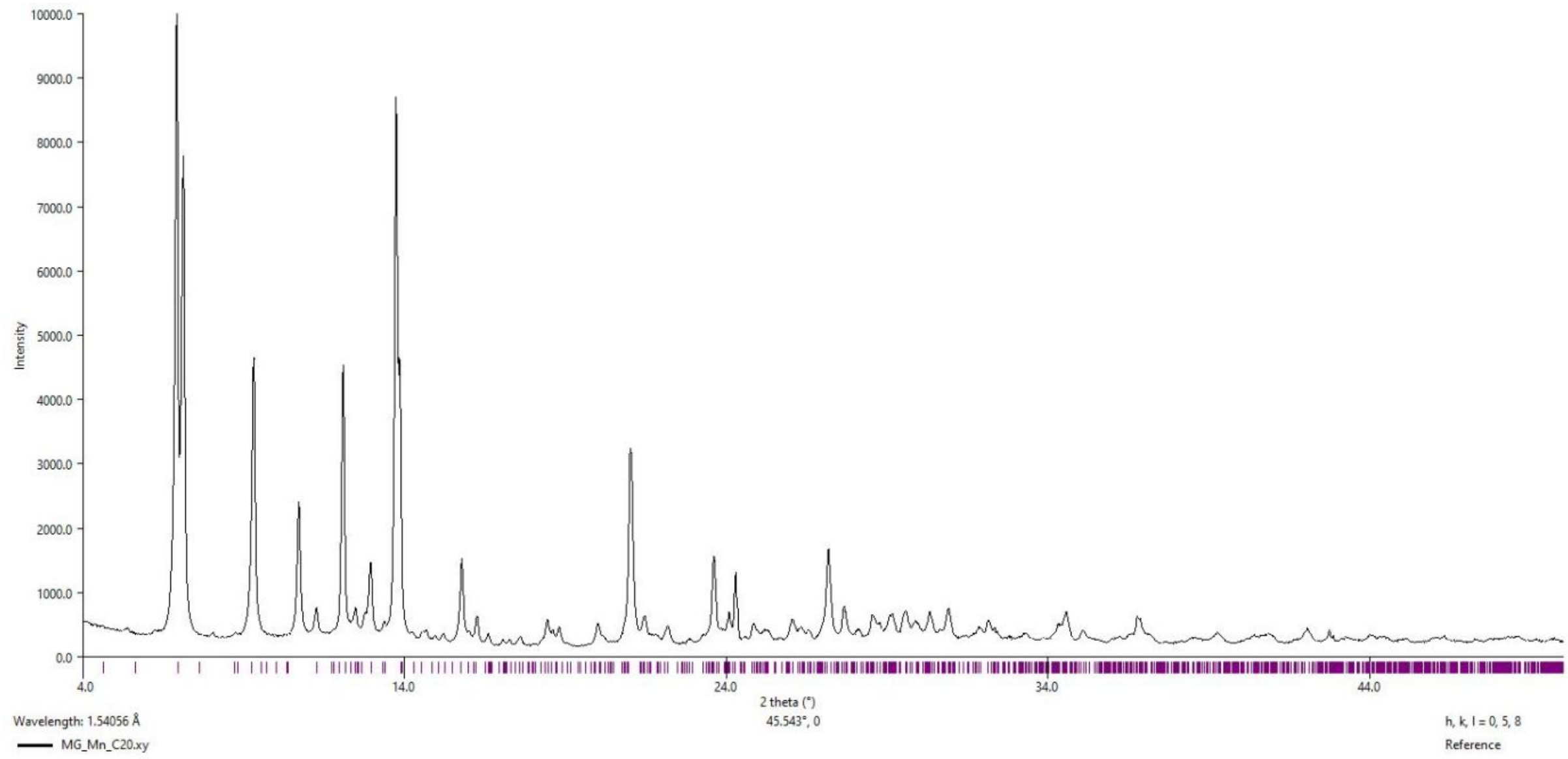

Figure S20. Powder X-ray diffraction (PXRD) pattern of complex C20.

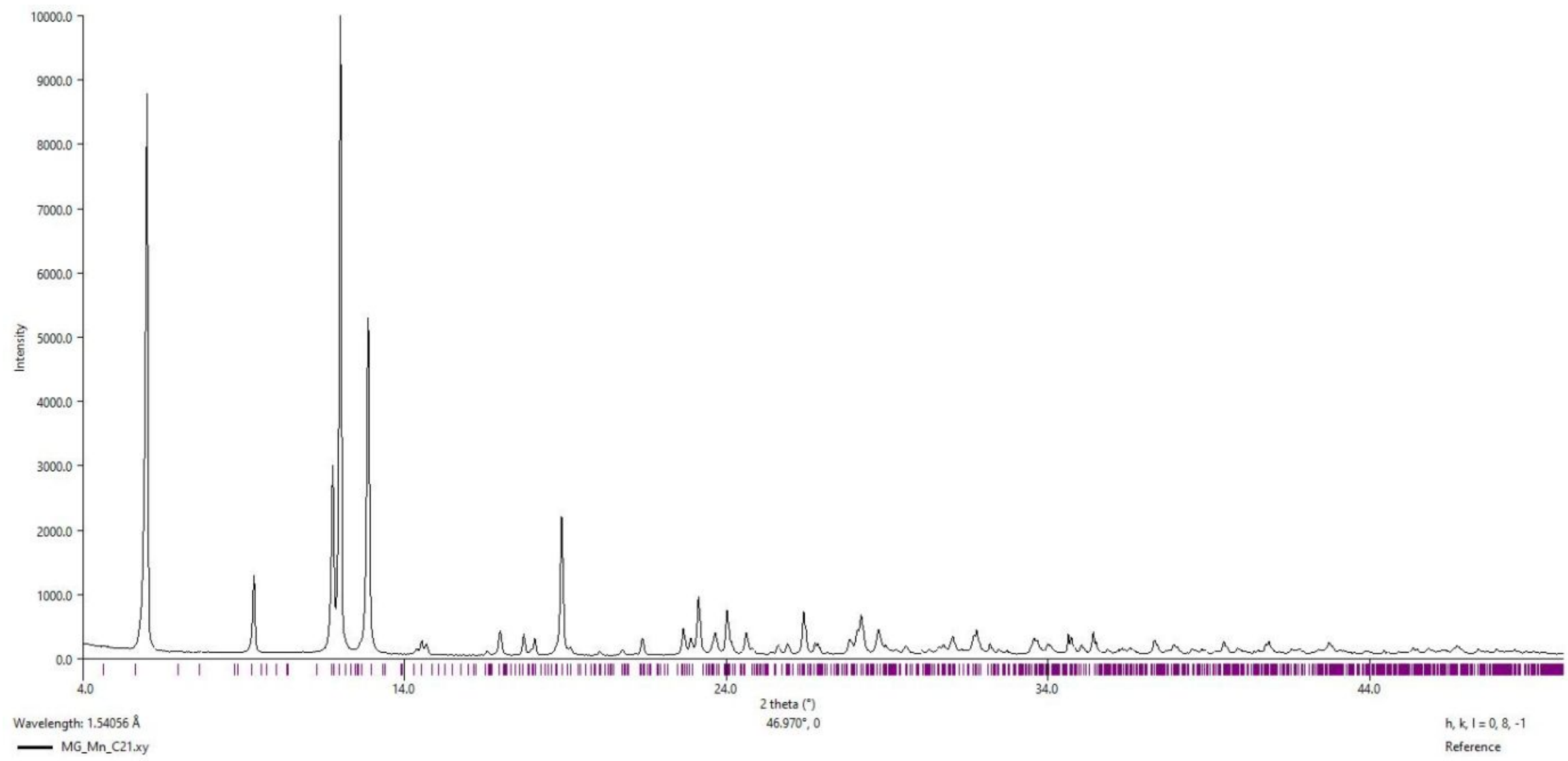

Figure S21. Powder X-ray diffraction (PXRD) pattern of complex C21.

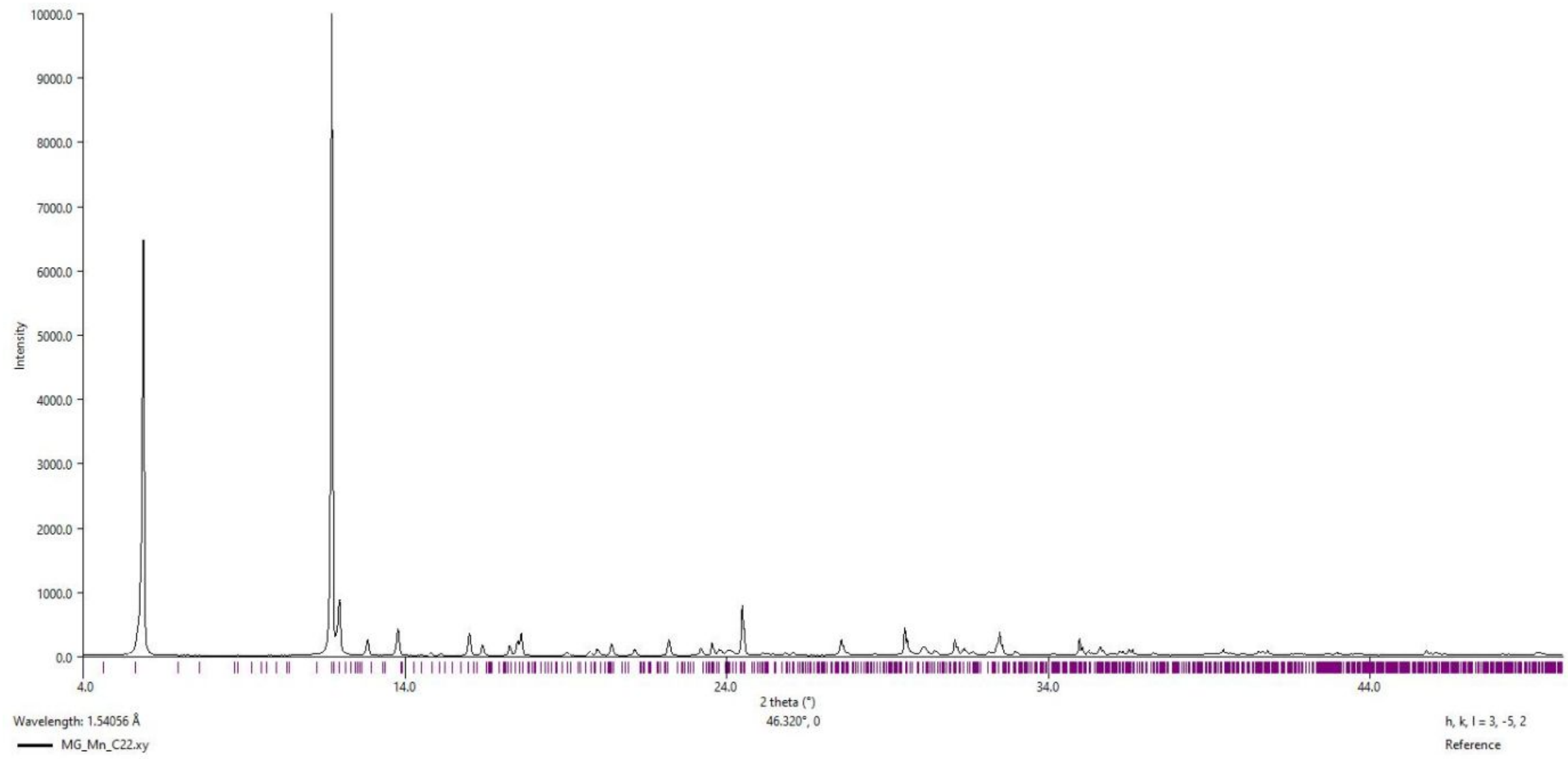

Figure S22. Powder X-ray diffraction (PXRD) pattern of complex C22.

The PXRD patterns and corresponding calculated patterns from the SCXRD structures are presented below to compare the bulk samples with the structurally characterized crystalline phases. Overall, the experimental patterns show reasonable agreement with the main reflections expected from the SCXRD structures, although the degree of similarity varies between the complexes. Differences in relative peak intensities, peak broadening, and additional weak reflections are observed in some cases. These deviations may be attributed to differences in crystallinity and preferred orientation, as well as the sensitivity of the complexes to moisture and oxygen. Despite storage under argon and minimizing air exposure, some samples became increasingly sticky during handling, suggesting partial moisture uptake and/or limited decomposition. Overall, the PXRD data provide support for some of the proposed structural assignments, while the variation in similarity means that they should not be considered conclusive evidence of phase purity.

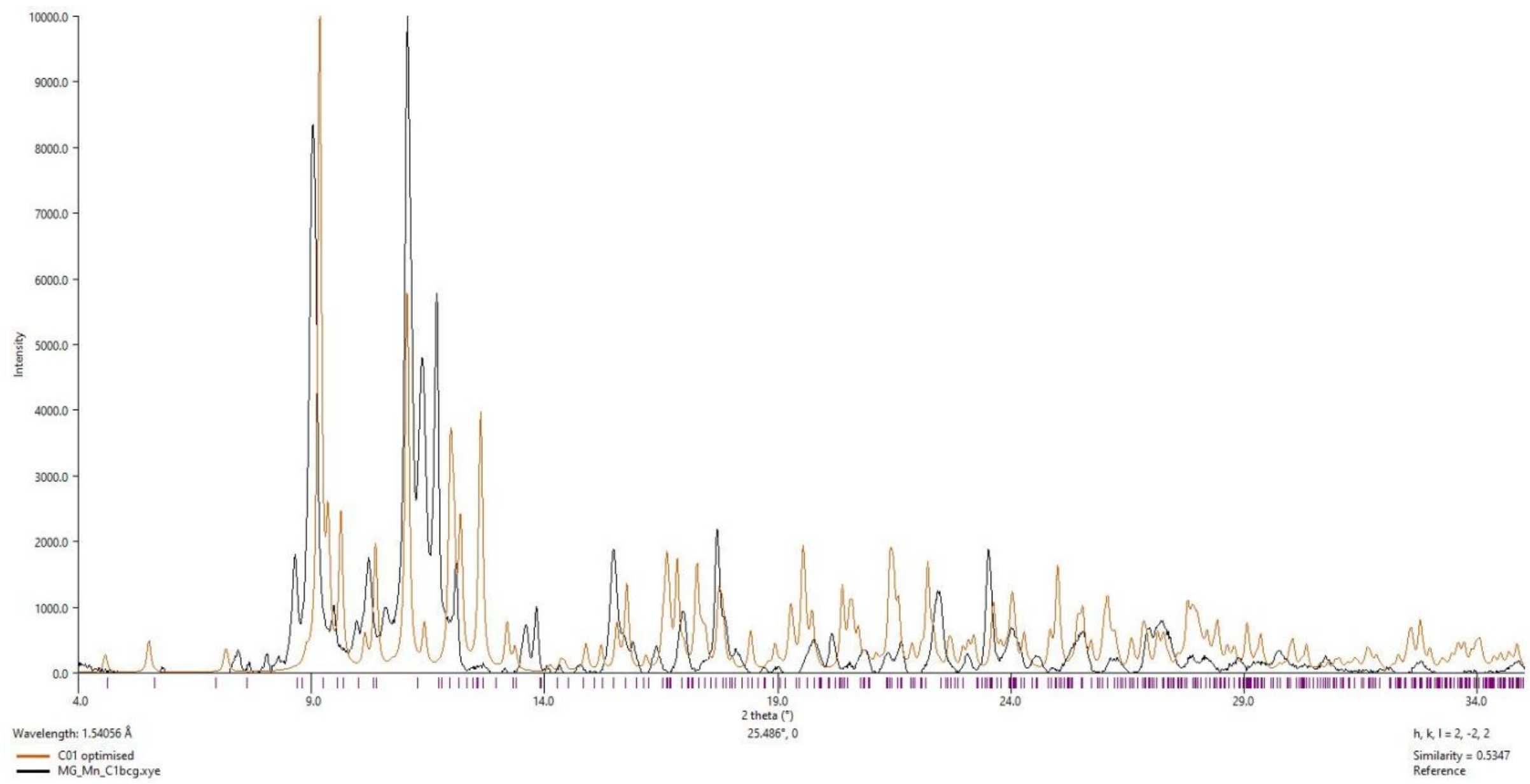

Figure S23. Comparison of the powder X-ray diffraction (PXRD) patterns of complex C1 and the corresponding calculated pattern derived from the SCXRD structure.

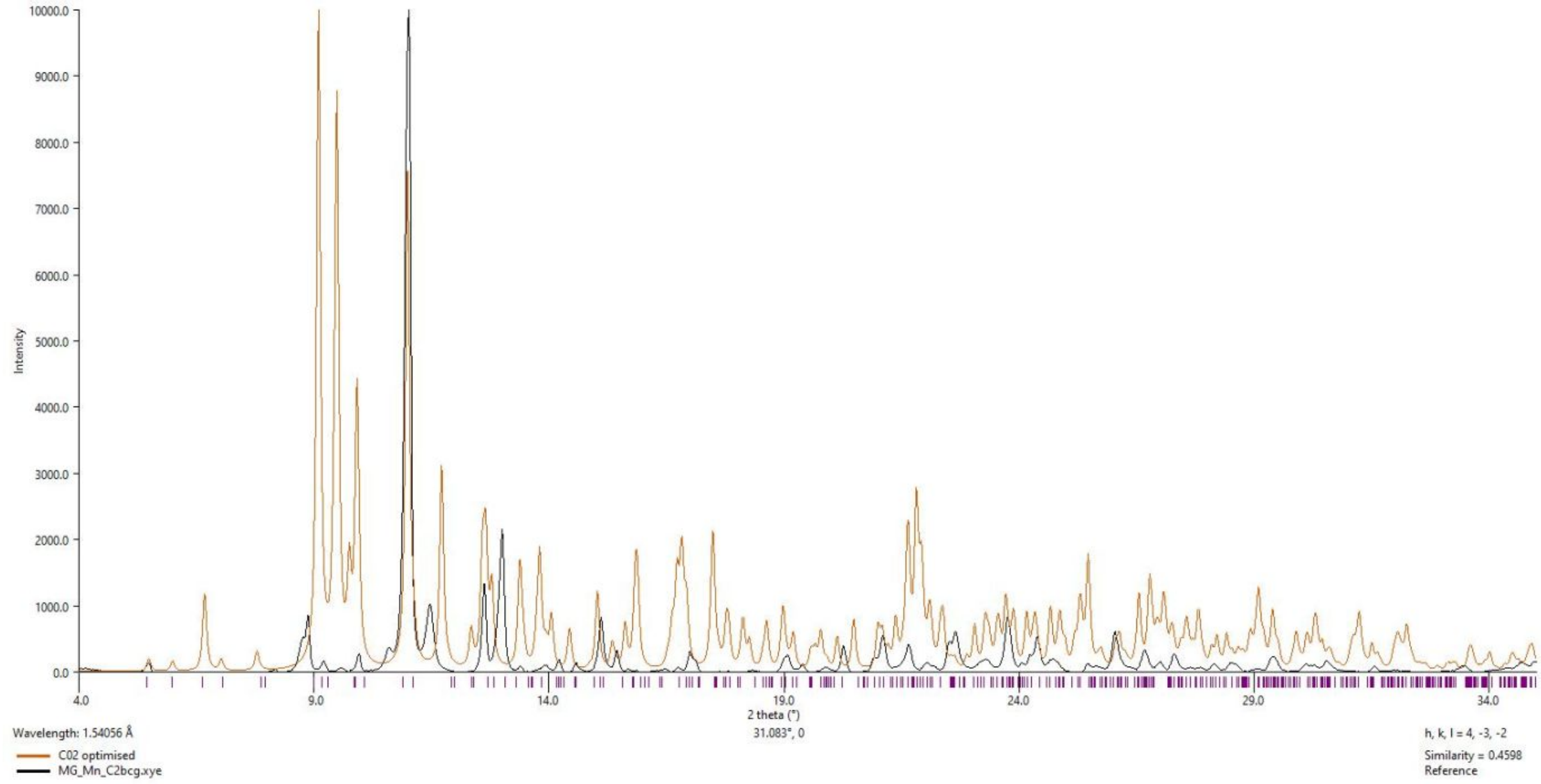

Figure S24. Comparison of the powder X-ray diffraction (PXRD) patterns of complex C2 and the corresponding calculated pattern derived from the SCXRD structure.

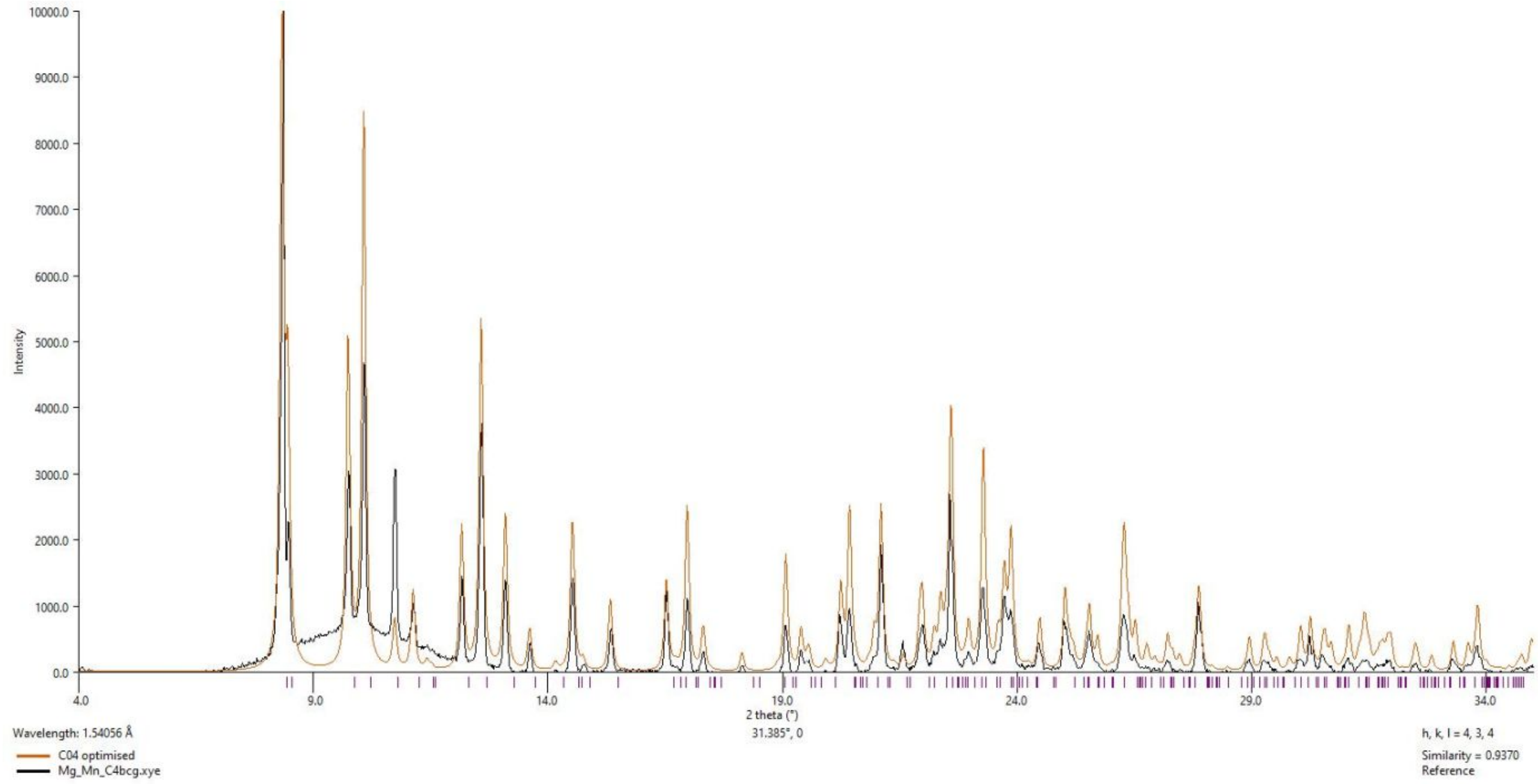

Figure S25. Comparison of the powder X-ray diffraction (PXRD) patterns of complex C4 and the corresponding calculated pattern derived from the SCXRD structure.

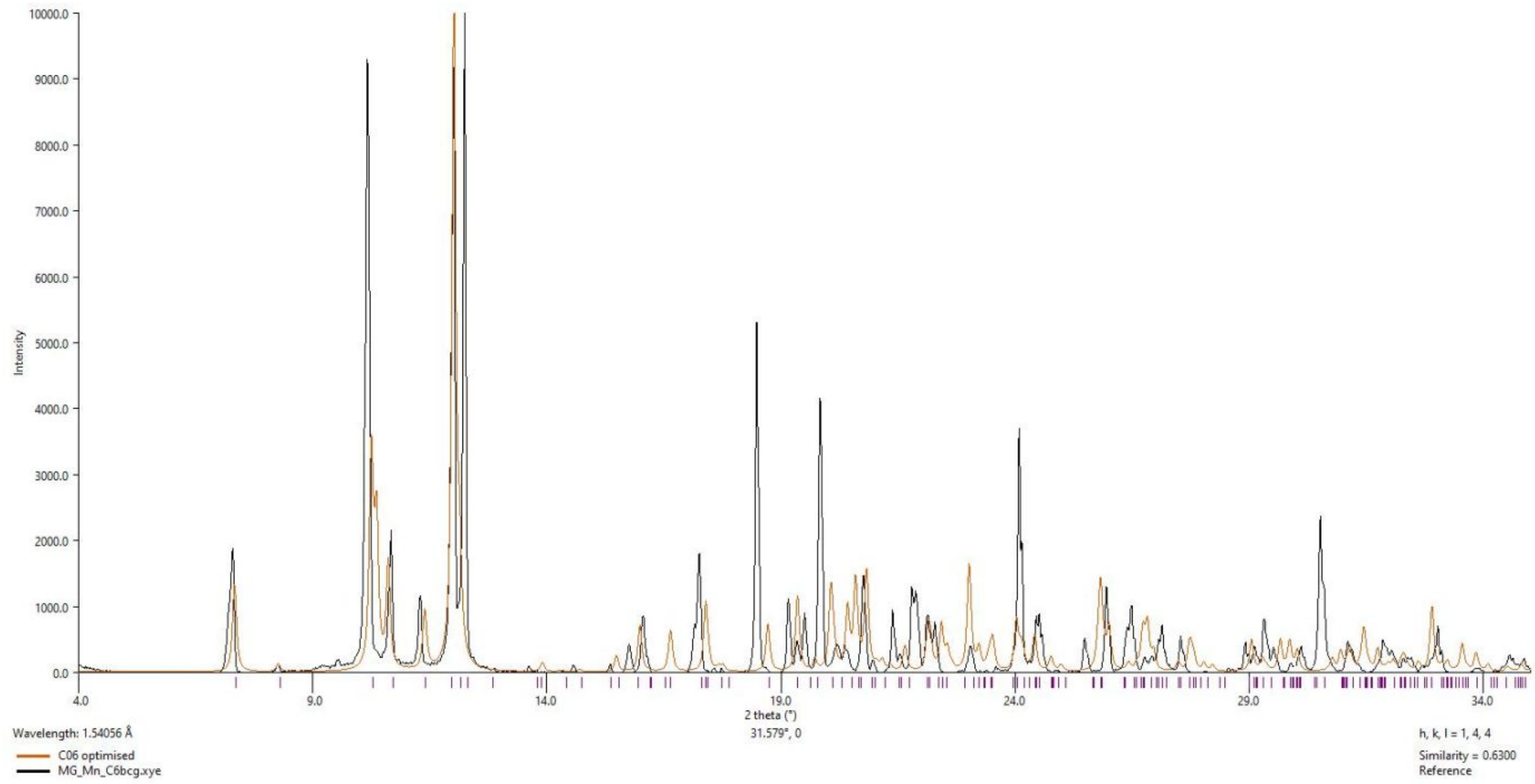

Figure S26. Comparison of the powder X-ray diffraction (PXRD) patterns of complex C6 and the corresponding calculated pattern derived from the SCXRD structure.

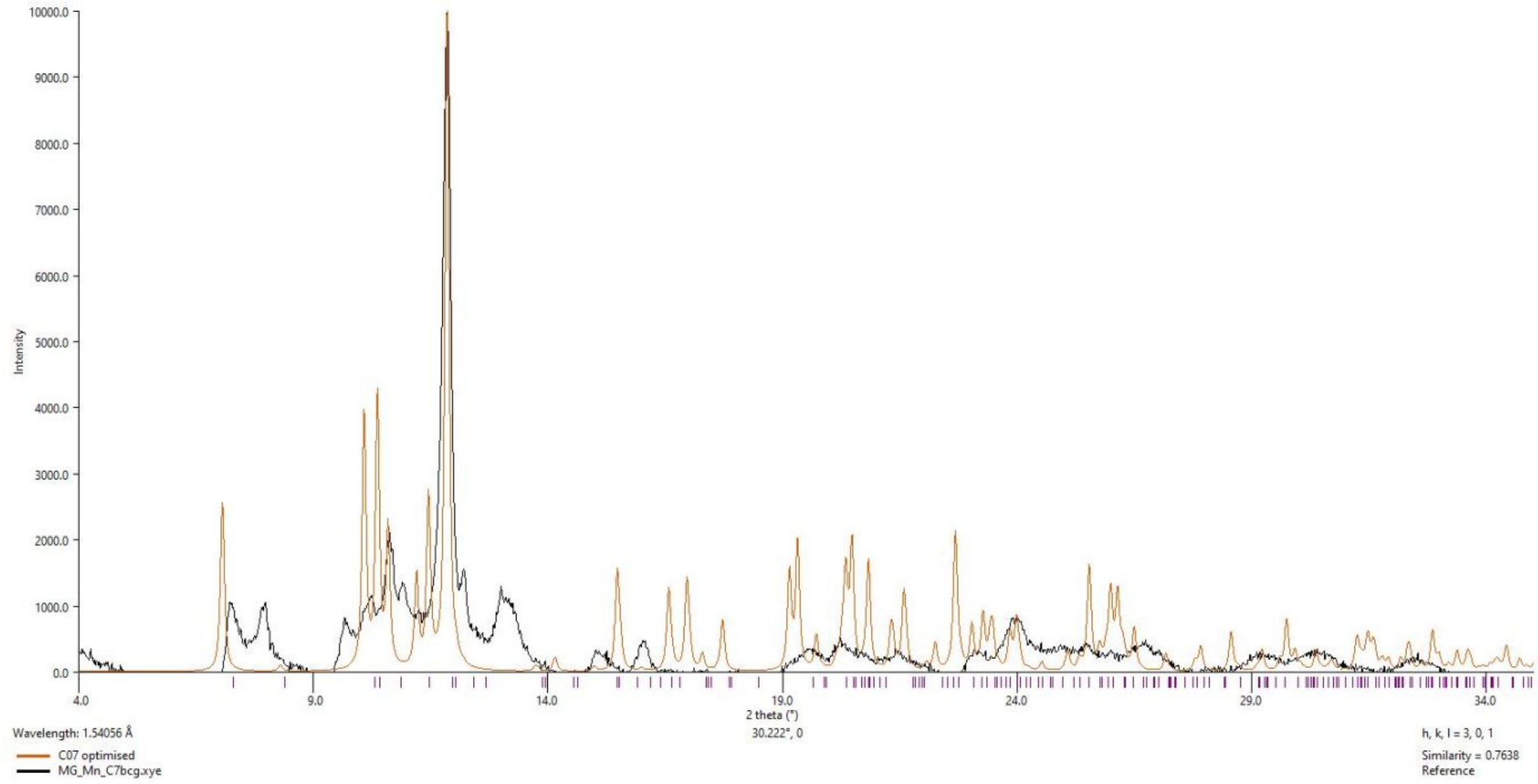

Figure S27. Comparison of the powder X-ray diffraction (PXRD) patterns of complex C7 and the corresponding calculated pattern derived from the SCXRD structure.

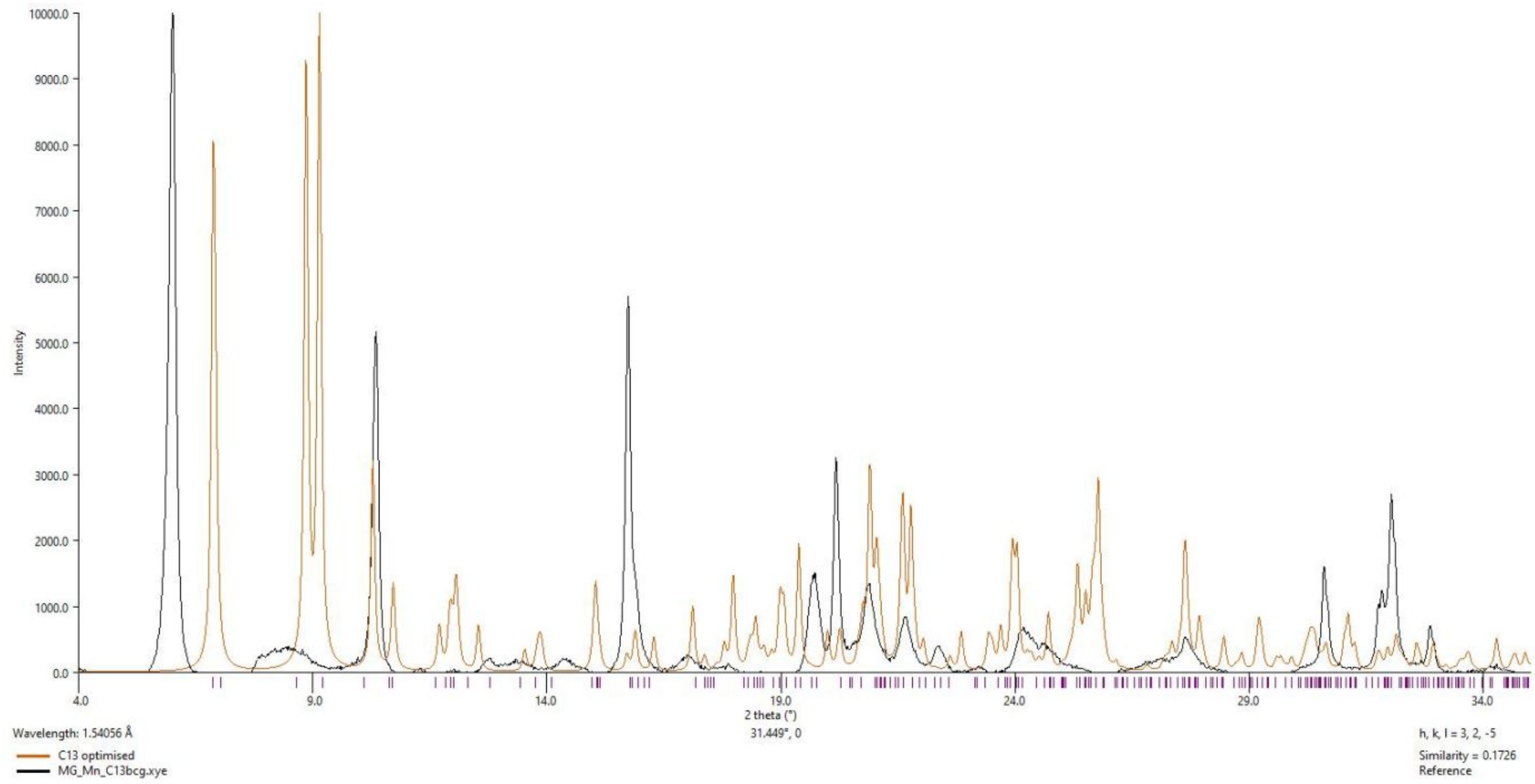

Figure S28. Comparison of the powder X-ray diffraction (PXRD) patterns of complex C13 and the corresponding calculated pattern derived from the SCXRD structure.

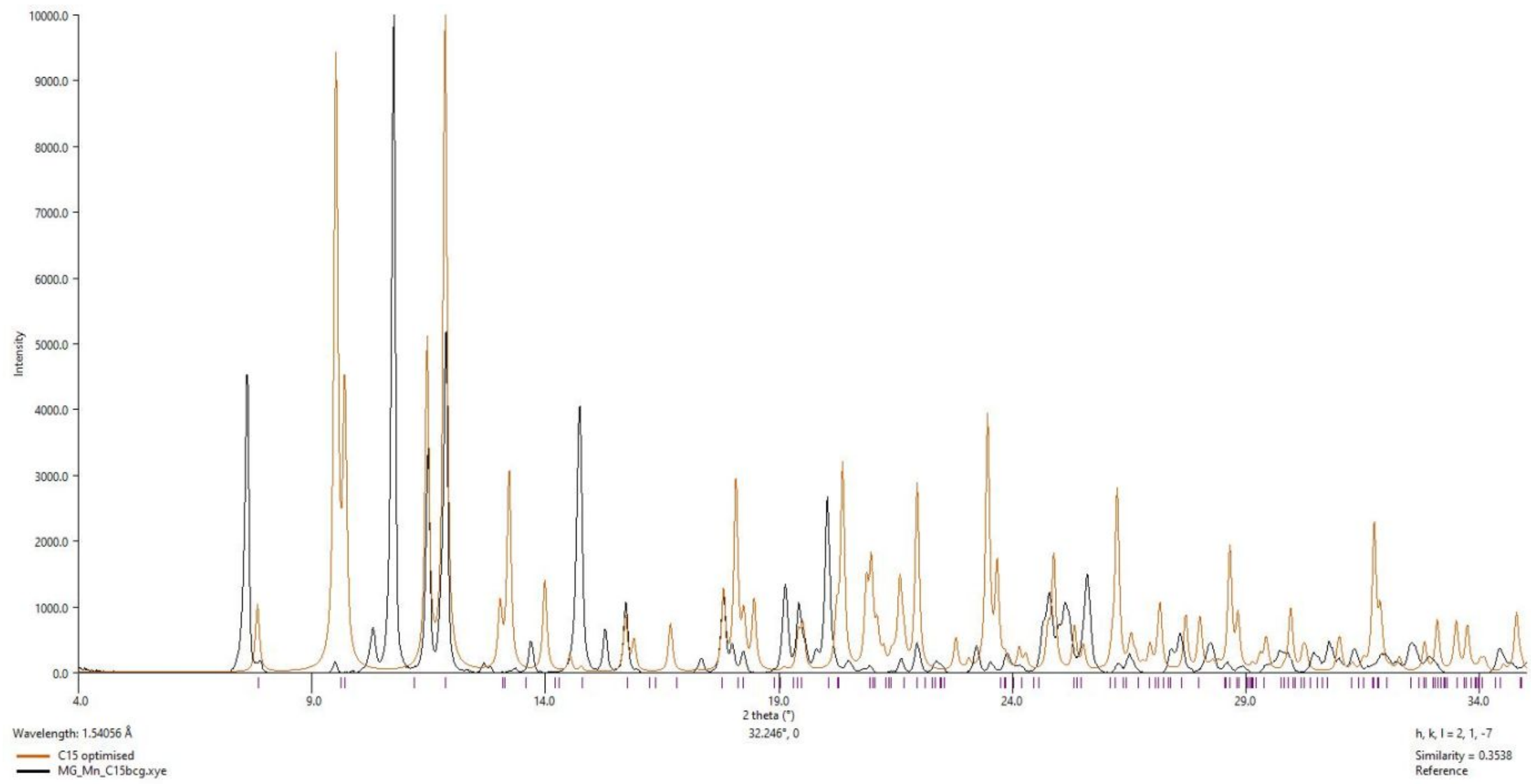

Figure S29. Comparison of the powder X-ray diffraction (PXRD) patterns of complex C15 and the corresponding calculated pattern derived from the SCXRD structure.

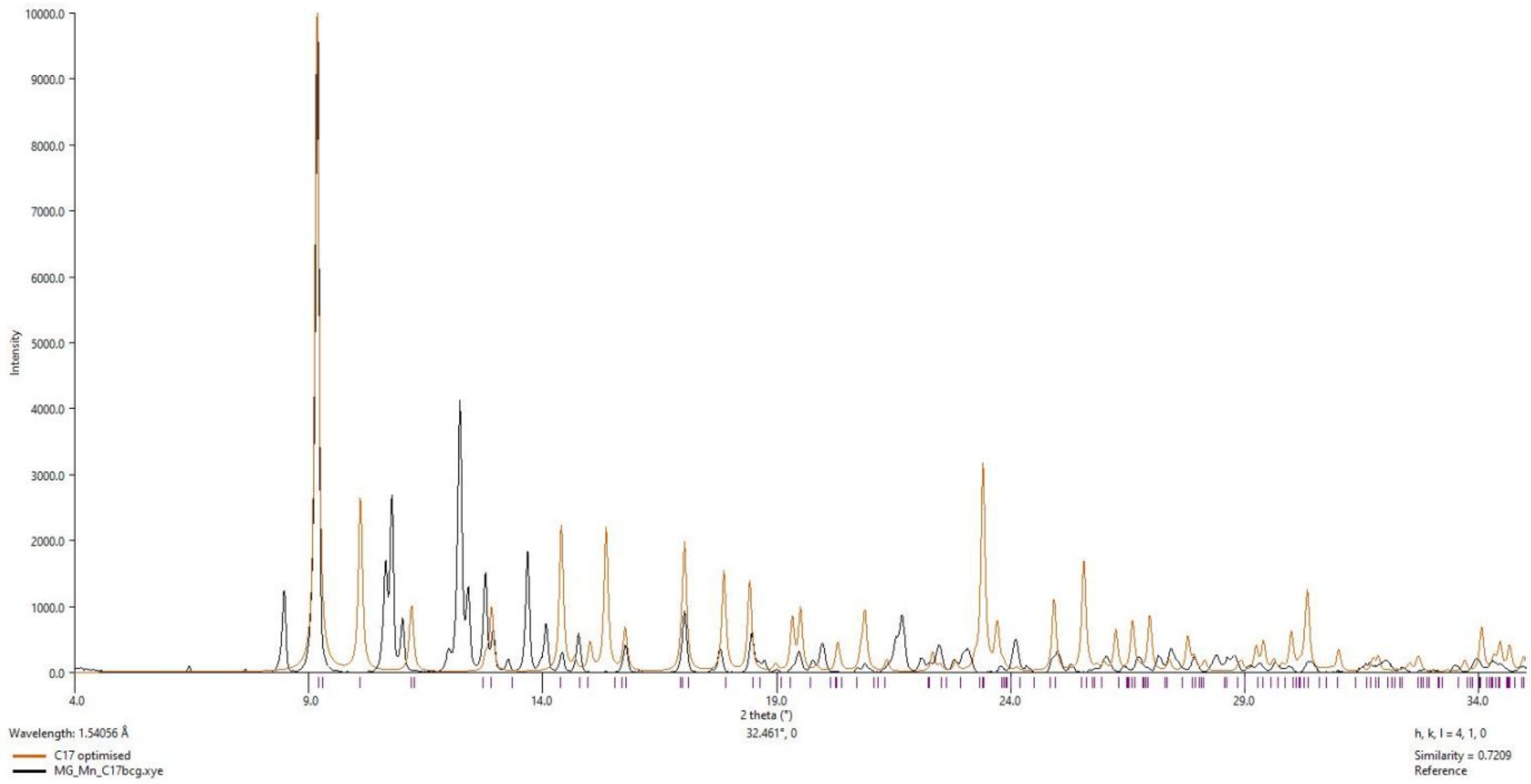

Figure S30. Comparison of the powder X-ray diffraction (PXRD) patterns of complex C17 and the corresponding calculated pattern derived from the SCXRD structure.

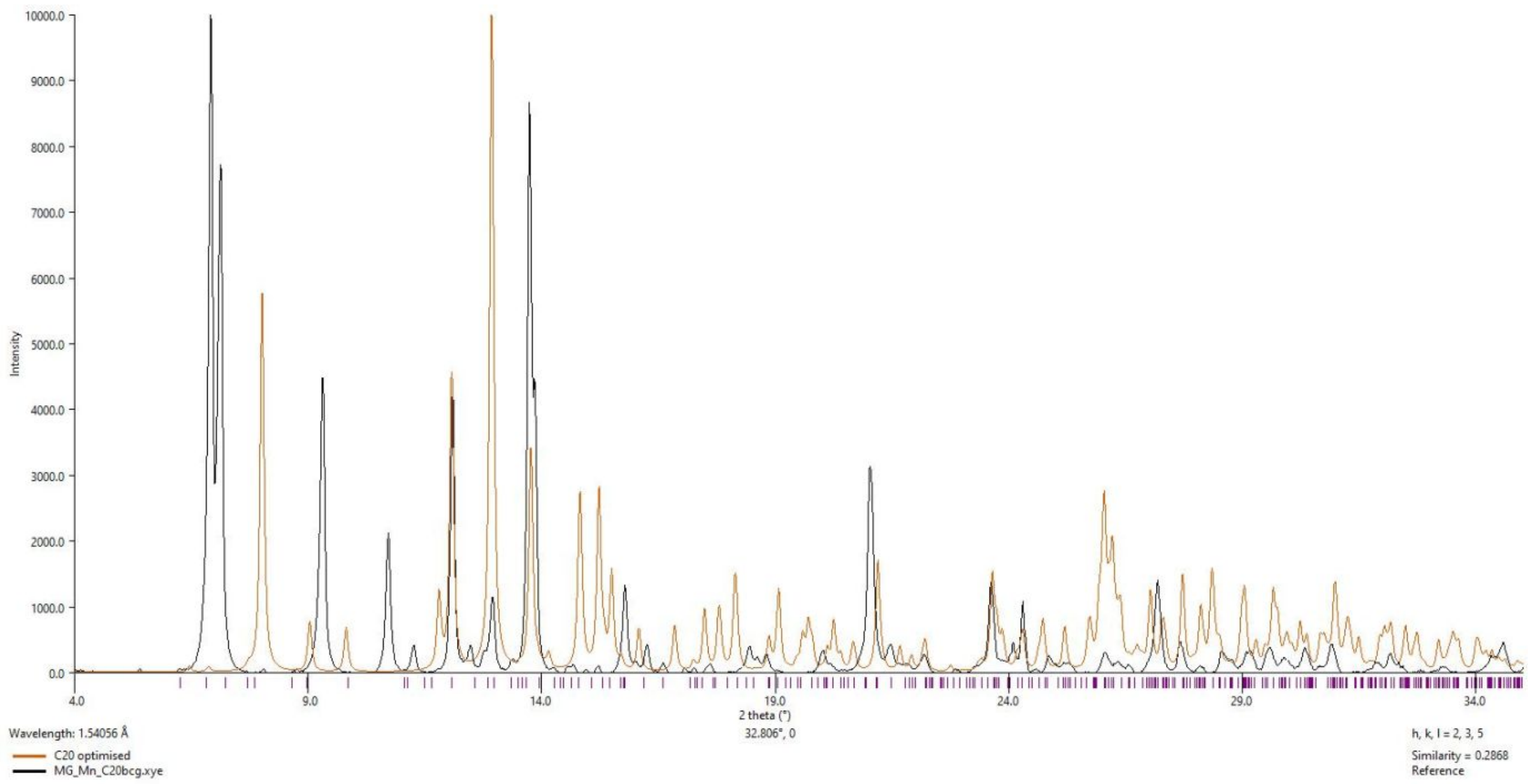

Figure S31. Comparison of the powder X-ray diffraction (PXRD) patterns of complex C20 and the corresponding calculated pattern derived from the SCXRD structure.

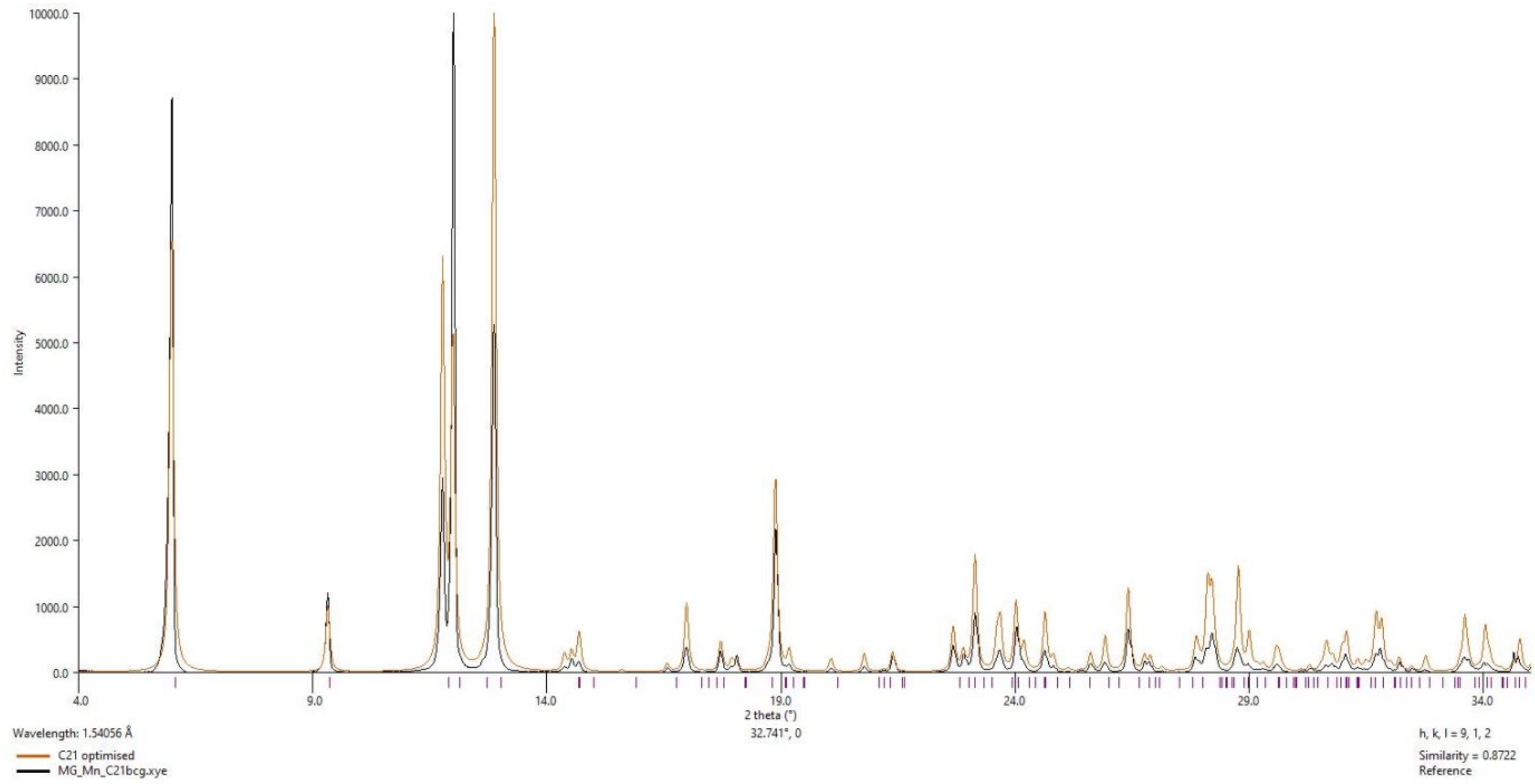

Figure S32. Comparison of the powder X-ray diffraction (PXRD) patterns of complex C21 and the corresponding calculated pattern derived from the SCXRD structure.

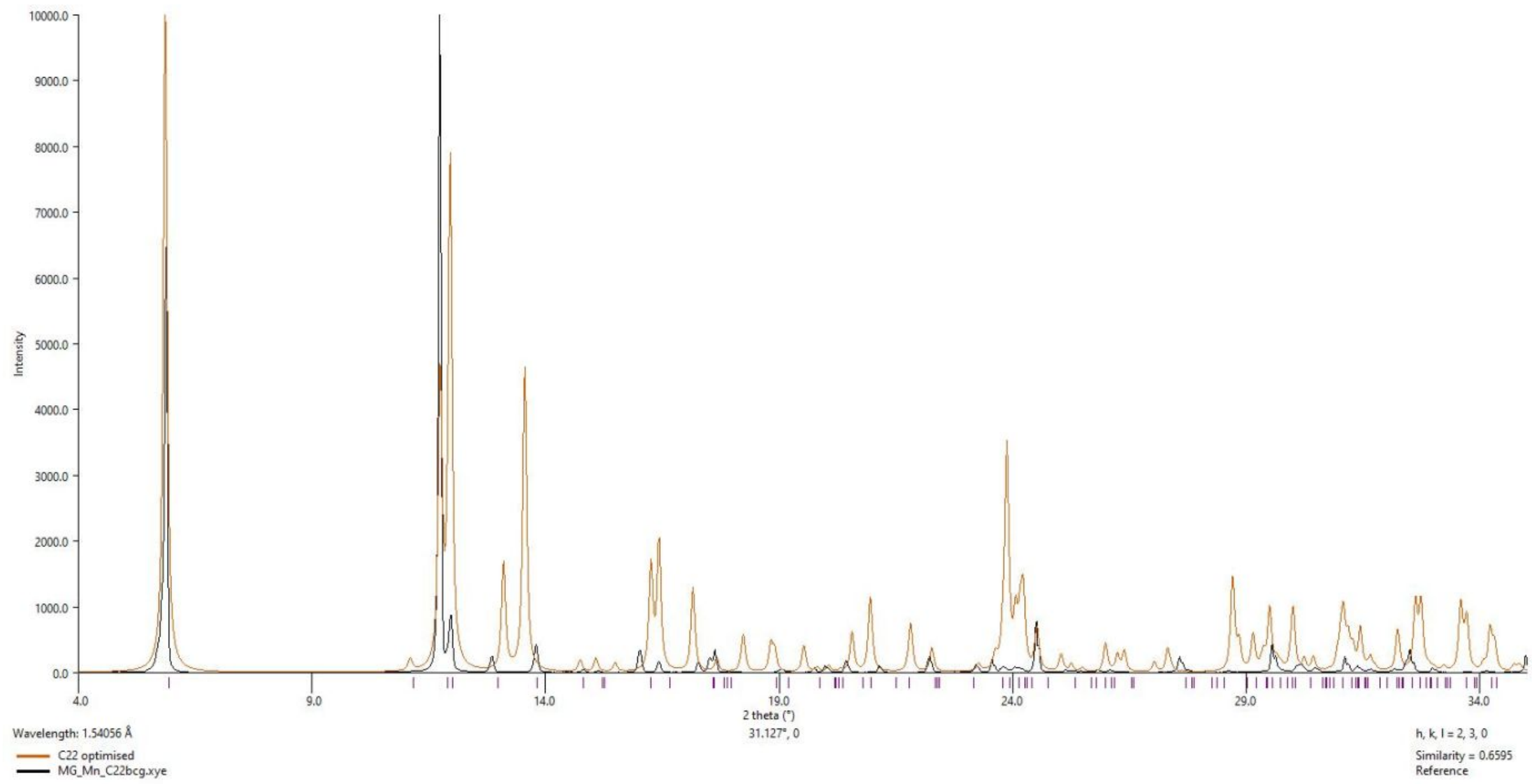

Figure S33. Comparison of the powder X-ray diffraction (PXRD) patterns of complex C22 and the corresponding calculated pattern derived from the SCXRD structure.

MECHANISTIC STUDIES

NMR studies

1. Reaction of Mn complex with $PhSiH_3$

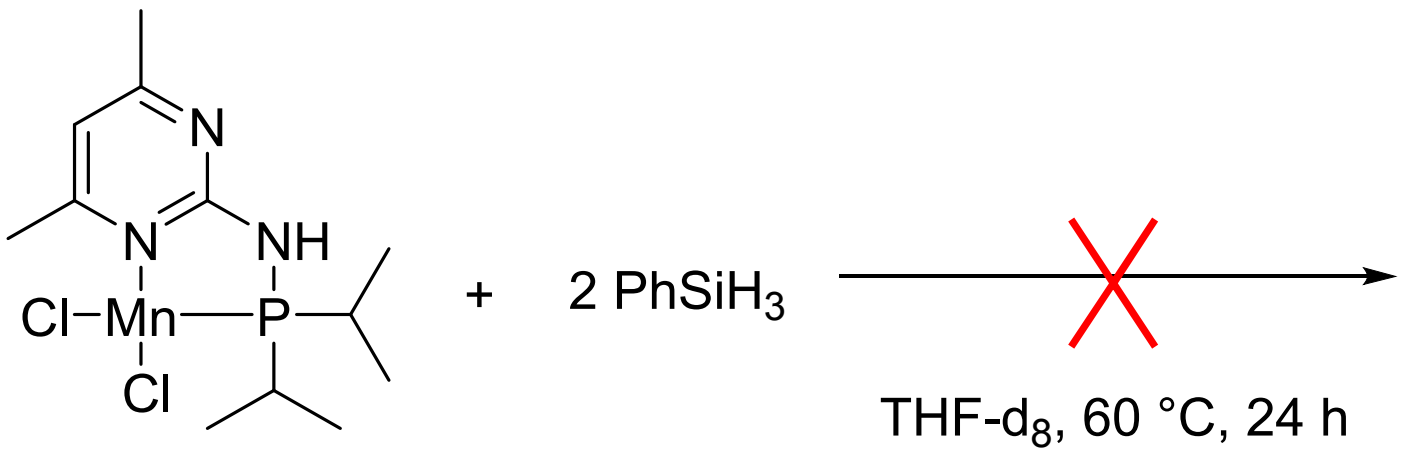

To a J. Young NMR tube under an inert atmosphere (glovebox), the catalyst (1.0 equiv., 0.05 mmol), THF-d_8 (500 µL) and $PhSiH_3$ (2.0 equiv., 0.1 mmol) were added. After sealing the tube, the solution was briefly mixed and transferred outside the glovebox. The mixture was then heated at 60 °C for 24 hours and subsequently was analyzed by ^{1}H and ^{13}C NMR and ^{29}Si spectroscopy to characterize species present in solution.

Results: The reaction mixture remained paramagnetic, consistent with the Mn(II) oxidation state of the starting complex. As a result, no interpretable NMR resonances could be obtained. Subsequent GC-MS analysis of the reaction mixture (after dilution with hexane containing trace water) showed only trace amounts of the disiloxane PhH_2Si-O-SiH_2Ph, at the very limit of detection. Any trace disiloxane likely arises from minor hydrolysis of chlorosilane intermediates, which could form if $PhSiH_3$ exchanged H with Cl from Mn, followed by reaction with residual water in hexane. These results indicate that no significant chloride/hydride exchange occurs under these conditions, in contrast to mechanisms proposed for related cobalt systems.[19,28]

2. Reaction of Mn complex with *t*-$BuMe_2SiOH$

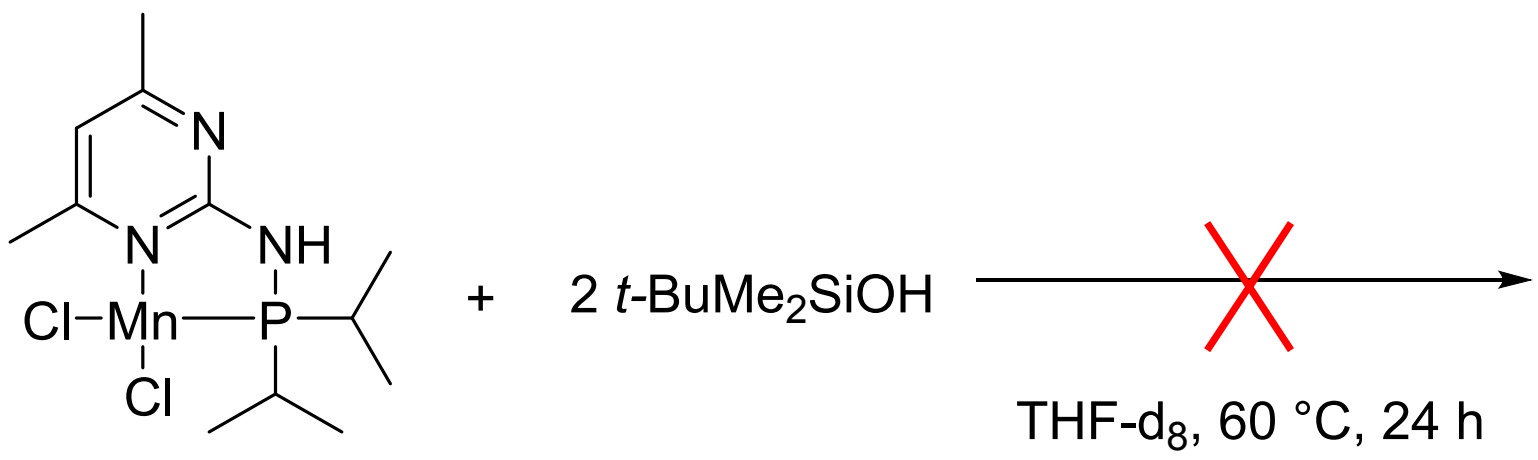

To a J. Young NMR tube under an inert atmosphere (glovebox), the catalyst (1.0 equiv., 0.05 mmol), THF-d_8 (500 µL) and *t*-$BuMe_2SiOH$ (2.0 equiv., 0.1 mmol) were added. After sealing the tube, the solution was briefly mixed and transferred outside the glovebox. The mixture was then heated at 60 °C for 24 hours and subsequently was analyzed by ^{1}H and ^{13}C NMR and ^{29}Si spectroscopy to characterize species present in solution.

Results: The reaction mixture remained paramagnetic, consistent with the Mn(II) oxidation state of the starting complex. As a result, no interpretable NMR resonances could be obtained.

IR studies

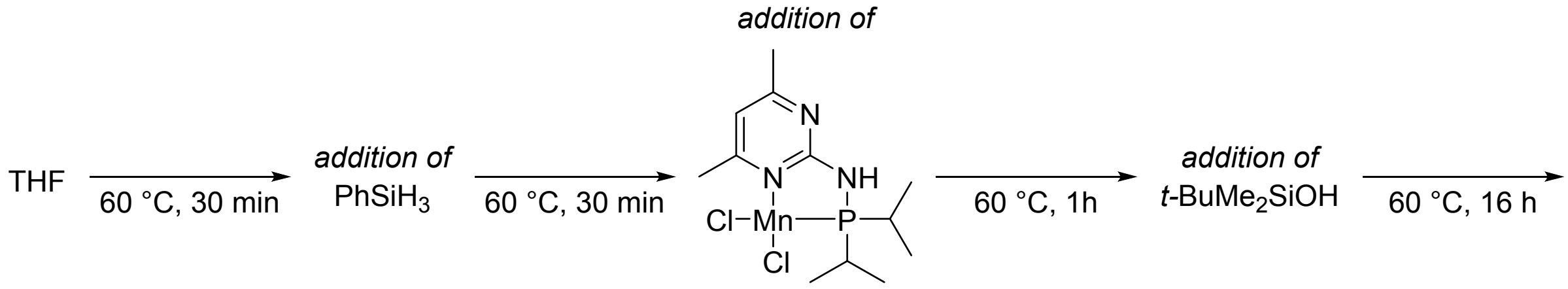

A 50 mL Schlenk flask equipped with a magnetic stirring bar was charged with THF (1500 µL) under an inert gas atmosphere and heated to 60 °C. Continuous real-time FT-IR monitoring was initiated at this stage and maintained throughout the entire experiment. $PhSiH_3$ (1 equiv., 0.1 mmol) was then added, and the mixture was heated for an additional 30 min. Subsequently, the catalyst (1 equiv., 0.1 mmol) was introduced, and heating at 60 °C was continued. After 1 h, *t*-$BuMe_2SiOH$ (2 equiv., 0.2 mmol) was added, and the reaction mixture was further heated for 16 h.

Results: The Si-H bending band at ca. 920 cm^{-1} (δ(Si-H)) and the Si-H stretching band at ca. 2160 cm^{-1} (ν(Si-H), attributable to $PhSiH_3$) showed no detectable shift or change in intensity upon addition of the Mn catalyst. In the 2000-1600 cm^{-1} region, where Mn-H stretching vibrations have been reported for certain manganese hydride species,[29,30] no new bands or other significant spectral changes were observed during the hour following catalyst addition. Upon addition of *t*-$BuMe_2SiOH$, the Si-H bending band at 920 cm^{-1} gradually decreased over time, consistent with consumption of the silane and formation of siloxane.

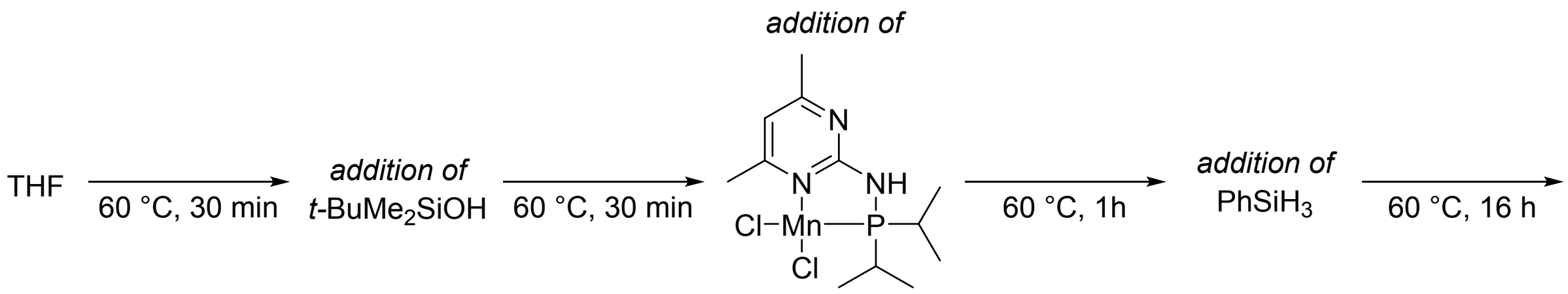

A 50 mL Schlenk flask equipped with a magnetic stirring bar was charged with THF (1500 µL) under an inert gas atmosphere and heated to 60 °C. Continuous real-time FT-IR monitoring was initiated at this stage and maintained throughout the entire experiment. *t*-$BuMe_2SiOH$ (2 equiv., 0.2 mmol) was then added, and the mixture was heated for an additional 30 min. Subsequently, the catalyst (1 equiv., 0.1 mmol) was introduced, and heating at 60 °C was continued. After 1 h, $PhSiH_3$ (1 equiv., 0.1 mmol) was added, and the reaction mixture was further heated for 16 h.

Results: Similarly to the experiment where silane was the first substrate added, no detectable shift or change in intensity of bands of silanol, upon addition of the Mn catalyst, was observed. No new bands or other significant spectral changes were observed during the hour following catalyst addition. Upon addition of silane to the system, the Si-H bending band appeared at 920 cm^{-1} and then gradually decreased over time, consistent with consumption of the silane and formation of siloxane.

Poisoning tests

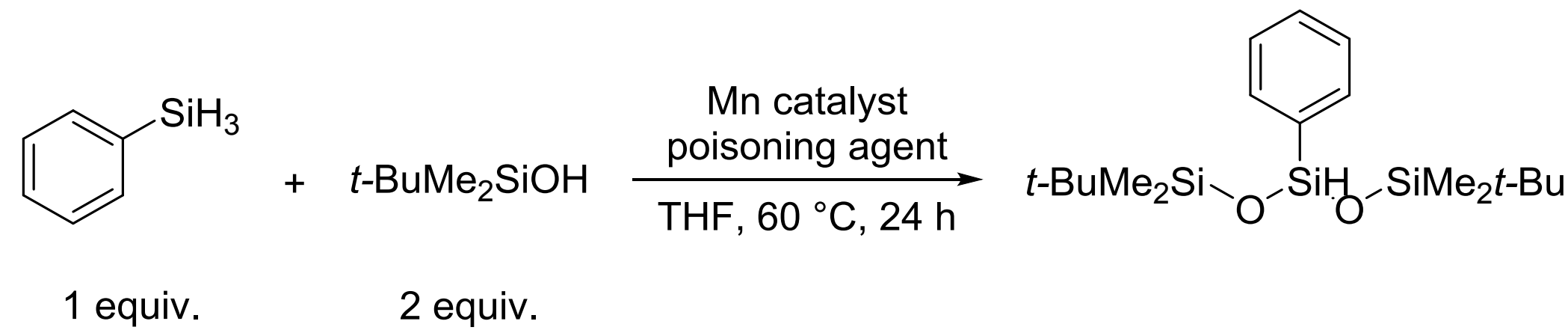

To a 12 mL vial equipped with a magnetic stirring bar under an inert atmosphere (glovebox) the catalyst (0.03 equiv.,), $PhSiH_3$ (1), *t*-$BuMe_2SiOH$ (2), poisoning agent, THF (400 uL) and n-dodecane (used as internal standard) were added. The reaction mixture was stirred at 60 °C for 24 hours. Reaction progress was monitored by GC-MS by determining the conversion of the silane using n-dodecane as the internal standard. Results are summarized in **Table S13**.

Table S13. Summary of catalyst poisoning tests.

	**Poisoning agent**	**Tested aspect**	**Expected effect if positive**	**Observed effect**	**Silane conversion**	**Interpretation**
1	Hg (1000 eq.)	Heterogeneous mechanism	Reaction inhibited (metal surface blocked)	No inhibition	99%	Rules out nanoparticle catalysis
2	QuadraPure (100mg)	Homogenous mechanism	Inhibition (binding of soluble form of metals)	Inhibition	33%[a]	Supports homogeneous catalysis
3	TEMPO (1 eq.)	Radical mechanism	Reaction inhibited (radicals quenched)	No inhibition	99%	Rules out radical involvement
4	1,10-phenanthroline	Silane σ-donation	Reaction inhibited (coordination sites occupied)	Inhibition	51%	Supports silane activation via σ-donation

a- Mono- to di-coupling selectivity = 78:22.

Kinetic profile

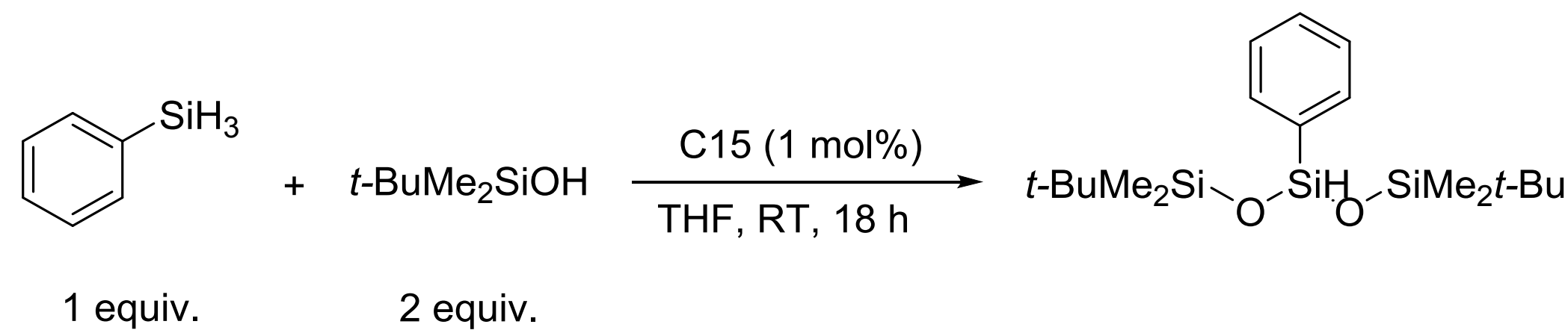

To a 12 mL vial equipped with a magnetic stirring bar under an inert atmosphere (glovebox) the catalyst (**C15**, 1 mol%), dry THF (500 uL), Trimethoxybenzene (internal standard; 18 mg), silanol (0.25 mmol) and silane (0.125 mmol) were added. The reaction mixture was stirred at room temperature for 18 hours. Sample for GC-MS analysis was taken at the beginning of reaction (where t0 = 0 hours) and each time after given amount of time. Peak area values obtained from GC-MS are provided in **Table S14**.

Table S14. Raw data obtained from GC-MS of the reaction between phenylsilane and tert-Butyldimethylsilanol. In brackets peak areas are provided.

	Silane	Silanol	Monosiloxane	$(MeO)_3Ph$	Disiloxane
t0	1706600	2460391	0	1835605	0
t1	589892	1085578	629728	971162	85627
t2	497249	1354011	2802329	2041298	1702873
t4	61384	386804	1672037	1393021	4026928
t18	0	0	557272	2707398	14309792

Table S15. Normalized data obtained by dividing each value by peak area of internal standard.

	Silane	Silanol	Monosiloxane	Disiloxane
t0	0,929721	1,340371	0	0
t1	0,607408	1,117814	0,648427	0,08817
t2	0,243595	0,663309	1,372817	0,834211
t4	0,044065	0,277673	1,200296	2,890788
t18	0	0	0,205833	5,285441

Table S16. Percentage share obtained from normalized data. Values were summed up, divided by the sum and equated to 100%.

	Silane	Silanol	Monosiloxane	Disiloxane
t0	40,96	59,04	0,00	0,00
t1	24,67	45,41	26,34	3,58
t2	7,82	21,30	44,09	26,79
t4	1,00	6,29	27,20	65,51
t18	0,00	0,00	3,75	96,25

Data obtained from **Table S16** was used in the making of diagram in manuscript (**Figure 6**).

Kinetic experiments were performed to investigate the mechanism of the manganese-catalyzed dehydrogenative coupling of silanols with hydrosilanes. Initial rates (v_0) were determined under varying concentrations of the manganese catalyst, diphenylsilane and *tert*-butyldimethylsilanol.

Rate-concentration analysis revealed a zero-order dependence on silanol, a second-order dependence on silane, and a fractional order of 0.33 with respect to the total catalyst concentration. While structural characterization indicates the precatalyst exists as a chloride-bridged dimer, the observed one-third fractional order suggests a more complex speciation in the solution state.

$$v = k_{obs}[\text{catalyst}]^{1/3}[\text{Ph}_2\text{SiH}_2]^2[t\text{-BuMe}_2\text{SiOH}]^0$$

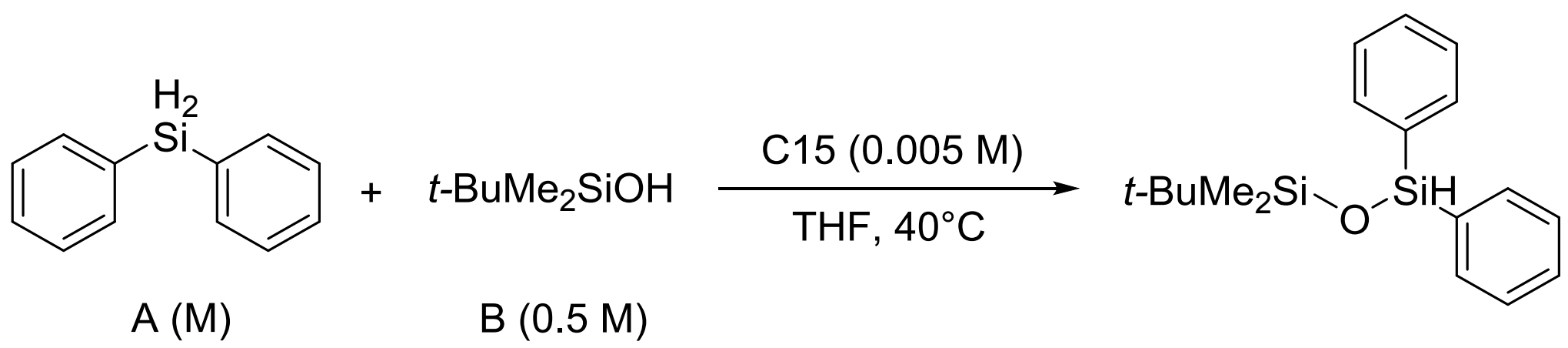

To a 12 mL vial equipped with a magnetic stirring bar under an inert atmosphere (glovebox) the catalyst (**C15**, 0.005 M), dry THF (to bring each reaction to a final volume of 500 µL), decane (internal standard; 24 mg), silanol B (0.5 M) and silane A (M) were added. The reaction mixture was stirred at 40°C room for 6 hours. Sample for GC-MS analysis was taken at the beginning of reaction and each time after given amount of time. The concentration of the product was calculated based on a calibration curve.

Table S17. Kinetic studies for silane order.

**Contentration of product for different concentrations of Ph_2SiH_2**						
Entry	Time [sec]	0.25 M	0.5 M	0.75 M	1.0 M	1.25 M
1	1800	0,042	0,042	0,053	0,063	0,088
2	3600	0,045	0,047	0,070	0,096	0,133
3	5400	0,047	0,056	0,082	0,126	0,211
4	7200	0,049	0,062	0,091	0,139	0,263
5	9000	0,049	0,066	0,103	0,192	0,285
6	10800	0,051	0,072	0,124	0,198	0,366
7	12600	0,053	0,079	0,128	0,227	0,419
4	14400	0,054	0,081	0,140	0,253	0,434
5	16200	0,055	0,089	0,146	0,285	0,515
10	18000	0,057	0,092	0,160	0,291	0,582
11	21600	0,061	0,105	0,194	0,368	0,622

Table S18. Concentration of product for different concentrations of Ph_2SiH_2

**Contentration of product for different concentrations of Ph_2SiH_2**		
Entry	A [M]	initial rate
1	0,25	8,77E-07
2	0,5	3,08E-06
3	0,75	6,69E-06
4	1	1,47E-05
5	1,25	2,81E-05

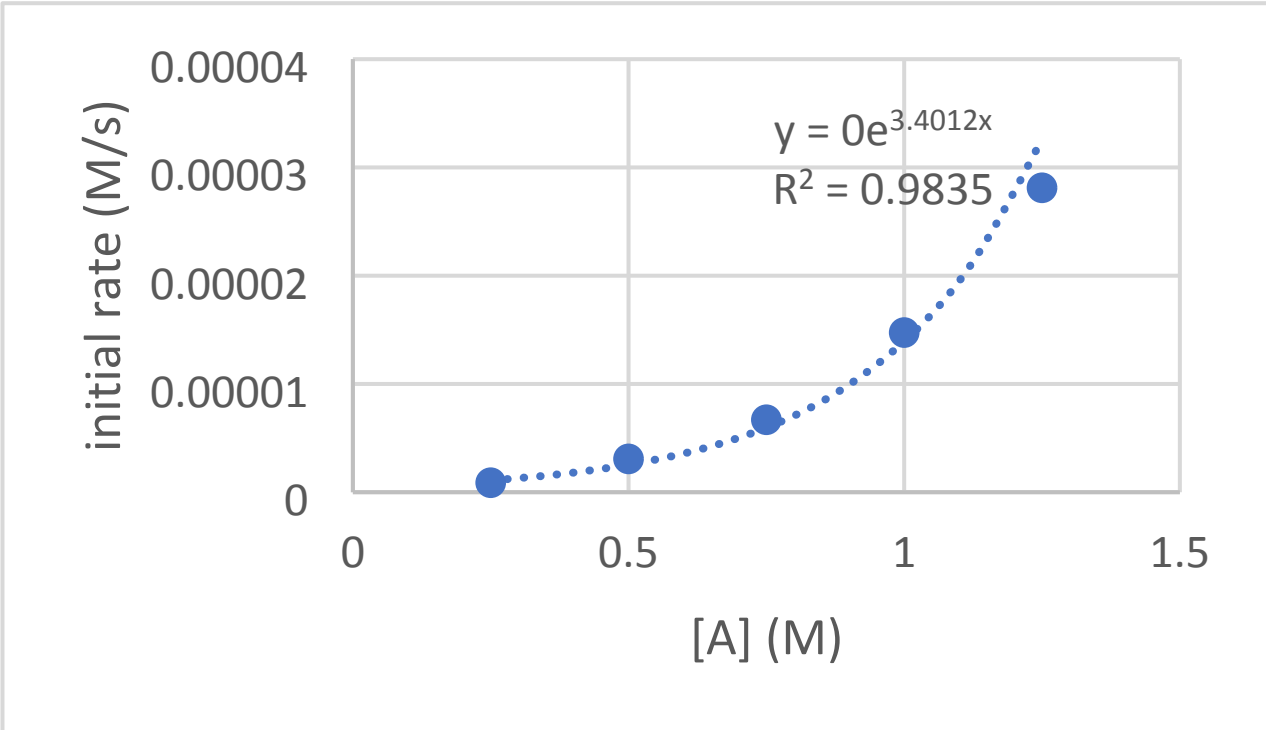

Figure S34. Graphic method showcasing initial rates vs silane concentration.

Table S19. Concentration of product for different concentrations of Ph_2SiH_2

**Logarytmic method**		
Entry	log(Si-H [M])	log(initial rate)
1	-0,60	-6,06
2	-0,30	-5,51
3	-0,12	-5,17
4	0,00	-4,83
5	0,10	-4,55

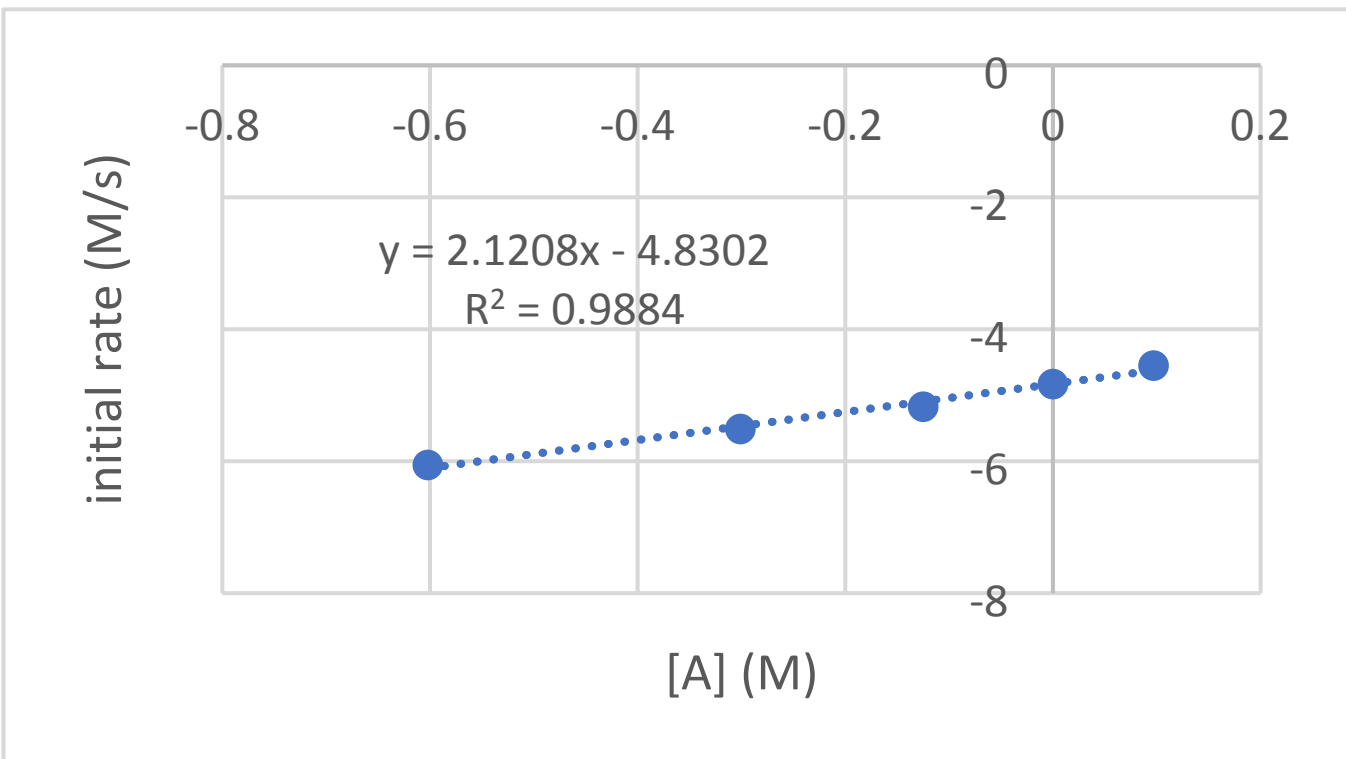

Figure S35. Logarytmic method showcasing angular coefficient.

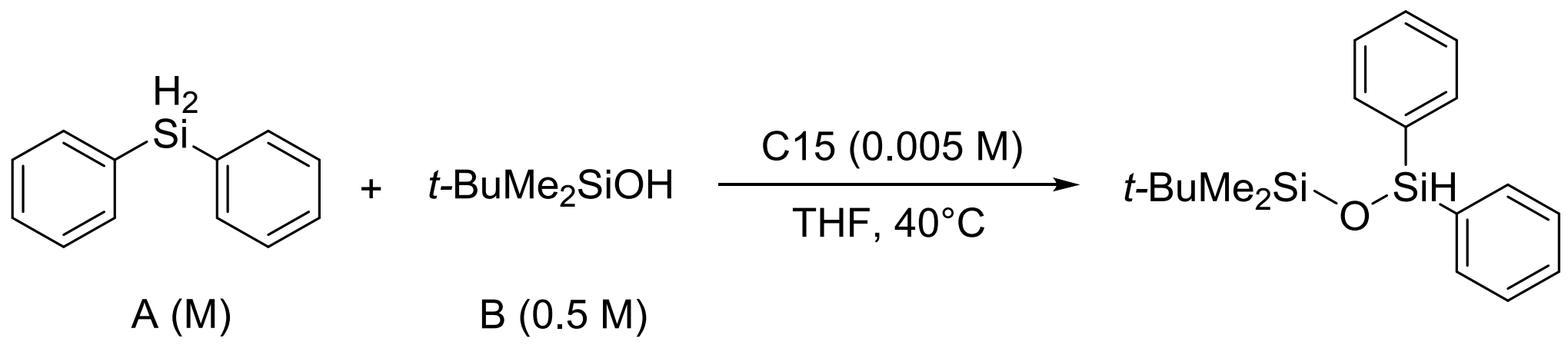

To a 12 mL vial equipped with a magnetic stirring bar under an inert atmosphere (glovebox) the catalyst (**C15**, 0.005 M), dry THF (to bring each reaction to a final volume of 500 µL), decane (internal standard; 24 mg), silanol B (M) and silane A (0.5 M) were added. The reaction mixture was stirred at 40°C room for 6 hours. Sample for GC-MS analysis was taken at the beginning of reaction and each time after given amount of time. The concentration of the product was calculated based on a calibration curve.

Table S20. Kinetic studies for silanol order.

Concentration of product for different concentrations of tBuMe2SiOH						
Entry	Time [sec]	0.5 M	1.0 M	1.5 M	2.0 M	2.5 M
1	1800	0,042	0,046	0,047	0,045	0,047
2	3600	0,047	0,054	0,052	0,051	0,054
3	5400	0,056	0,063	0,055	0,055	0,060
4	7200	0,062	0,064	0,062	0,065	0,062
5	9000	0,066	0,071	0,073	0,067	0,075
6	10800	0,072	0,080	0,074	0,071	0,081
7	12600	0,079	0,089	0,082	0,079	0,086
4	14400	0,081	0,094	0,085	0,083	0,095
5	16200	0,089	0,102	0,093	0,088	0,099
10	18000	0,092	0,104	0,096	0,096	0,108
11	21600	0,105	0,116	0,105	0,117	0,124

Table S21. Concentration of product for different concentrations of silanol.

Concentration of product for different concentrations of t-BuMe$_2$SiOH		
Entry	Si-OH [M]	initial rate
1	0,5	3,08E-06
2	1,0	3,59E-06
3	1,5	3,06E-06
4	2,0	3,35E-06
5	2,5	3,85E-06

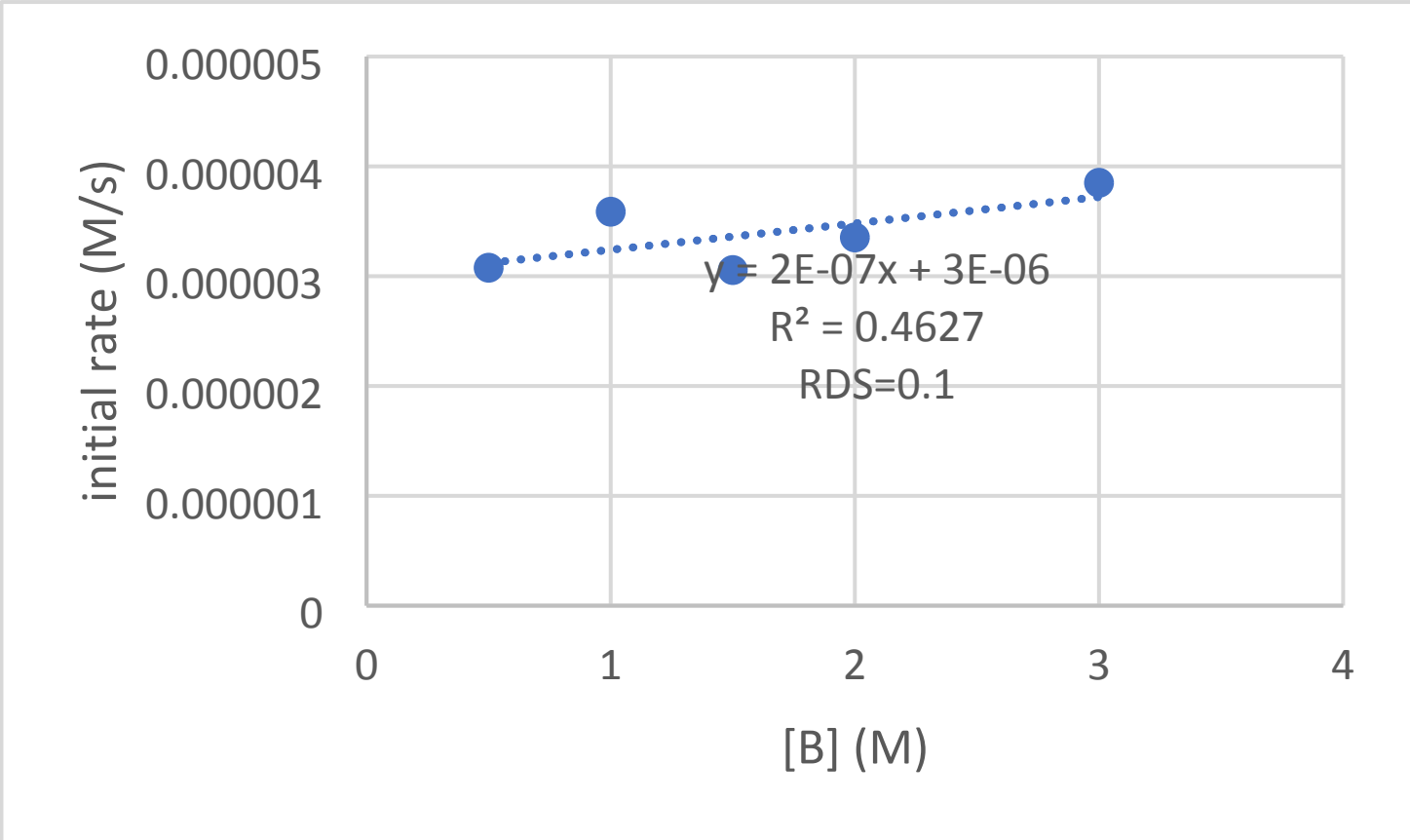

Figure S36. Graphic method showcasing initial rates vs silanol concentration.

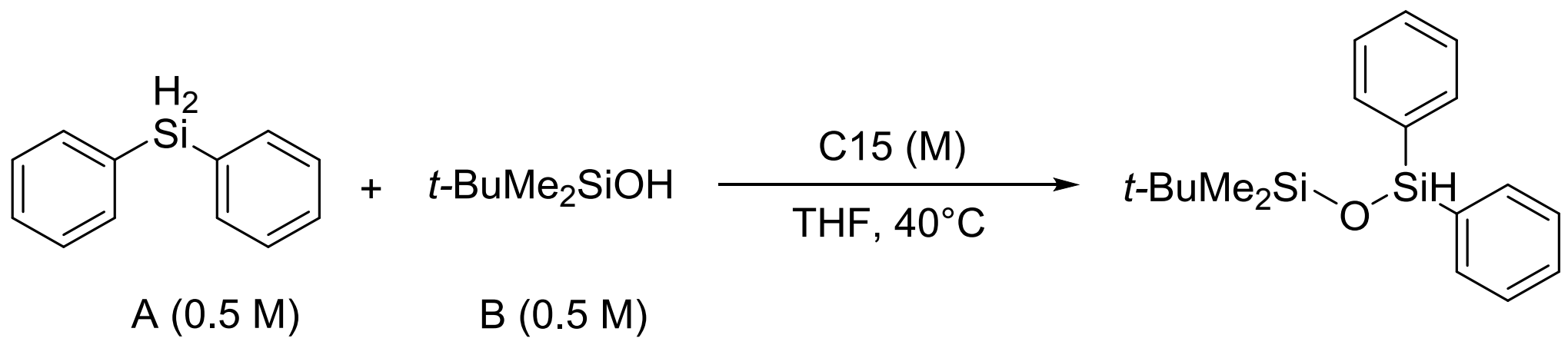

To a 12 mL vial equipped with a magnetic stirring bar under an inert atmosphere (glovebox) the catalyst (**C15**, M), dry THF (to bring each reaction to a final volume of 500 µL), decane (internal standard; 24 mg), silanol B (0.5 M) and silane A (0.5 M) were added. The reaction mixture was stirred at 40°C room for 6 hours. Sample for GC-MS analysis was taken at the beginning of reaction and each time after given amount of time. The concentration of the product was calculated based on a calibration curve.

Table S22. Kinetic studies for catalyst order.

Concentration of product for different concentrations of catalyst						
Entry	Time [sec]	0.0025 M	0.005 M	0.01125 M	0.0175 M	0.025 M
1	1800	0,044	0,042	0,045	0,045	0,048
2	3600	0,046	0,047	0,047	0,047	0,063
3	5400	0,051	0,056	0,055	0,053	0,072
4	7200	0,053	0,062	0,060	0,056	0,079
5	9000	0,060	0,066	0,070	0,071	0,088
6	10800	0,059	0,072	0,069	0,080	0,103
7	12600	0,069	0,079	0,083	0,089	0,108
4	14400	0,075	0,081	0,089	0,095	0,116
5	16200	0,078	0,089	0,098	0,107	0,130
10	18000	0,083	0,092	0,109	0,115	0,135
11	21600	0,091	0,105	0,120	0,134	0,162

Table S23. Concentration of product for different concentrations of catalyst.

Concentration of product for different concentrations of catalyst		
Entry	cat. [M]	initial rate
1	0,0025	2,50E-06
2	0,0050	3,08E-06
3	0,0113	3,99E-06
4	0,0175	4,71E-06
5	0,0250	5,46E-06

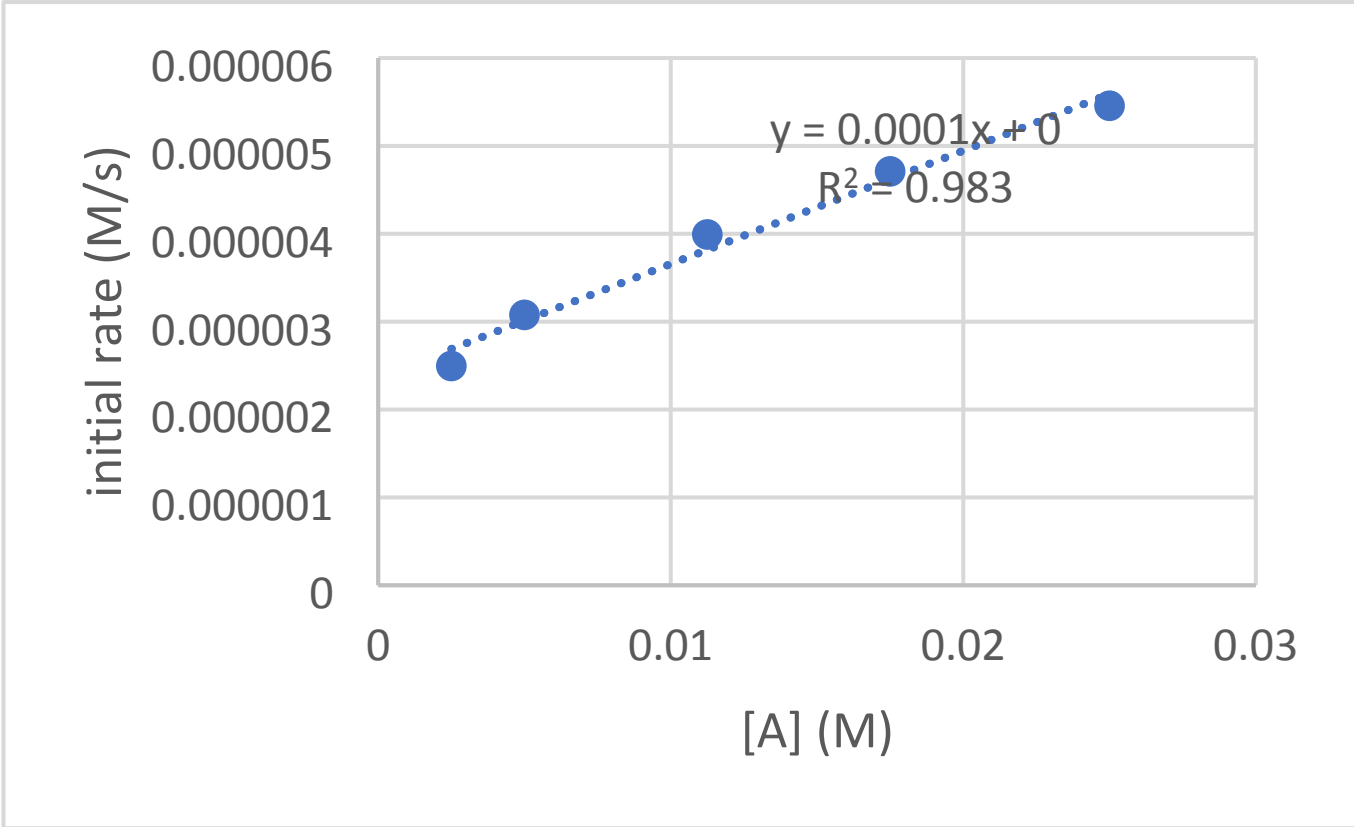

Figure S37. Graphic method showcasing initial rates vs silanol concentration.

Table S24. Concentration of product for different concentrations of catalyst.

Logarytmic method		
Entry	log(cat. [M])	log(initial rate)
1	-2,60	-5,60
2	-2,30	-5,51
3	-1,95	-5,40
4	-1,76	-5,33
5	-1,60	-5,26

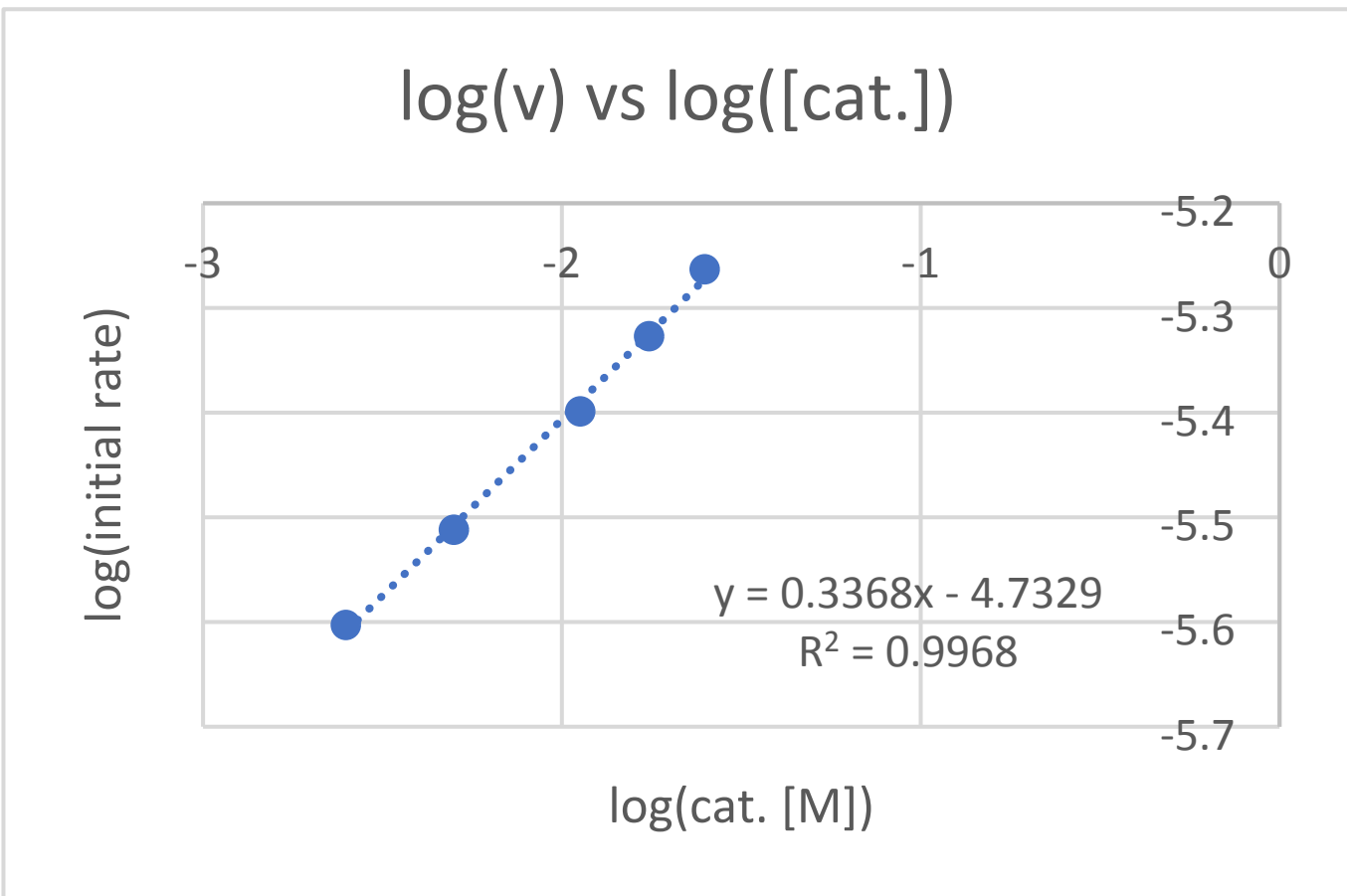

Figure S38. Logarytmic method showcasing angular coefficient.

To further elucidate the nature of the rate-limiting step and probe the precise mechanism of Si-H bond cleavage, kinetic isotope effect (KIE) studies were conducted utilizing deuterated diphenylsilane (Ph_2SiD_2) under optimized reaction conditions. Parallel initial rate measurements provided a distinct primary KIE value of k_H/k_D = 3.0. This substantial value demonstrates that significant Si-H bond lengthening and cleavage occur probably directly within the transition state of the rate-determining step.

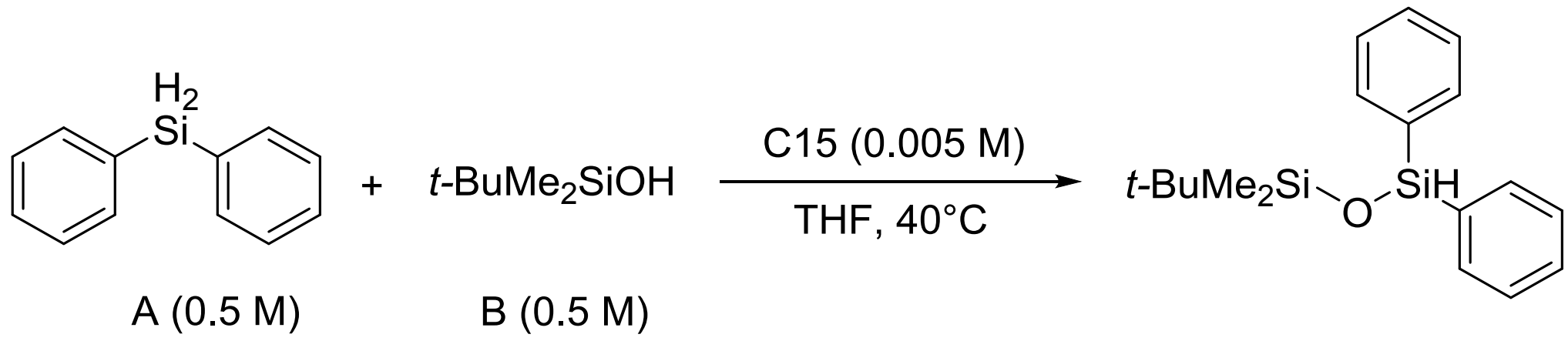

A (0.5 M) B (0.5 M)

To a 12 mL vial equipped with a magnetic stirring bar under an inert atmosphere (glovebox) the catalyst (**C15**, 0.005 M), dry THF (to bring each reaction to a final volume of 500 µL), decane (internal standard; 24 mg), silanol B (0.5 M) and silane A (0.5 M) were added. The reaction mixture was stirred at 40°C room for 6 hours. Sample for GC-MS analysis was taken at the beginning of reaction and each time after given amount of time. The concentration of the product was calculated based on a calibration curve.

Table S25. Concentration of product in reaction with Ph_2SiH_2 for KIE studies.

Entry	Time [sec]	Concentration of product [M]
1	1800	0,042
2	3600	0,047
3	5400	0,056
4	7200	0,062
5	9000	0,066
6	10800	0,072
7	12600	0,079
8	14400	0,081
9	16200	0,089
10	18000	0,092
11	21600	0,105

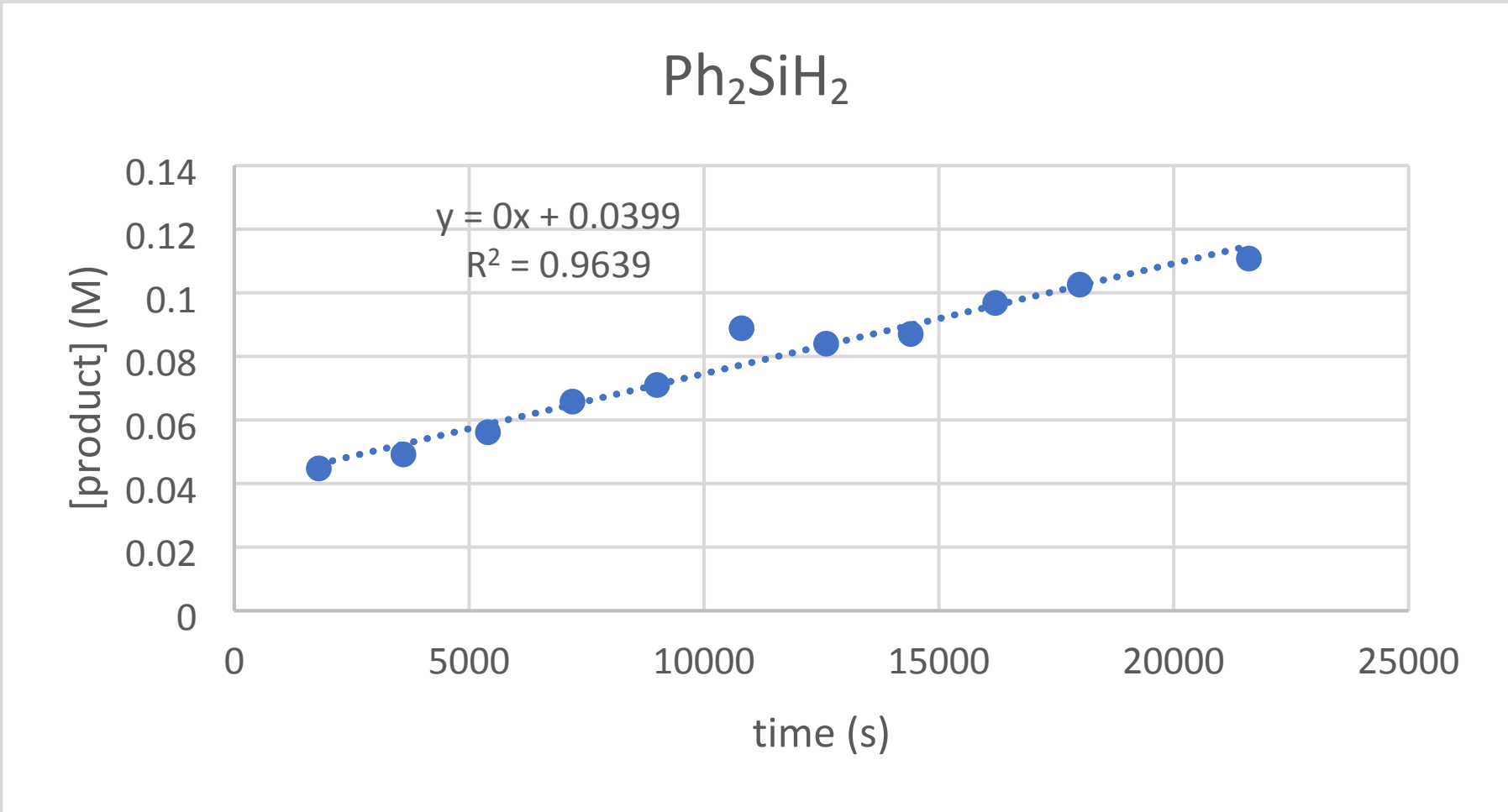

Figure S39. Chart showcasing change in product concentration over time.

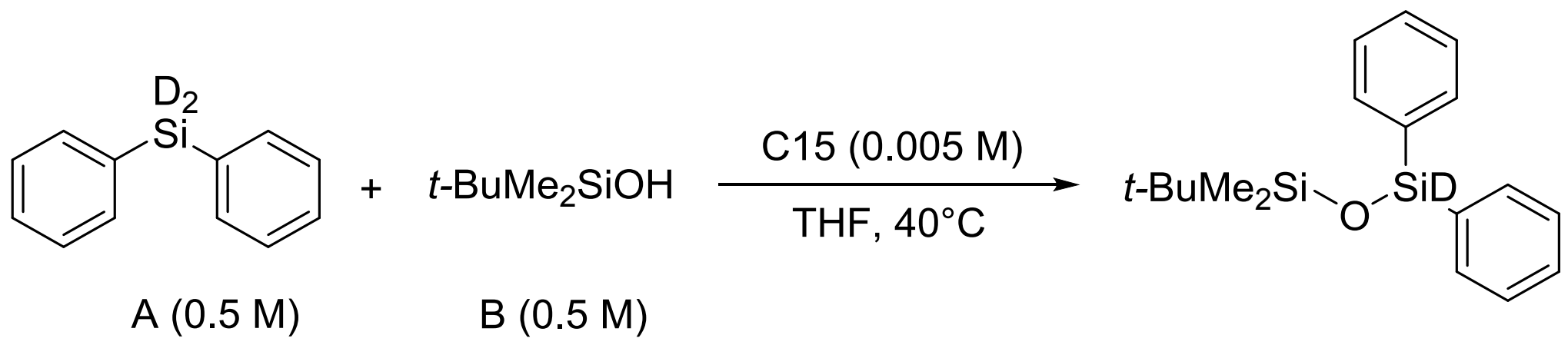

To a 12 mL vial equipped with a magnetic stirring bar under an inert atmosphere (glovebox) the catalyst (**C15**, 0.005 M), dry THF (to bring each reaction to a final volume of 500 µL), decane (internal standard; 24 mg), silanol B (0.5 M) and deutered silane A (0.5 M) were added. The reaction mixture was stirred at 40°C room for 6 hours. Sample for GC-MS analysis was taken at the beginning of reaction and each time after given amount of time. The concentration of the product was calculated based on a calibration curve.

Table S26. Concentration of product in reaction with Ph_2SiD_2 for KIE studies.

Entry	Time [sec]	Concentration of product [M]
1	1800	0,044
2	3600	0,047
3	5400	0,049
4	7200	0,051
5	9000	0,052
6	10800	0,055
7	12600	0,054
8	14400	0,055
9	16200	0,061
10	18000	0,062
11	21600	0,064

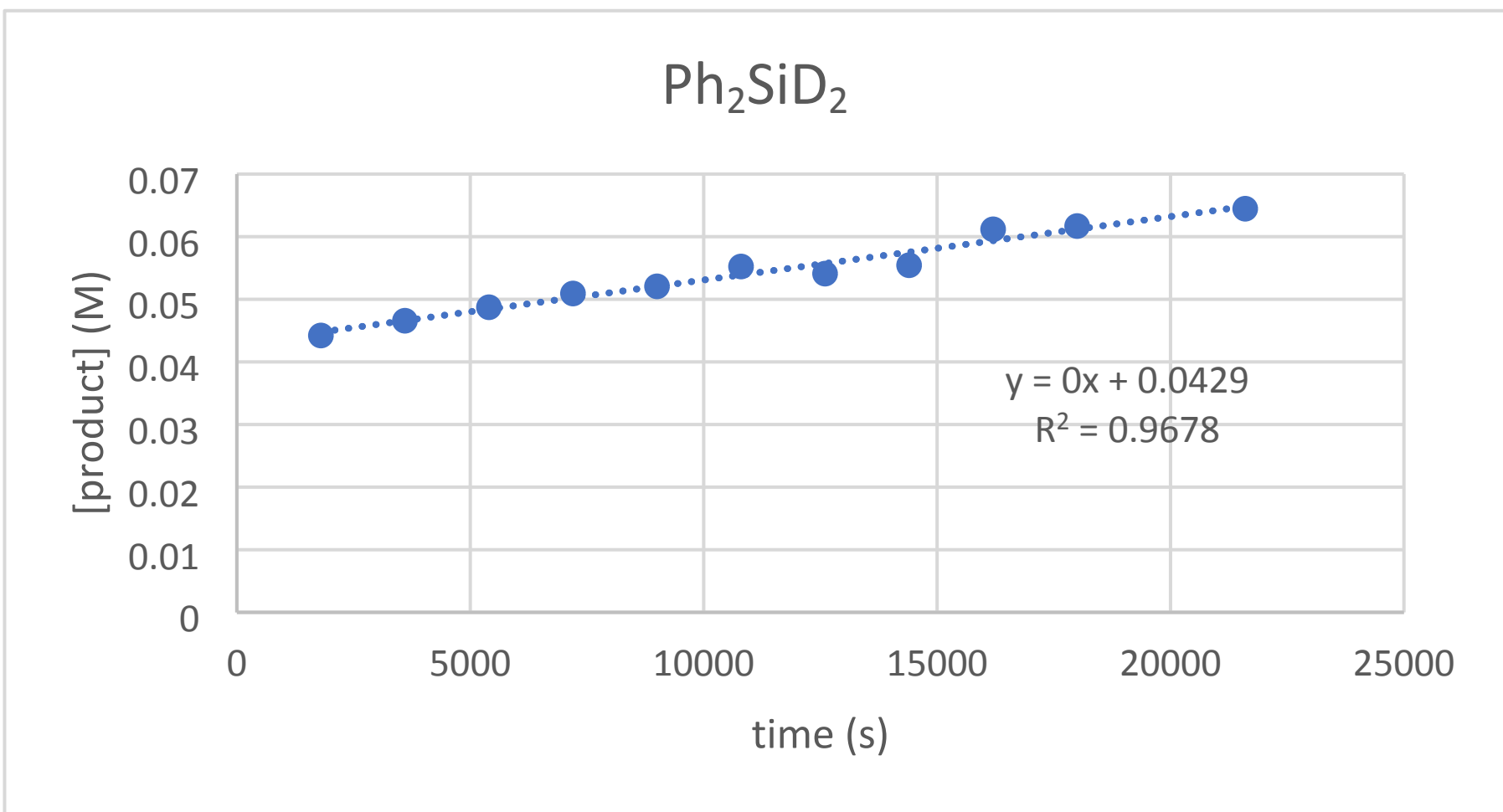

Figure S40. Chart showcasing change in product concentration over time with use of deutered silane.

Based on the provided figures and kinetic data, KIE = 3.0, obtained by dividing angular coefficients of silane Ph_2SiH_2 (3,08E-06) and deutered silane Ph_2SiD_2 (1,01E-06).

CHARACTERIZATION DATA FOR ALL PRODUCTS

Ligands **L1**-**L16** were prepared according to the previously reported method.[2] All of the NMR spectra and X-ray analysis are available under said Supporting Information.

Manganese(II) complexes exhibited strong paramagnetism; therefore, their NMR spectra could not be obtained. Several of these complexes were successfully recrystallized, and suitable single crystals were subjected to X-ray diffraction analysis, which confirmed the proposed structures. In contrast, manganese(I) complexes were not paramagnetic, allowing NMR spectra to be recorded (except **C20** given it's very poor solubility). Additionally, X-ray diffraction analysis was performed for **C20**, **C21**, and **C22**, which are representative of the manganese(I) complex series. Moreover, we provide elemental analysis for all of the complexes.

Elemental analysis of obtained manganese complexes

Procedure: The measurements were performed using a Thermo Scientific FLASH 2000 elemental analyzer. The FLASH 2000 analyzer is based on dynamic combustion technology. First, the samples are weighed in tin capsules (approx. 2–4 mg) and introduced into the reactor via an autosampler along with a specific, precisely defined portion of oxygen. After combustion at a temperature of 900–1000°C, the combustion gases are carried by a helium flow into a copper-filled reactor furnace and then through a water trap to a chromatographic column, which separates the individual products from one another. Finally, the separated gases are detected by a thermal conductivity detector (TCD). The data analysis software - Thermo Scientific EagerXperience - automatically generates and displays a complete report at the end of each measurement cycle.

Table S27. NCH Content Markings.

Structure	Material	N %	C %	H %
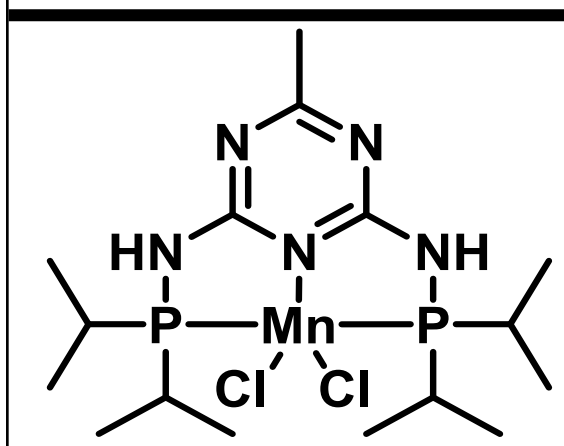	C1 (run 1)	14,68	39,92	7,10
	C1 (run 2)	14,69	39,86	7,09
	C1 - Calculated	14,49	39,77	6,88

Structure	Material	N %	C %	H %
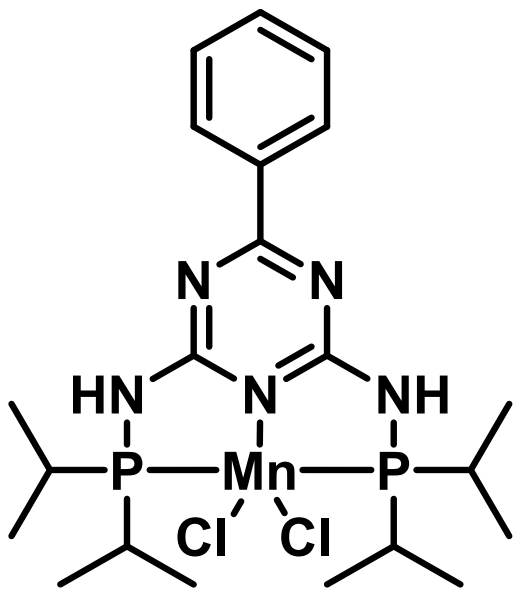	C2 (run 1)	12,15	46,15	6,29
	C2 (run 2)	12,86	45,90	6,17
	C2 - Calculated	12,84	46,25	6,47
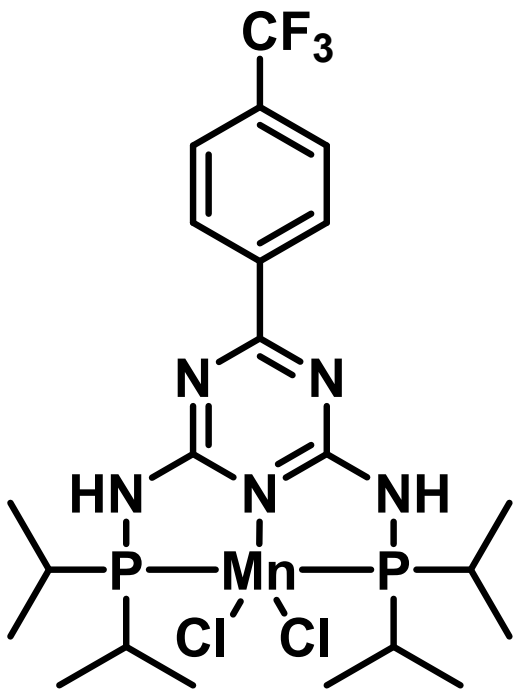	C3 (run 1)	11,24	43,16	5,32
	C3 (run 2)	11,29	43,24	5,36
	C3 - Calculated	11,42	43,08	5,59
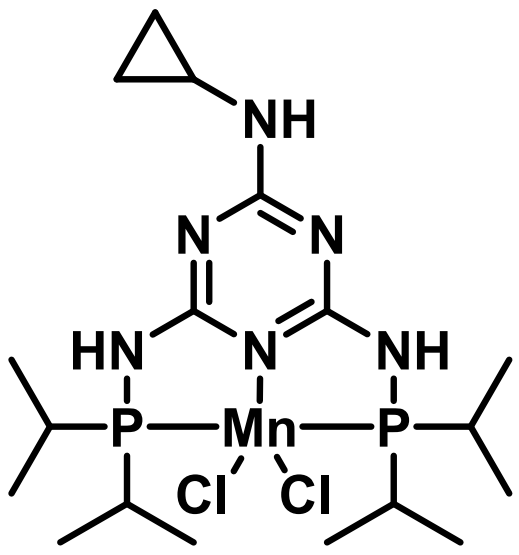	C4 (run 1)	14,25	42,75	6,88
	C4 (run 2)	14,07	42,84	6,88
	C4 - Calculated	16,03	41,23	6,92
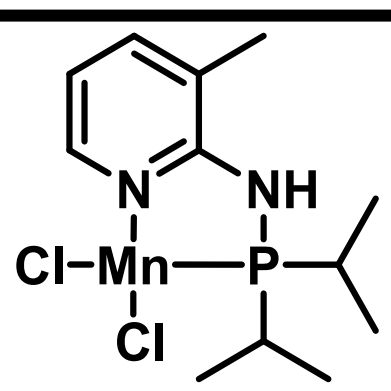	C5 (run 1)	7,61	40,81	5,93
	C5 (run 2)	7,86	40,98	5,94
	C5 - Calculated	8,00	41,17	6,05
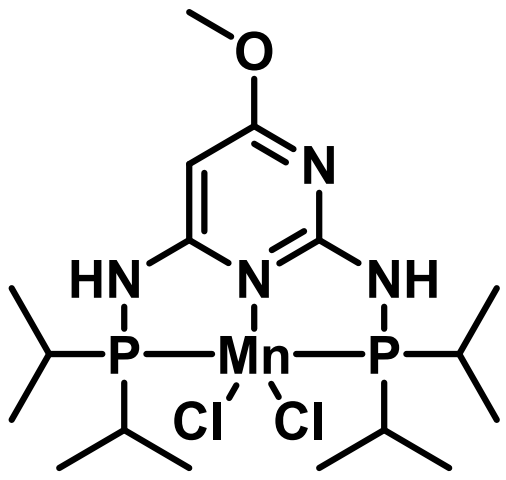	C6 (run 1)	8,72	42,38	6,82
	C6 (run 2)	8,94	43,02	6,94
	C6 - Calculated	11,24	40,98	6,88

Structure	Material	N %	C %	H %
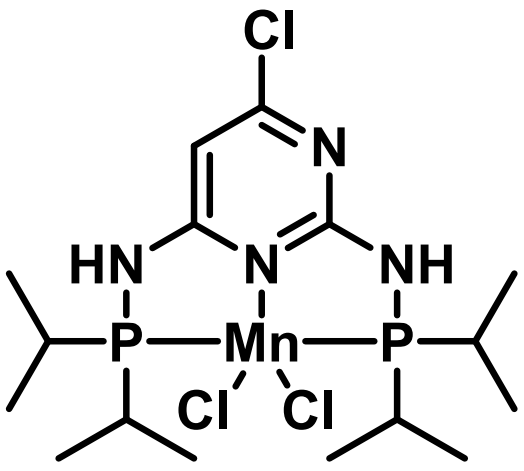	C7 (run 1)	11,40	38,09	5,90
	C7 (run 2)	11,36	38,18	5,99
	C7 - Calculated	11,15	38,23	6,22
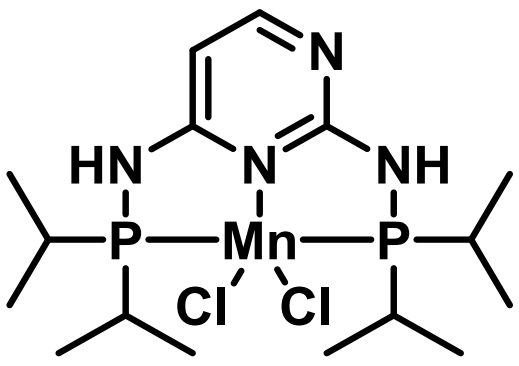	C8 (run 1)	11,86	40,95	6,67
	C8 (run 2)	11,80	40,95	6,66
	C8 - Calculated	11,97	41,04	6,89
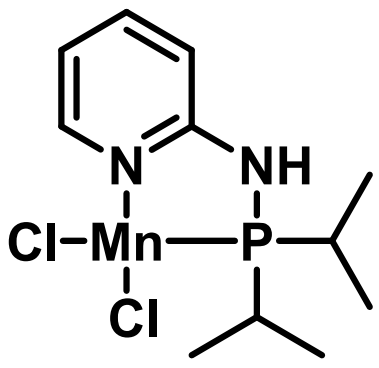	C9 (run 1)	8,56	39,35	5,51
	C9 (run 2)	8,43	38,99	5,47
	C9 - Calculated	8,34	39,31	5,70
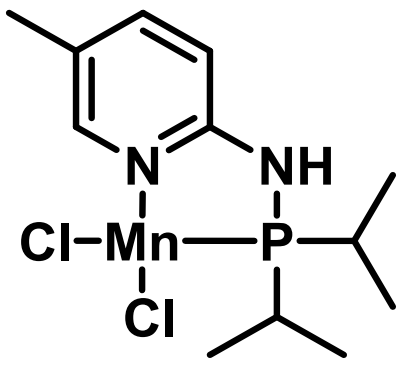	C10 (run 1)	8,08	41,34	5,76
	C10 (run 2)	8,00	41,76	5,82
	C10 - Calculated	8,00	41,17	6,05
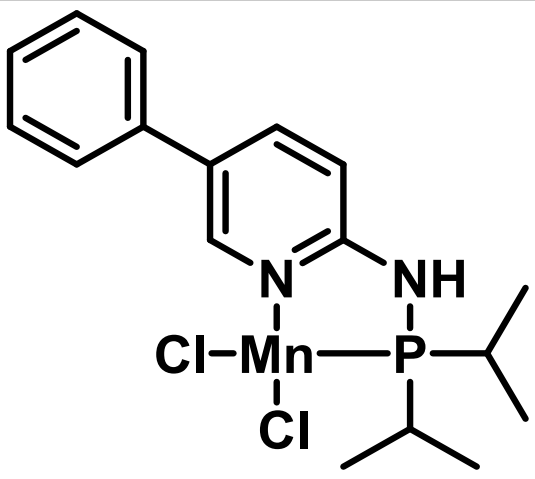	C11 (run 1)	6,46	50,08	5,57
	C11 (run 2)	6,47	49,73	5,47
	C11 - Calculated	6,80	49,54	5,62
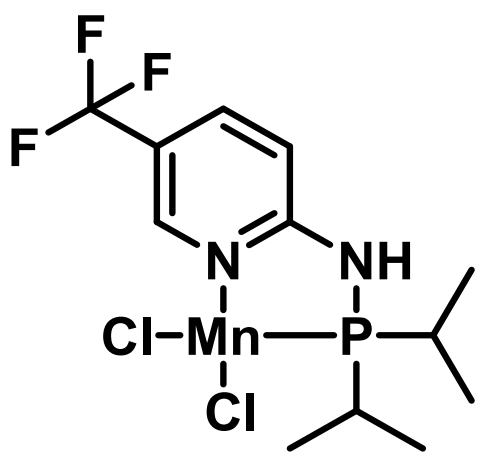	C12 (run 1)	6,03	35,19	4,54
	C12 (run 2)	6,40	34,94	4,54
	C12 - Calculated	6,93	35,67	4,49

Structure	Material	**N %**	**C %**	**H %**
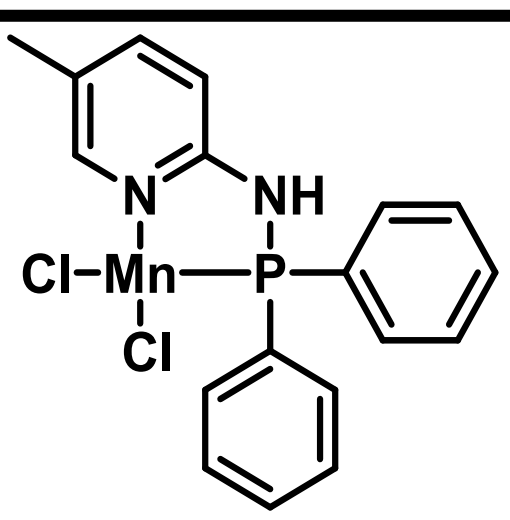	C13 (run 1)	6,18	51,54	4,15
	C13 (run 2)	6,33	51,71	4,14
	C13 - Calculated	6,70	51,70	4,10
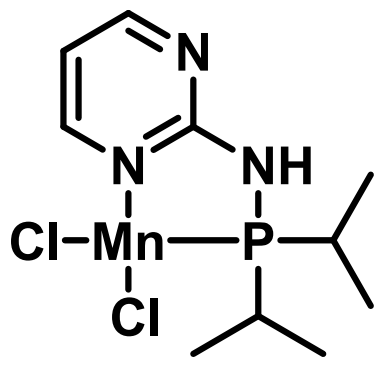	C14 (run 1)	12,60	35,78	5,30
	C14 (run 2)	12,56	35,72	5,22
	C14 - Calculated	12,47	35,63	5,38
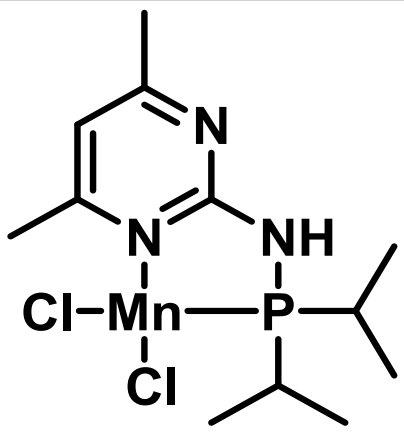	C15 (run 1)	11,23	39,15	5,88
	C15 (run 2)	11,60	39,84	5,94
	C15 - Calculated	11,51	39,47	6,07
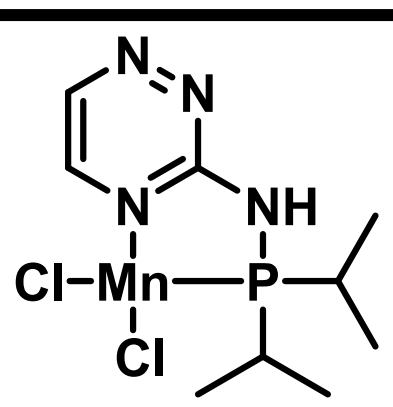	C16 (run 1)	16,20	32,22	5,25
	C16 (run 2)	16,04	32,00	5,05
	C16 - Calculated	16,57	31,97	5,07
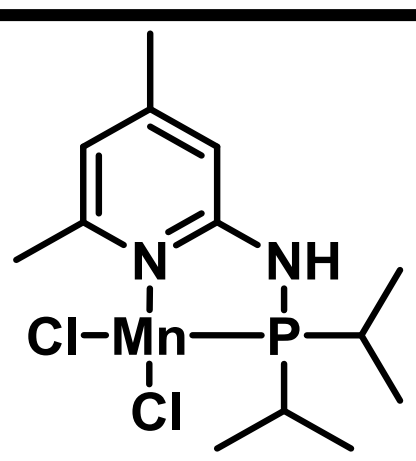	C17 (run 1)	7,87	42,42	6,28
	C17 (run 2)	7,94	42,75	6,31
	C17 - Calculated	7,69	42,88	6,37
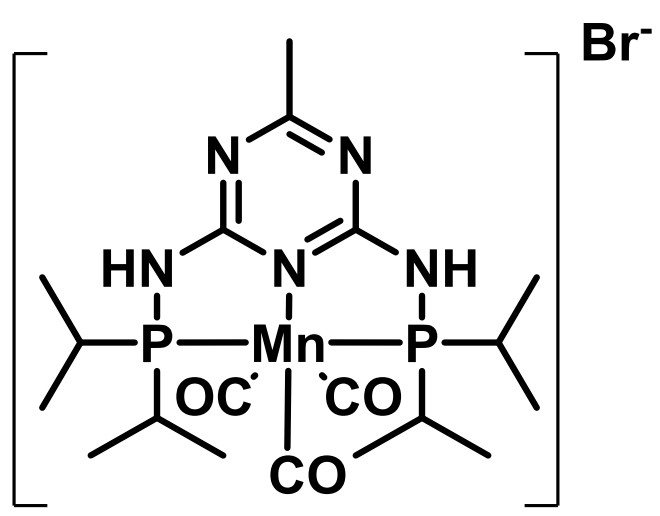	C18 (run 1)	11,98	39,49	5,71
	C18 (run 2)	11,96	39,11	5,76
	C18 - Calculated	12,15	39,60	5,77

Structure	Material	N %	C %	H %
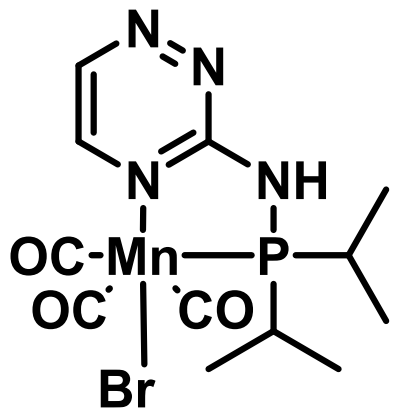	C19 (run 1)	12,89	33,05	4,09
	C19 (run 2)	12,74	33,05	4,03
	C19 - Calculated	13,00	33,43	3,97
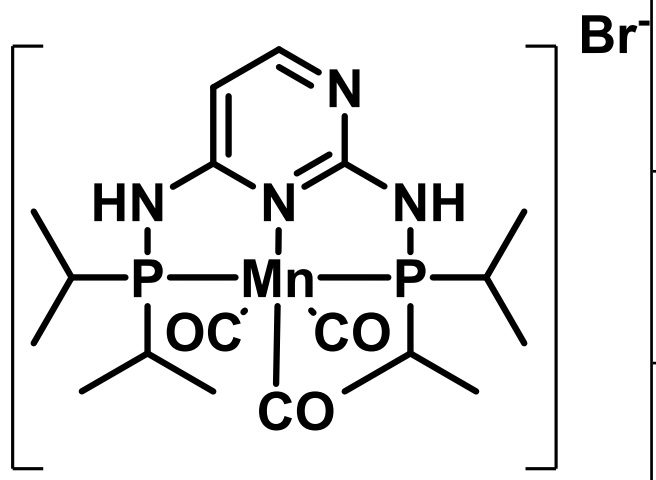	C20 (run 1)	9,54	47,65	6,27
	C20 (run 2)	9,48	47,48	6,08
	C20 - Calculated	9,98	40,66	5,75
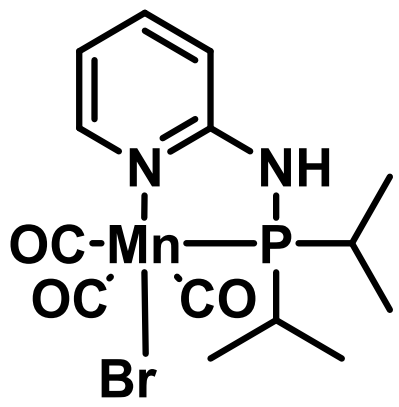	C21 (run 1)	6,33	40,23	4,33
	C21 (run 2)	6,46	40,55	4,29
	C21 - Calculated	6,53	39,18	4,46
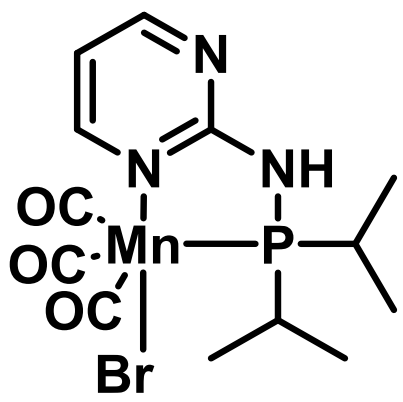	C22 (run 1)	9,34	36,65	4,14
	C22 (run 2)	9,49	36,50	4,05
	C22 - Calculated	9,77	36,30	4,22

$Mn(CO)_3Br(L1)$ (C18)

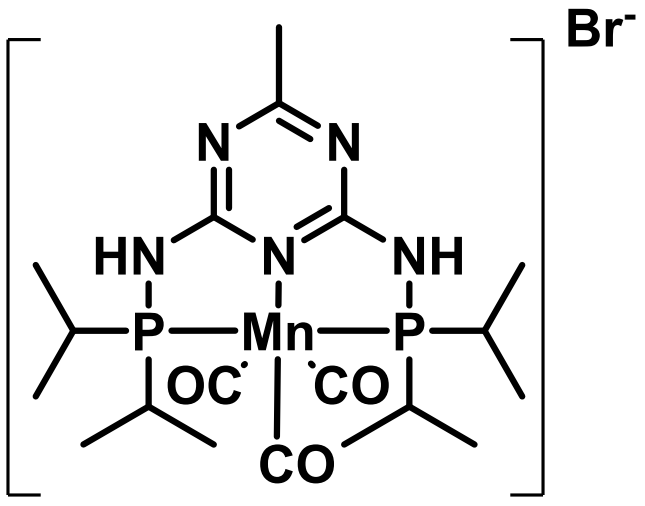

$Mn(CO)_3Br(L9)$ (**C18)** was obtained as yellow powder in 99% yield. The title compound was previously unknown in the literature.

^{1}H NMR (400 MHz, THF): δ 2.20 (s, 3H), 2.14 (s, 2H), 1.34 – 1.16 (m, 28H).
^{13}C NMR (101 MHz, THF): δ 137.45, 128.69, 127.92, 125.06, 29.65, 27.98, 27.87, 27.77, 26.89, 26.78, 26.68, 20.50, 18.65, 18.62, 18.58, 17.88, 17.80, 17.52, 16.68.
^{31}P NMR (101 MHz, THF): δ 134.8, 134.5.

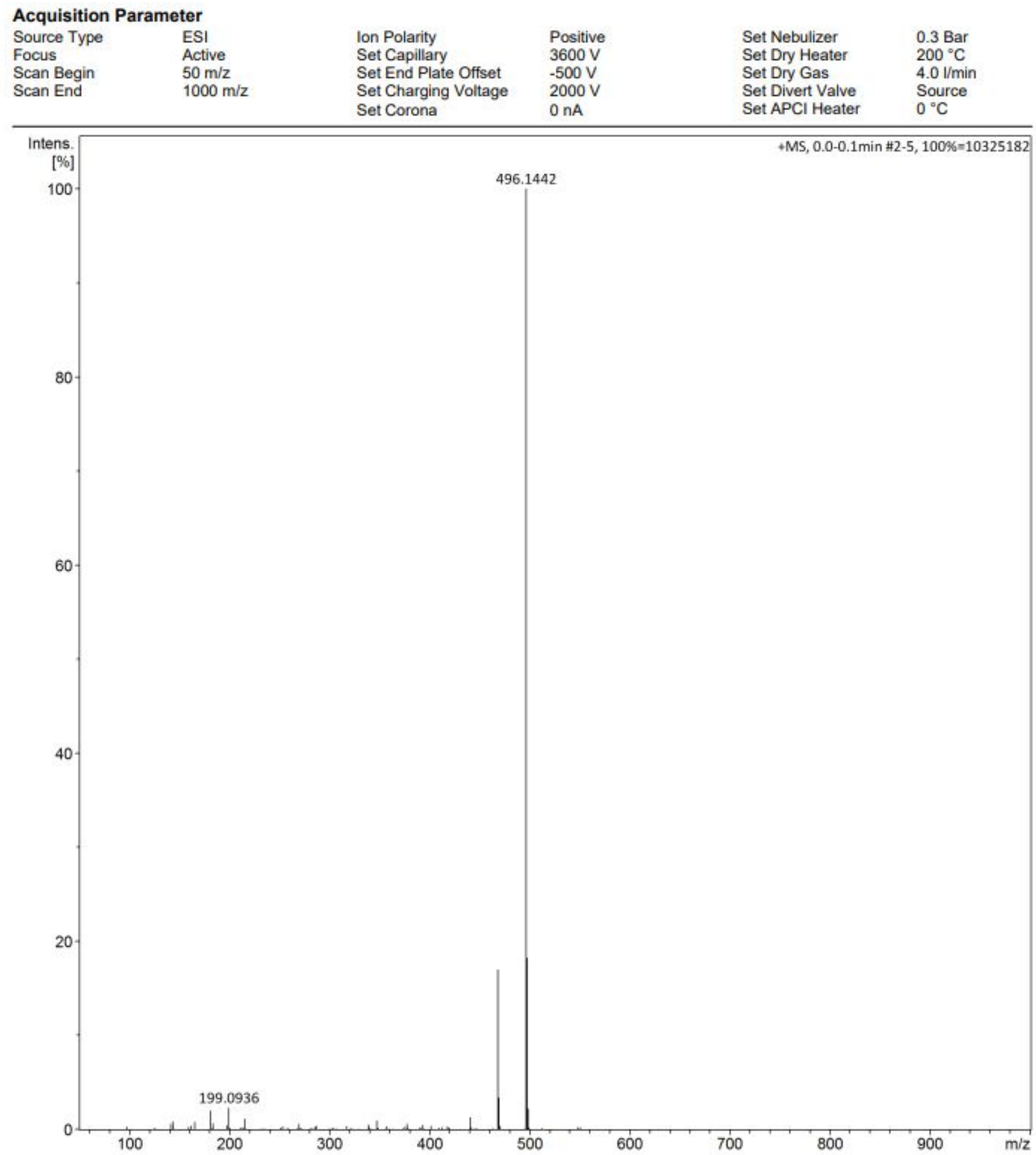

Figure S41. HRMS analysis of $Mn(CO)_3Br(L9)$ (**C18).**

$Mn(CO)_3Br(L16)$ (C19)

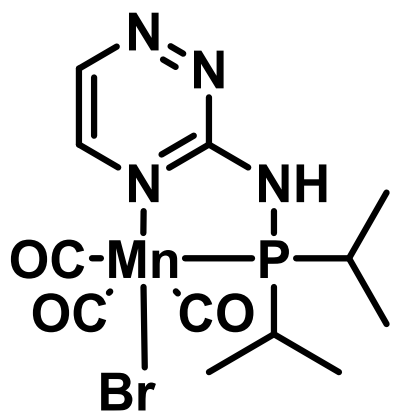

$Mn(CO)_3Br(L16)$ (**C19)** was obtained as dark-brown crystalline powder in 99% yield. The title compound was previously unknown in the literature.

^{1}H NMR (400 MHz, THF): δ 8.64 – 8.29 (m, 1H), 7.05 (d, J = 21.8 Hz, 1H), 2.20 (s, 1H), 1.46 – 1.01 (m, 14H).
^{13}C NMR (101 MHz, THF): δ 137.44, 128.73, 127.98, 124.69, 27.72, 27.47, 26.93, 26.70, 20.62, 18.41, 18.17, 17.47, 16.62.
^{31}P NMR (101 MHz, THF): δ 123.6.

Mn(CO)3Br(L9) (C21)

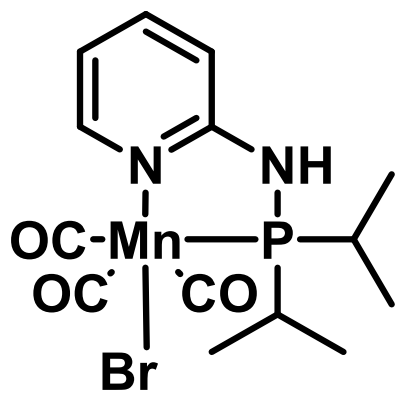

$Mn(CO)_3Br(L9)$ (**C21)** was obtained as orange powder in 99% yield and recrystallized from THF yielding orange crystals. The title compound was known in the literature and all spectroscopic data are in agreement.[31]

^{1}H NMR (400 MHz, THF): δ 8.41 (d, J = 5.4 Hz, 1H), 7.40 (d, J = 7.1 Hz, 2H), 6.70 (s, 1H), 6.58 (t, J = 5.9 Hz, 1H), 3.36 – 3.24 (m, 1H), 2.68 – 2.54 (m, 1H), 1.35 – 1.10 (m, 12H).
^{13}C NMR (101 MHz, THF): δ 162.25, 162.12, 153.32, 153.28, 138.80, 115.32, 110.47, 110.40, 28.10, 27.86, 27.05, 26.83, 18.46, 18.38, 18.28, 18.21, 17.59, 16.30, 16.26.
^{31}P NMR (101 MHz, THF): δ 125.5.

$Mn(CO)_3Br(L14)$ (C22)

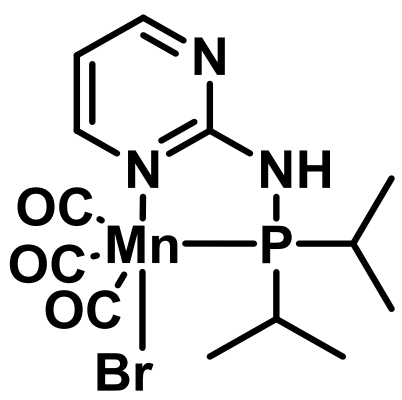

$Mn(CO)_3Br(L14)$ (**C22)** was obtained as orange powder in 99% yield and recrystallized from THF yielding orange crystals. The title compound was previously unknown in the literature.

^{1}H NMR (400 MHz, THF): δ 8.68 (s, 1H), 8.29 (s, 1H), 6.70 (d, *J* = 5.4 Hz, 1H), 2.72 – 2.59 (m, 1H), 1.41 – 1.10 (m, 14H).
^{13}C NMR (101 MHz, THF): δ 162.94, 159.79, 113.19, 28.05, 27.81, 26.82, 26.59, 18.33, 18.25, 18.21, 18.15, 17.46, 16.36, 16.32.
^{31}P NMR (101 MHz, THF): δ 117.3.

1,1,1-triethyl-3-phenyldisiloxan (1)

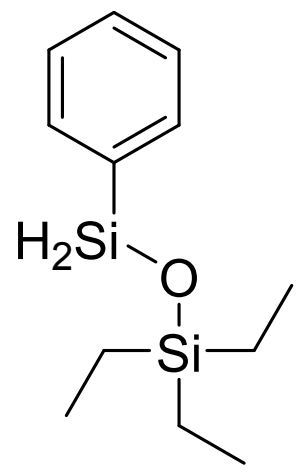

((3-ethylpentan-3-yl)oxy)(phenyl)silane (**1**) was obtained as colorless oil in 74% isolated yield. The title compound was known in the literature and all spectroscopic data are in agreement.[32]

^{1}H NMR (400 MHz, $CDCl_3$): δ 7.57 – 7.53 (m, 2H), 7.36 – 7.27 (m, 3H), 5.04 (s, 2H), 0.92 – 0.81 (m, 9H), 0.55 – 0.46 (m, 6H).
^{13}C NMR (101 MHz, $CDCl_3$): δ 135.23, 133.90, 130.15, 127.98, 6.63, 5.93.
EI-MS m/z (rel. int.): 209 (100%), 153 (42), 107 (22), 78 (35), 74 (10).

1-(tert-butyl)-1,1-dimethyl-3-phenyldisiloxane (2)

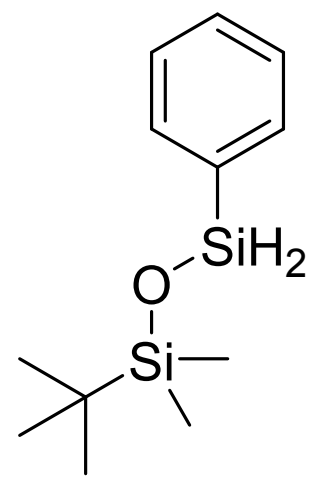

1-(tert-butyl)-1,1-dimethyl-3-phenyldisiloxane (**2**) was obtained as colorless oil in 65% isolated yield. The title compound was known in the literature and all spectroscopic data are in agreement.[19]

^{1}H NMR (400 MHz, $CDCl_3$): δ 7.57 – 7.53 (m, 2H), 7.39 – 7.29 (m, 3H), 5.03 (s, 2H), 0.82 (s, 9H), -0.00 (s, 6H).
^{13}C NMR (101 MHz, $CDCl_3$): δ 135.23, 133.90, 130.15, 127.98, 6.63, 5.93.
EI-MS m/z (rel. int.): 181 (100%), 179 (21), 165 (8), 121 (8), 78 (10).

1,1,1-triisopropyl-3-phenyldisiloxane (3)

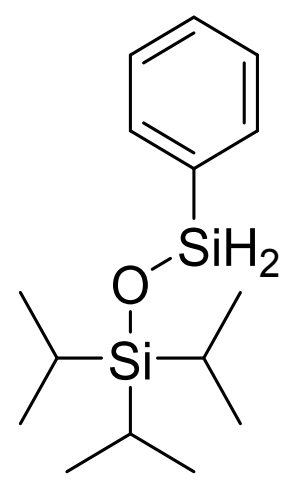

1,1,1-triisopropyl-3-phenyldisiloxane (**3**) was obtained as colorless oil in 68% isolated yield. The title compound was known in the literature and all spectroscopic data are in agreement.[19]

^{1}H NMR (400 MHz, $CDCl_3$): δ 7.64 – 7.58 (m, 2H), 7.41 – 7.34 (m, 3H), 5.16 (s, 2H), 1.00 (d, J = 3.0 Hz, 21H).

13C NMR (101 MHz, $CDCl_3$): δ 135.32, 133.93, 130.12, 127.98, 17.77, 12.64.
EI-MS m/z (rel. int.): 237 (100%), 209 (28), 181 (33), 167 (36), 77 (30).

1,1-dimethyl-1,3-diphenyldisiloxane (4)

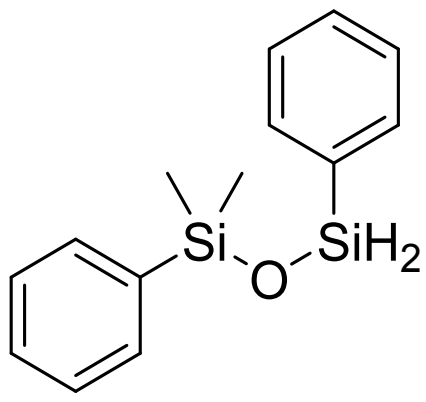

1,1-dimethyl-1,3-diphenyldisiloxane (**4**) was obtained as colorless oil in 78% isolated yield. The title compound was known in the literature and all spectroscopic data are in agreement.[33]

1H NMR (400 MHz, $CDCl_3$): δ 7.54 – 7.49 (m, 2H), 7.49 – 7.45 (m, 2H), 7.37 – 7.26 (m, 5H), 5.05 (s, 2H), 0.29 (s, 6H).
13C NMR (101 MHz, $CDCl_3$): δ 138.60, 134.41, 133.81, 132.79, 130.04, 129.30, 127.80, 127.60, 0.00.
EI-MS m/z (rel. int.): 393 (70%), 330 (53), 315 (84), 252 (53), 197 (100).

1,1,1-trimethyl-3,3-diphenyldisiloxane (5)

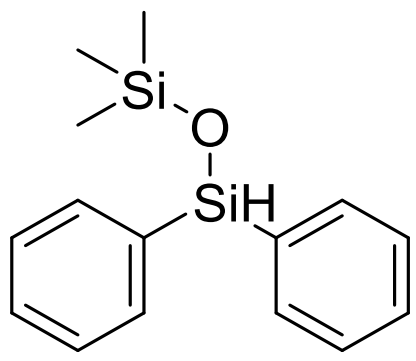

1,1,1-trimethyl-3,3-diphenyldisiloxane (**5**) was obtained as colorless oil in 69% isolated yield. The title compound was known in the literature and all spectroscopic data are in agreement.[20]

1H NMR (400 MHz, $CDCl_3$): δ 7.49 – 7.44 (m, 4H), 7.30 – 7.22 (m,6H), 5.36 (s, 1H), -0.00 (s, 9H).
13C NMR (101 MHz, $CDCl_3$) δ 134.33, 134.00, 132.48, 128.31, 126.45, 126.21, -0.00.
EI-MS m/z (rel. int.): 179 (100%), 135 (26), 105 (11), 78 (46), 51 (10).

1-(tert-butyl)-1,1-dimethyl-3,3-diphenyldisiloxane (6)

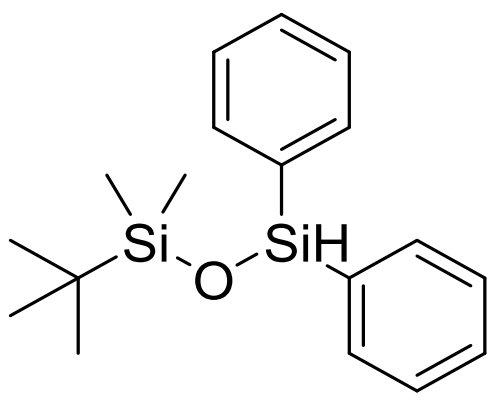

1-(tert-butyl)-1,1-dimethyl-3,3-diphenyldisiloxane (**6**) was obtained as colorless oil in 86% isolated yield. The title compound was known in the literature and all spectroscopic data are in agreement.[20]

1H NMR (400 MHz, $CDCl_3$): δ 7.53 (dd, *J* = 7.7, 1.8 Hz, 4H), 7.36 – 7.26 (m, 6H), 5.46 (s, `H), 0.82 (s, 9H), -0.00 (s, 6H).
13C NMR (101 MHz, $CDCl_3$) δ 136.19, 134.27, 130.08, 127.97, 25.79, 18.42, -3.05.
EI-MS m/z (rel. int.): 257 (100%), 179 (67), 135 (18), 78 (27), 57 (20).

1-(tert-butyl)-1,1,3-trimethyl-3-phenyldisiloxane (7)

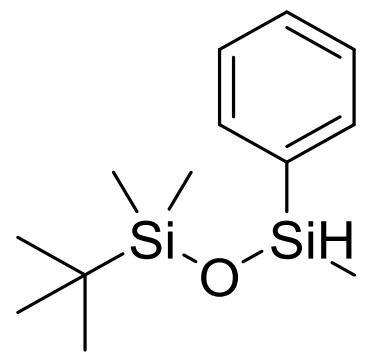

1-(tert-butyl)-1,1,3-trimethyl-3-phenyldisiloxane (**7**) was obtained as colorless oil in 79% isolated yield. The title compound was known in the literature and all spectroscopic data are in agreement.[18]

^{1}H NMR (400 MHz, $CDCl_3$): δ 7.54 – 7.50 (m, 2H), 7.36 – 7.29 (m, 3H), 5.06 (dd, *J* = 2.8, 1.6 Hz, 1H), 0.83 (d, *J* = 1.4 Hz, 9H), 0.36 (dd, *J* = 2.9, 1.3 Hz, 3H), -0.00 (s, 6H).
^{13}C NMR (101 MHz, $CDCl_3$) δ 138.19, 133.50, 129.86, 128.01, 25.85, 18.39, -0.00, -2.97, -3.02.
EI-MS m/z (rel. int.): 195 (100%), 179 (18), 135 (13), 78 (24), 27 (16).

1-(tert-butyl)-3,3-bis(4-fluorophenyl)-1,1-dimethyldisiloxane (8)

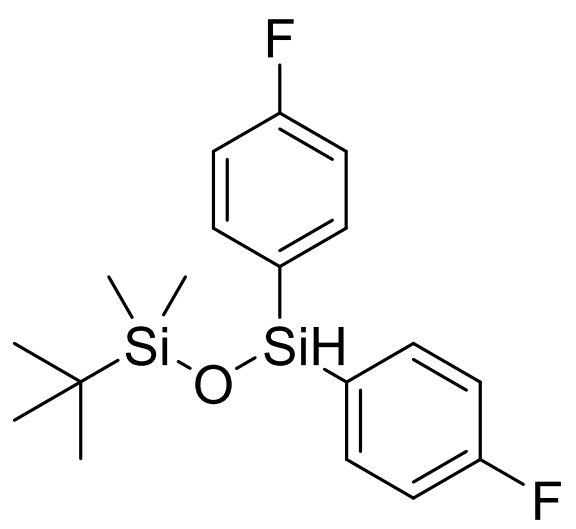

1-(tert-butyl)-3,3-bis(4-fluorophenyl)-1,1-dimethyldisiloxane (**8**) was obtained as colorless oil in 98% isolated yield. Title compound was previously unknown in literature.

^{1}H NMR (400 MHz, $CDCl_3$): δ 7.49 (dd, *J* = 8.5, 6.2 Hz, 4H), 7.12 – 6.91 (m, 4H), 5.44 (s, 1H), 0.82 (s, 9H), 0.00 (s, 6H).
^{13}C NMR (101 MHz, $CDCl_3$) δ 165.63, 163.15, 136.29, 136.21, 131.54, 131.50, 115.37, 115.17, 25.66, 18.30, -3.15.
EI-MS m/z (rel. int.): 293 (100%), 213 (73), 197 (28), 165 (45), 124 (24).

1-(tert-butyl)-3-hexyl-1,1-dimethyldisiloxanez (9)

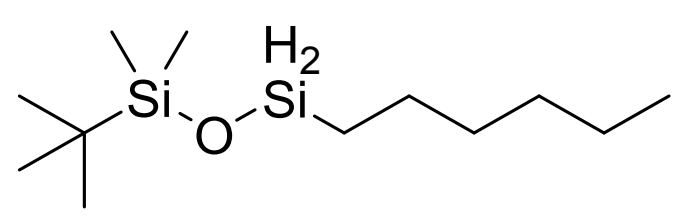

1-(tert-butyl)-3-hexyl-1,1-dimethyldisiloxanez (**9**) was obtained as colorless oil in 71% isolated yield. Title compound was previously unknown in literature.

^{1}H NMR (400 MHz, $CDCl_3$): δ 4.60 – 4.53 (m, 2H), 2.42 – 2.26 (m, 3H), 1.27 – 1.20 (m, 10H), 0.93 – 0.79 (m, 9H), 0.07 – -0.01 (m, 6H).
.^{13}C NMR (101 MHz, $CDCl_3$) δ 32.37, 31.58, 25.66, 25.62, 22.55, 16.25, 14.93, 14.10, -3.57, -3.61.
EI-MS m/z (rel. int.): 189 (58%), 105 (100), 103 (14), 88 (6), 57 (15).

1,1,1,5,5,5-hexaisobutyl-3-phenyltrisiloxane (10)

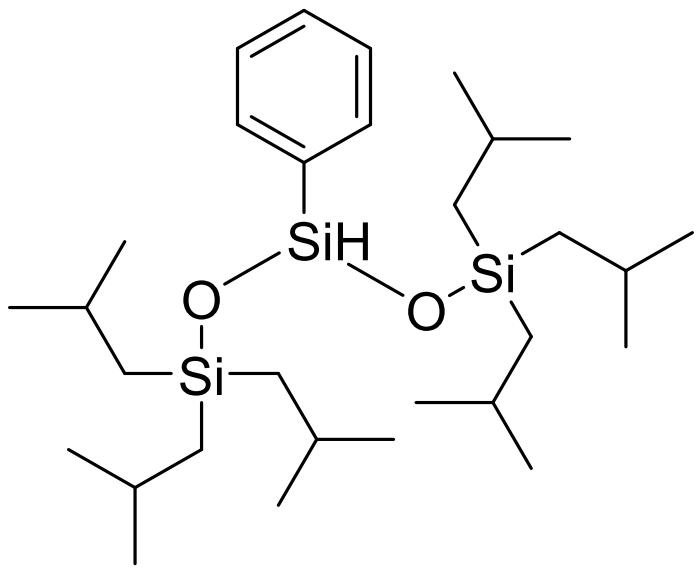

1,1,1,5,5,5-hexaisobutyl-3-phenyltrisiloxane (**10**) was obtained as colorless oil in 88% isolated yield. The title compound was known in the literature and all spectroscopic data are in agreement.[20]

^{1}H NMR (400 MHz, $CDCl_3$): δ 7.51 – 7.45 (m, 2H), 7.35 – 7.23 (m, 3H), 4.96 (s, 1H), 1.73 (dp, *J* = 13.3, 6.6 Hz, 6H), 0.83 (d, *J* = 6.7 Hz, 36H), 0.52 (d, *J* = 6.9 Hz, 12H).
^{13}C NMR (101 MHz, $CDCl_3$): δ 137.45, 133.08, 129.79, 127.56, 27.41, 26.44, 24.19.
EI-MS m/z (rel. int.): 479 (66%), 423 (56), 367 (54), 199 (100), 113 (25).

1,1,5,5-tetramethyl-1,3,5-triphenyltrisiloxane (11)

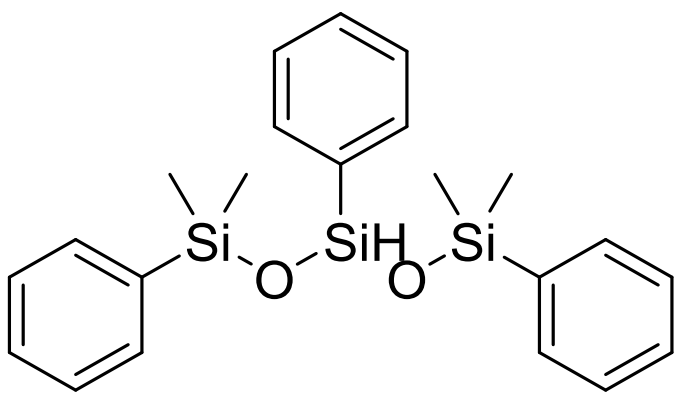

1,1,5,5-tetramethyl-1,3,5-triphenyltrisiloxane (**11**) was obtained as colorless oil in 87% isolated yield. The title compound was known in the literature and all spectroscopic data are in agreement.[18]

^{1}H NMR (400 MHz, $CDCl_3$): δ 7.54 – 7.40 (m, 6H), 7.36 – 7.18 (m, 9H), 5.03 (d, *J* = 3.1 Hz, 1H), 0.26 (s, 12H).
^{13}C NMR (101 MHz, $CDCl_3$): 138.44, 135.84, 132.60, 132.47, 129.58, 128.82, 127.23, 127.16, -0.00, -0.02.
EI-MS m/z (rel. int.): 243 (46%), 180 (39), 165 (100), 121 (35), 78 (61).

1,1,1,5,5,5-hexamethyl-3-phenyltrisiloxane (12)

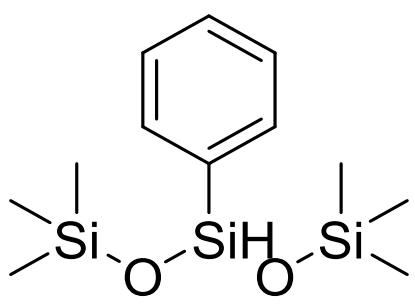

1,1,1,5,5,5-hexamethyl-3-phenyltrisiloxane (**12**) was obtained as colorless oil in 81% isolated yield. The title compound was known in the literature and all spectroscopic data are in agreement.[34]

^{1}H NMR (400 MHz, $CDCl_3$): δ 7.46 – 7.40 (m, 2H), 7.30 – 7.19 (m, 3H), 4.83 (s, 1H), -0.00 (s, 18H).
^{13}C NMR (101 MHz, $CDCl_3$): δ 131.39, 128.28, 126.04, 0.00.
EI-MS m/z (rel. int.): 269 (100%), 195 (17), 179 (11), 135 (83), 121 (28).

1,1,1,5,5,5-hexaethyl-3-phenyltrisiloxane (13)

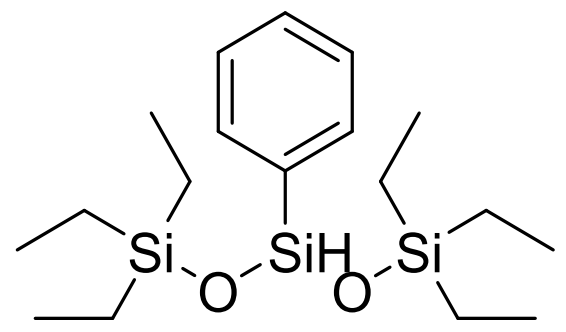

1,1,1,5,5,5-hexaethyl-3-phenyltrisiloxane (**13**) was obtained as colorless oil in 79% isolated yield. The title compound was known in the literature and all spectroscopic data are in agreement.[34]

^{1}H NMR (400 MHz, $CDCl_3$): δ 7.50 (d, *J* = 6.8 Hz, 2H), 7.30 (dd, *J* = 10.4, 6.7 Hz, 3H), 4.97 (s, 1H), 0.86 (t, *J* = 8.0 Hz, 18H), 0.50 (q, *J* = 7.9 Hz, 12H).
^{13}C NMR (101 MHz, $CDCl_3$): δ 137.47, 133.02, 129.91, 127.70, 6.67, 6.13.
EI-MS m/z (rel. int.): 339 (100%), 311 (10), 135 (17), 107 (27), 87 (7).

1,5-di-tert-butyl-1,1,5,5-tetramethyl-3-phenyltrisiloxane (14)

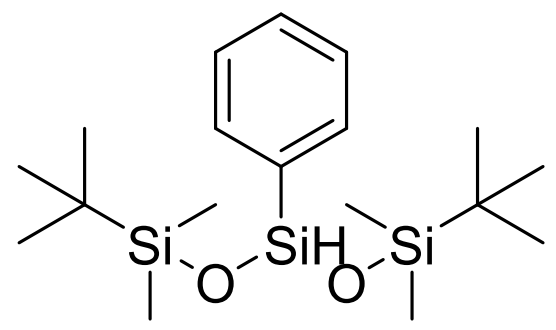

1,5-di-tert-butyl-1,1,5,5-tetramethyl-3-phenyltrisiloxane (**14**) was obtained as colorless oil in 78% isolated yield. The title compound was known in the literature and all spectroscopic data are in agreement.[20]

^{1}H NMR (400 MHz, $CDCl_3$): δ 7.50 (dt, *J* = 6.3, 1.7 Hz, 2H), 7.35 – 7.25 (m, 3H), 4.95 (s, 1H), 0.81 (s, 18H), -0.00 (s, 6H), -0.02 (s, 6H).
^{13}C NMR (101 MHz, $CDCl_3$): δ 137.18, 133.09, 129.97, 127.74, 25.66, 18.17, -3.05.
EI-MS m/z (rel. int.): 132 (87%), 105 (100), 78 (45), 53 (18), 49 (11).

1,1,1,5,5,5-hexaisopropyl-3-phenyltrisiloxane (15)

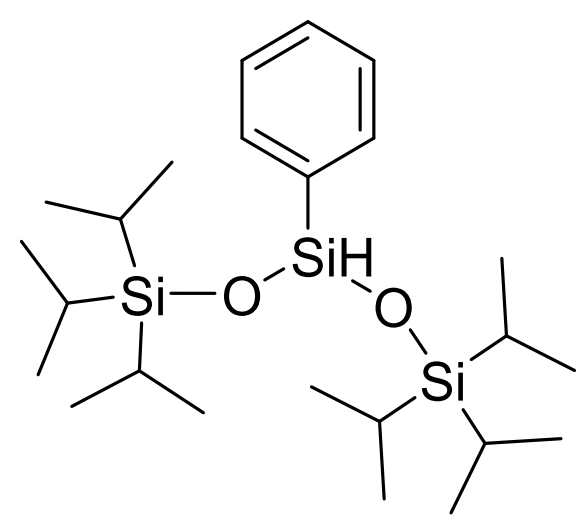

1,1,1,5,5,5-hexaisopropyl-3-phenyltrisiloxane (**15**) was obtained as colorless oil in 88% isolated yield. The title compound was known in the literature and all spectroscopic data are in agreement.[35]

^{1}H NMR (400 MHz, $CDCl_3$): δ 7.55 – 7.49 (m, 2H), 7.34 – 7.24 (m, 3H), 5.10 (s, 1H), 0.92 (s, 42H).
^{13}C NMR (101 MHz, $CDCl_3$): δ 137.67, 132.94, 129.86, 127.61, 17.78, 17.76, 12.75.
EI-MS m/z (rel. int.): 409 (100%), 367 (11), 135 (13), 127 (29), 78 (17).

3-phenyl-1,1,1,5,5,5-hexapropyltrisiloxane (16)

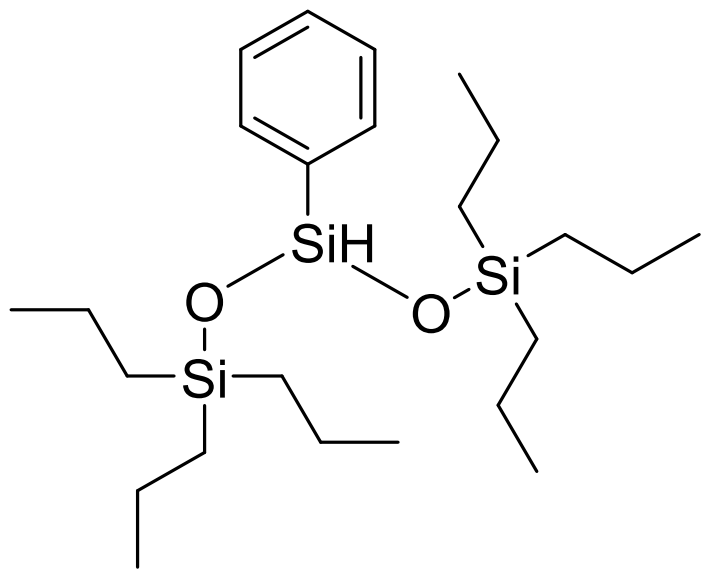

3-phenyl-1,1,1,5,5,5-hexapropyltrisiloxane (**16**) was obtained as colorless oil in 67% isolated yield. The title compound was known in the literature and all spectroscopic data are in agreement.[20]

^{1}H NMR (400 MHz, $CDCl_3$): δ 7.50 – 7.46 (m, 2H), 7.34 – 7.26 (m, 3H), 4.92 (s, 1H), 1.34 – 1.21 (m, 12H), 0.85 (t, *J* = 7.3 Hz, 18H), 0.53 – 0.45 (m, 12H).
^{13}C NMR (101 MHz, $CDCl_3$) δ 137.47, 133.01, 129.86, 127.66, 18.32, 18.20, 16.68.
EI-MS m/z (rel. int.): 409 (100%), 367 (18), 325 (17), 198 (21), 78 (14),

1,1,1,5,5,5-hexahexyl-3-phenyltrisiloxane (17)

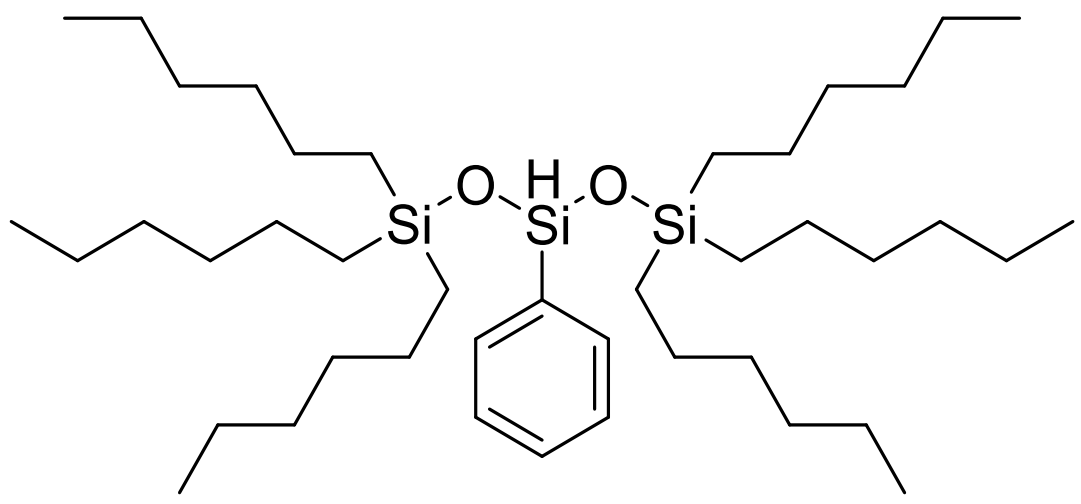

1,1,1,5,5,5-hexahexyl-3-phenyltrisiloxane (**17**) was obtained as colorless oil in 78% isolated yield. Title compound was previously unknown in literature.

^{1}H NMR (400 MHz, $CDCl_3$): δ 7.50 – 7.45 (m, 2H), 7.35 – 7.24 (m, 3H), 4.92 (s, 1H), 1.27 – 1.14 (m, 48H), 0.86 – 0.75 (m, 18H), 0.53 – 0.42 (m, 12H)
^{13}C NMR (101 MHz, $CDCl_3$) δ 137.52, 133.05, 129.83, 127.62, 33.35, 31.59, 23.09, 22.64, 15.44, 14.14.
EI-MS m/z (rel. int.): 215 (100%), 131 (89), 113 (38), 100 (11), 55 (11).

1,5-di-tert-butyl-1,1,3,5,5-pentaphenyltrisiloxane (18)

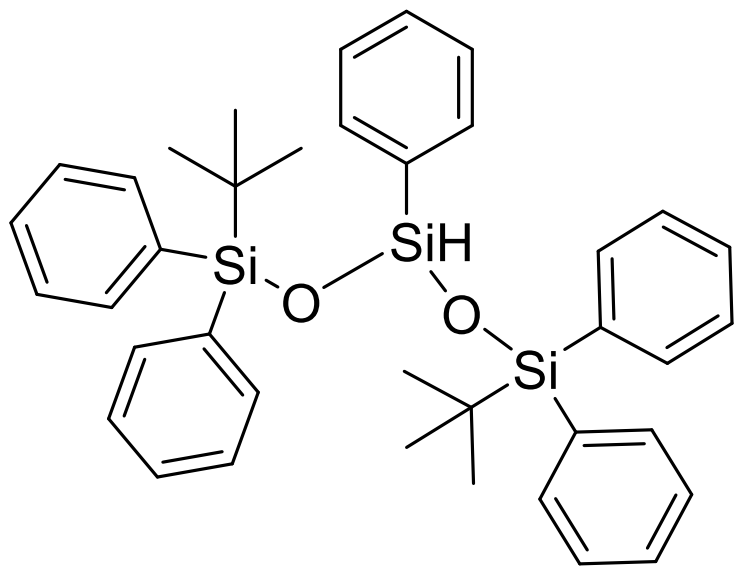

1,5-di-tert-butyl-1,1,3,5,5-pentaphenyltrisiloxane (**18**) was obtained as colorless oil in 86% isolated yield. Title compound was previously unknown in literature.

^{1}H NMR (400 MHz, $CDCl_3$): δ 7.51 (dq, *J* = 8.2, 1.6 Hz, 8H), 7.42 (dt, *J* = 8.2, 1.6 Hz, 2H), 7.28 – 7.21 (m, 5H), 7.19 – 7.10 (m, 10H), 5.38 (s, 1H), 0.89 (d, *J* = 2.0 Hz, 18H).
^{13}C NMR (101 MHz, $CDCl_3$) δ 135.94, 135.05, 134.97, 134.92, 133.31, 130.20, 129.41, 127.75, 127.52, 26.61, 19.36.
^{29}Si NMR (80 MHz, $CDCl_3$) δ -9.92, -47.51.
EI-MS m/z (rel. int.): 305 (100%), 227 (34), 181 (10), 78 (19), 57 (8).

1,5-diethyl-1,1,5,5-tetraisopropyl-3-phenyltrisiloxane (19)

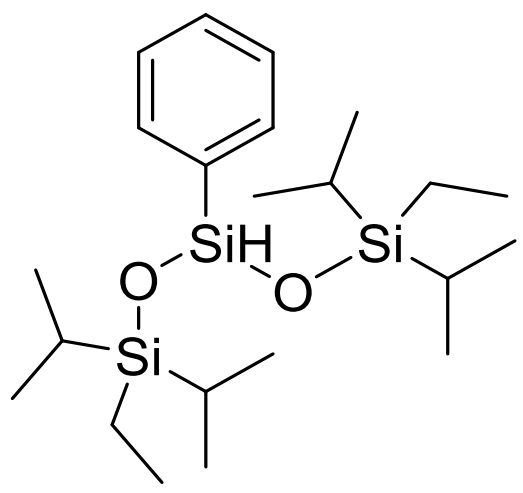

1,5-diethyl-1,1,5,5-tetraisopropyl-3-phenyltrisiloxane (**19**) was obtained as colorless oil in 62% isolated yield. The title compound was known in the literature and all spectroscopic data are in agreement.[20]

^{1}H NMR (400 MHz, $CDCl_3$): δ 7.57 – 7.45 (m, 2H), 7.32 – 7.25 (m, 3H), 5.05 (s, 1H), 0.95 – 0.84 (m, 34H), 0.52 (q, *J* = 8.0 Hz, 4H).
^{13}C NMR (101 MHz, $CDCl_3$): δ 137.59, 132.97, 129.87, 127.64, 17.46 – 17.30 (m), 13.01 (d, *J* = 1.8 Hz), 7.11, 3.73.
EI-MS m/z (rel. int.): 381 (100%), 339 (7), 141 (12), 135 (12), 120 (16).

3,3-diisopropyl-1,5-diphenyltrisiloxane (20)

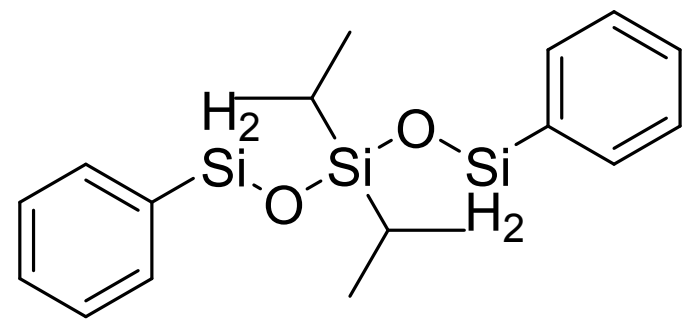

3,3-diisopropyl-1,5-diphenyltrisiloxane (**20**) was obtained as colorless oil in 79% isolated yield. The title compound was known in the literature and all spectroscopic data are in agreement.[19]

^{1}H NMR (400 MHz, $CDCl_3$): δ 7.56 – 7.50 (m, 4H), 7.35 – 7.25 (m, 6H), 5.06 (s, 4H), 0.89 (m, 14H).
^{13}C NMR (101 MHz, $CDCl_3$): δ 134.62, 134.04, 130.30, 128.04, 16.83, 13.08.
EI-MS m/z (rel. int.): 317 (100%), 239 (12), 211 (28), 197 (14), 151 (16).

1,1,1,3,5,5,5-heptaphenyltrisiloxane (21)

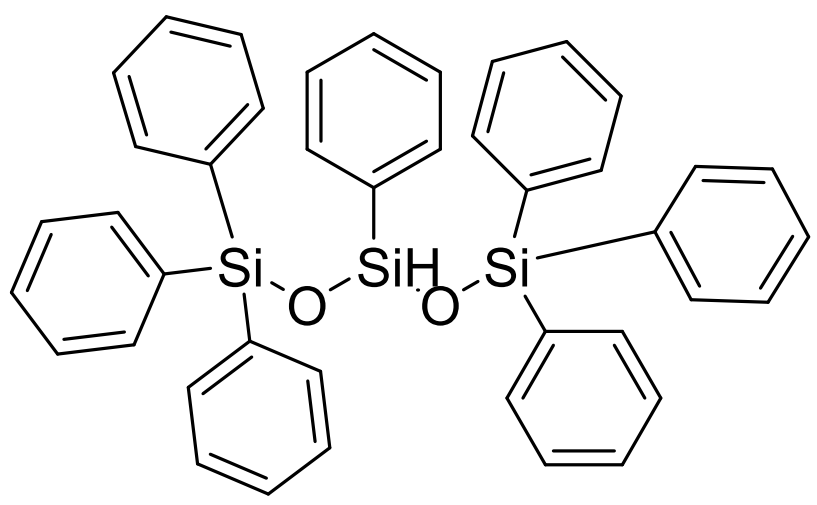

1,1,1,3,5,5,5-heptaphenyltrisiloxane (**21**) was obtained as colorless oil in 59% isolated yield. Title compound was previously unknown in literature.

^{1}H NMR (400 MHz, $CDCl_3$): δ 7.37 (dd, *J* = 7.9, 1.5 Hz, 12H), 7.31 – 7.21 (m, 9H), 7.16 – 7.06 (m, 14H), 5.30 (s, 1H).
^{13}C NMR (101 MHz, $CDCl_3$) δ 134.32, 134.04, 133.98, 132.33, 129.17, 128.80, 126.74, 126.72, 126.69, 126.67.
^{29}Si NMR (80 MHz, $CDCl_3$) δ -18.49, -46.89.
EI-MS m/z (rel. int.): 425 (28%), 348 (62), 303 (34), 243 (100), 181 (37).

1,1,1,5,5,5-hexaethyl-3-(p-tolyl)trisiloxane (22)

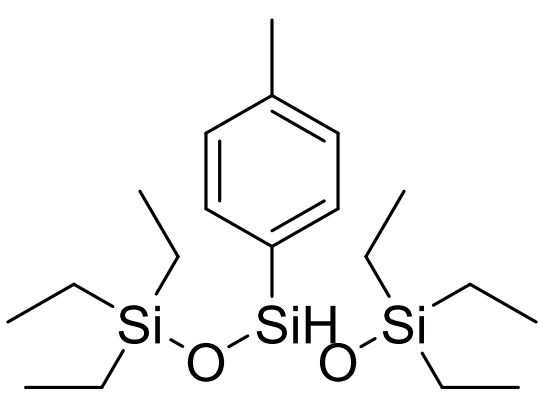

1,1,1,5,5,5-hexaethyl-3-(p-tolyl)trisiloxane (**22**) was obtained as colorless oil in 94% isolated yield. The title compound was known in the literature and all spectroscopic data are in agreement.[20]

^{1}H NMR (400 MHz, $CDCl_3$): δ 7.39 (d, *J* = 7.3 Hz, 2H), 7.10 (d, *J* = 7.6 Hz, 2H), 4.95 (s, 1H), 2.28 (s, 3H), 0.86 (t, *J* = 8.1 Hz, 18H), 0.49 (q, *J* = 8.0 Hz, 12H).
^{13}C NMR (101 MHz, $CDCl_3$) δ 139.78, 134.01, 133.10, 128.52, 21.61, 6.69, 6.15.
^{29}Si NMR (80 MHz, $CDCl_3$) δ 12.76, -49.26.
EI-MS m/z (rel. int.): 353 (100%), 351 (10), 149 (14), 121 (21), 91 (6).

1,5-di-tert-butyl-1,1,5,5-tetramethyl-3-(p-tolyl)trisiloxane (23)

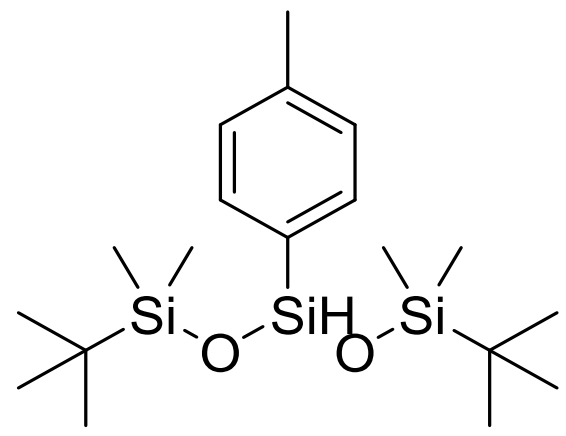

1,5-di-tert-butyl-1,1,5,5-tetramethyl-3-(p-tolyl)trisiloxane (**23**) was obtained as colorless oil in 92% isolated yield. The title compound was known in the literature and all spectroscopic data are in agreement.[19]

^{1}H NMR (400 MHz, $CDCl_3$): δ 7.40 (d, *J* = 7.8 Hz, 2H), 7.12 (d, *J* = 7.5 Hz, 2H), 4.94 (s, 1H), 2.29 (s, 3H), 0.81 (s, 18H), -0.01 (d, *J* = 5.8 Hz, 12H).
^{13}C NMR (101 MHz, $CDCl_3$) δ 139.84, 133.71, 133.15, 128.53, 25.67, 21.61, 18.17, -3.05.
EI-MS m/z (rel. int.): 325 (76%), 283 (37), 207 (24), 149 (100), 91 (28).

1,1,1,5,5,5-hexaisopropyl-3-(p-tolyl)trisiloxane (24)

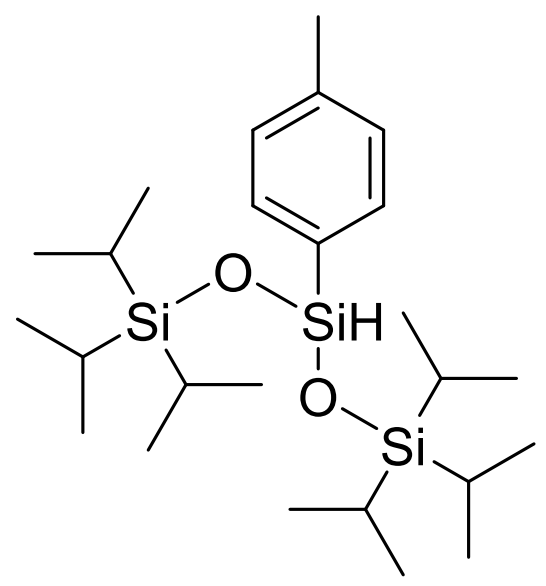

1,1,1,5,5,5-hexaisopropyl-3-(p-tolyl)trisiloxane (**24**) was obtained as colorless oil in 63% isolated yield. Title compound was previously unknown in literature.

^{1}H NMR (400 MHz, $CDCl_3$): δ 7.41 (d, *J* = 7.9 Hz, 2H), 7.09 (d, *J* = 7.6 Hz, 2H), 5.08 (s, 1H), 2.27 (s, 3H), 0.92 (s, 42H)
^{13}C NMR (101 MHz, $CDCl_3$) δ 139.63, 134.16, 133.01, 128.41, 21.64, 17.80, 17.79, 12.76.
EI-MS m/z (rel. int.): 423 (100%), 381 (13), 149 (10), 91 (31), 59 (10).

1,1,1,5,5,5-hexahexyl-3-(p-tolyl)trisiloxane (25)

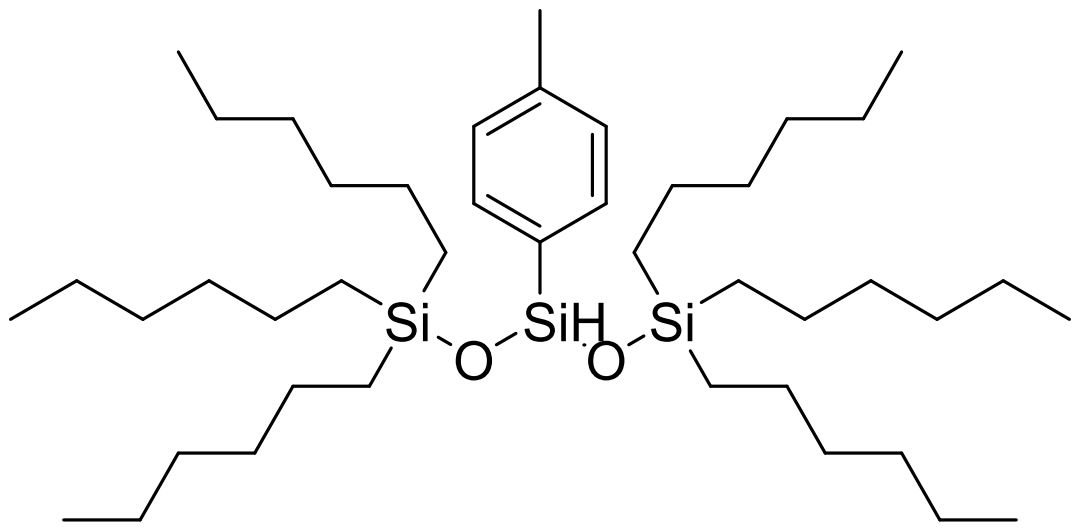

1,1,1,5,5,5-hexahexyl-3-(p-tolyl)trisiloxane (**25**) was obtained as colorless oil in 70% isolated yield. Title compound was previously unknown in literature.

^{1}H NMR (400 MHz, $CDCl_3$): δ 7.37 (d, *J* = 7.6 Hz, 2H), 7.10 (d, *J* = 7.5 Hz, 2H), 4.90 (s, 1H), 2.28 (s, 3H), 1.22 – 1.16 (m, 48H), 0.83 – 0.77 (m, 18H), 0.47 (t, *J* = 7.9 Hz, 12H).
^{13}C NMR (101 MHz, $CDCl_3$) δ 139.67, 134.07, 133.11, 128.42, 33.36, 31.59, 23.09, 22.64, 21.57, 15.45, 14.14.
^{29}Si NMR (80 MHz, $CDCl_3$) δ 9.95, -49.46.
EI-MS m/z (rel. int.): 215 (100%), 131 (83), 112 (29), 100 (10), 55 (9).

1,1,1,5,5,5-hexaisobutyl-3-(p-tolyl)trisiloxane (26)

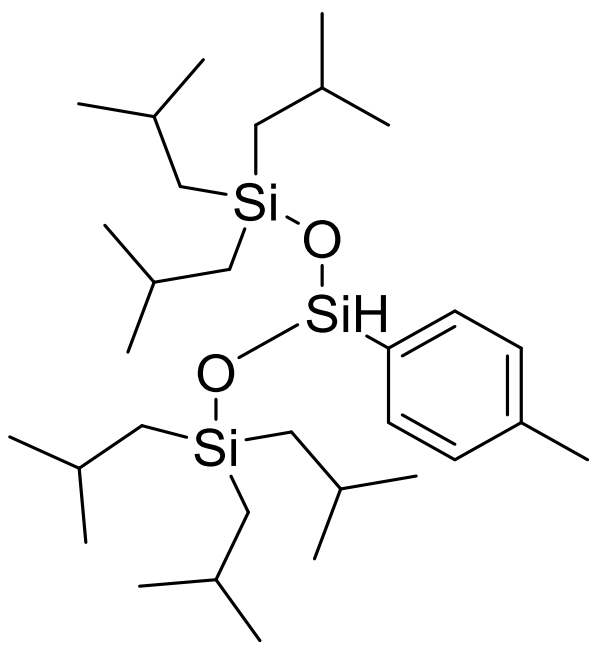

1,1,1,5,5,5-hexaisobutyl-3-(p-tolyl)trisiloxane (**26**) was obtained as colorless oil in 79% isolated yield. Title compound was previously unknown in literature.

^{1}H NMR (400 MHz, $CDCl_3$): δ 7.37 (d, *J* = 7.9 Hz, 2H), 7.09 (d, *J* = 7.5 Hz, 2H), 4.94 (s, 1H), 2.28 (s, 3H), 1.73 (m, 6H), 0.83 (d, *J* = 6.6 Hz, 36H), 0.52 (d, *J* = 6.9 Hz, 12H).
^{13}C NMR (101 MHz, $CDCl_3$) δ 139.60, 133.96, 133.16, 128.35, 27.42, 26.46, 24.19, 21.60.
EI-MS m/z (rel. int.): 493 (46%), 381 (31), 269 (35), 212 (100), 119 (21).

1,1,5,5-tetramethyl-1,5-diphenyl-3-(p-tolyl)trisiloxane (27)

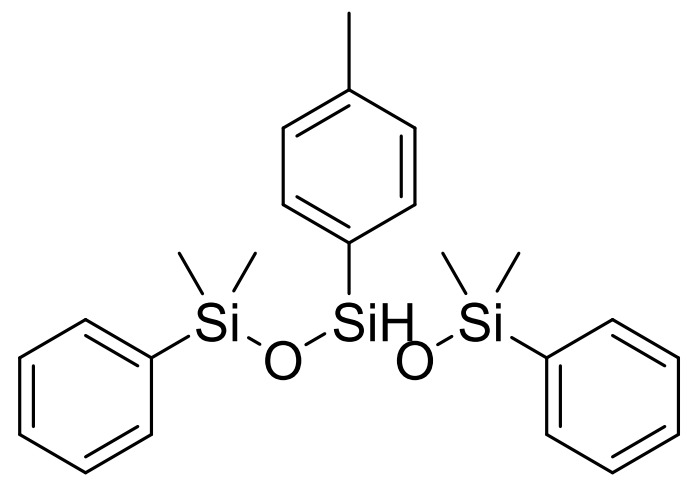

1,1,5,5-tetramethyl-1,5-diphenyl-3-(p-tolyl)trisiloxane (**27**) was obtained as colorless oil in 60% isolated yield. Title compound was previously unknown in literature.

^{1}H NMR (400 MHz, $CDCl_3$): δ 7.35 (d, *J* = 7.5 Hz, 4H), 7.25 (d, *J* = 6.1 Hz, 2H), 7.21 – 7.11 (m, 6H), 6.98 (d, *J* = 7.4 Hz, 2H), 4.91 (s, 1H), 2.17 (s, 3H), 0.15 (s, 12H).
^{13}C NMR (101 MHz, $CDCl_3$) δ 139.60, 133.96, 133.16, 128.35, 27.42, 26.46, 24.19, 21.60.
EI-MS m/z (rel. int.): 407 (27%), 329 (55), 313 (36), 197 (30), 134 (29).

3,3-diisopropyl-1,5-di-p-tolyltrisiloxane (28)

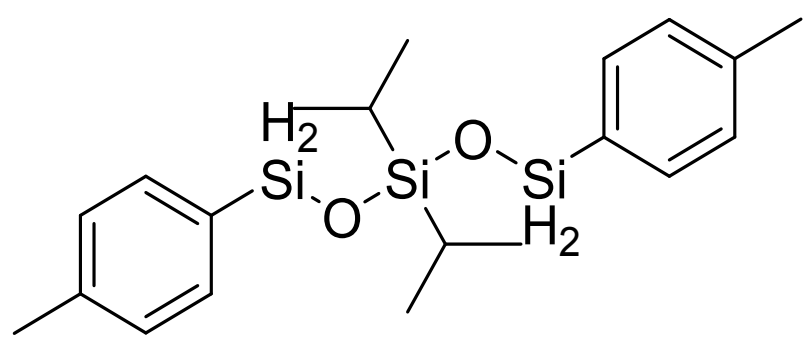

3,3-diisopropyl-1,5-di-p-tolyltrisiloxane (**28**) was obtained as colorless oil in 77% isolated yield. Title compound was previously unknown in literature.

^{1}H NMR (400 MHz, $CDCl_3$): δ 7.42 (d, *J* = 7.6 Hz, 4H), 7.10 (d, *J* = 7.7 Hz, 4H), 5.04 (s, 4H), 2.27 (s, 6H), 0.93 – 0.83 (m, 14H).
^{13}C NMR (101 MHz, $CDCl_3$) δ 140.27, 134.14, 131.17, 128.84, 21.66, 16.86, 13.09.
EI-MS m/z (rel. int.): 345 (100%), 353 (41), 225 (52), 179 (19), 121 (18).

3-hexyl-1,1,1,5,5,5-hexamethyltrisiloxane (29)

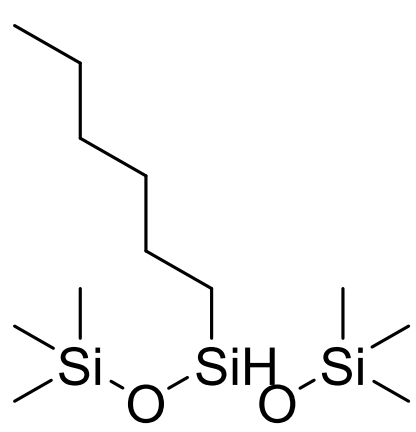

3-hexyl-1,1,1,5,5,5-hexamethyltrisiloxane (**29**) was obtained as colorless oil in 84% isolated yield. The title compound was known in the literature and all spectroscopic data are in agreement.[19]

^{1}H NMR (400 MHz, $CDCl_3$): δ 4.44 (s, 1H), 1.25 – 1.13 (m, 8H), 0.77 (t, *J* = 6.8 Hz, 3H), 0.40 (t, *J* = 1.8 Hz, 2H), 0.00 (s, 18H).
^{13}C NMR (101 MHz, $CDCl_3$) δ 30.88, 29.93, 20.90, 20.31, 15.60, 12.43, 0.00.
EI-MS m/z (rel. int.): 277 (42%), 207 (100), 193 (63), 133 (11), 73 (31).

1,5-di-tert-butyl-3-hexyl-1,1,5,5-tetramethyltrisiloxane (30)

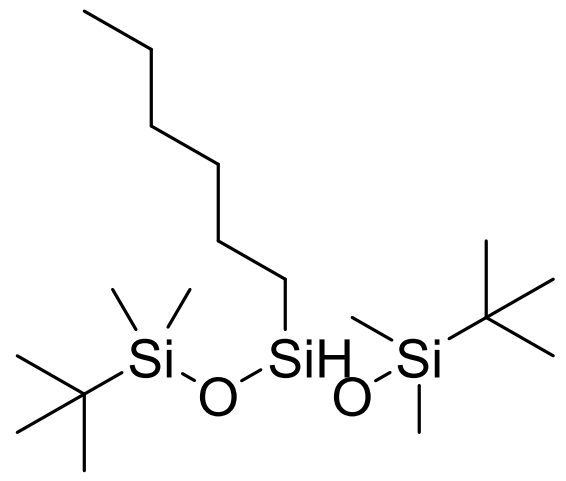

1,5-di-tert-butyl-3-hexyl-1,1,5,5-tetramethyltrisiloxane (**30**) was obtained as colorless oil in 88% isolated yield. The title compound was known in the literature and all spectroscopic data are in agreement.[19]

^{1}H NMR (400 MHz, $CDCl_3$): δ 4.56 (s, 1H), 1.34 – 1.17 (m, 9H), 0.83 (d, J = 2.8 Hz, 20H), 0.62 – 0.30 (m, 2H), 0.00 (s, 12H).
^{13}C NMR (101 MHz, $CDCl_3$) δ 32.66, 31.64, 25.65, 22.58, 22.09, 18.11, 17.48, 14.10, -3.11, -3.16.
EI-MS m/z (rel. int.): 319 (67%), 235 (78), 207 (74), 193 (100), 73 (80).

trimethoxy(phenyl)silane (31)

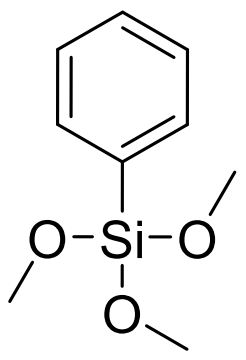

Trimethoxy(phenyl)silane (**31**) was obtained as colorless oil in 82% isolated yield. The title compound was known in the literature and all spectroscopic data are in agreement.[25]

^{1}H NMR (400 MHz, $CDCl_3$): δ 7.76 – 7.67 (m, 2H), 7.51 – 7.29 (m, 3H), 3.66 (s, 9H).
^{13}C NMR (101 MHz, $CDCl_3$) δ 134.78, 134.57, 130.67, 128.02, 50.86.
EI-MS m/z (rel. int.): 198 (30%), 167 (21), 120 (100), 91 (64), 59 (33).

triethoxy(phenyl)silane (32)

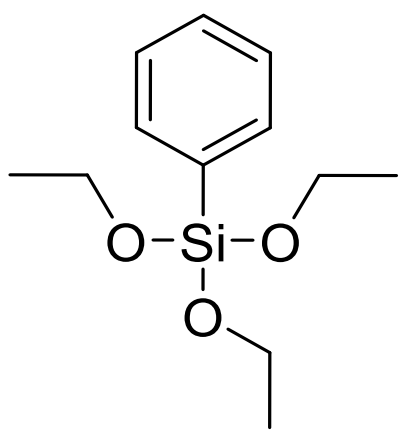

Triethoxy(phenyl)silane (**32**) was obtained as colorless oil in 71% isolated yield. The title compound was known in the literature and all spectroscopic data are in agreement.[36]

^{1}H NMR (400 MHz, C_6D_6): δ 7.71 – 7.68 (m, 2H), 7.06 (d, J = 2.2 Hz, 3H), 3.69 (q, J = 7.0 Hz, 6H), 1.00 (t, J = 7.0 Hz, 9H).
^{13}C NMR (101 MHz, C_6D_6) δ 134.89, 131.82, 130.18, 58.54, 18.14.
EI-MS m/z (rel. int.): 195 (51%), 162 (32), 147 (100), 135 (33), 78 (32).

bis(pentan-3-yloxy)(phenyl)silane (33)

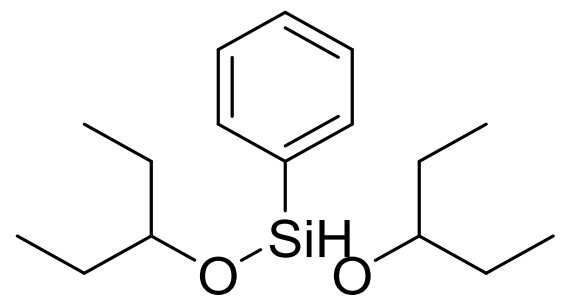

Bis(pentan-3-yloxy)(phenyl)silane (**33**) was obtained as colorless oil in 81% isolated yield. This compound has been previously mentioned in the literature, but it has not been fully characterized.[37]

^{1}H NMR (400 MHz, C_6D_6): δ 7.61 – 7.57 (m, 2H), 7.00 (dd, *J* = 5.5, 1.7 Hz, 3H), 5.13 (s, 1H), 3.72 – 3.38 (m, 2H), 1.46 – 1.01 (m, 8H), 0.69 (dt, *J* = 22.1, 7.5 Hz, 12H).
^{13}C NMR (101 MHz, C_6D_6) δ 134.81, 134.06, 130.21, 127.81, 76.25, 29.26, 9.61.
EI-MS m/z (rel. int.): 251 (62%), 139 (25), 123 (100), 105 (10), 78 (15).

dimethoxydiphenylsilane (34)

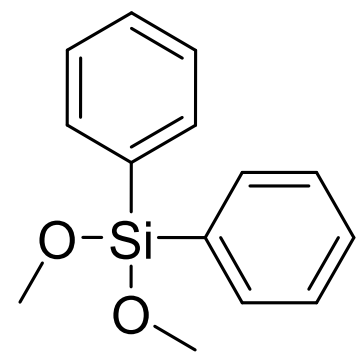

Dimethoxydiphenylsilane (**34**) was obtained as colorless oil in 97% isolated yield. The title compound was known in the literature and all spectroscopic data are in agreement.[25]

^{1}H NMR (400 MHz, C_6D_6): δ 7.88 – 7.74 (m, 4H), 7.27 – 7.12 (m, 6H), 3.45 (s, 6H).
^{13}C NMR (101 MHz, C_6D_6) δ 135.02, 132.72, 130.24, 127.88, 50.27.
EI-MS m/z (rel. int.): 244 (18%), 167 (100), 137 (31), 107 (27), 77 (19).

diethoxy(phenyl)silane (35)

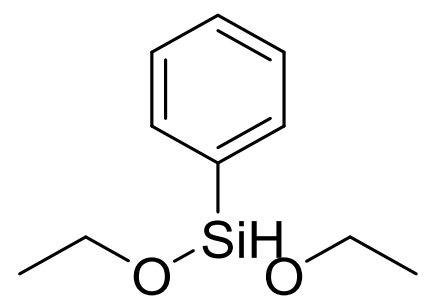

Diethoxy(phenyl)silane (**35**) was obtained as colorless oil in 64% isolated yield. The title compound was known in the literature and all spectroscopic data are in agreement.[38]

^{1}H NMR (400 MHz, $CDCl_3$): δ 7.57 (tt, *J* = 4.6, 1.5 Hz, 2H), 7.40 – 7.27 (m, 3H), 4.86 (s, 1H), 3.86 – 3.75 (m, 4H), 1.23 – 1.13 (m, 6H).
.^{13}C NMR (101 MHz, $CDCl_3$) δ 134.82, 134.06, 130.62, 127.99, 59.47, 18.26.
EI-MS m/z (rel. int.): 195 (80%), 151 (47), 122 (82), 117 (100), 78 (59).

phenyl(prop-2-yn-1-yloxy)silane (36)

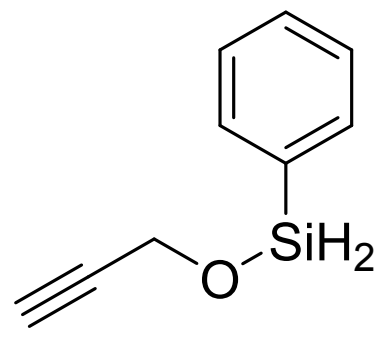

Phenyl(prop-2-yn-1-yloxy)silane (**36**) was obtained as colorless oil in 98% isolated yield. Title compound was previously unknown in literature.

^{1}H NMR (400 MHz, C_6D_6): δ 7.74 – 7.63 (m, 2H), 7.10 – 6.99 (m, 3H), 4.29 (d, *J* = 2.4 Hz, 1H), 4.23 (d, *J* = 2.5 Hz, 2H), 1.94 – 1.91 (m, 2H).
.^{13}C NMR (101 MHz, C_6D_6) δ 135.08, 130.92, 128.64, 127.88, 81.08, 73.61, 51.37.
EI-MS m/z (rel. int.): 210 (18%), 184 (20), 155 (100), 128 (28), 115 (86).

8-phenyl-4,7,9,12-tetraoxa-8-silapentadeca-1,14-diene (37)

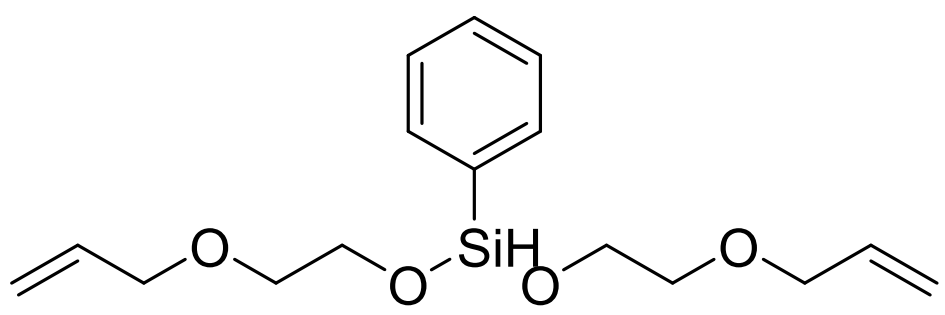

8-phenyl-4,7,9,12-tetraoxa-8-silapentadeca-1,14-diene (**37**) was obtained as colorless oil in 79% isolated yield. Title compound was previously unknown in literature.

^{1}H NMR (400 MHz, C_6D_6): δ 7.97 – 7.84 (m, 2H), 7.26 – 7.17 (m, 3H), 5.97 – 5.67 (m, 2H), 5.20 (dq, *J* = 17.3, 1.8 Hz, 2H), 5.00 (dq, *J* = 10.4, 1.6 Hz, 2H), 4.08 – 3.95 (m, 5H), 3.81 (dt, *J* = 5.3, 1.6 Hz, 4H), 3.51 – 3.36 (m, 4H).
.^{13}C NMR (101 MHz, C_6D_6) δ 135.26, 135.17, 131.07, 130.29, 127.93, 127.69, 127.44, 115.67, 71.68, 71.17, 62.59.
EI-MS m/z (rel. int.): 307 (14%), 189 (17), 165 (39), 147 (100), 117 (26).

1,5-di-tert-butyl-3-decyl-1,1,5,5-tetramethyl-3-phenyltrisiloxane (38)

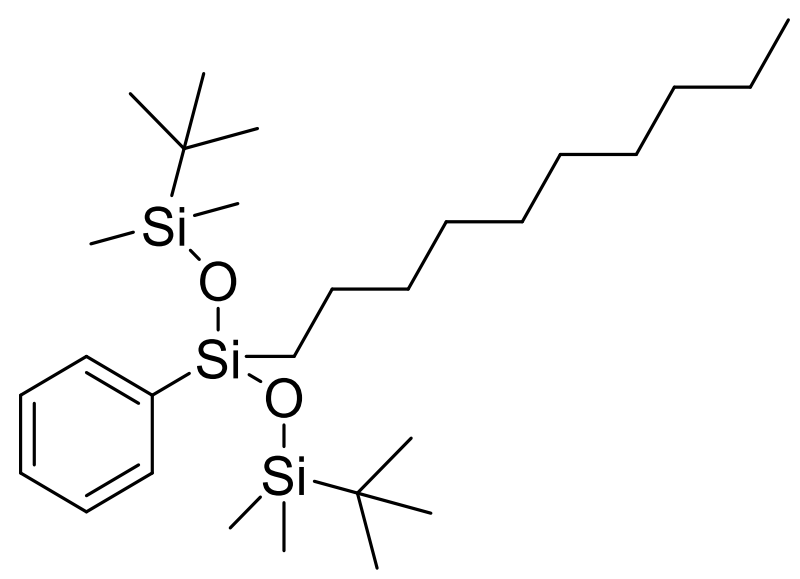

1,5-di-tert-butyl-3-decyl-1,1,5,5-tetramethyl-3-phenyltrisiloxane (**38**) was obtained as colorless oil in 71% isolated yield. Title compound was previously unknown in literature.

^{1}H NMR (400 MHz, C_6D_6): δ 7.52 – 7.47 (m, 2H), 7.32 – 7.24 (m, 3H), 1.36 – 1.13 (m, 14H), 0.89 – 0.75 (m, 25H), 0.07 – -0.10 (m, 12H).

.^{13}C NMR (101 MHz, C_6D_6) δ 137.75, 133.47, 129.22, 127.41, 33.40, 31.84, 29.55, 29.47, 29.27, 29.18, 25.71, 22.99, 22.62, 18.13, 16.25, 14.05, -2.85, -2.87.
EI-MS m/z (rel. int.): 451 (100%), 311 (55), 267 (63), 134 (81), 57 (83).

1-(tert-butyl)-1,1,3-trimethyl-3-phenyl-3-(2-(trimethoxysilyl)ethyl)disiloxane (39)

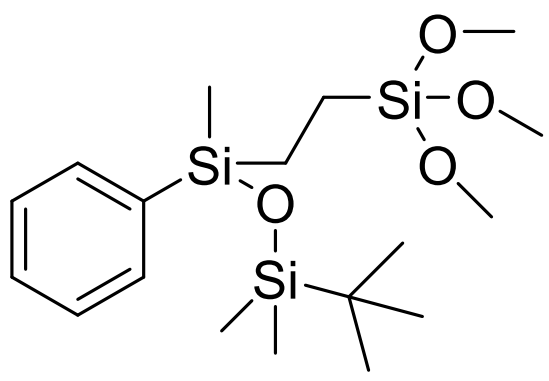

1-(tert-butyl)-1,1,3-trimethyl-3-phenyl-3-(2-(trimethoxysilyl)ethyl)disiloxane (**39**) was obtained as colorless oil in 61% isolated yield. Title compound was previously unknown in literature.

^{1}H NMR (400 MHz, $CDCl_3$): δ 7.56 – 7.52 (m, 2H), 7.18 – 7.11 (m, 3H), 3.37 (s, 9H), 0.90 – 0.88 (m, 4H), 0.86 (s, 9H), 0.29 (s, 3H), -0.00 (d, J = 2.7 Hz, 6H).
.^{13}C NMR (101 MHz, $CDCl_3$) δ 140.74, 135.57, 131.51, 129.97, 52.27, 27.81, 20.25, 10.70, 3.41, 0.22, -0.79.
EI-MS m/z (rel. int.): 343 (100%), 209 (15), 193 (12), 135 (23), 120 (28).

SPECTRA DATA FOR ALL PRODUCTS

Mn(CO)$_3$Br(L1) (C18)

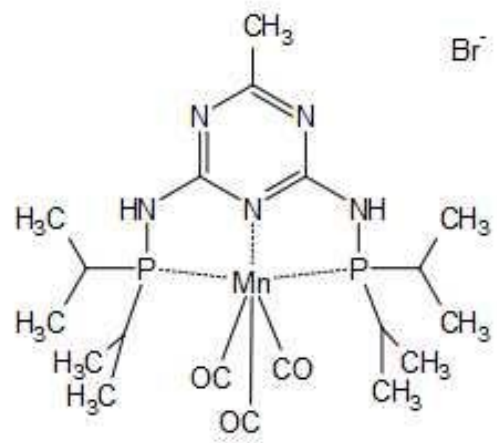

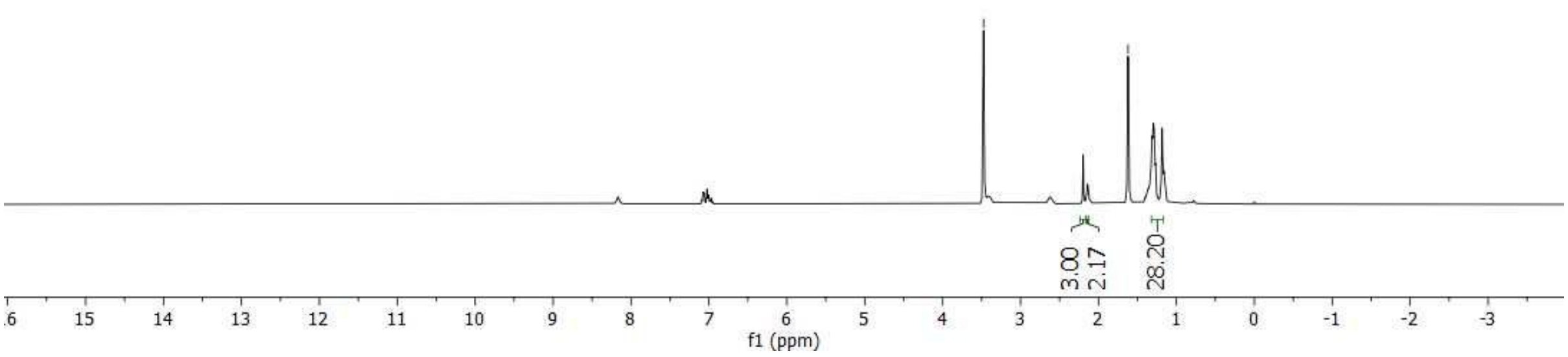

Figure SS1. ^{1}H NMR Spectrum of **C18** (400 MHz, THF)

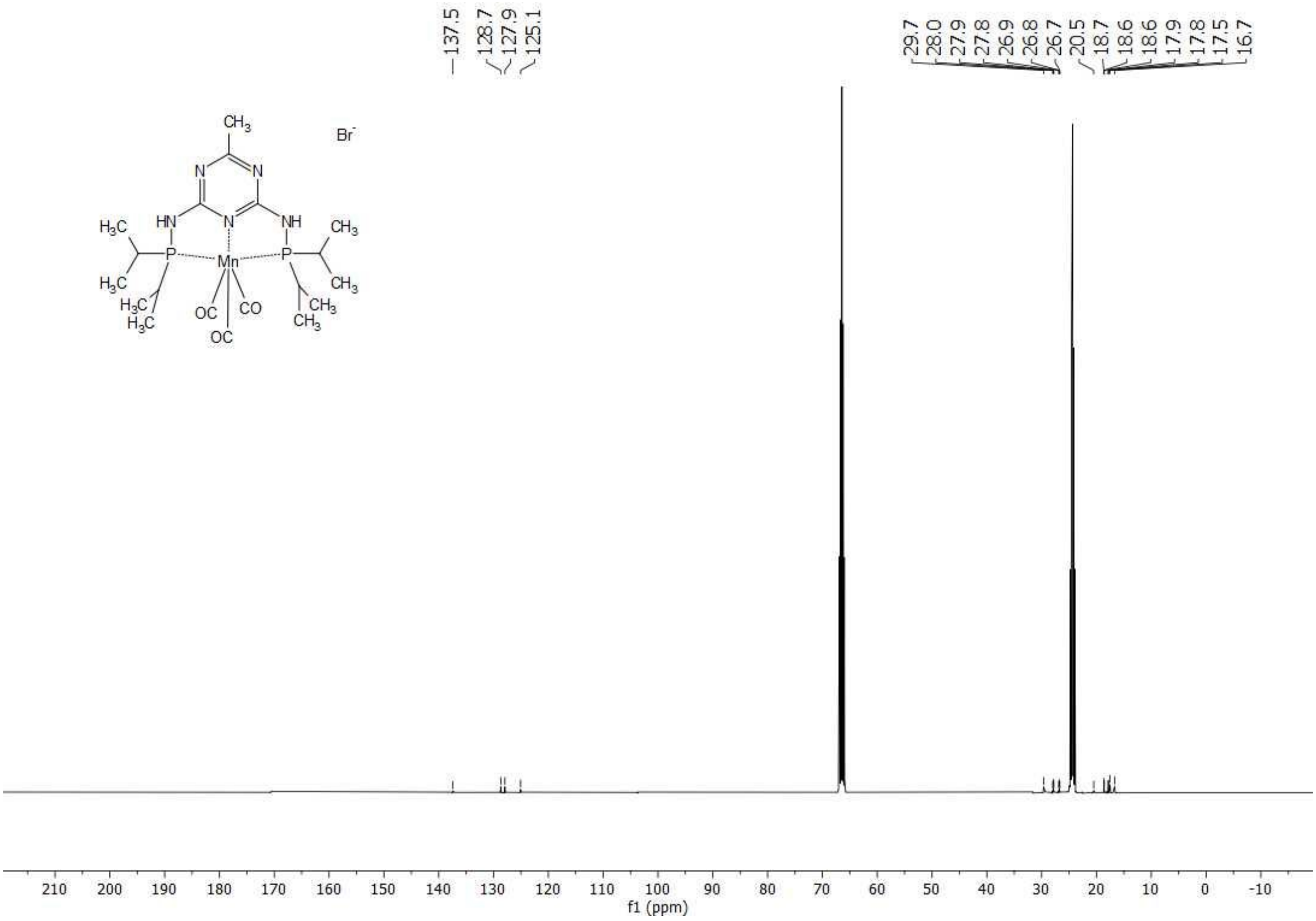

Figure SS2. ^{13}C NMR Spectrum of **C18** (101 MHz, THF)

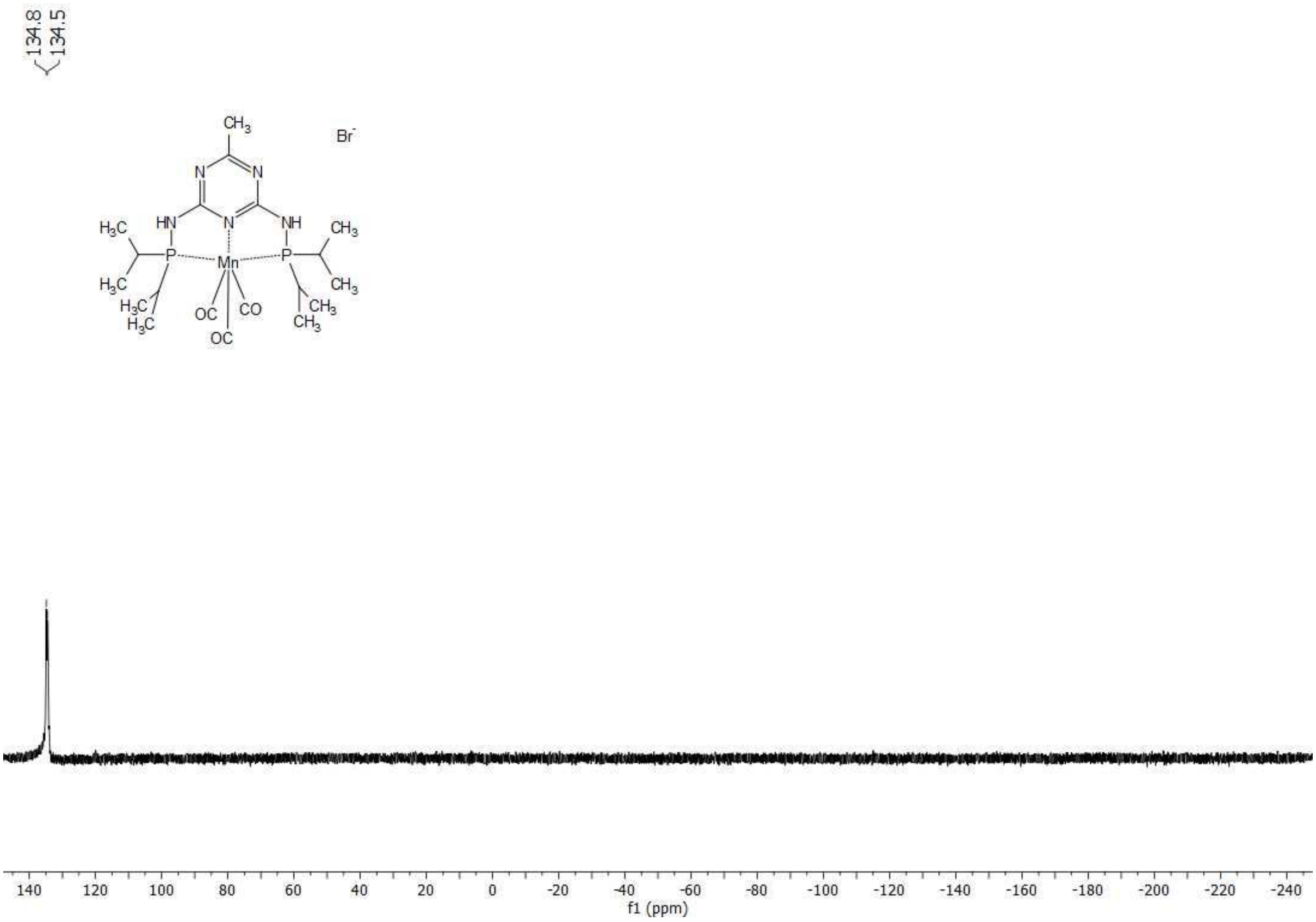

Figure SS3. ^{31}P NMR Spectrum of **C18** (162 MHz, THF)

$Mn(CO)_3Br$(L16) (C19)

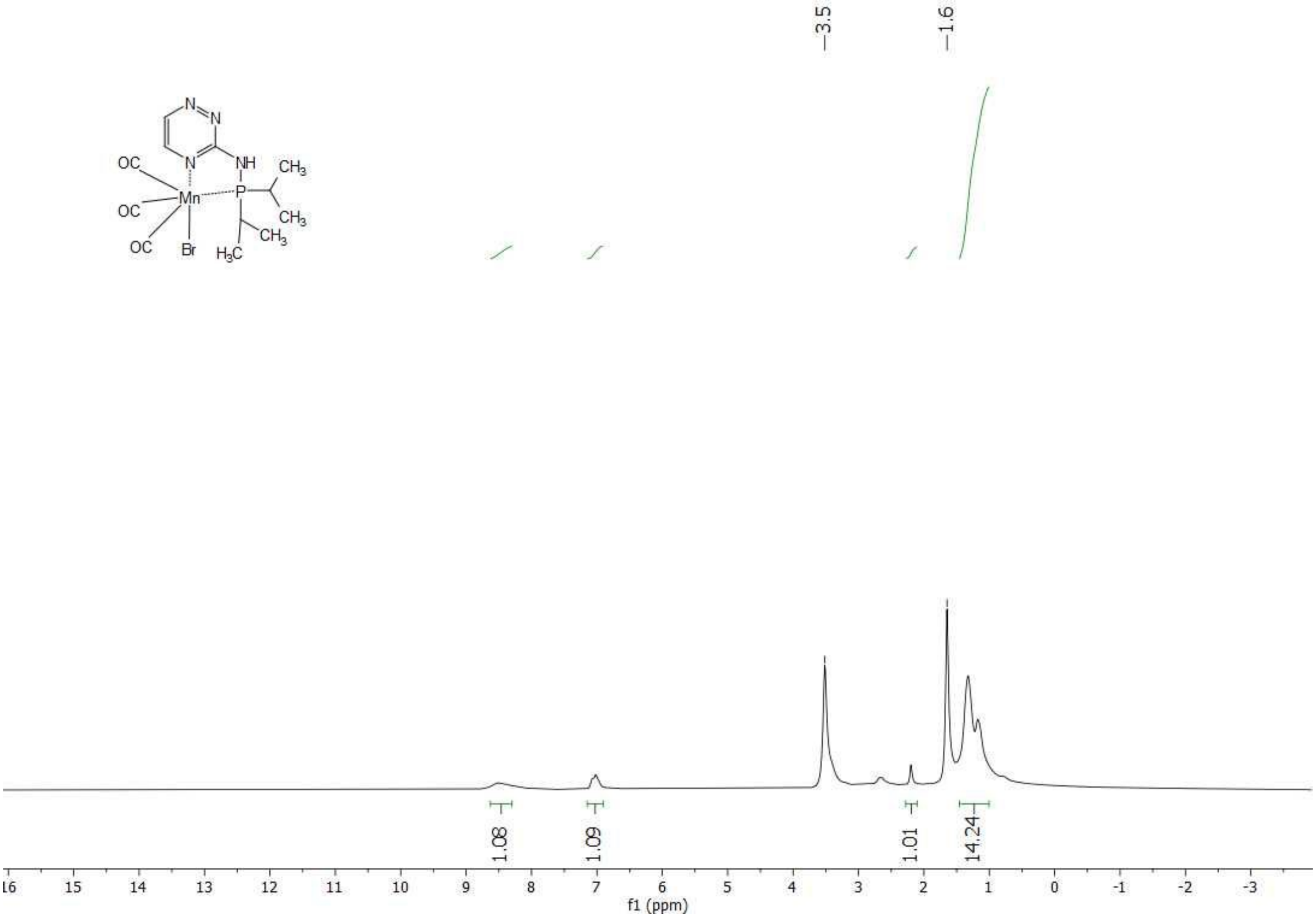

Figure SS4. ^{1}H NMR Spectrum of **C19** (400 MHz, THF)

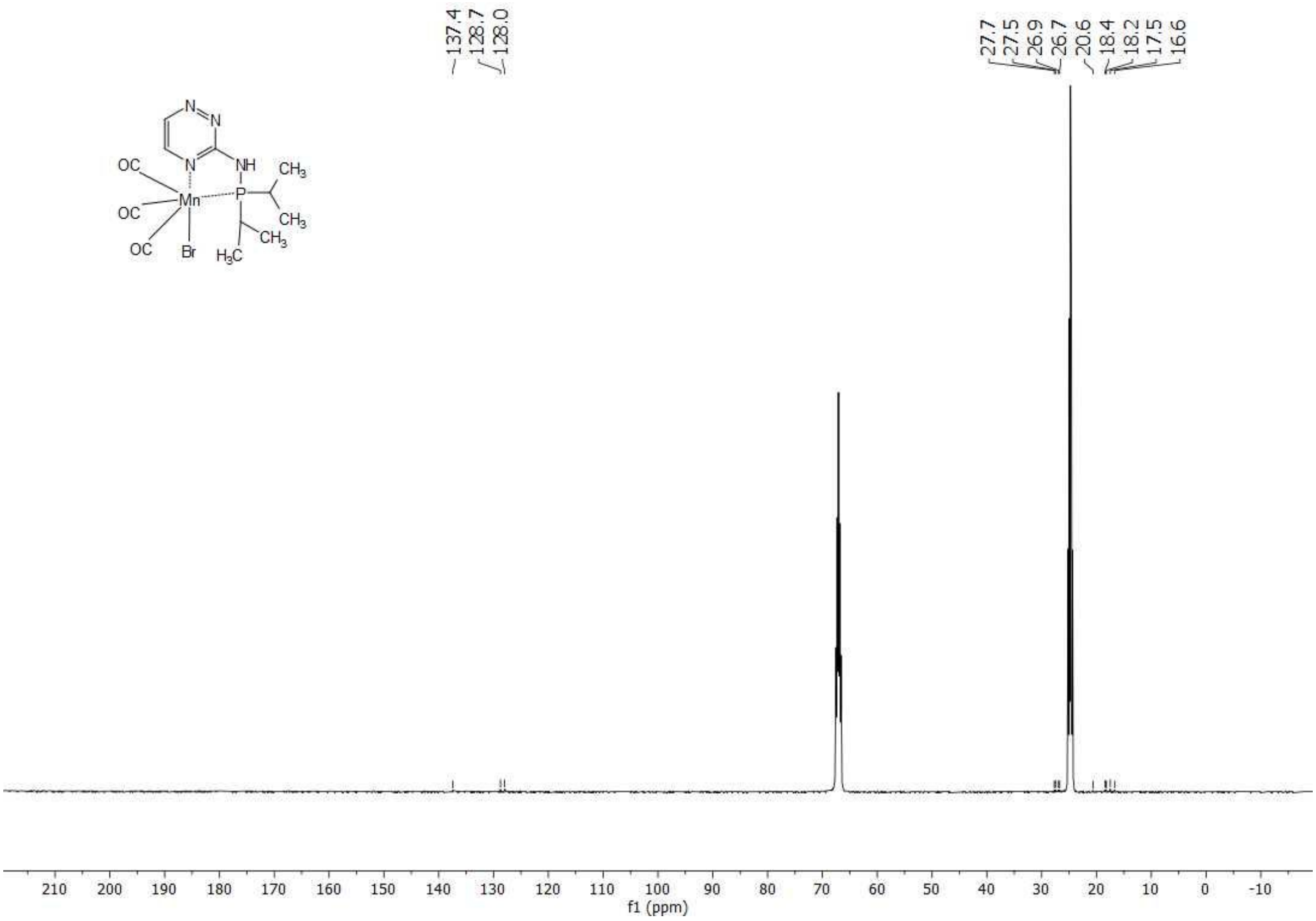

Figure SS5. ^{13}C NMR Spectrum of **C19** (101 MHz, THF)

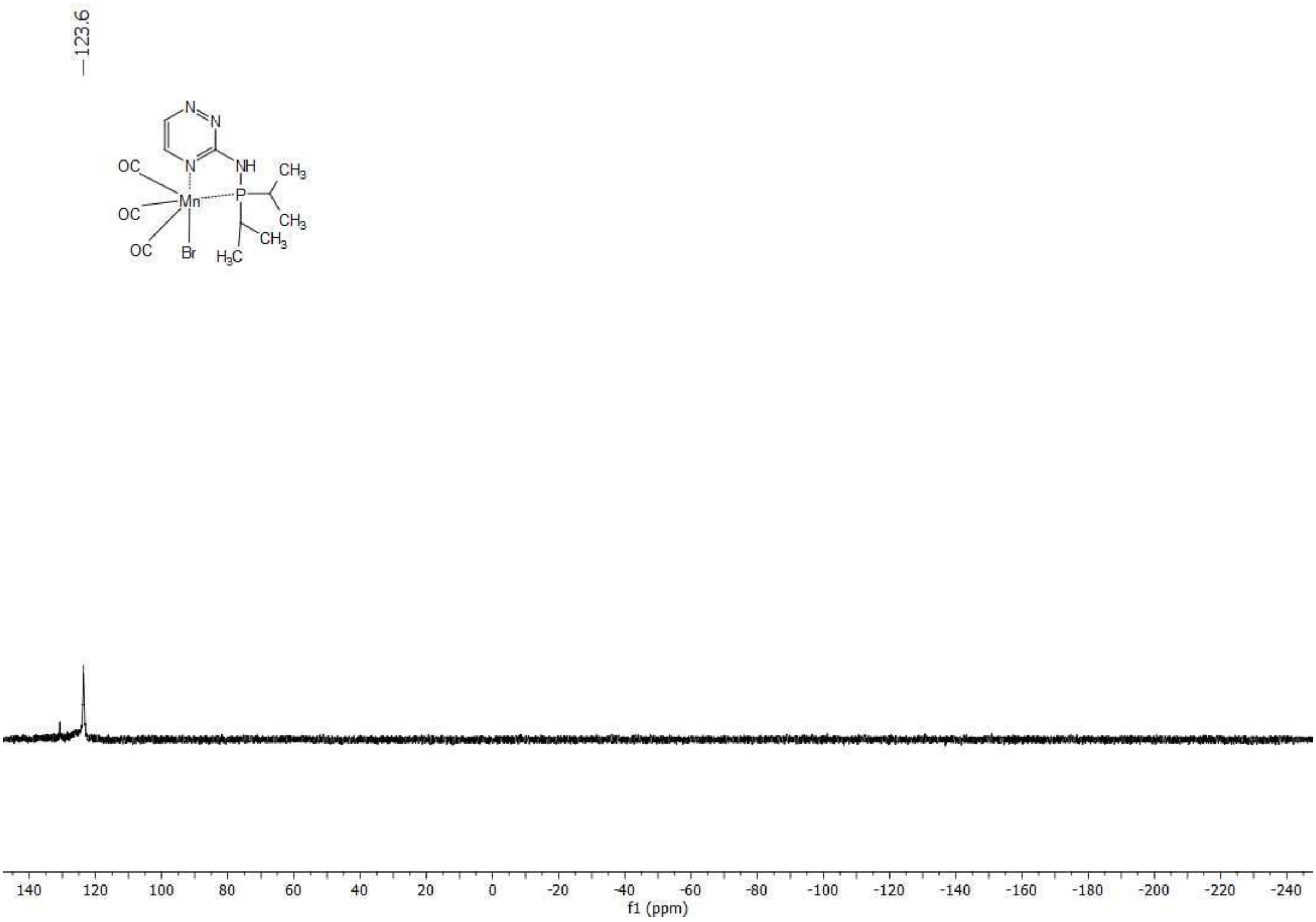

Figure SS6. ^{31}P NMR Spectrum of **C19** (162 MHz, THF)

Mn(CO)$_3$Br(L9) (C21)

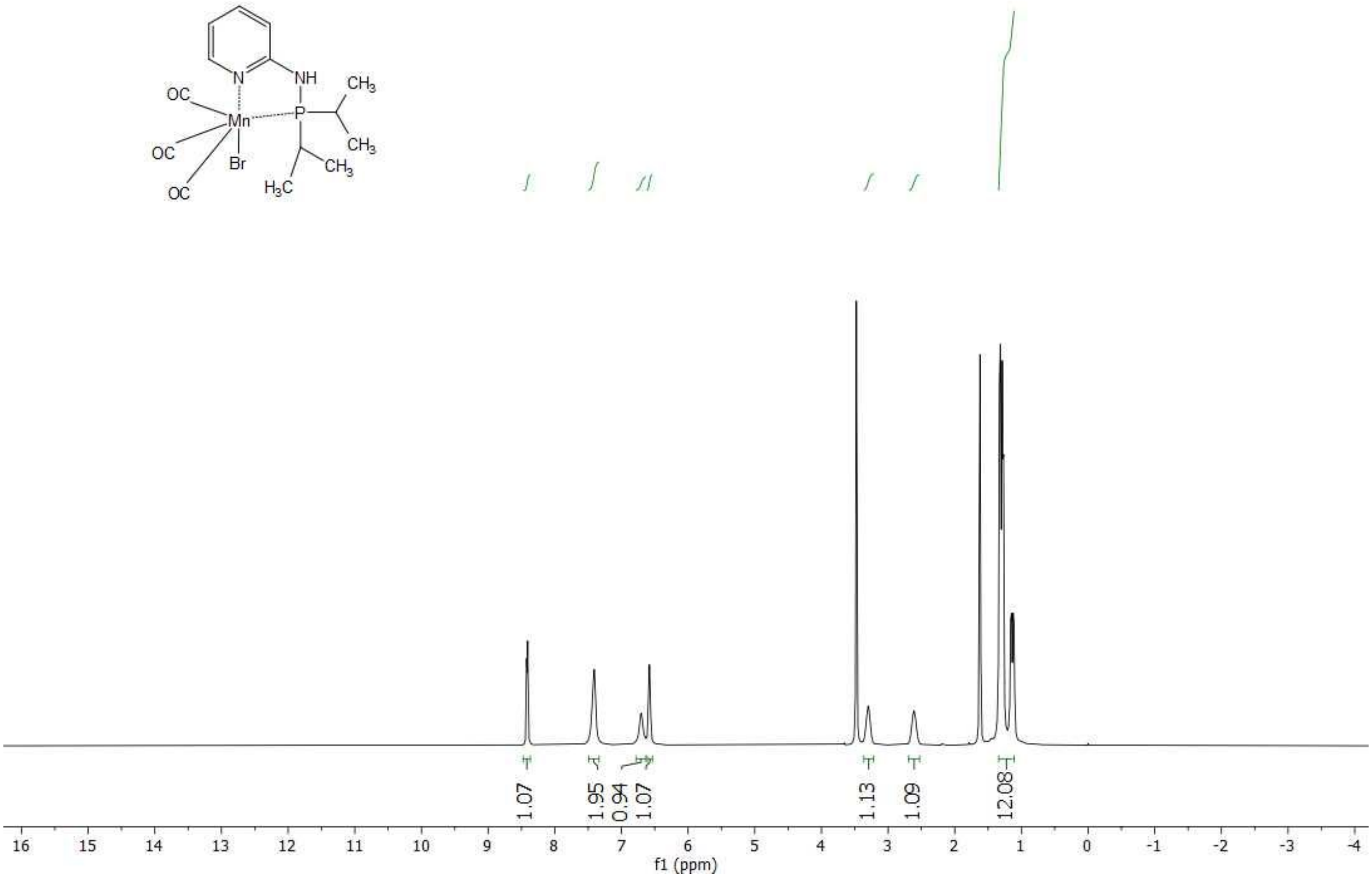

Figure SS7. ^{1}H NMR Spectrum of **C21** (400 MHz, THF)

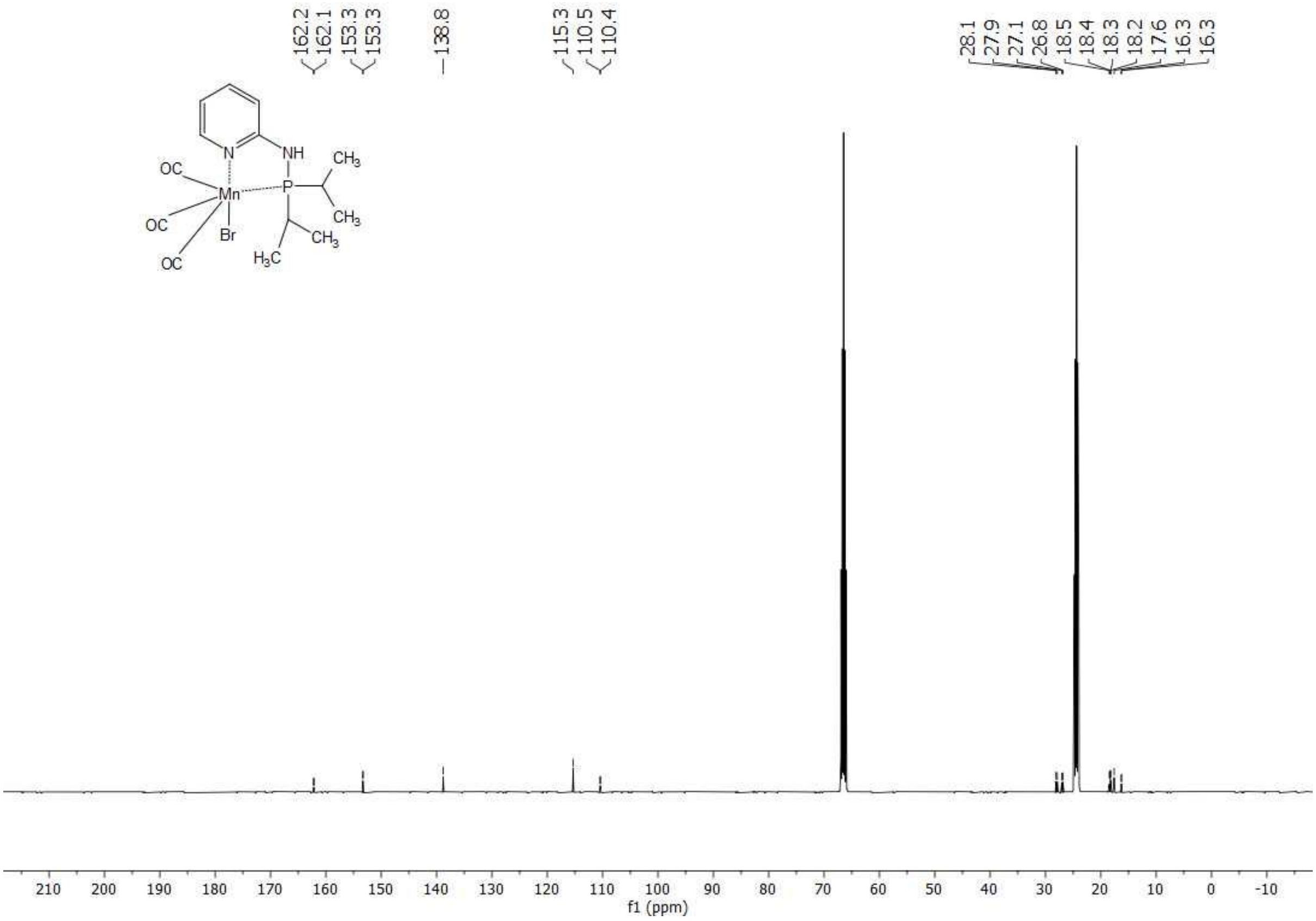

Figure SS8. ^{13}C NMR Spectrum of **C21** (101 MHz, THF)

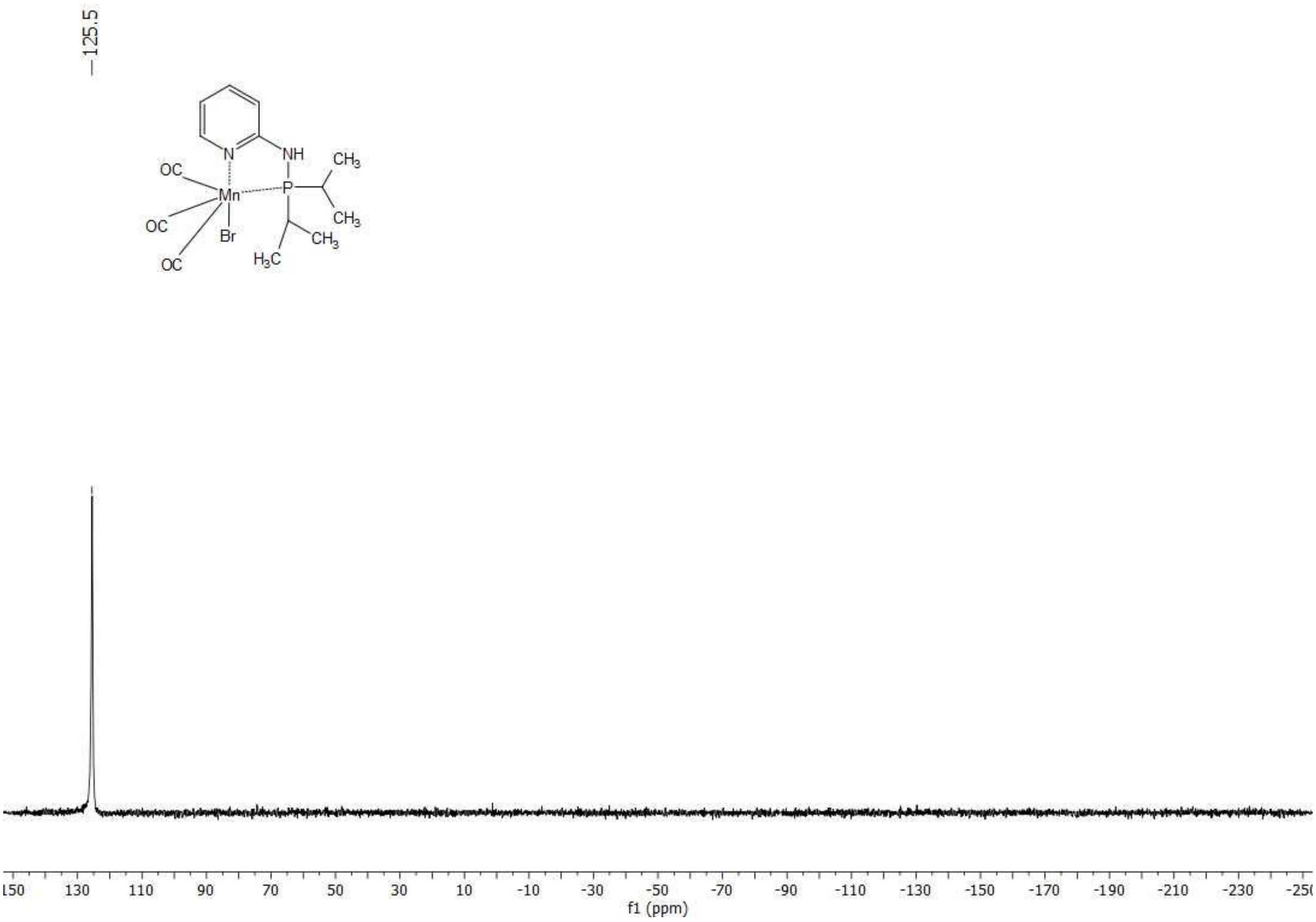

Figure SS9. ^{31}P NMR Spectrum of **C21** (162 MHz, THF)

Mn(CO)$_3$Br(L14) (C22)

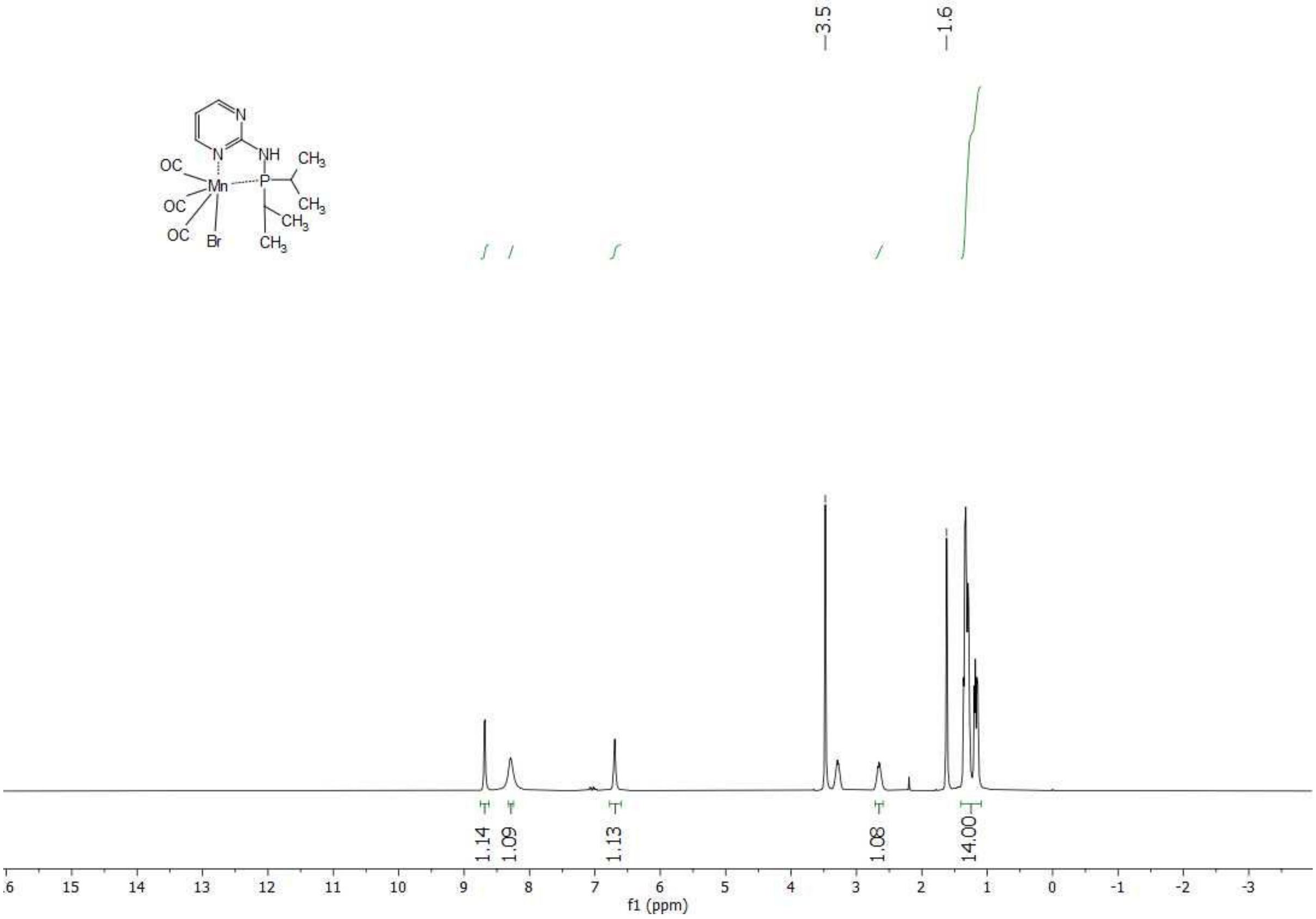

Figure SS10. ^{1}H NMR Spectrum of **C22** (400 MHz, THF)

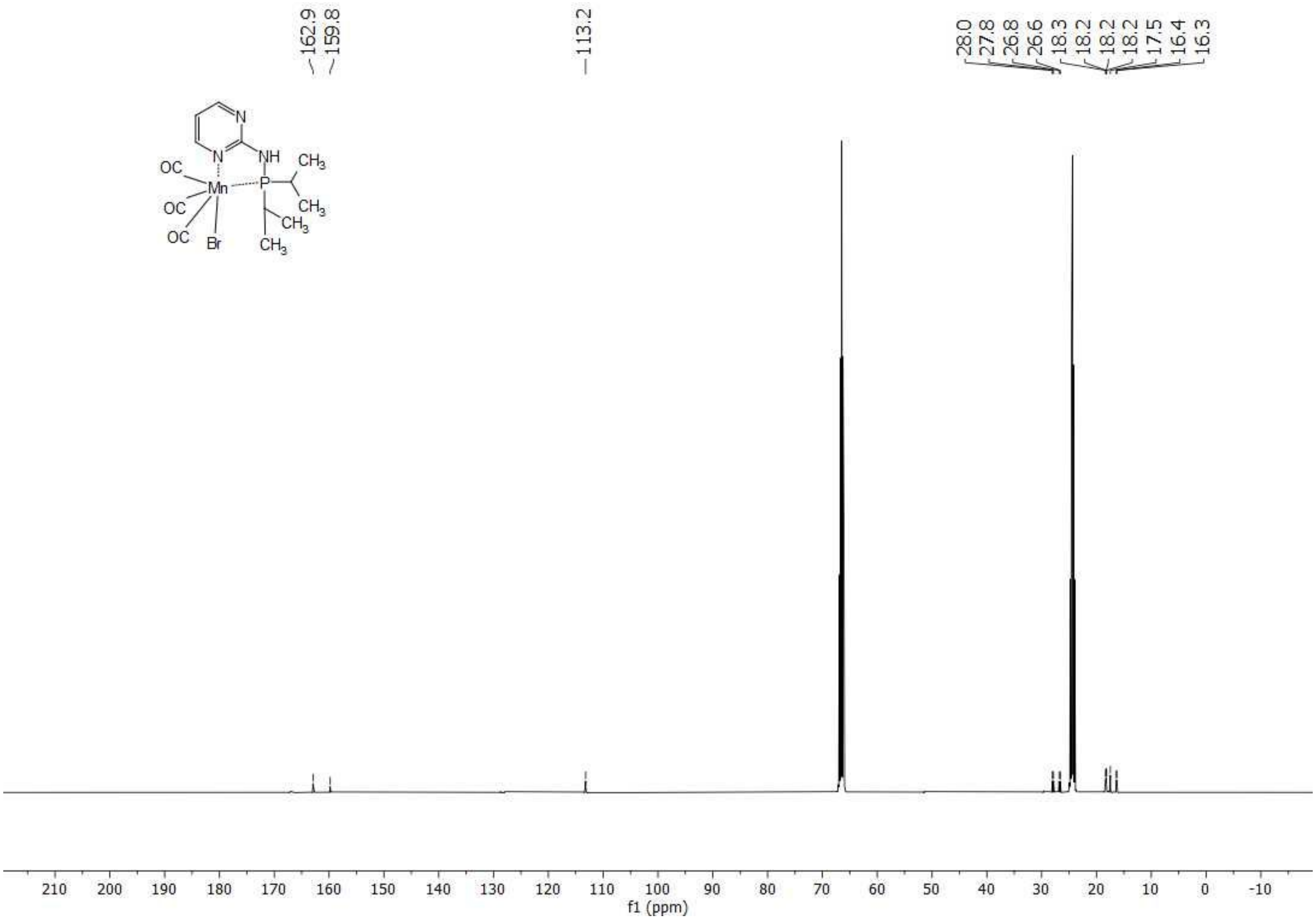

Figure SS11. ^{13}C NMR Spectrum of **C22** (101 MHz, THF)

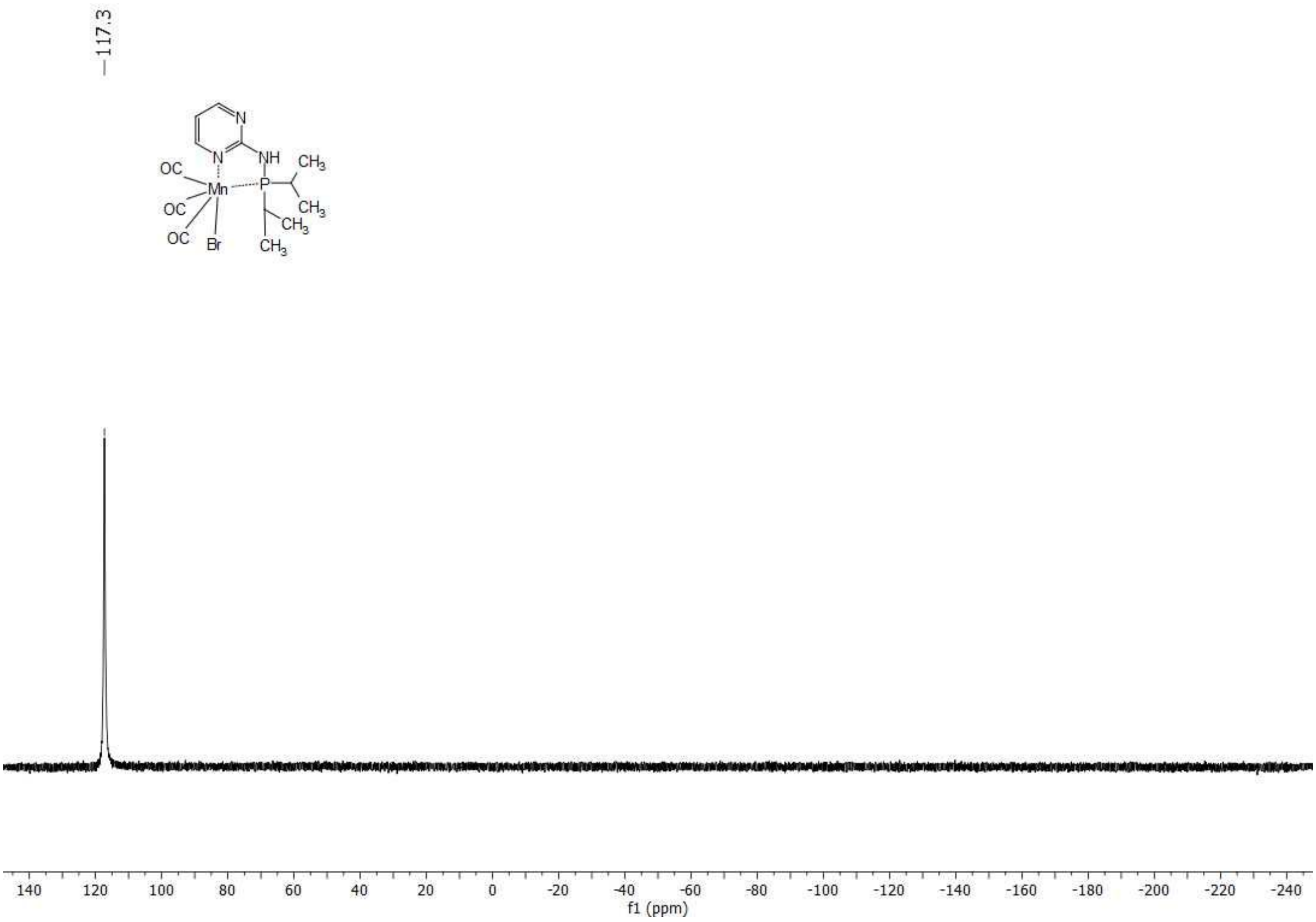

Figure SS12. ^{31}P NMR Spectrum of **C22** (162 MHz, THF)

1,1,1-triethyl-3-phenyldisiloxane (1)

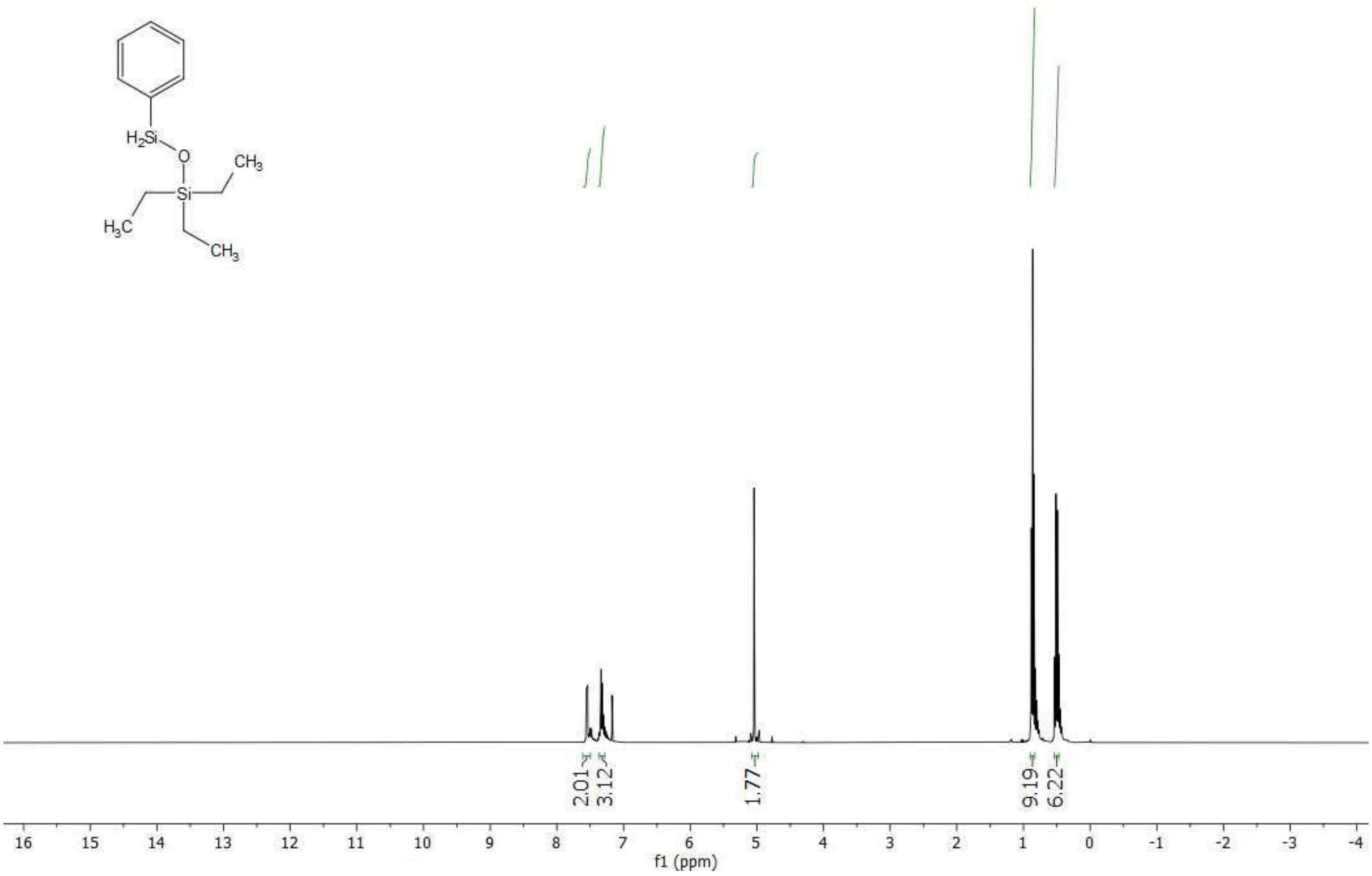

Figure SS13. ^1H NMR Spectrum of **1** (400 MHz, $CDCl_3$)

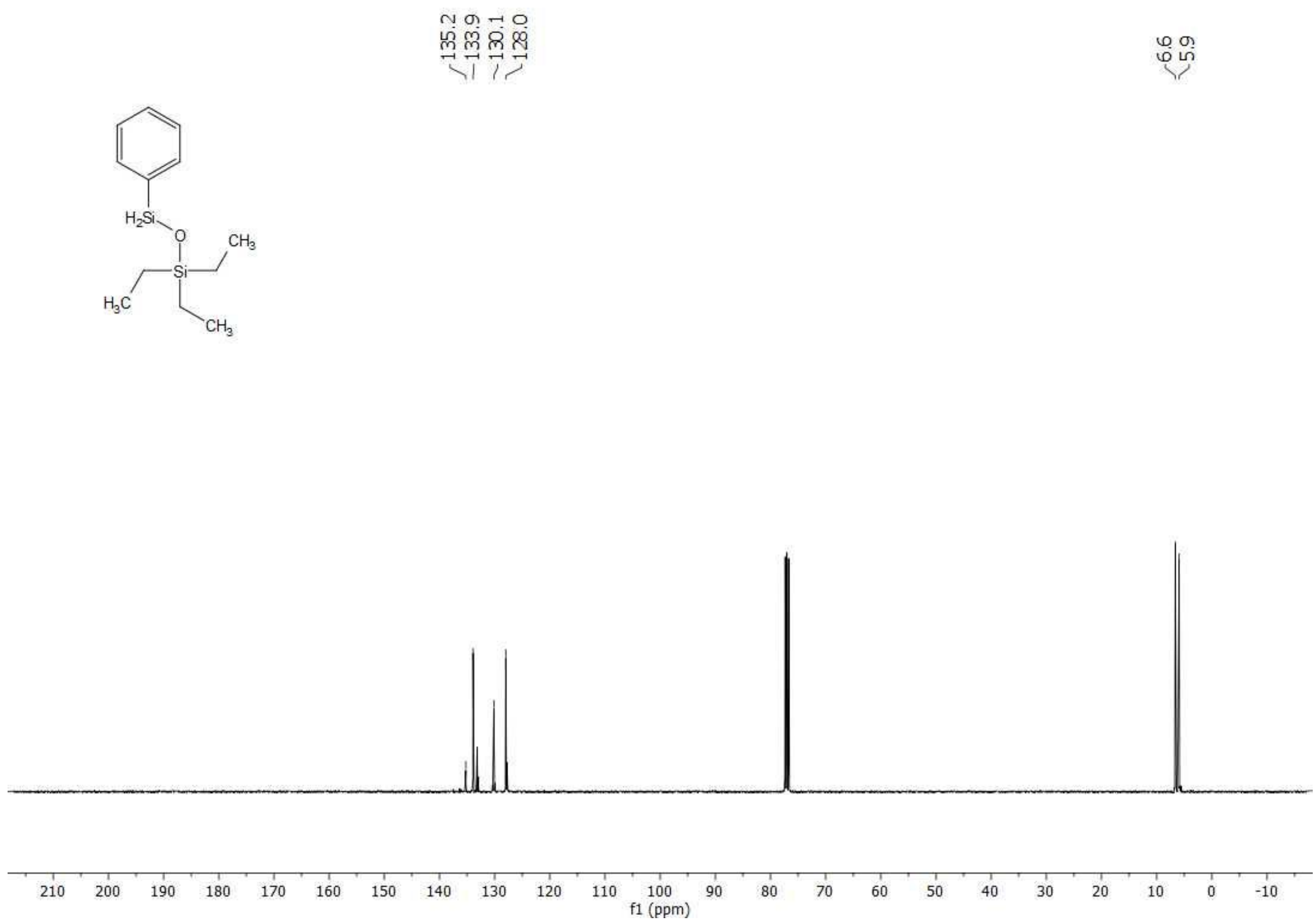

Figure SS14. ^{13}C NMR Spectrum of **1** (101 MHz, $CDCl_3$)

1-(tert-butyl)-1,1-dimethyl-3-phenyldisiloxane (2)

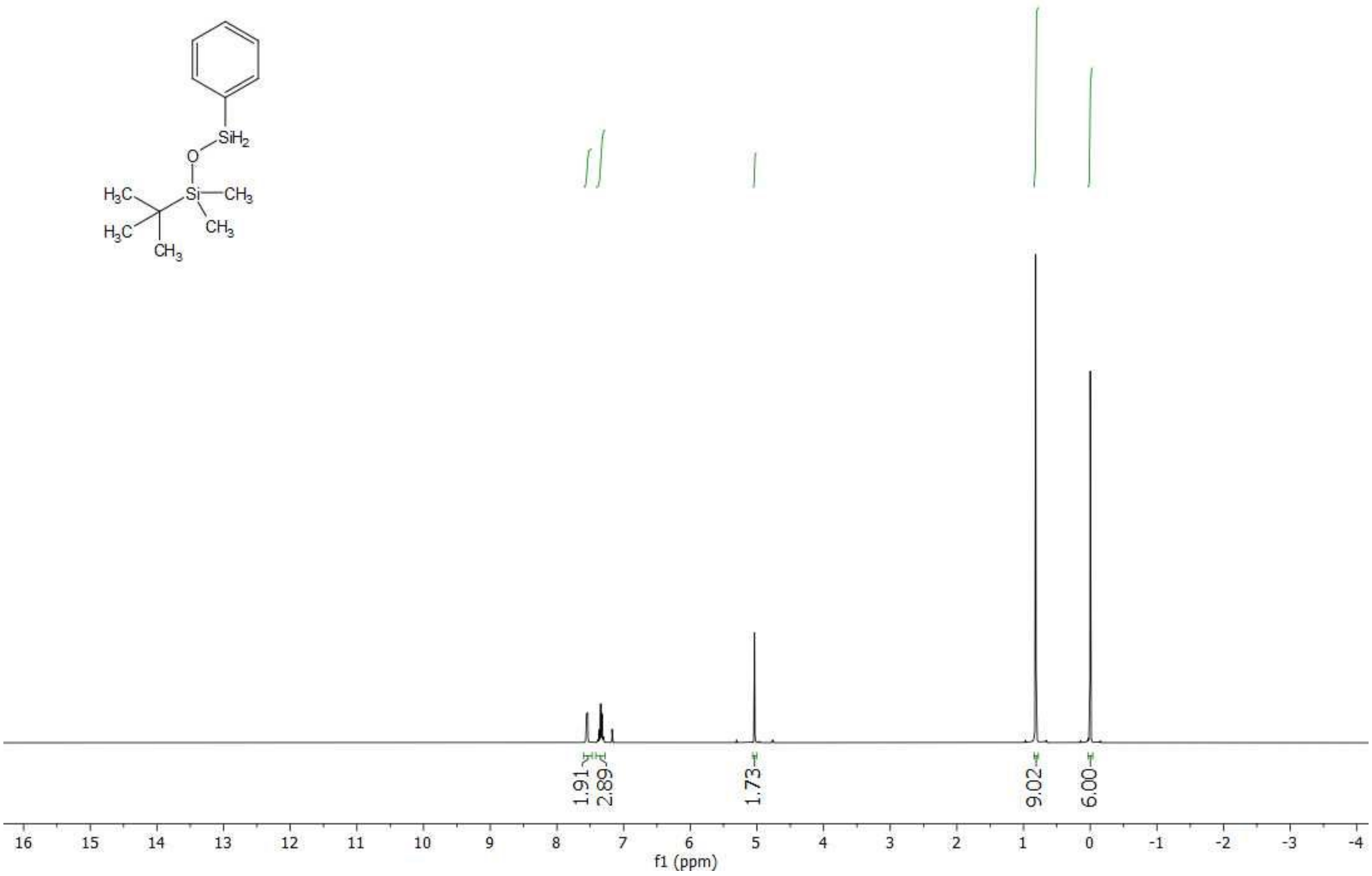

Figure SS15. ^{1}H NMR Spectrum of **2** (400 MHz, $CDCl_3$)

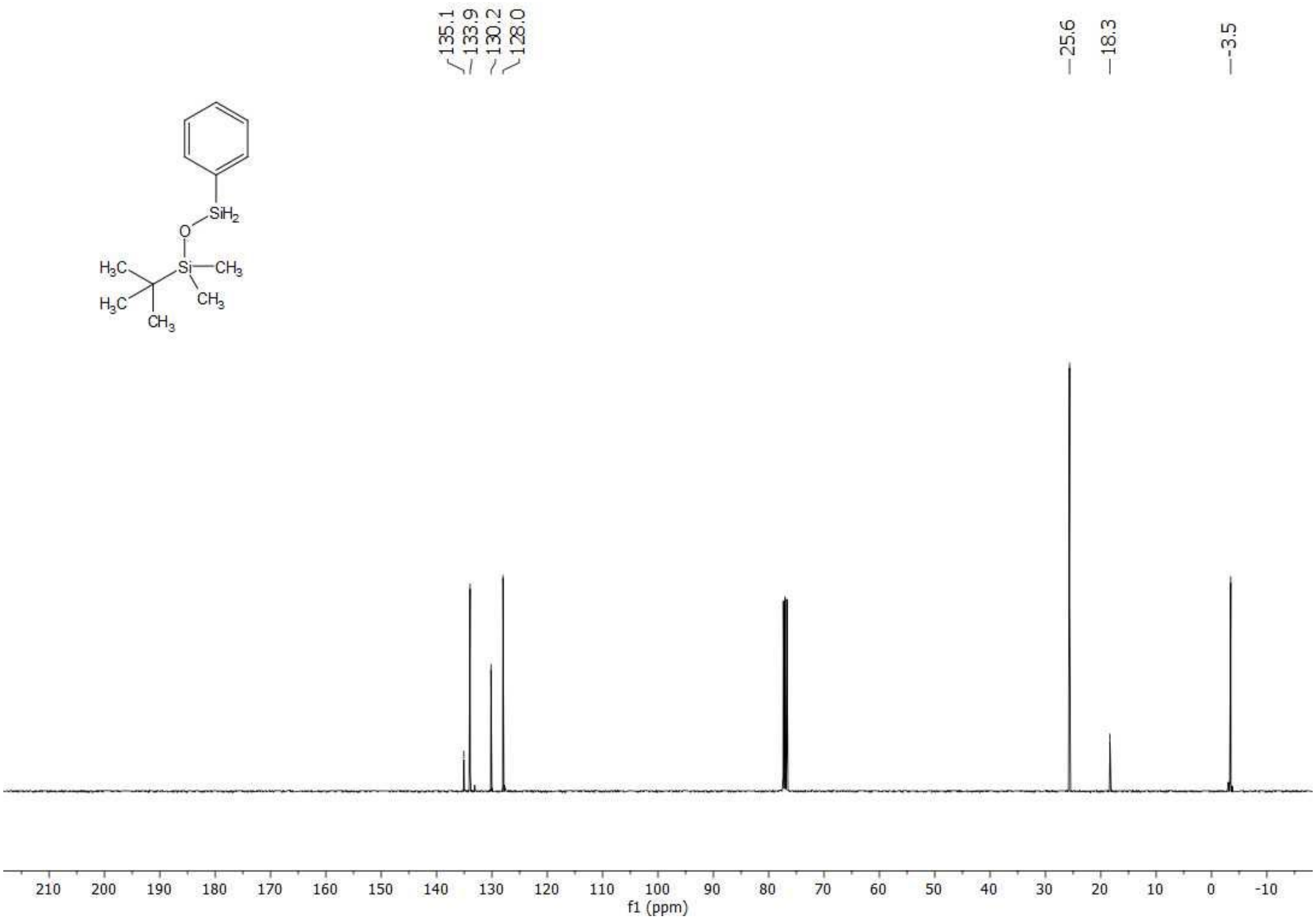

Figure SS16. ^{13}C NMR Spectrum of **2** (101 MHz, $CDCl_3$)

1,1,1-triisopropyl-3-phenyldisiloxane (3)

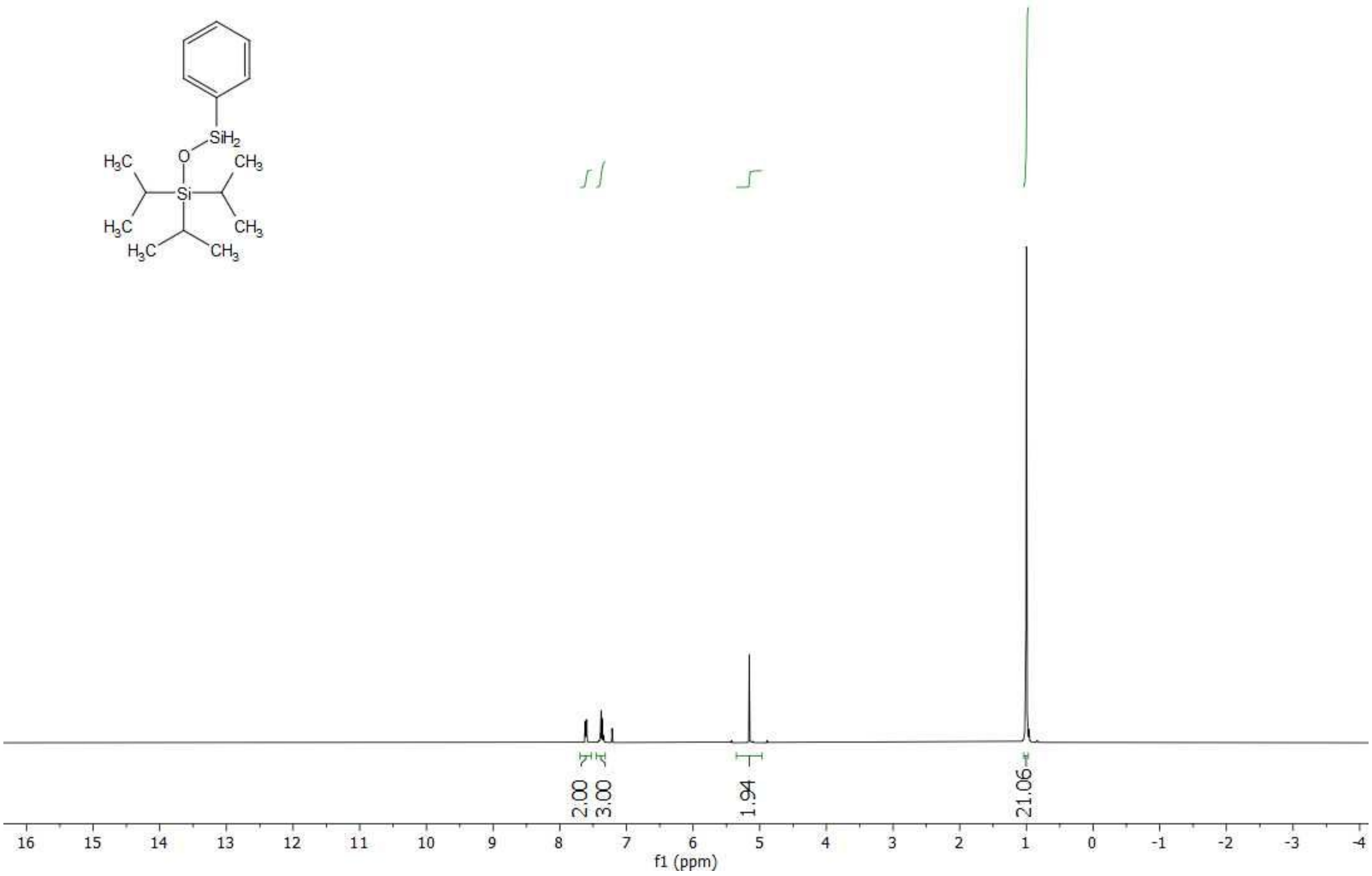

Figure SS17. ^{1}H NMR Spectrum of **3** (400 MHz, $CDCl_3$)

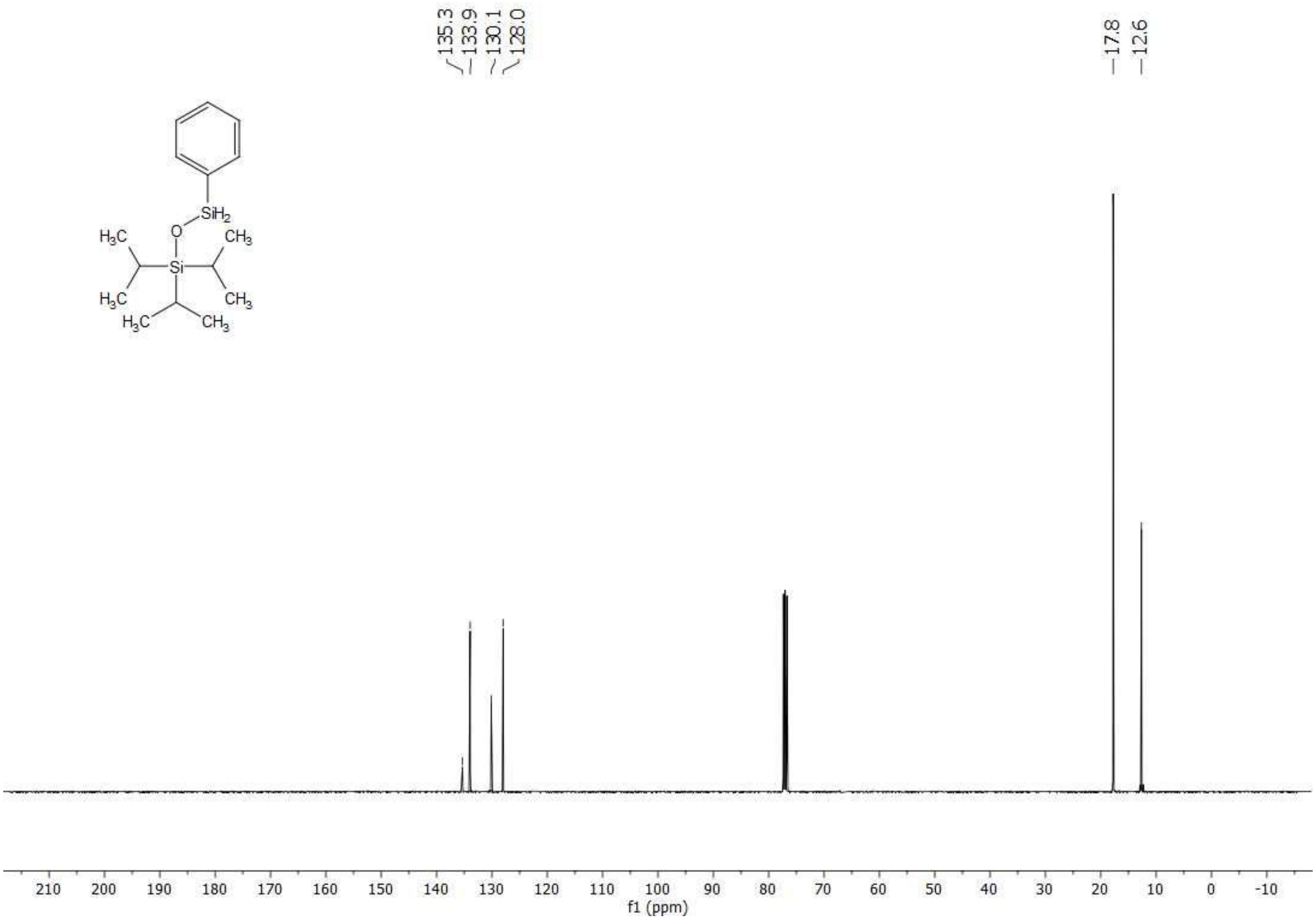

Figure SS18. ^{13}C NMR Spectrum of **3** (101 MHz, $CDCl_3$)

1,1-dimethyl-1,3-diphenyldisiloxane (4)

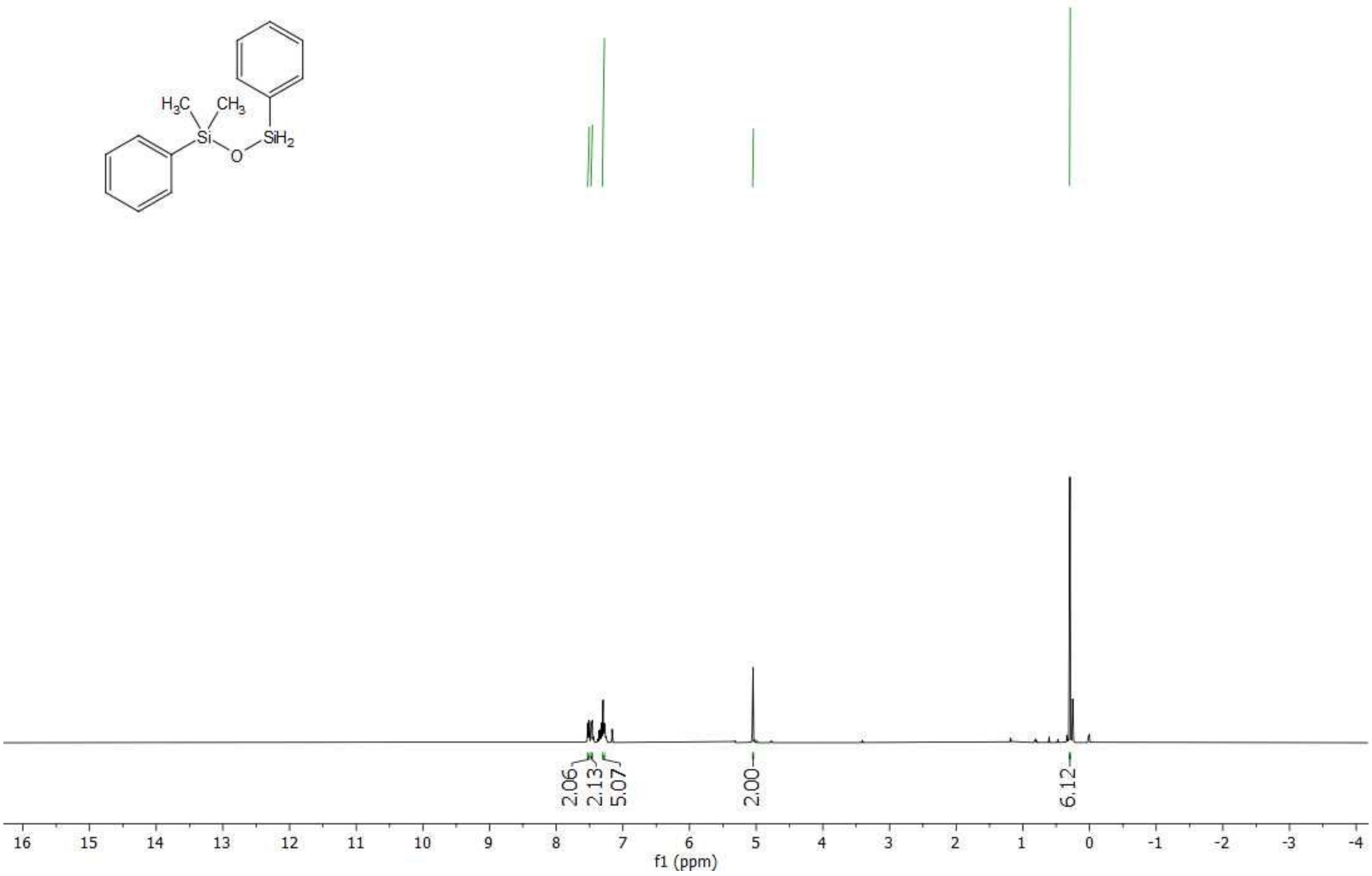

Figure SS19. ^{1}H NMR Spectrum of **4** (400 MHz, $CDCl_3$)

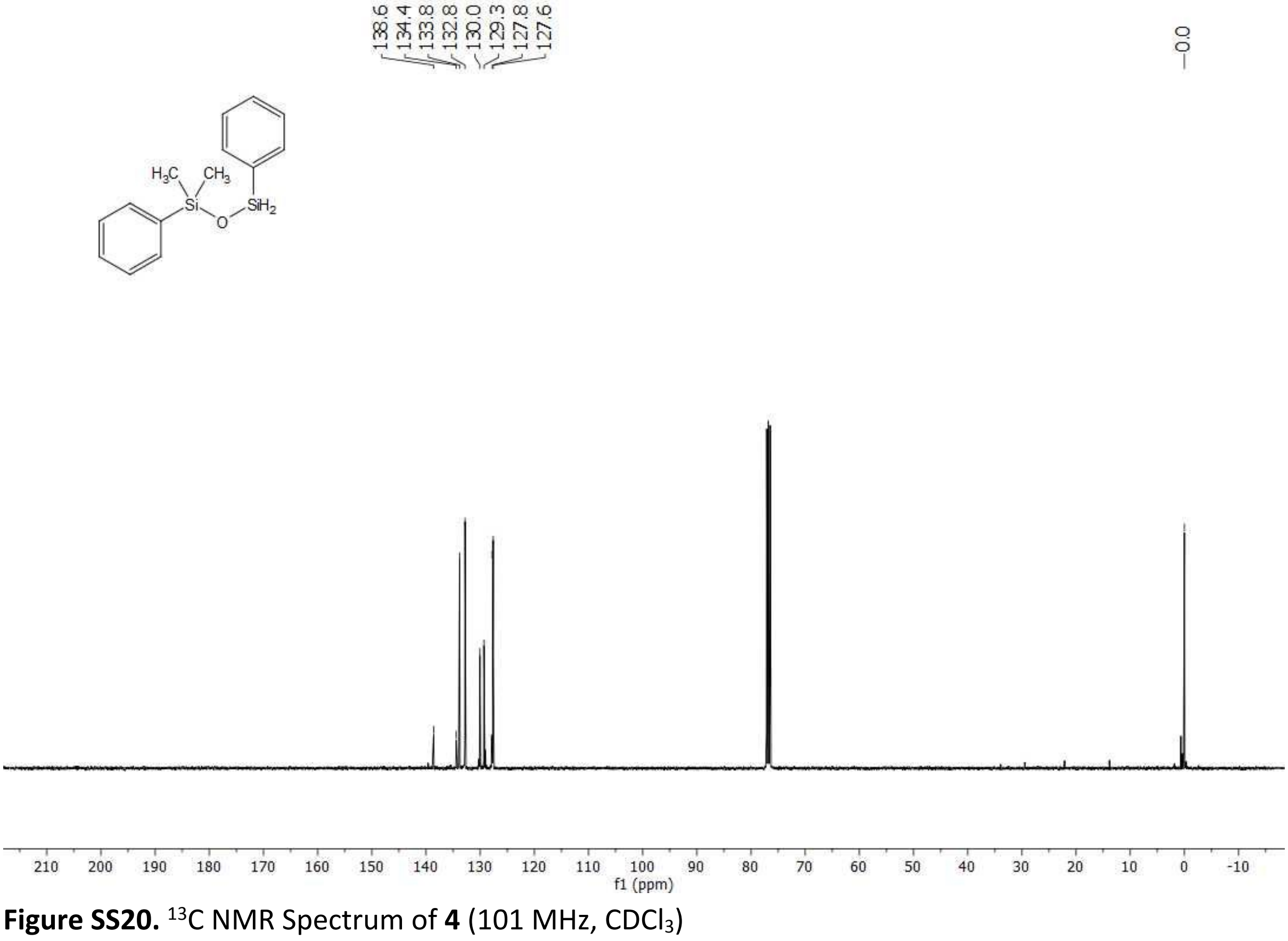

Figure SS20. ^{13}C NMR Spectrum of **4** (101 MHz, $CDCl_3$)

1,1,1-trimethyl-3,3-diphenyldisiloxane (5)

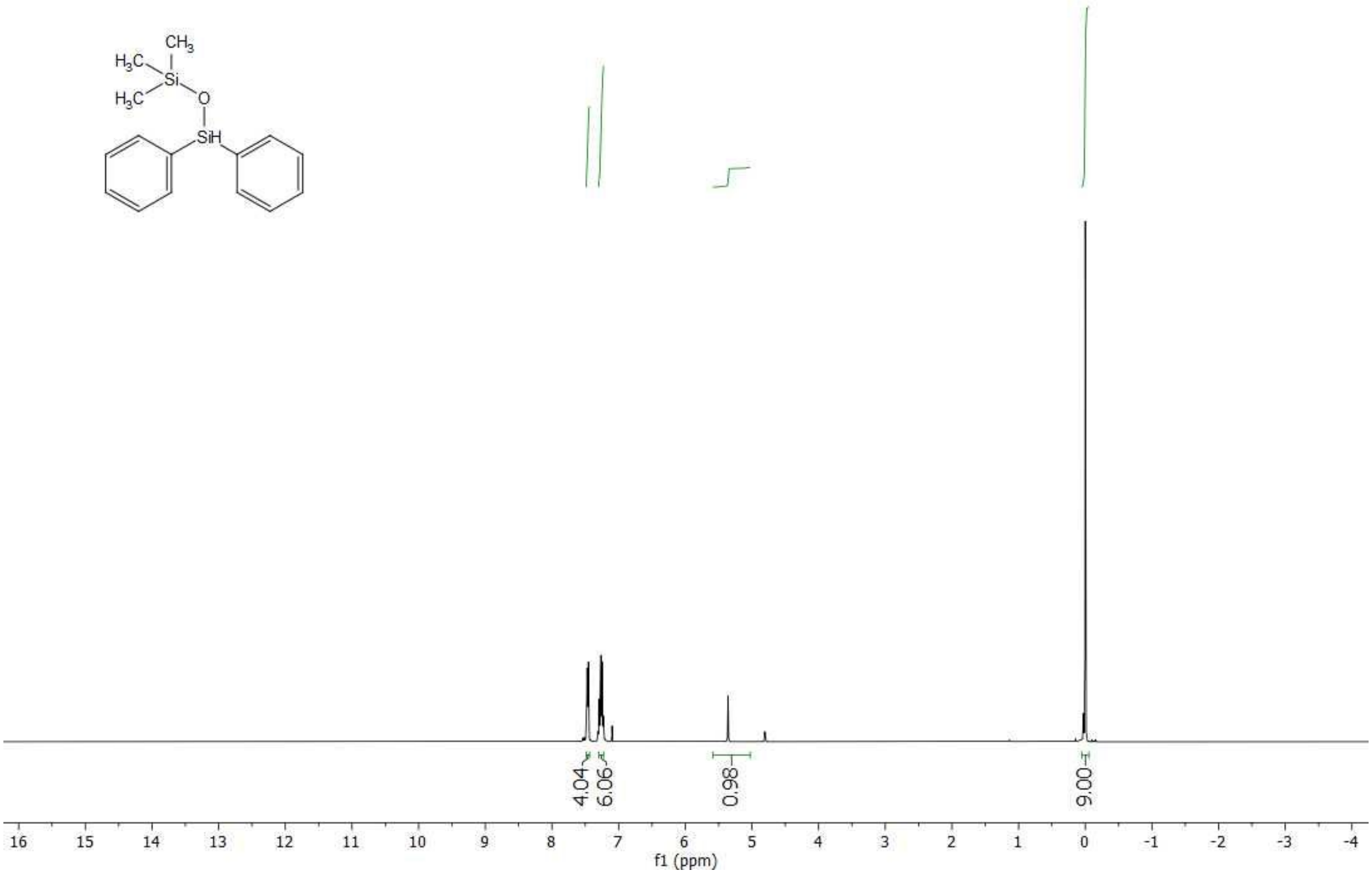

Figure SS21. ^{1}H NMR Spectrum of **5** (400 MHz, $CDCl_3$)

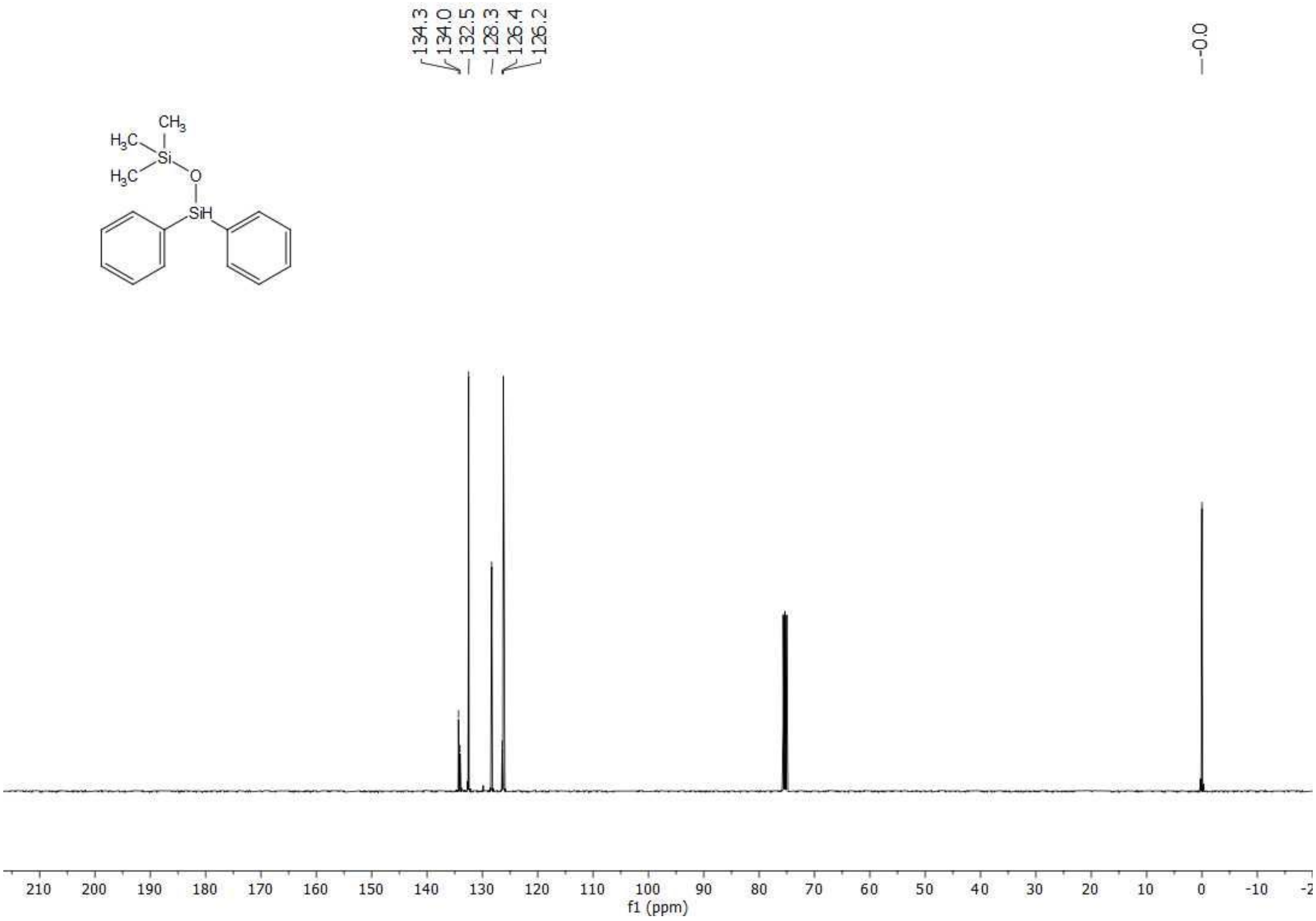

Figure SS22. ^{13}C NMR Spectrum of **5** (101 MHz, $CDCl_3$)

1-(tert-butyl)-1,1-dimethyl-3,3-diphenyldisiloxane (6)

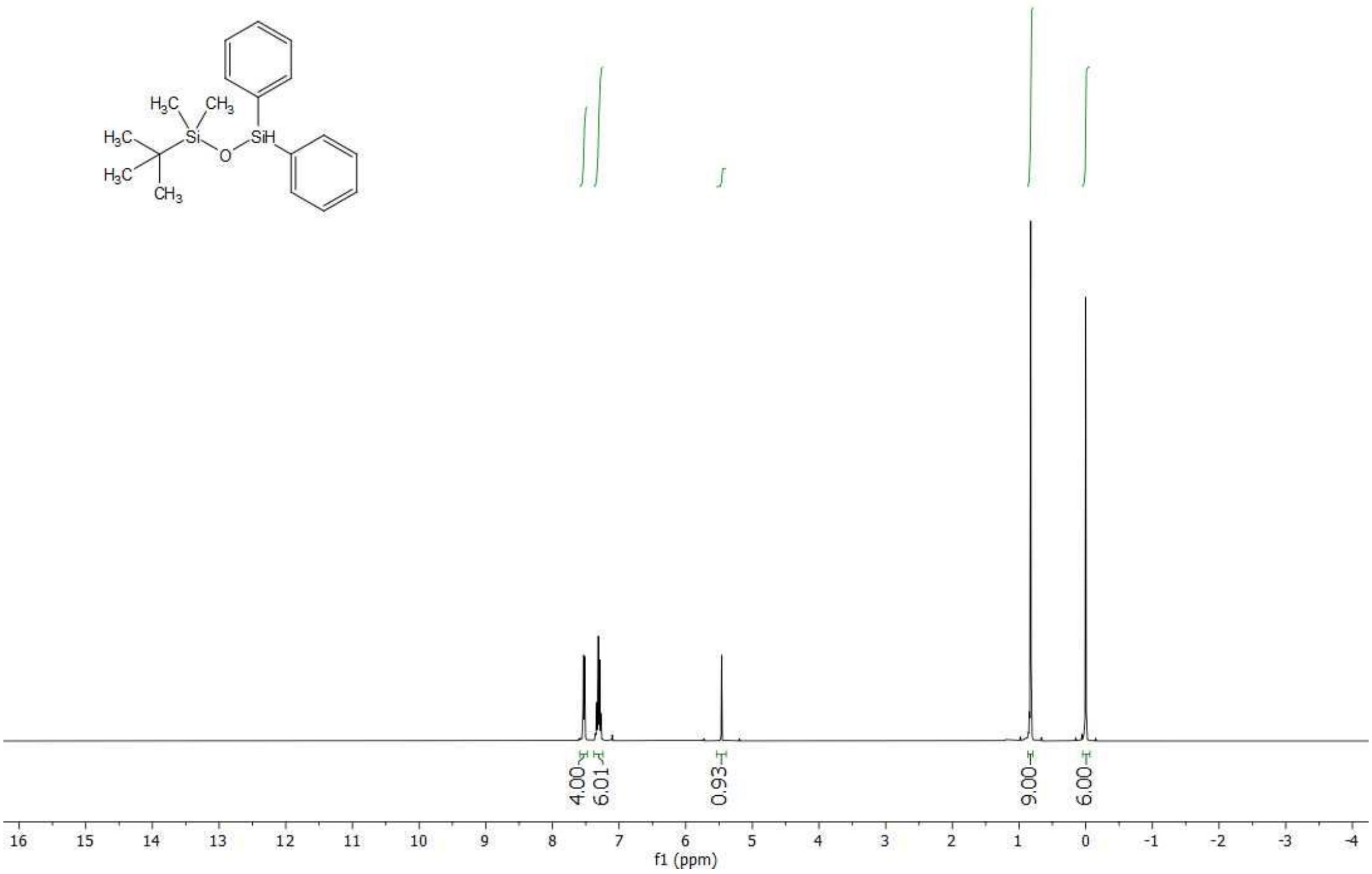

Figure SS23. ^{1}H NMR Spectrum of **6** (400 MHz, $CDCl_3$)

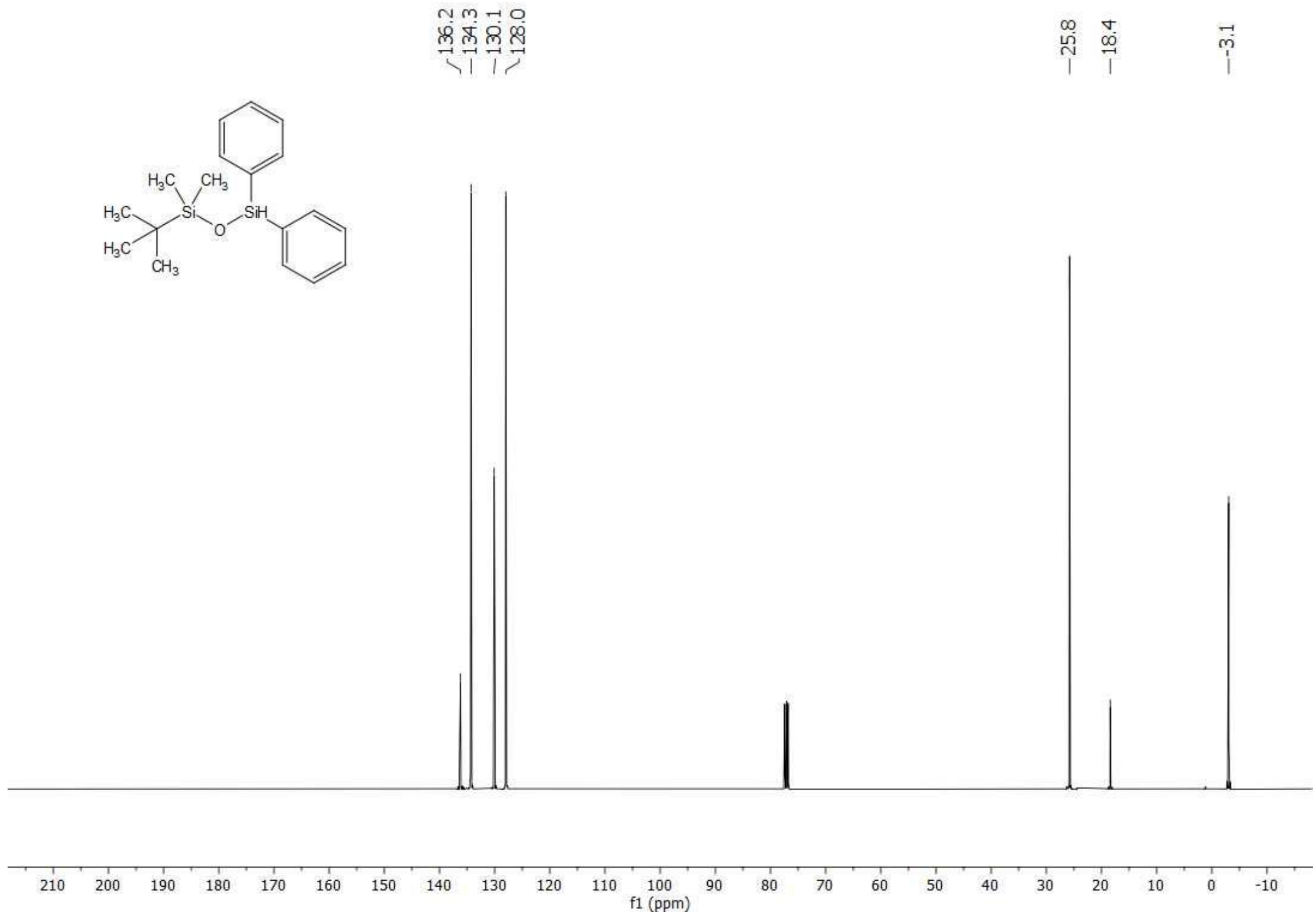

Figure SS24. ^{13}C NMR Spectrum of **6** (101 MHz, $CDCl_3$)

1-(tert-butyl)-1,1,3-trimethyl-3-phenyldisiloxane (7)

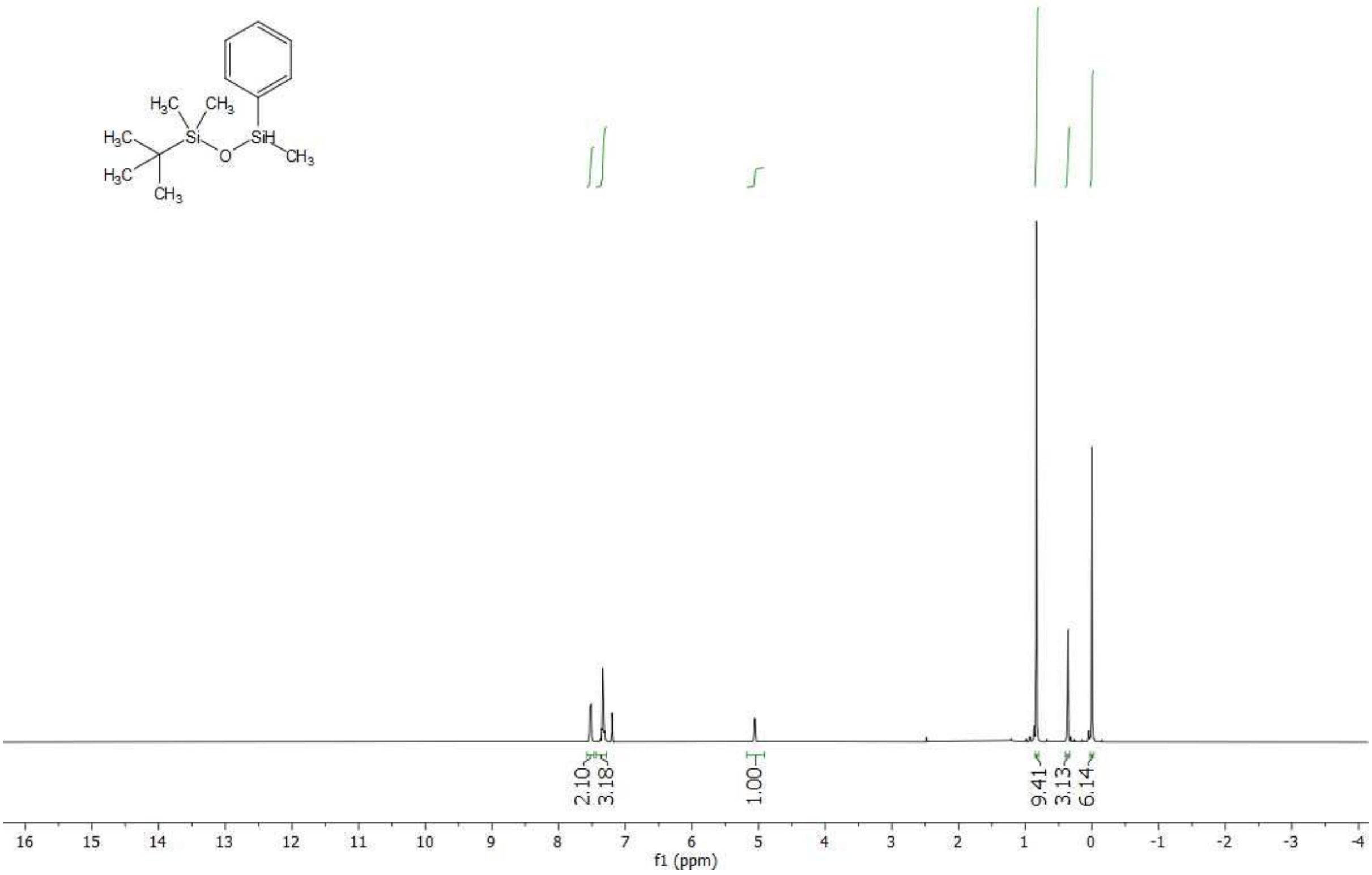

Figure SS25. ^{1}H NMR Spectrum of **7** (400 MHz, $CDCl_3$)

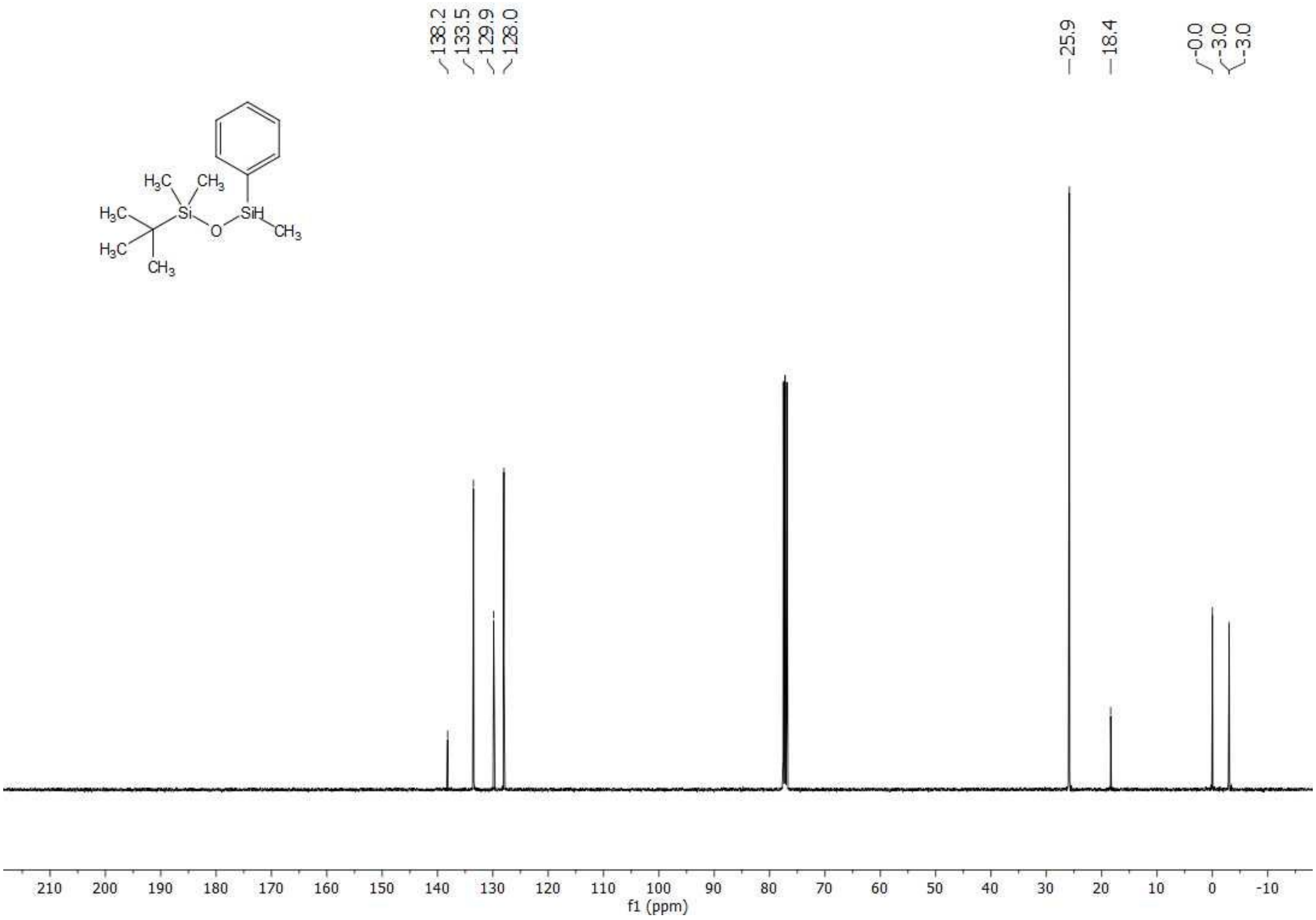

Figure SS26. ^{13}C NMR Spectrum of **7** (101 MHz, $CDCl_3$)

1-(tert-butyl)-3,3-bis(4-fluorophenyl)-1,1-dimethyldisiloxane (8)

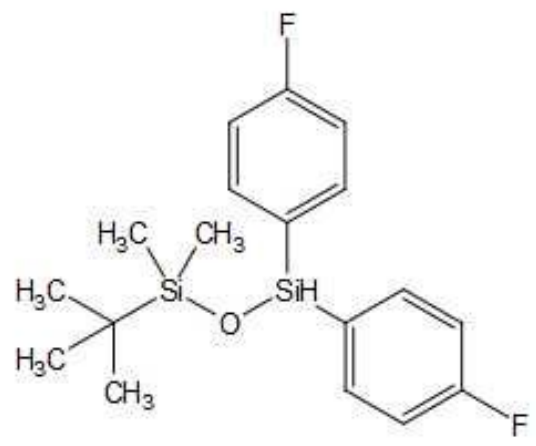

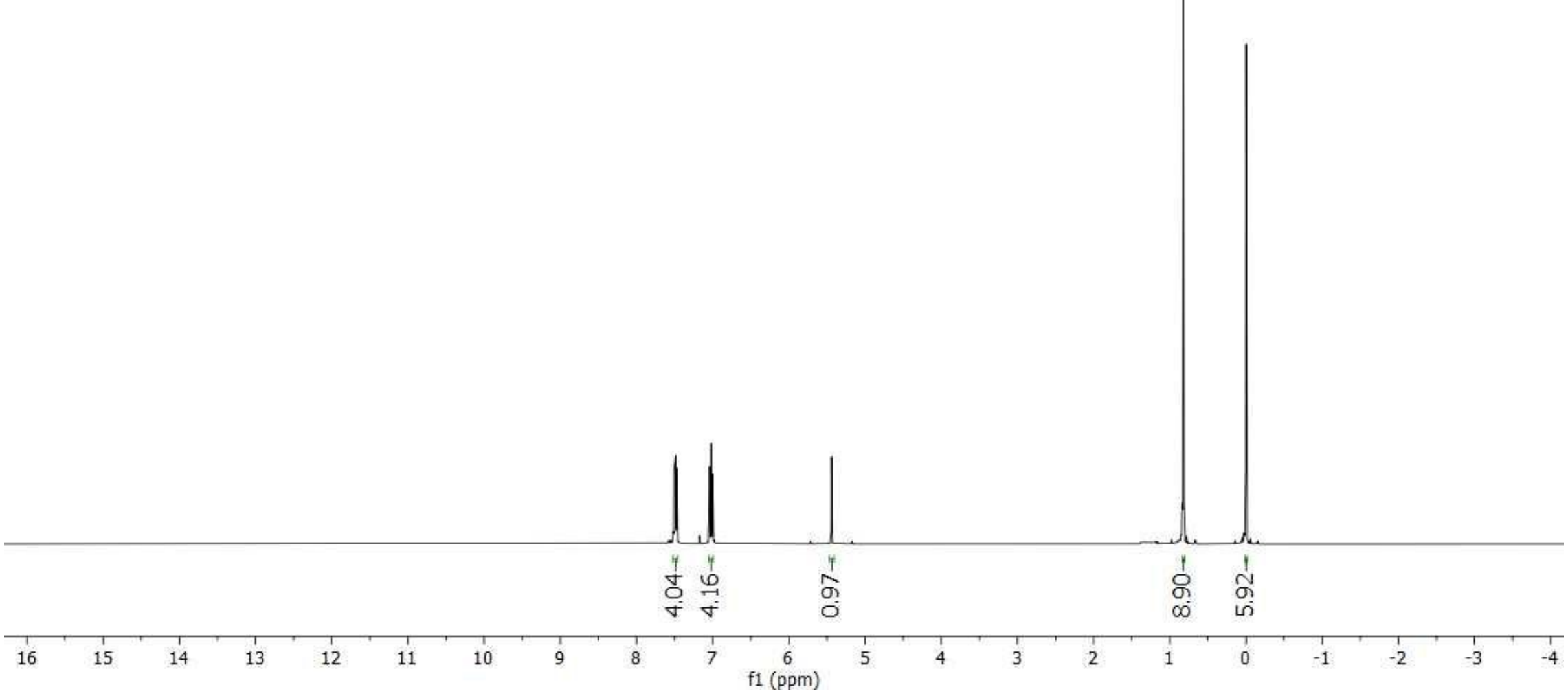

Figure SS27. ^{1}H NMR Spectrum of **8** (400 MHz, $CDCl_3$)

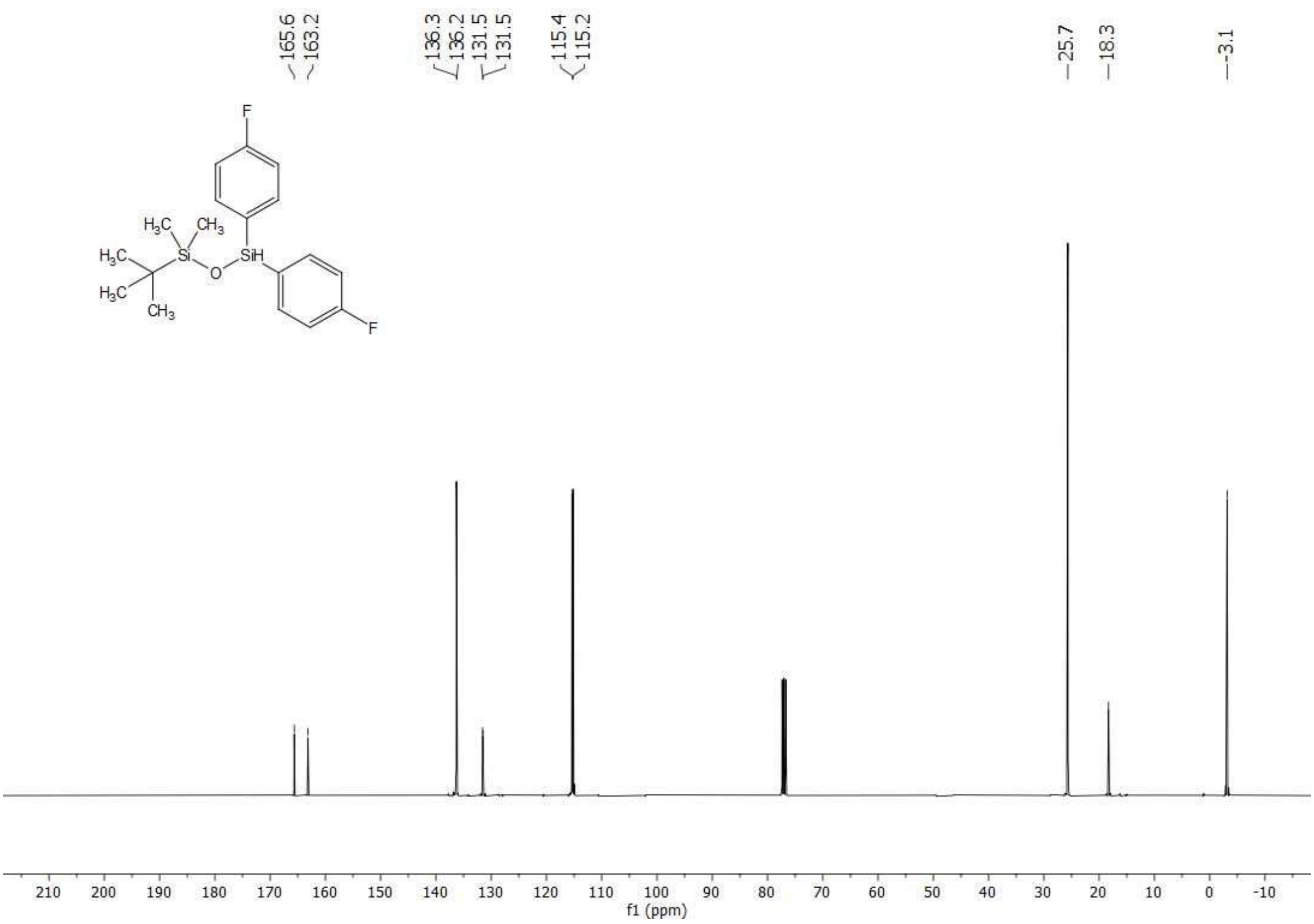

Figure SS28. ^{13}C NMR Spectrum of **8** (101 MHz, $CDCl_3$)

1-(tert-butyl)-3-hexyl-1,1-dimethyldisiloxane (9)

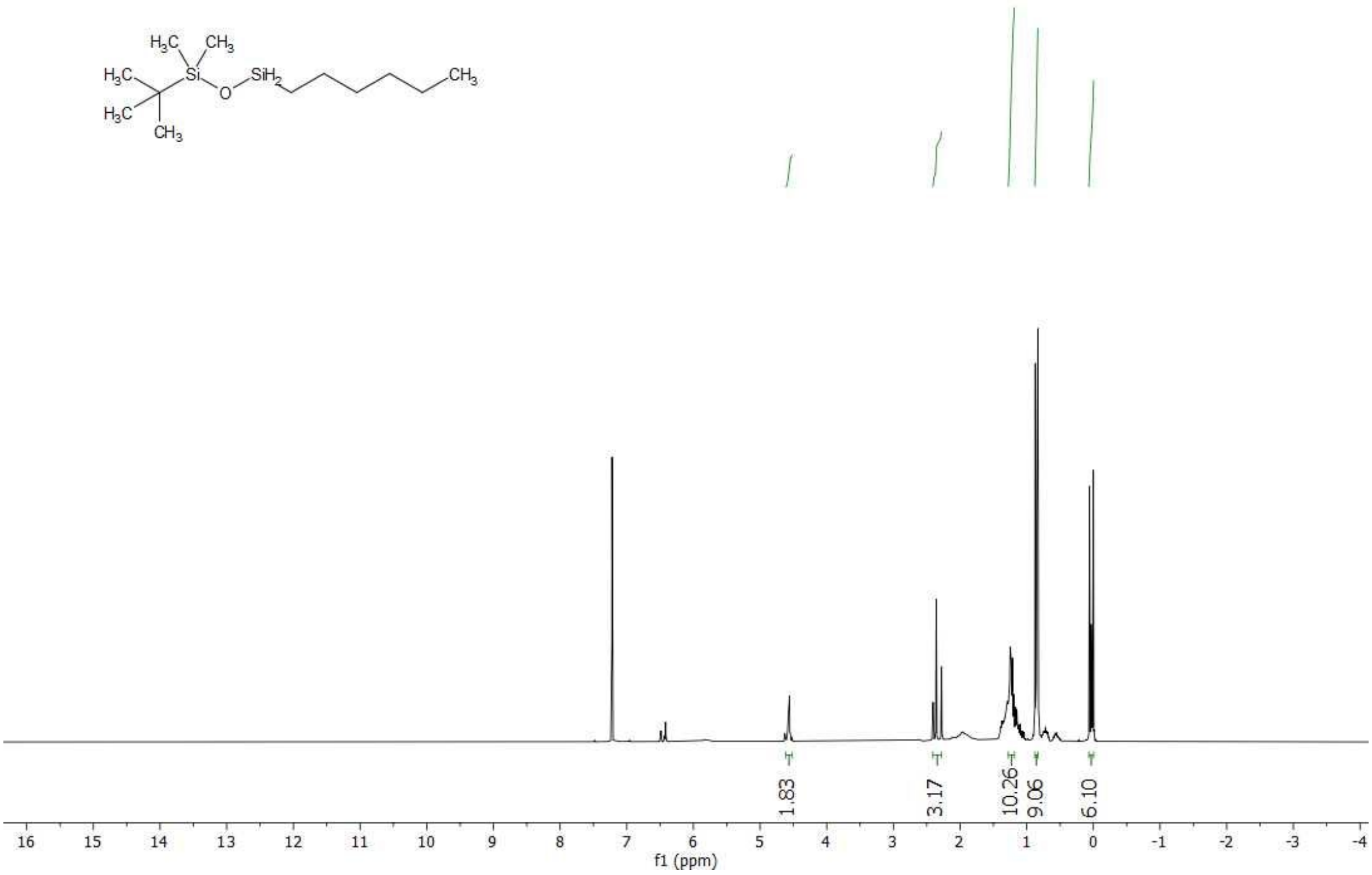

Figure SS29. ^{1}H NMR Spectrum of **9** (400 MHz, $CDCl_3$)

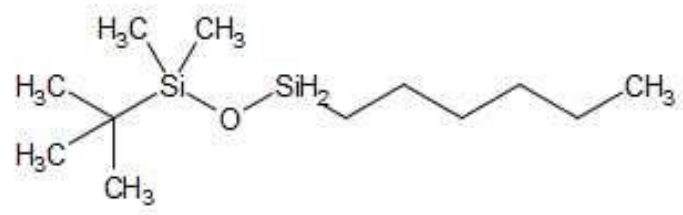

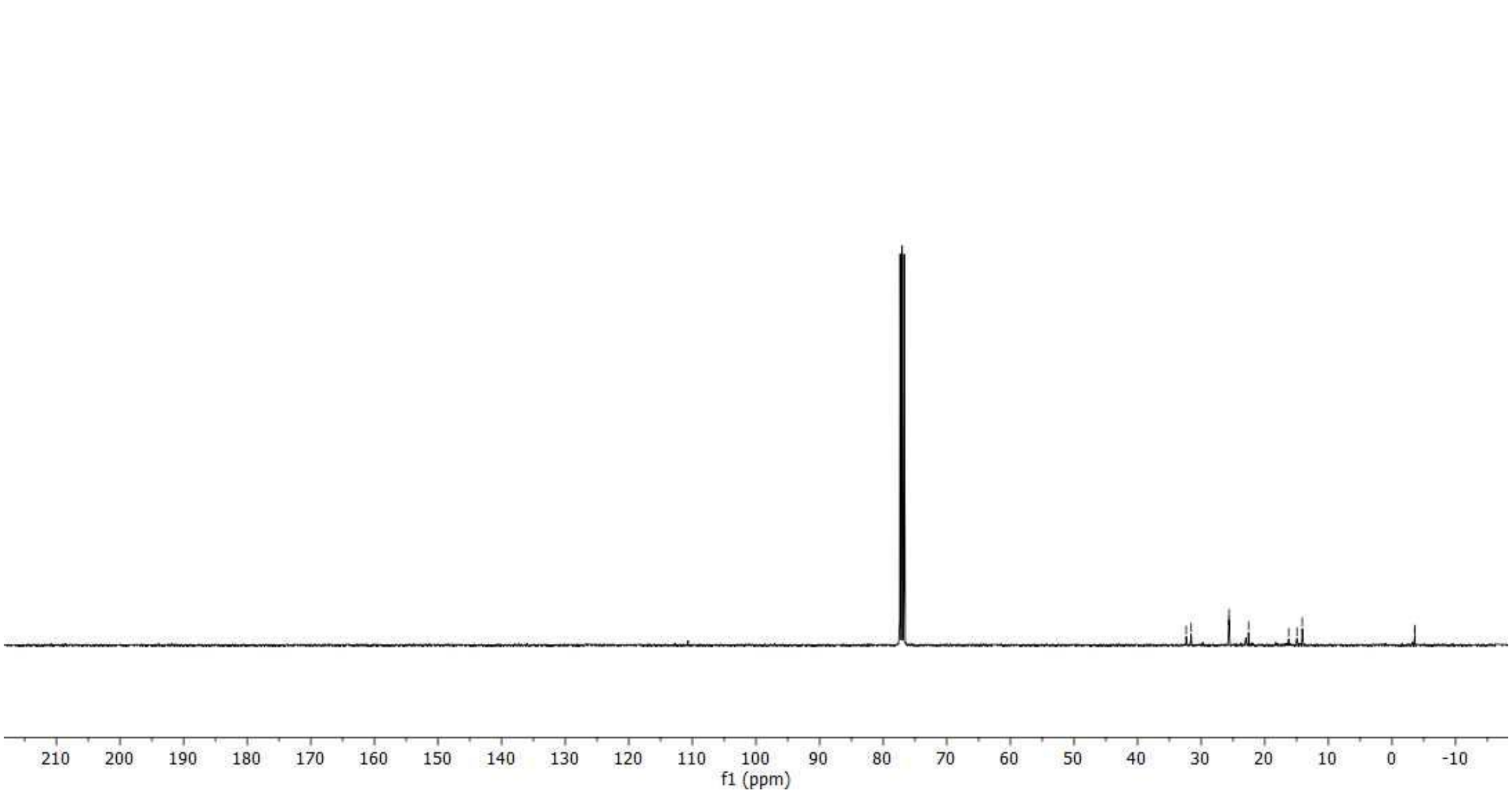

Figure SS30. ^{13}C NMR Spectrum of **9** (101 MHz, $CDCl_3$)

1,1,1,5,5,5-hexaisobutyl-3-phenyltrisiloxane (10)

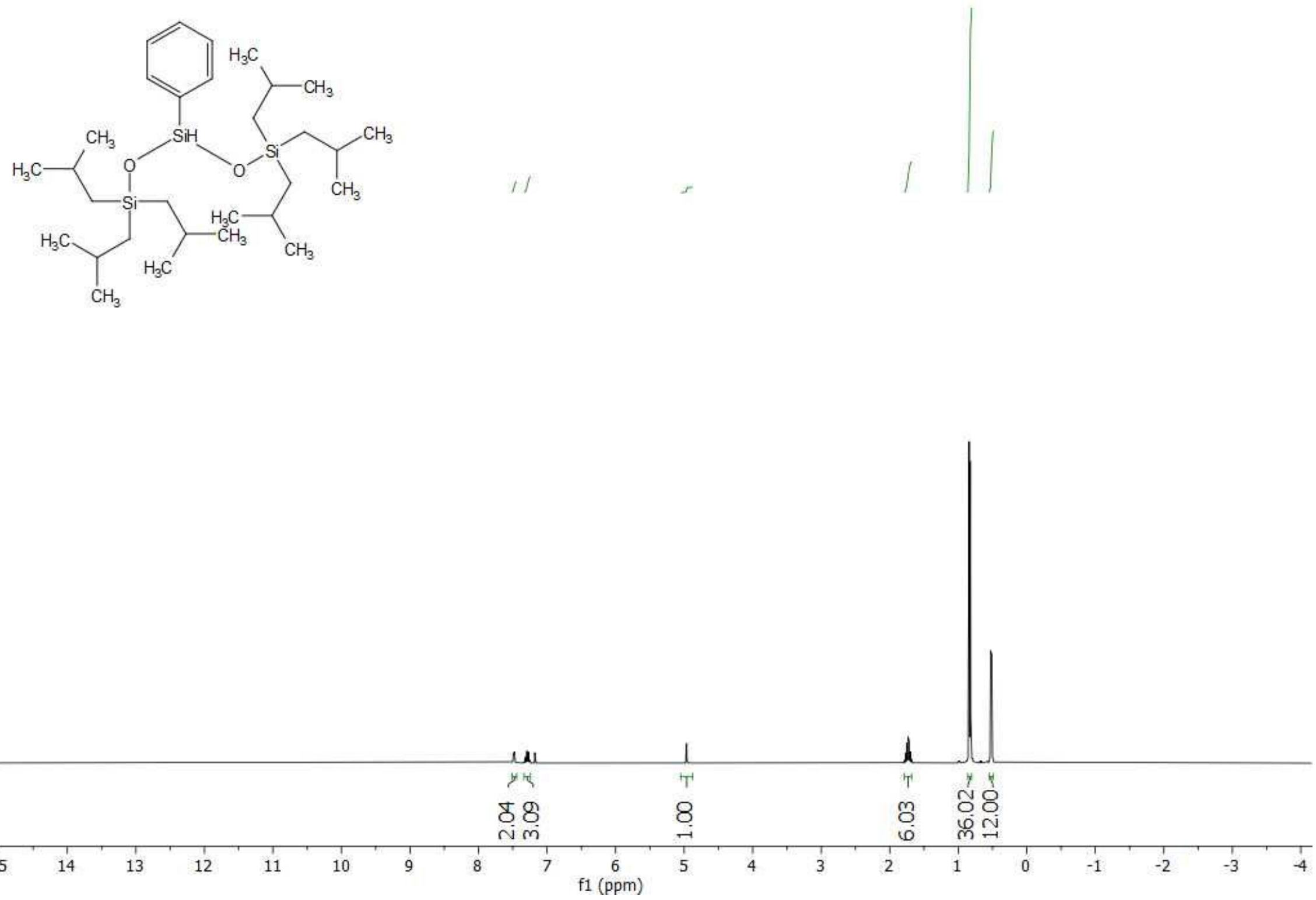

Figure SS31. ^1H NMR Spectrum of **10** (400 MHz, $CDCl_3$)

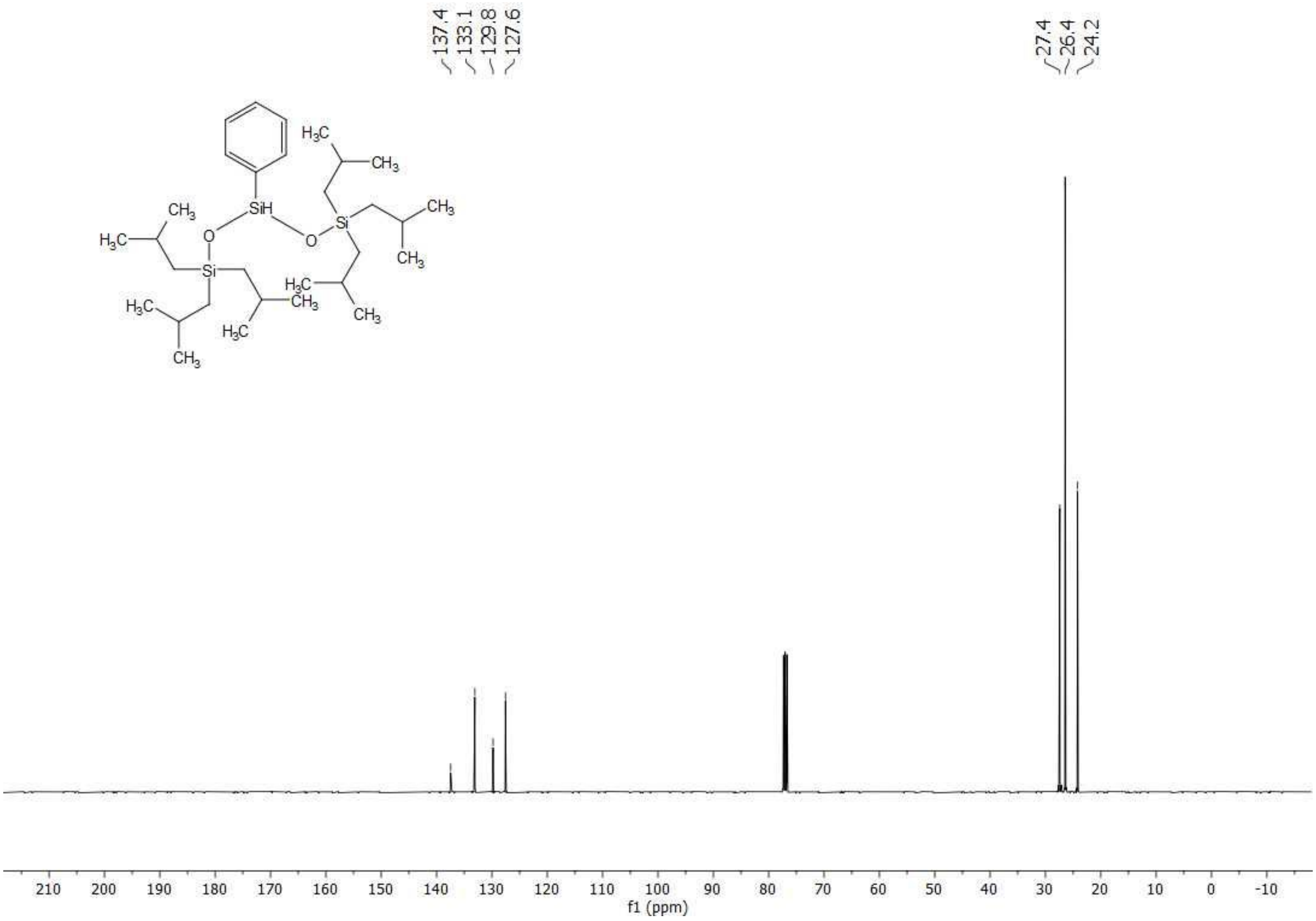

Figure SS32. ^{13}C NMR Spectrum of **10** (101 MHz, $CDCl_3$)

1,1,5,5-tetramethyl-1,3,5-triphenyltrisiloxane (11)

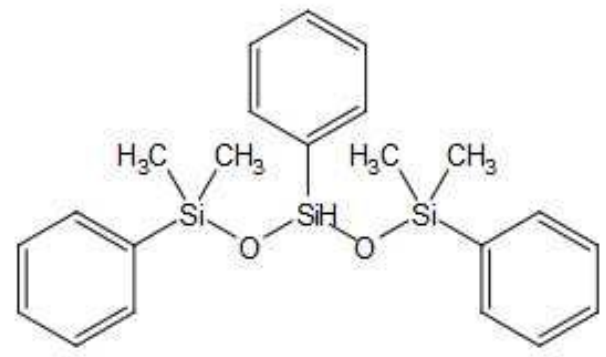

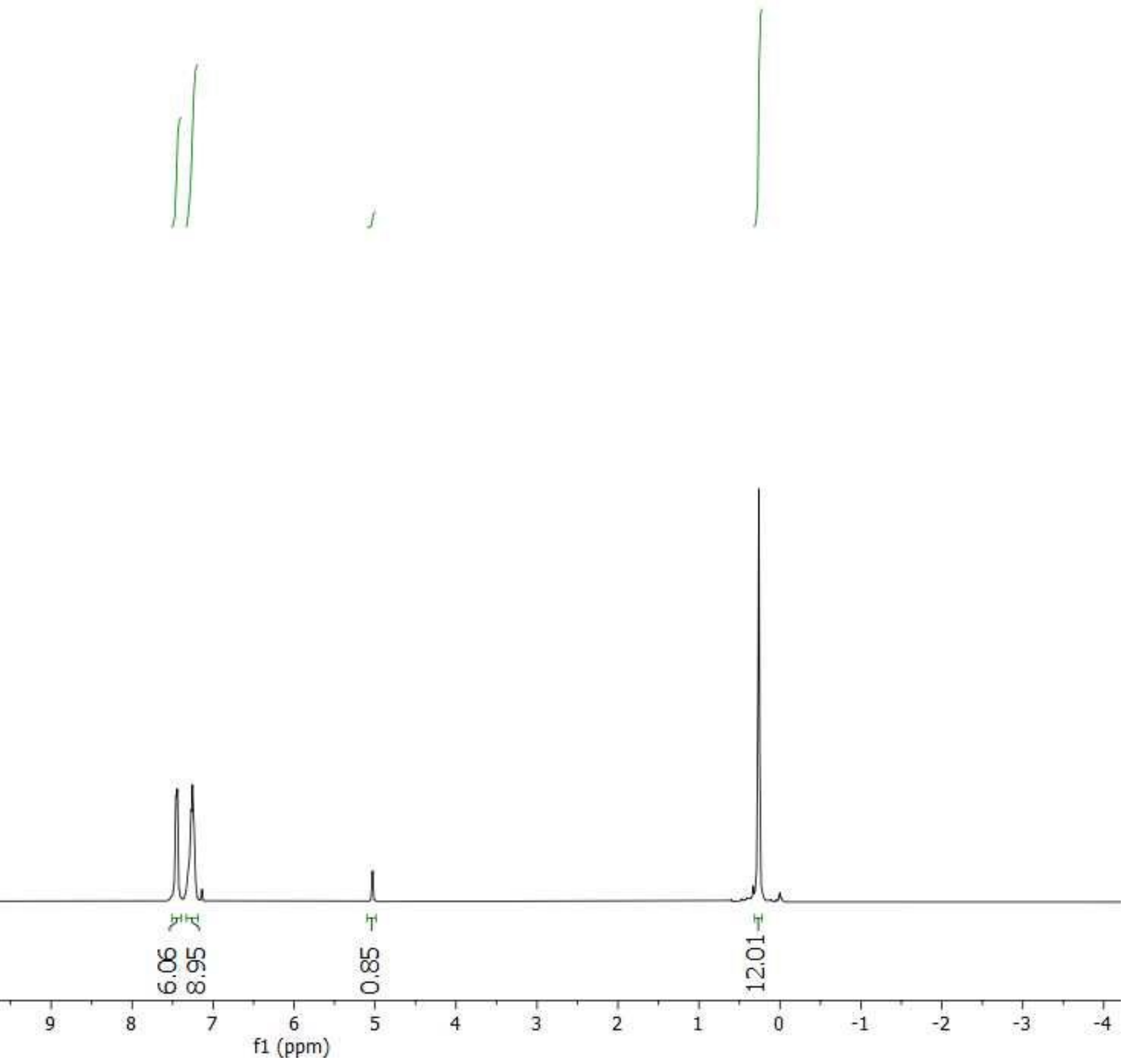

Figure SS33. ^{1}H NMR Spectrum of **11** (400 MHz, $CDCl_3$)

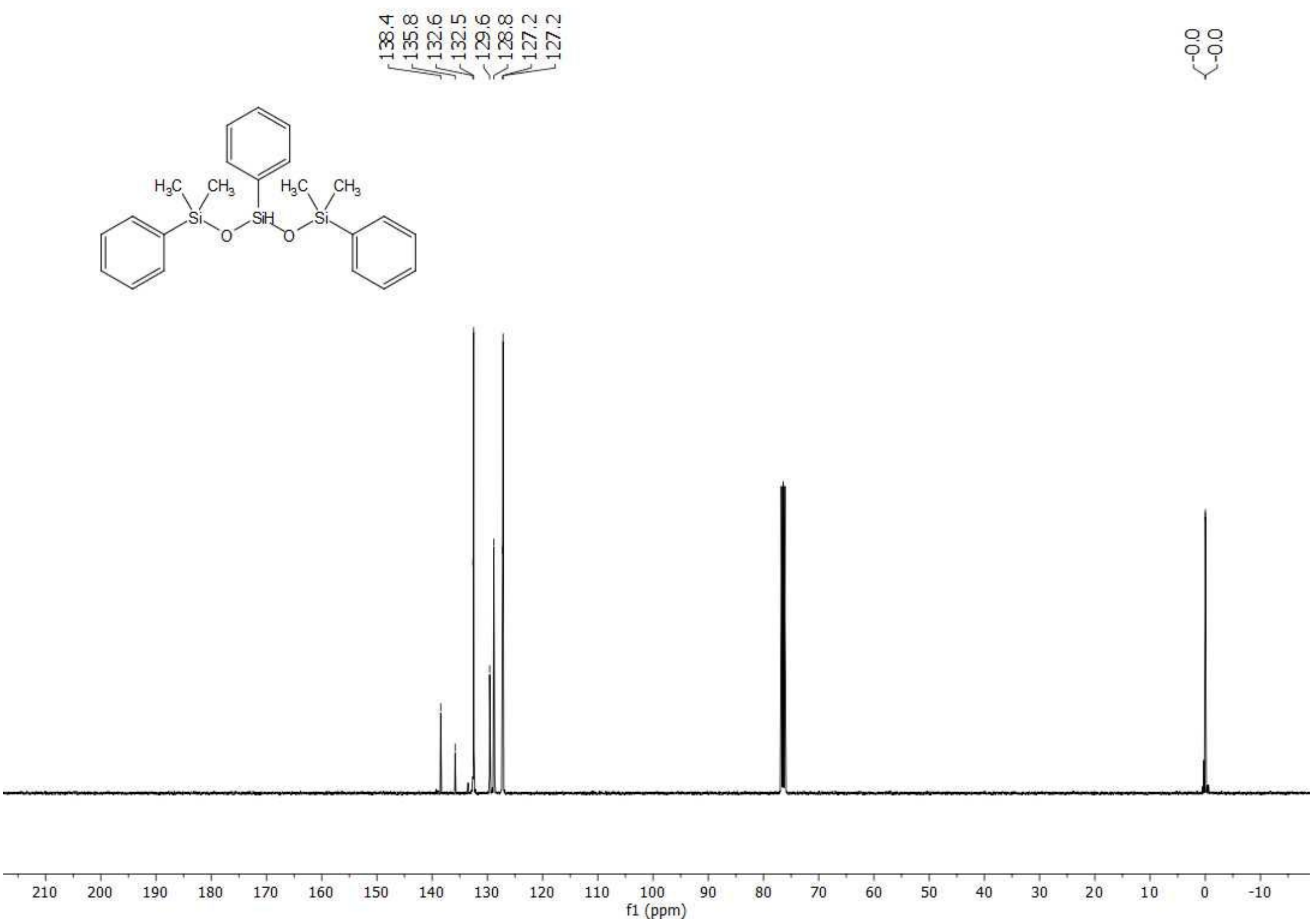

Figure SS34. ^{13}C NMR Spectrum of **11** (101 MHz, $CDCl_3$)

1,1,1,5,5,5-hexamethyl-3-phenyltrisiloxane (12)

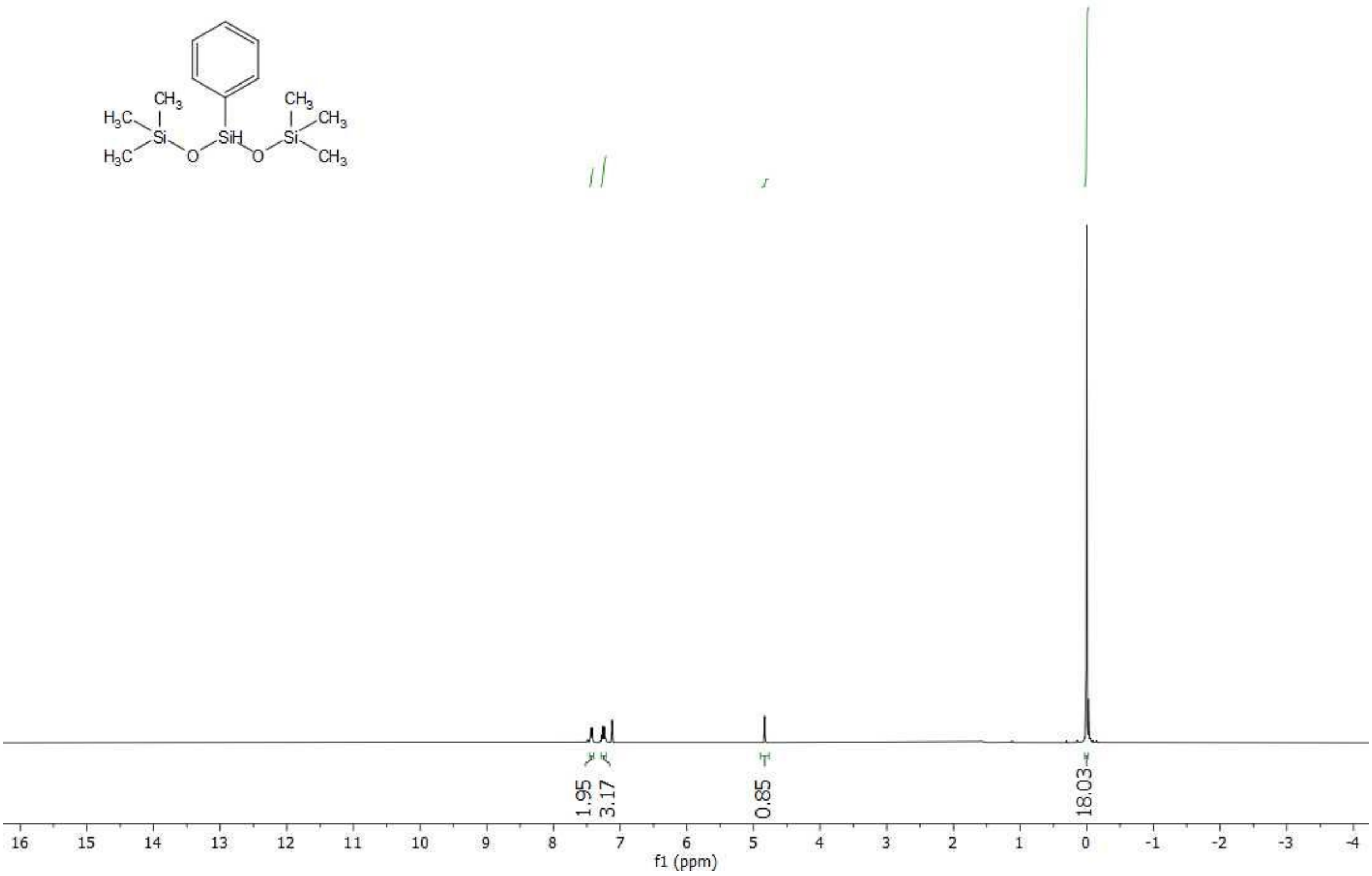

Figure SS35. ^{1}H NMR Spectrum of **12** (400 MHz, $CDCl_3$)

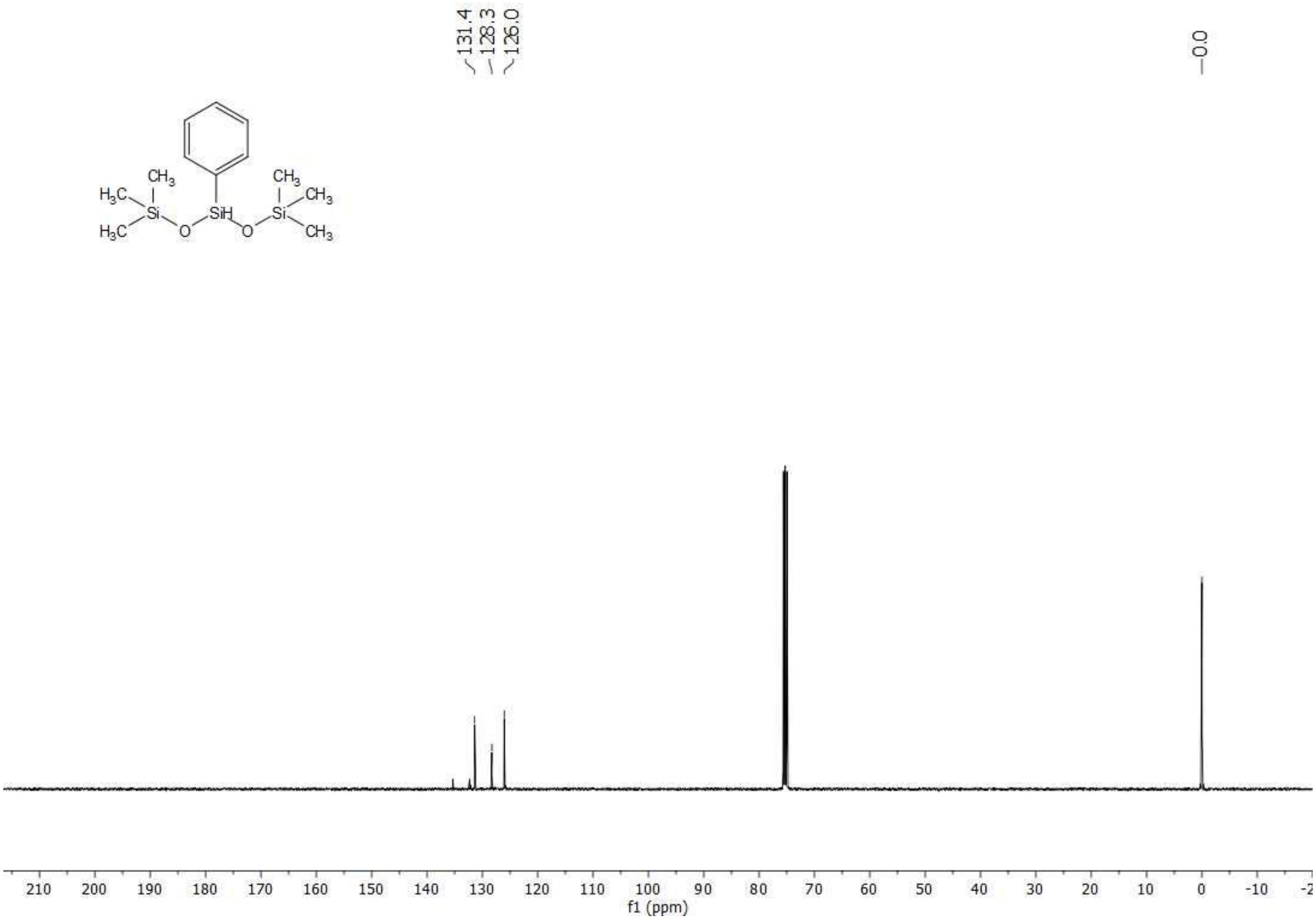

Figure SS36. ^{13}C NMR Spectrum of **12** (101 MHz, $CDCl_3$)

1,1,1,5,5,5-hexaethyl-3-phenyltrisiloxane (13)

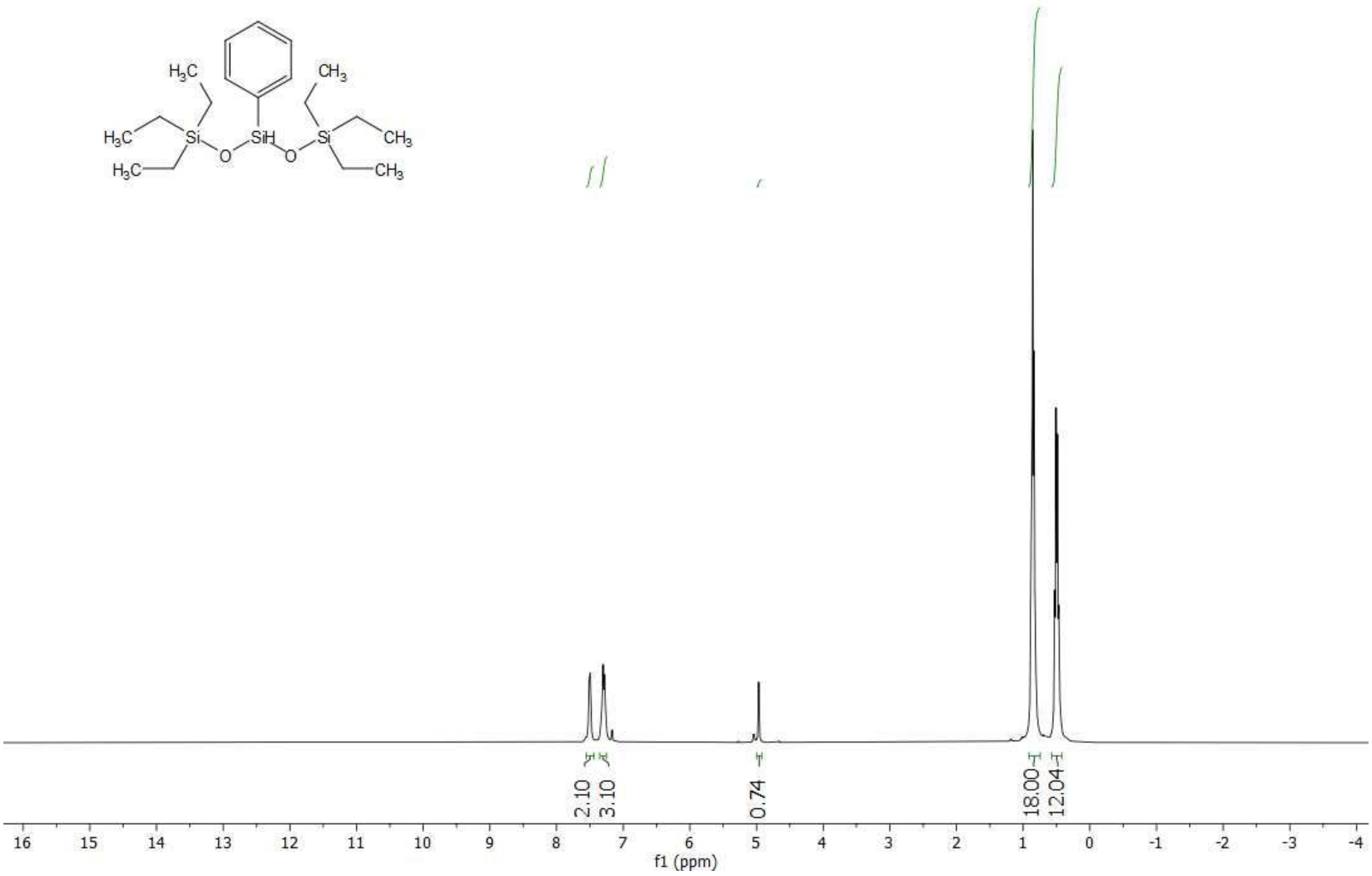

Figure SS37. ^{1}H NMR Spectrum of **13** (400 MHz, $CDCl_3$)

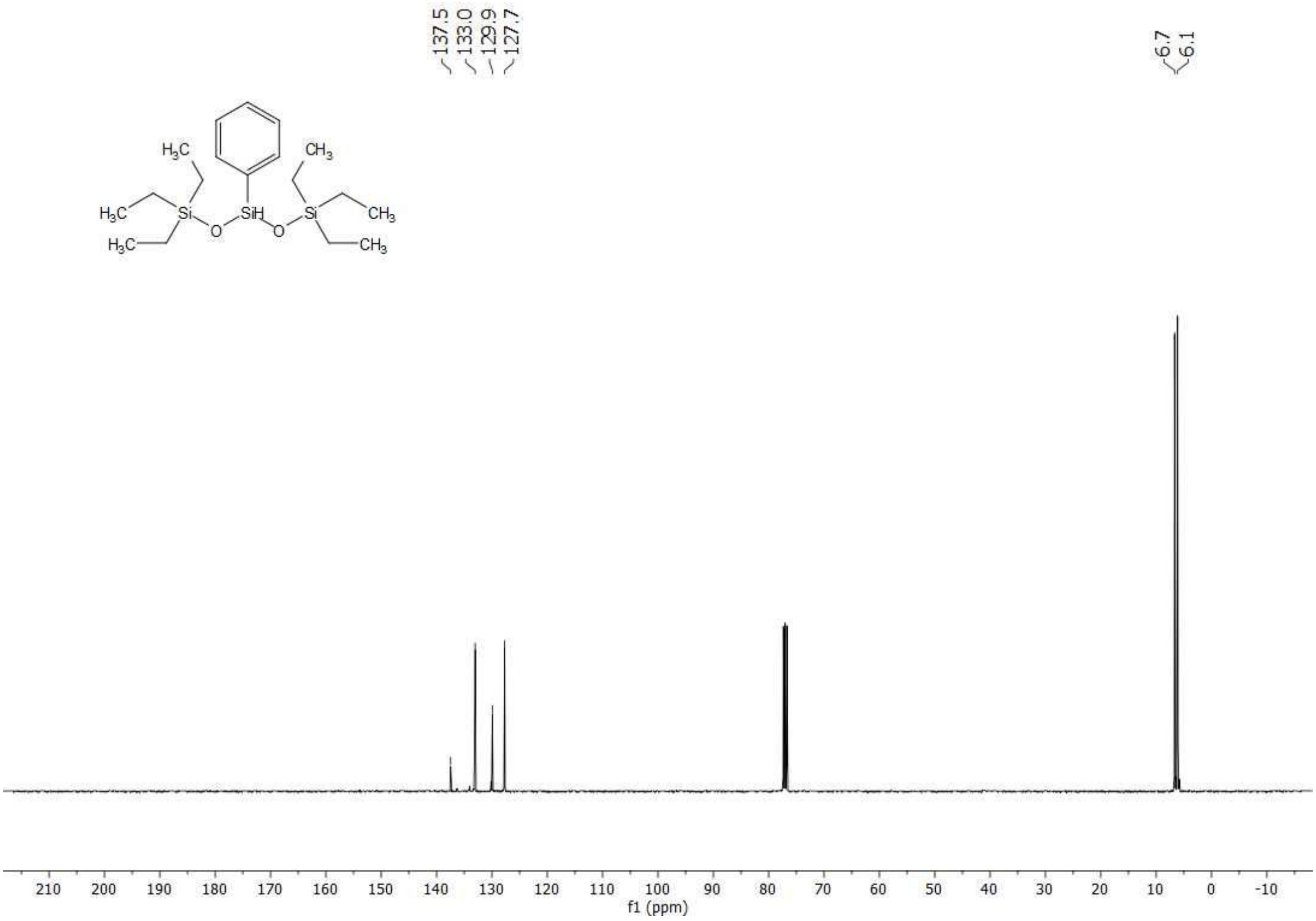

Figure SS38. ^{13}C NMR Spectrum of **13** (101 MHz, $CDCl_3$)

1,5-di-tert-butyl-1,1,5,5-tetramethyl-3-phenyltrisiloxane (14)

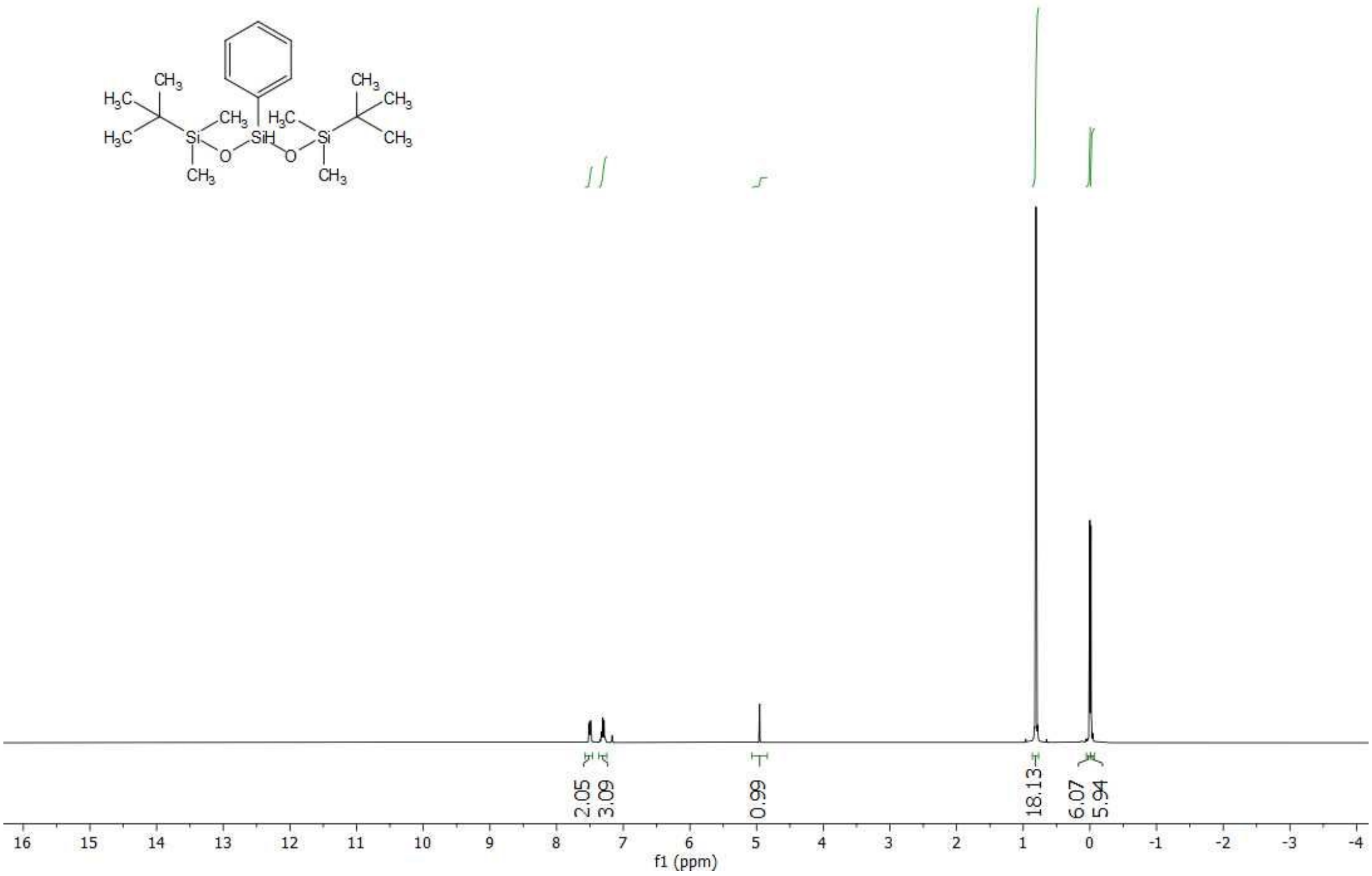

Figure SS39. ^{1}H NMR Spectrum of **14** (400 MHz, $CDCl_3$)

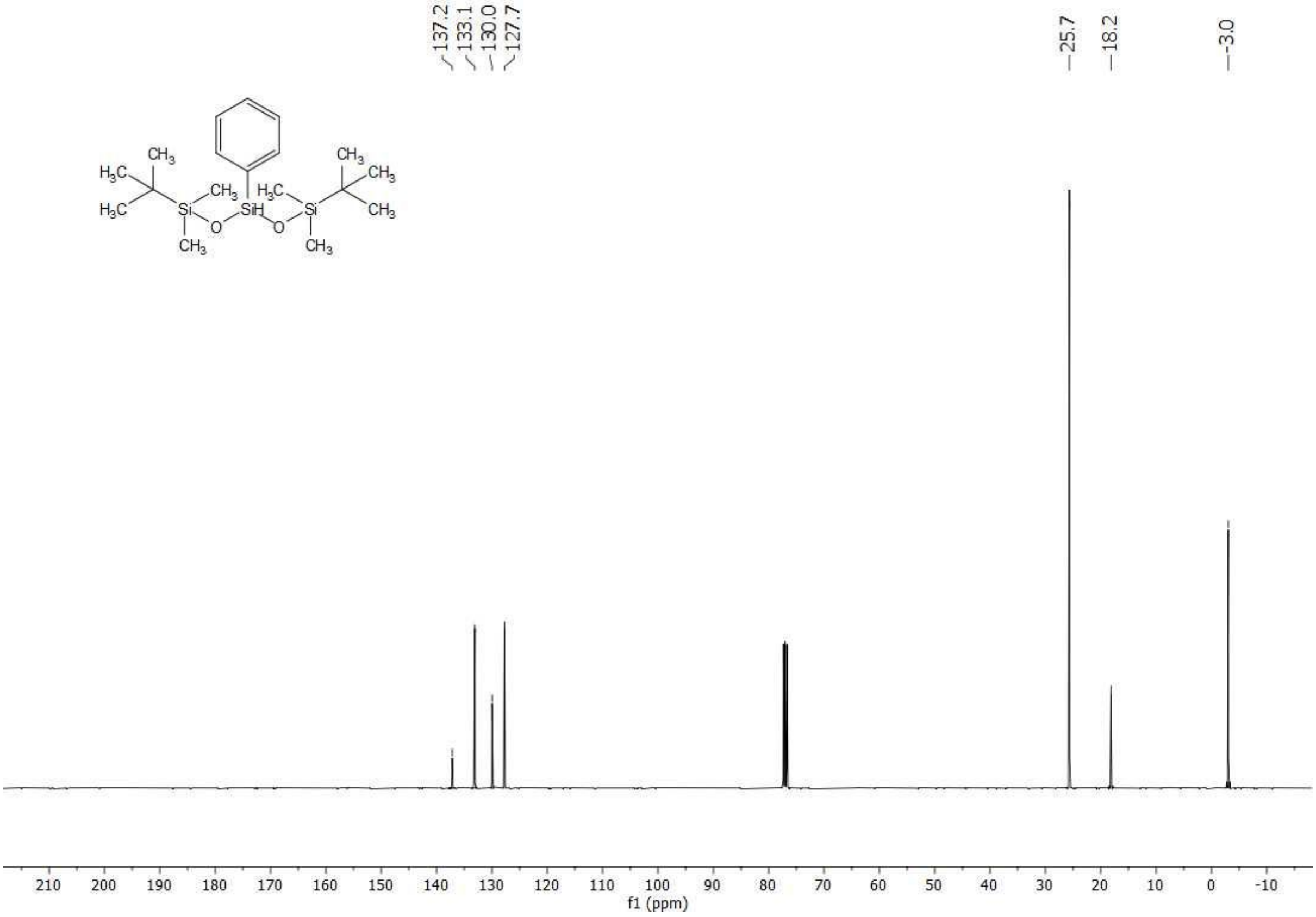

Figure SS40. ^{13}C NMR Spectrum of **14** (101 MHz, $CDCl_3$)

1,1,1,5,5,5-hexaisopropyl-3-phenyltrisiloxane (15)

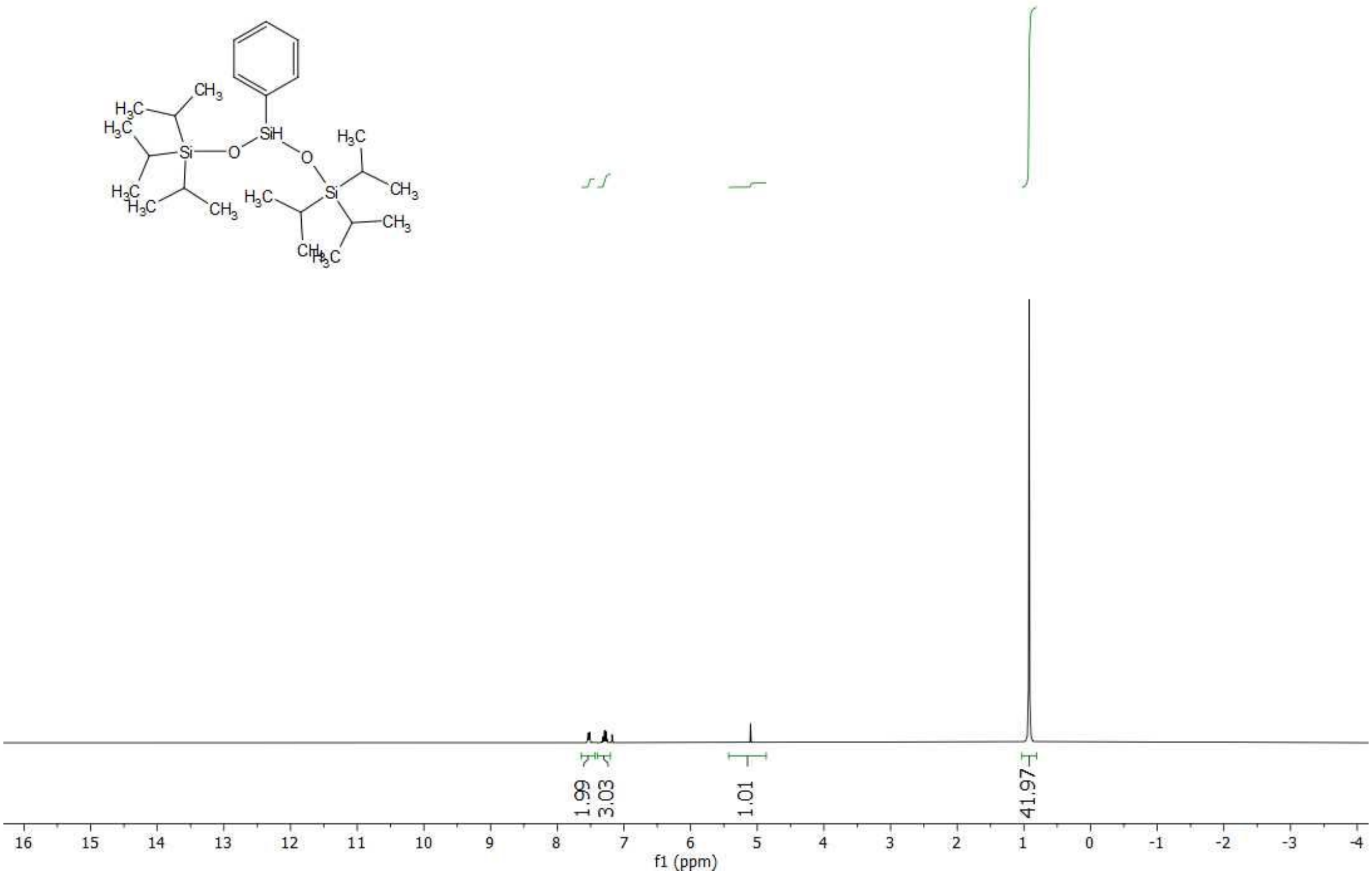

Figure SS41. ^{1}H NMR Spectrum of **15** (400 MHz, $CDCl_3$)

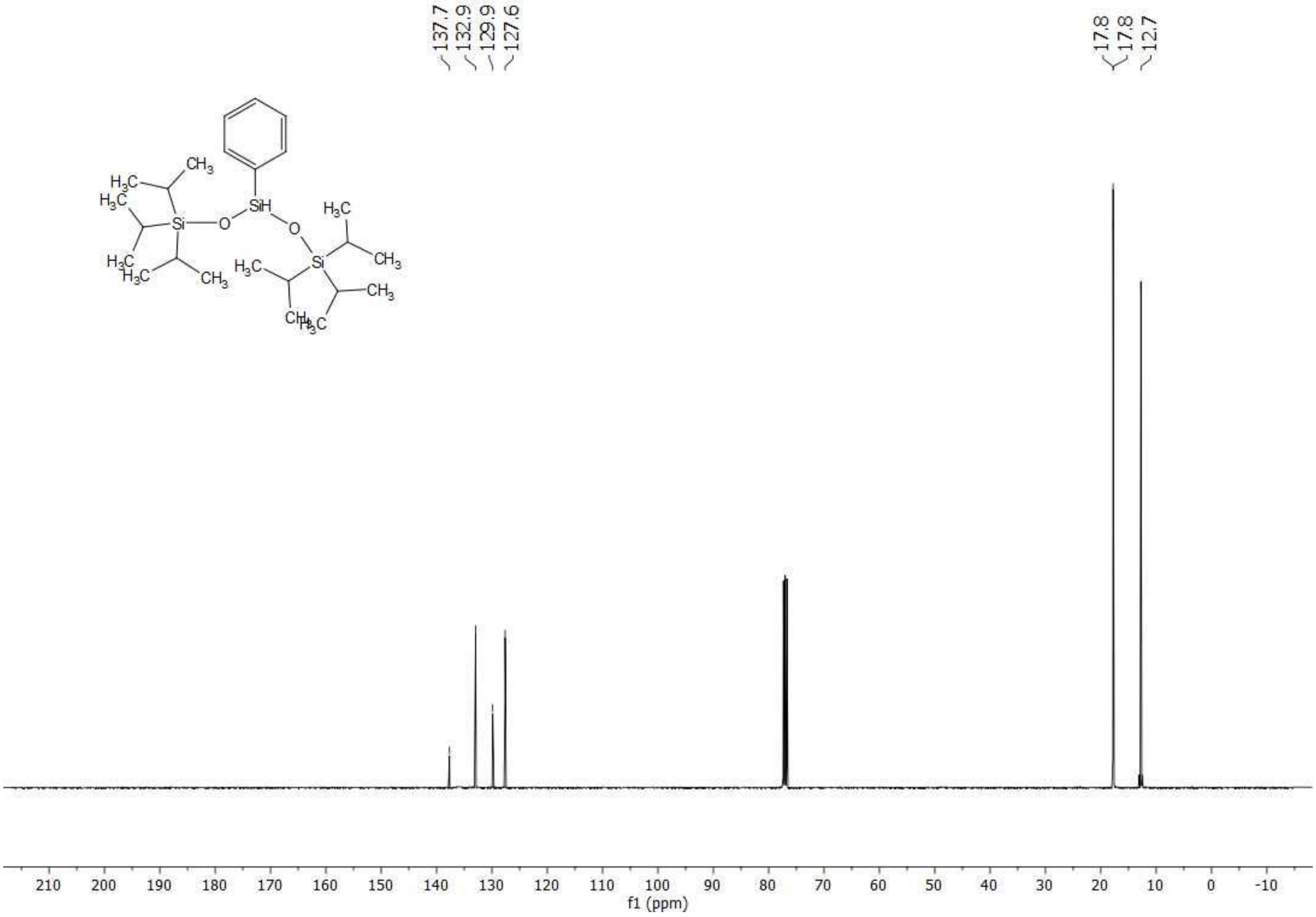

Figure SS42. ^{13}C NMR Spectrum of **15** (101 MHz, $CDCl_3$)

3-phenyl-1,1,1,5,5,5-hexapropyltrisiloxane (16)

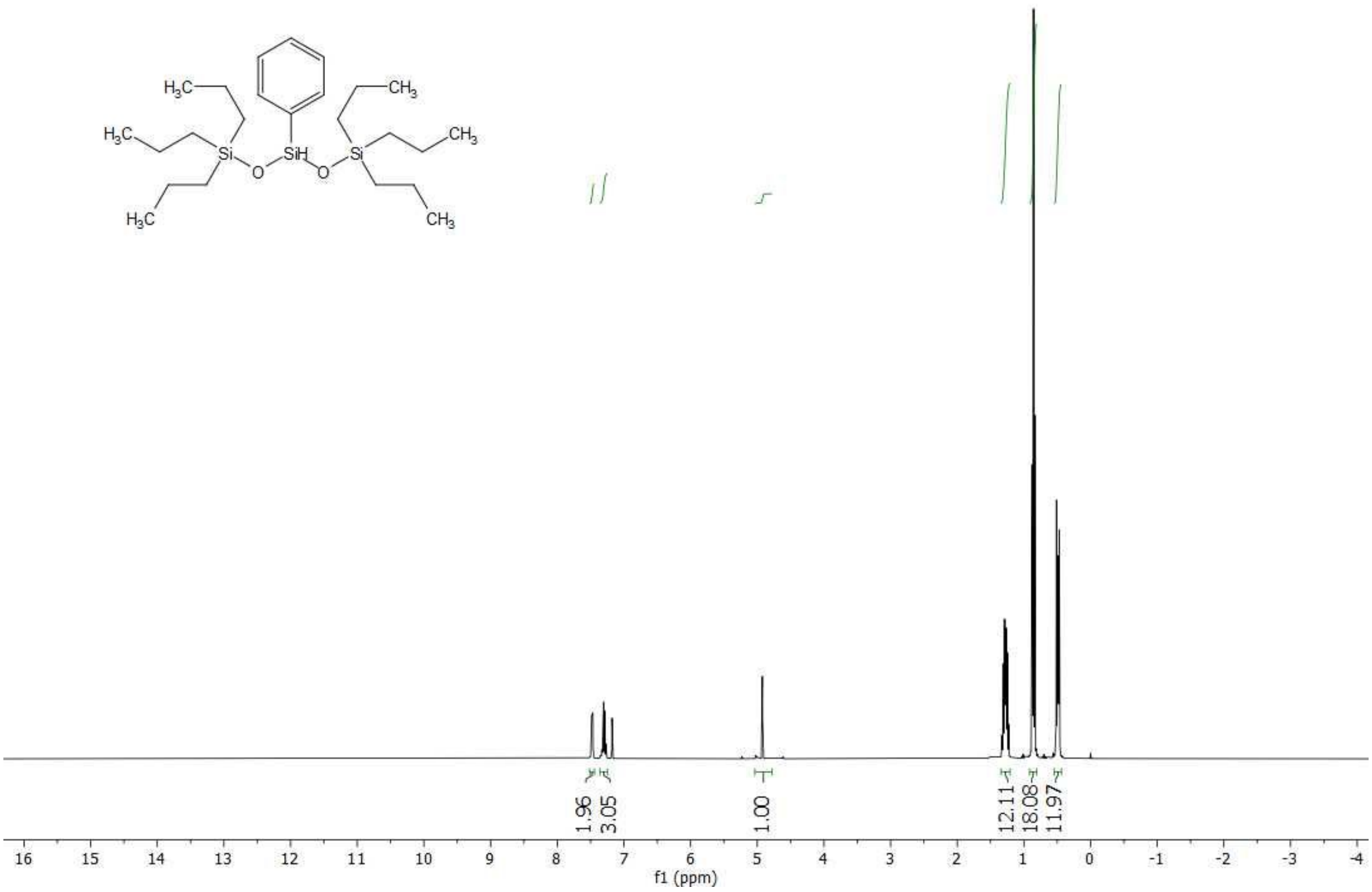

Figure SS43. ^{1}H NMR Spectrum of **16** (400 MHz, $CDCl_3$)

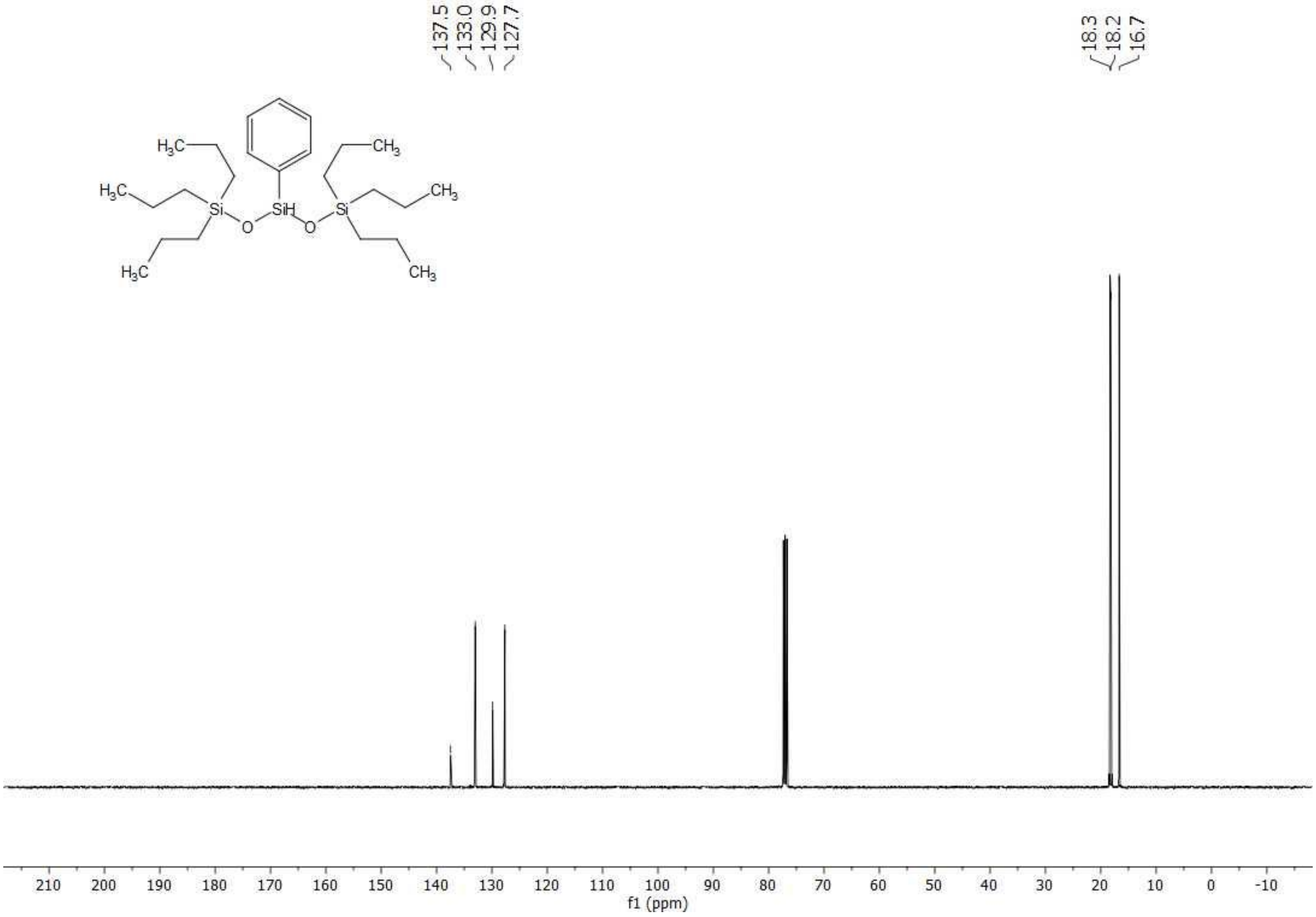

Figure SS44. ^{13}C NMR Spectrum of **16** (101 MHz, $CDCl_3$)

1,1,1,5,5,5-hexahexyl-3-phenyltrisiloxane (17)

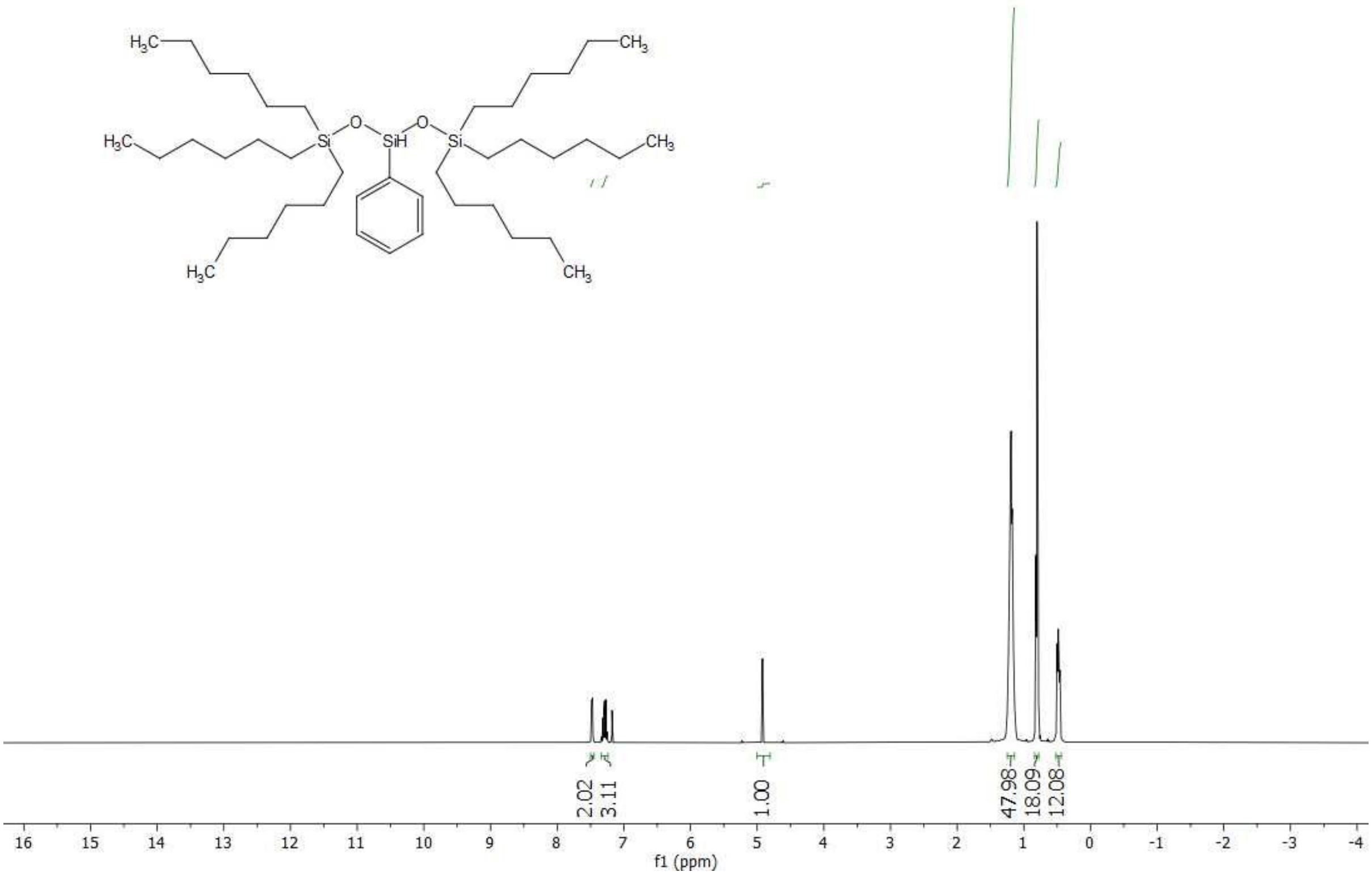

Figure SS45. ^{1}H NMR Spectrum of **17** (400 MHz, $CDCl_3$)

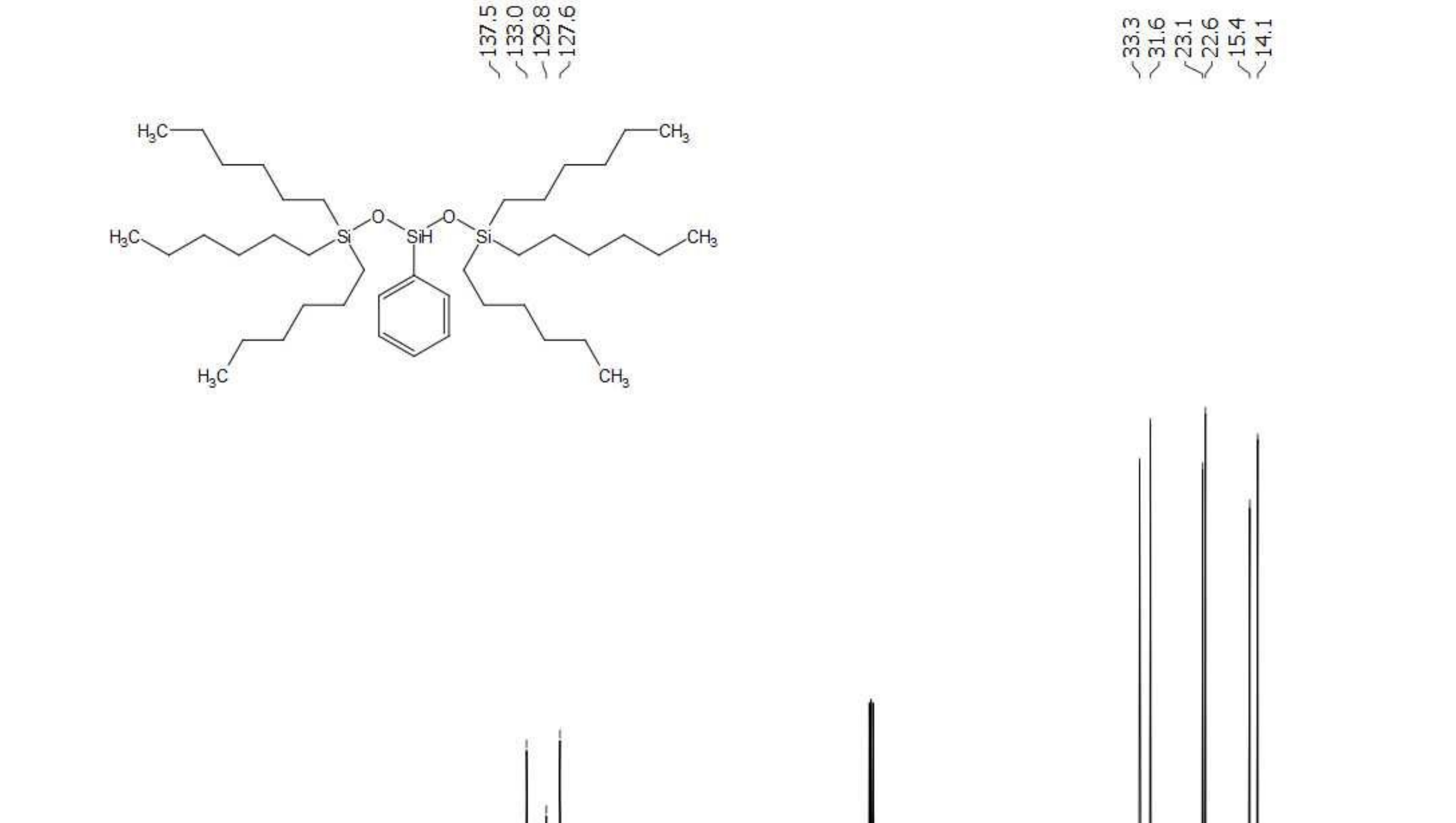

Figure SS46. ^{13}C NMR Spectrum of **17** (101 MHz, $CDCl_3$)

1,5-di-tert-butyl-1,1,3,5,5-pentaphenyltrisiloxane (18)

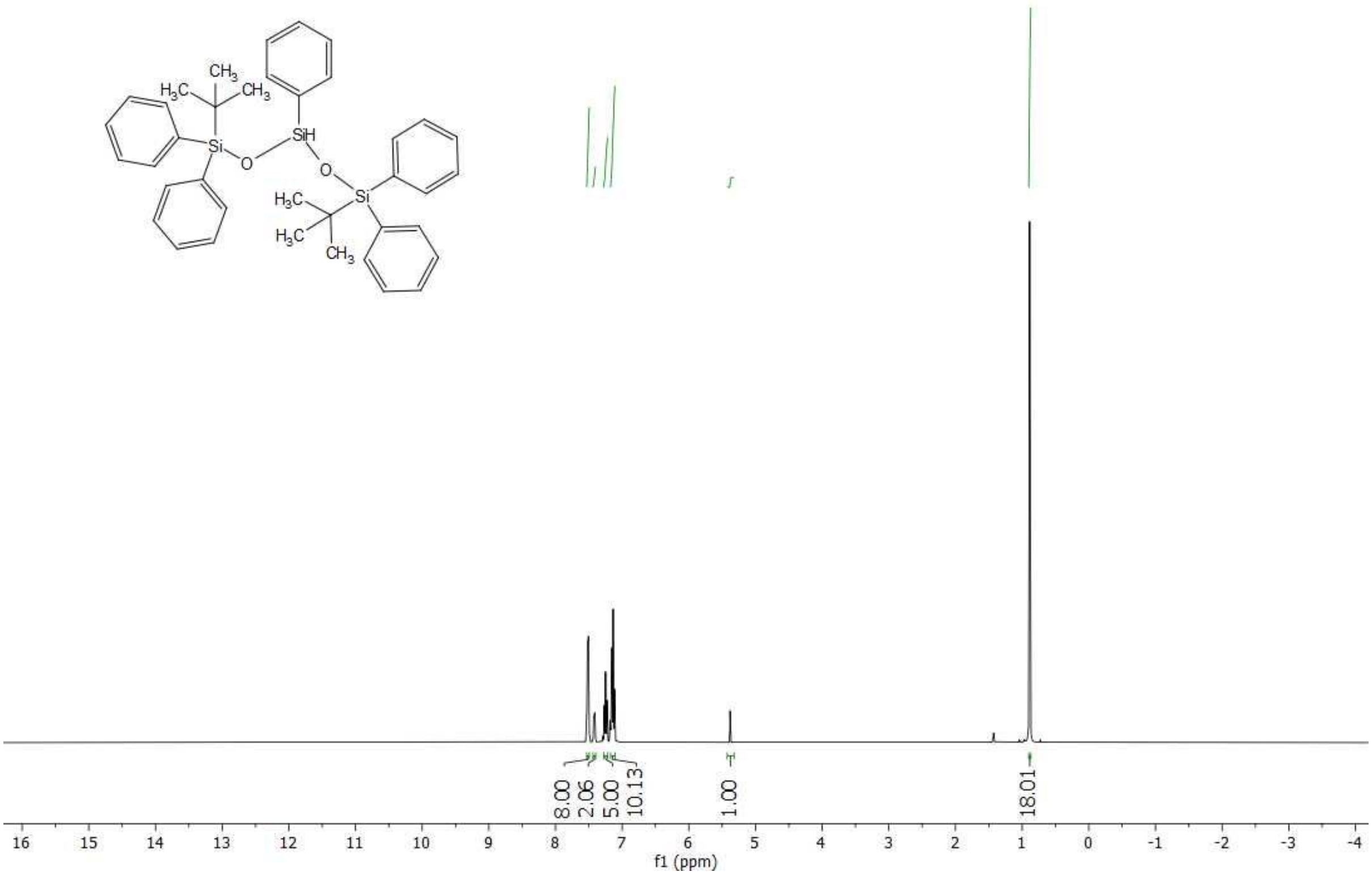

Figure SS47. ^{1}H NMR Spectrum of **18** (400 MHz, $CDCl_3$)

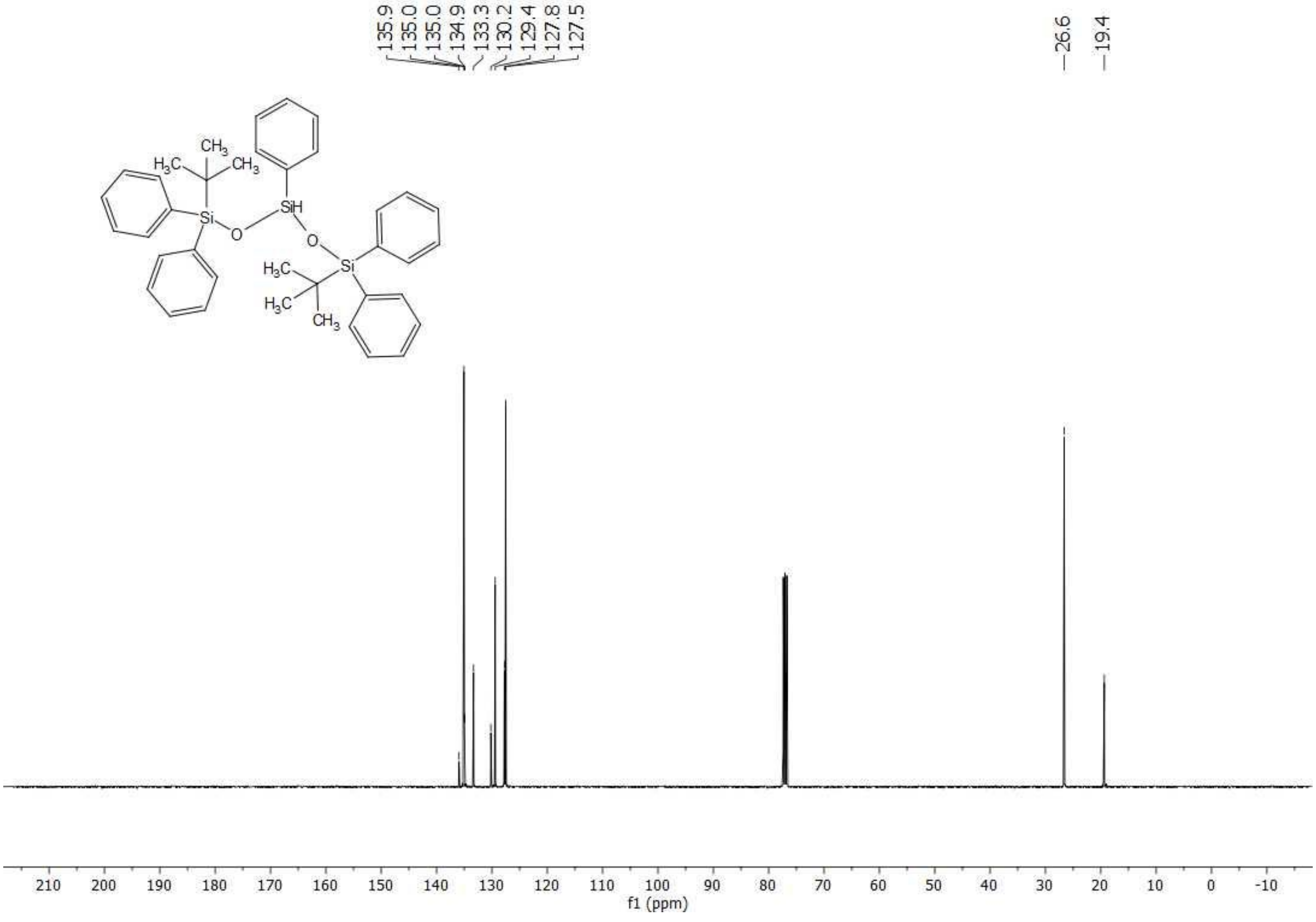

Figure SS48. ^{13}C NMR Spectrum of **18** (101 MHz, $CDCl_3$)

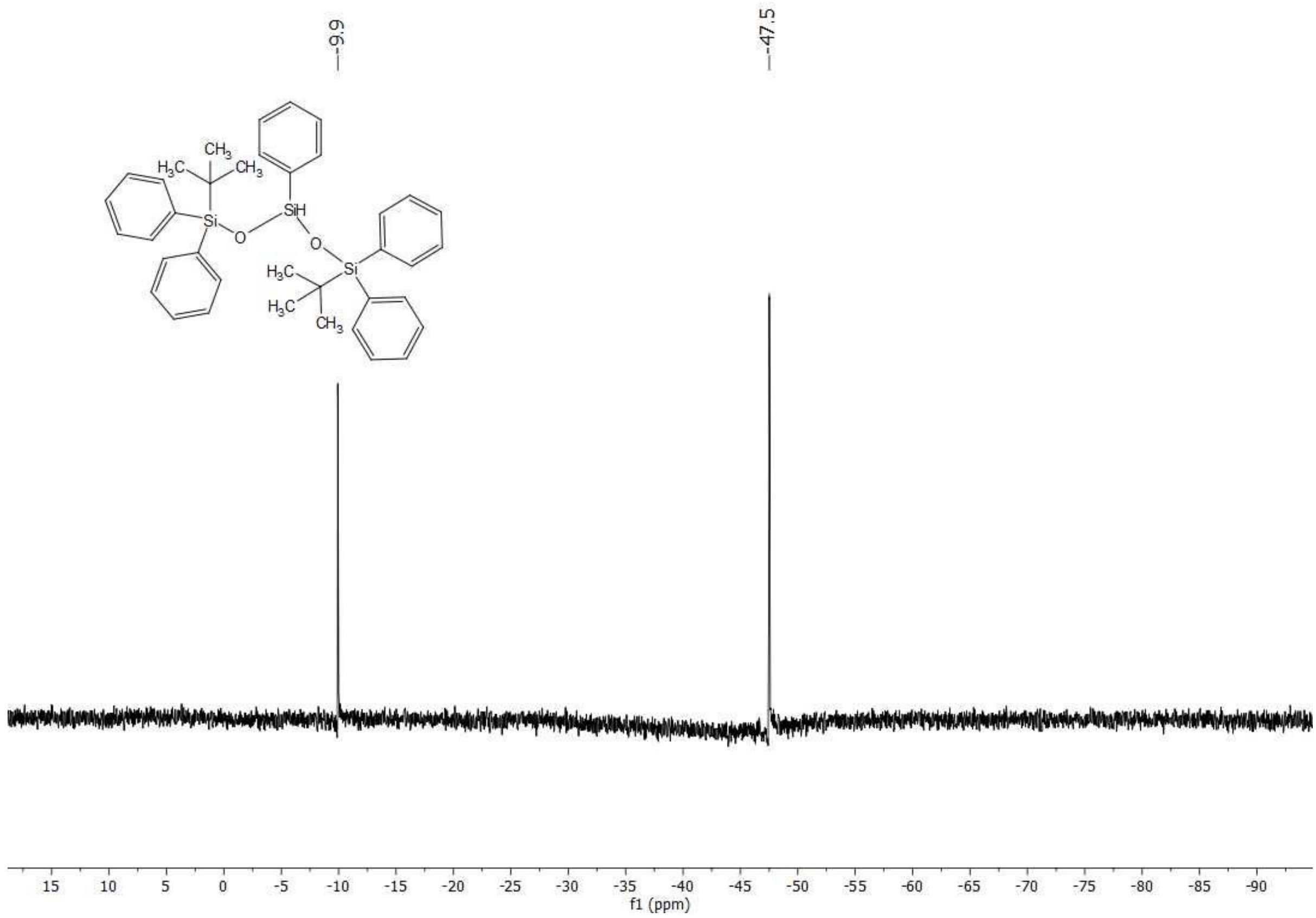

Figure SS49. ^{29}Si NMR Spectrum of **18** (80 MHz, $CDCl_3$)

1,5-diethyl-1,1,5,5-tetraisopropyl-3-phenyltrisiloxane (19)

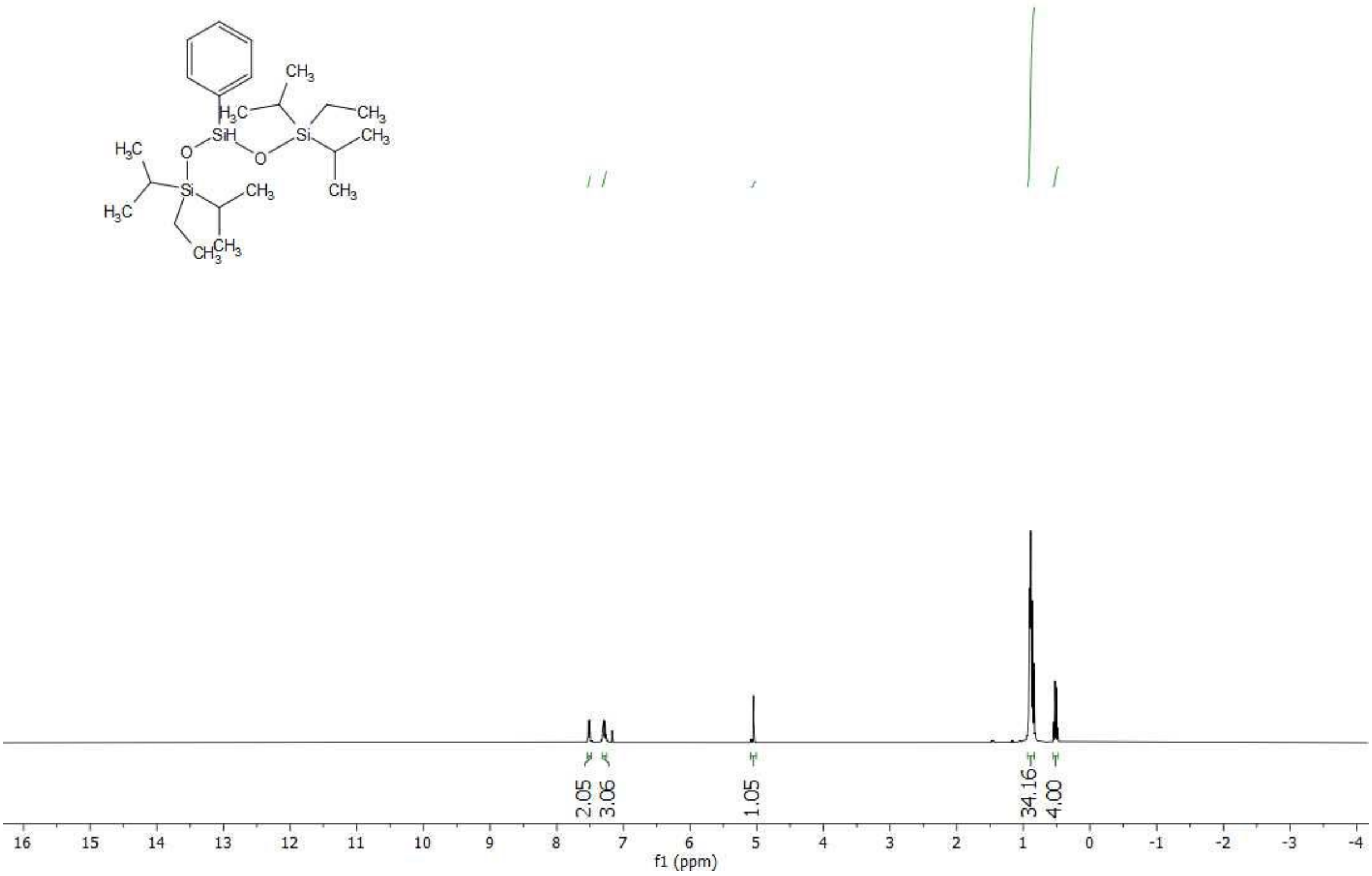

Figure SS50. ^{1}H NMR Spectrum of **19** (400 MHz, $CDCl_3$)

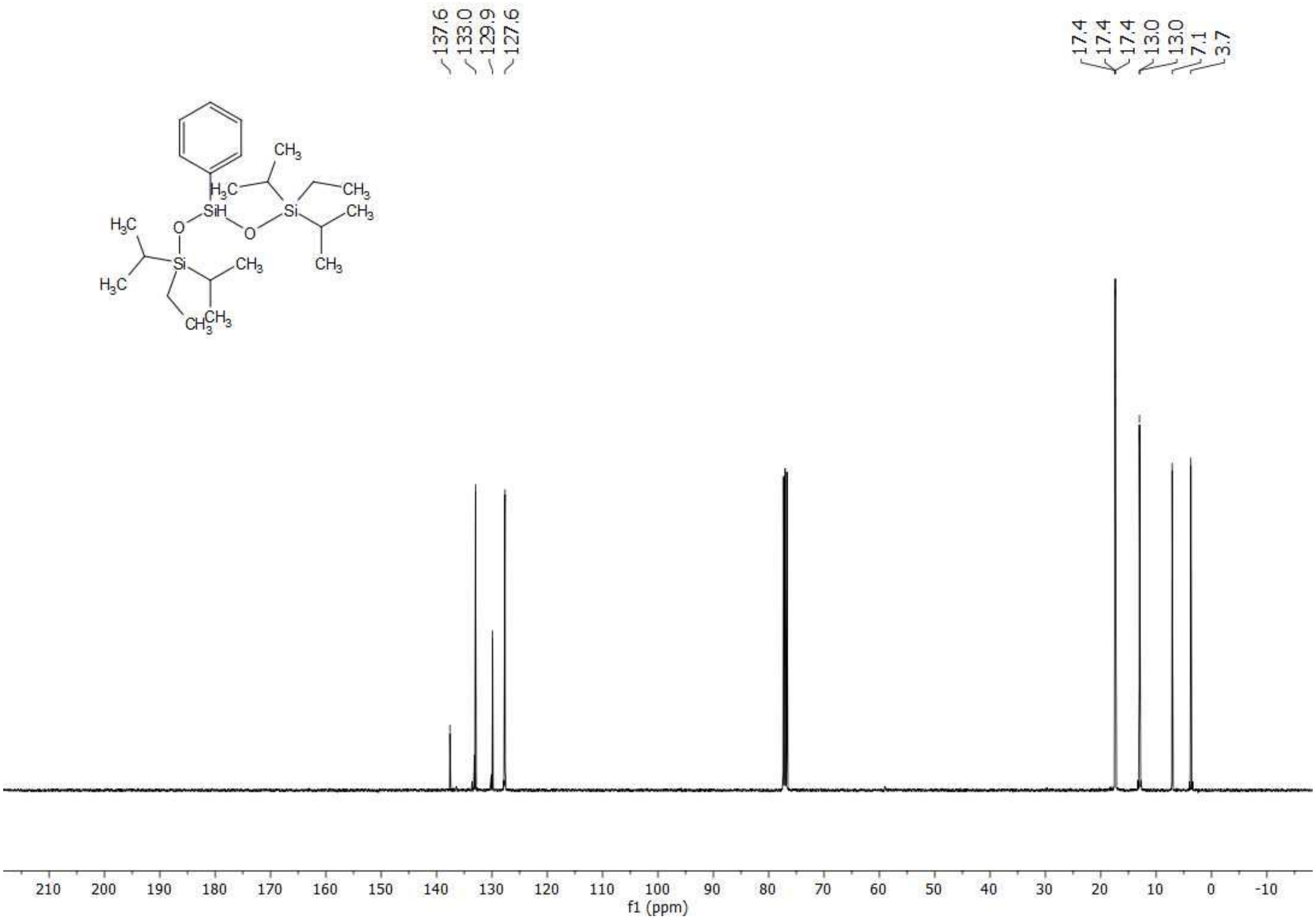

Figure SS51. ^{13}C NMR Spectrum of **19** (101 MHz, $CDCl_3$)

3,3-diisopropyl-1,5-diphenyltrisiloxane (20)

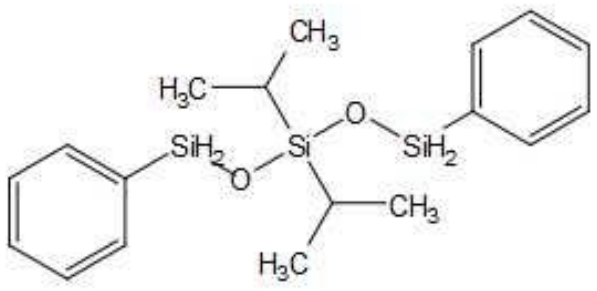

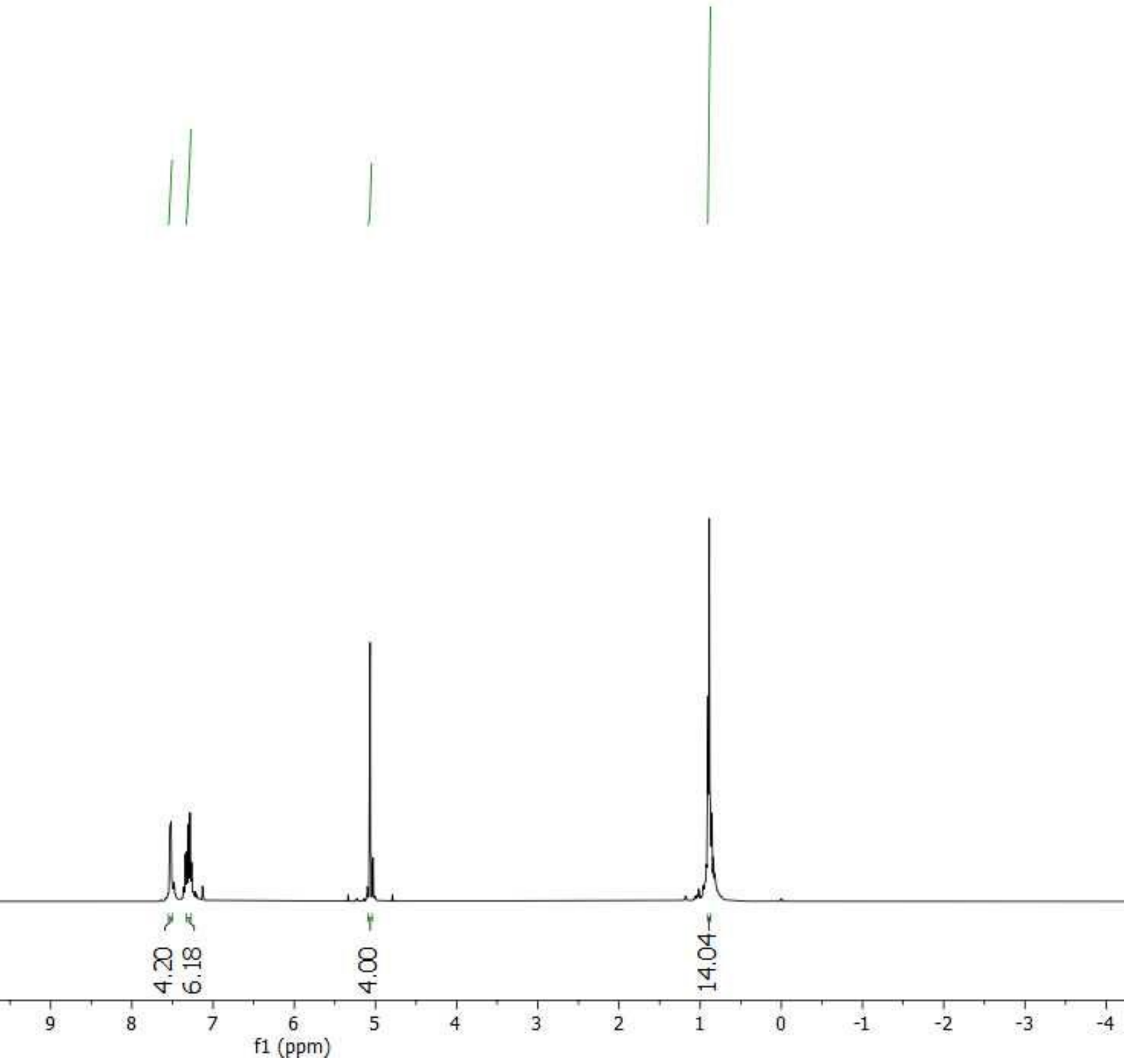

Figure SS52. ^{1}H NMR Spectrum of **20** (400 MHz, $CDCl_3$)

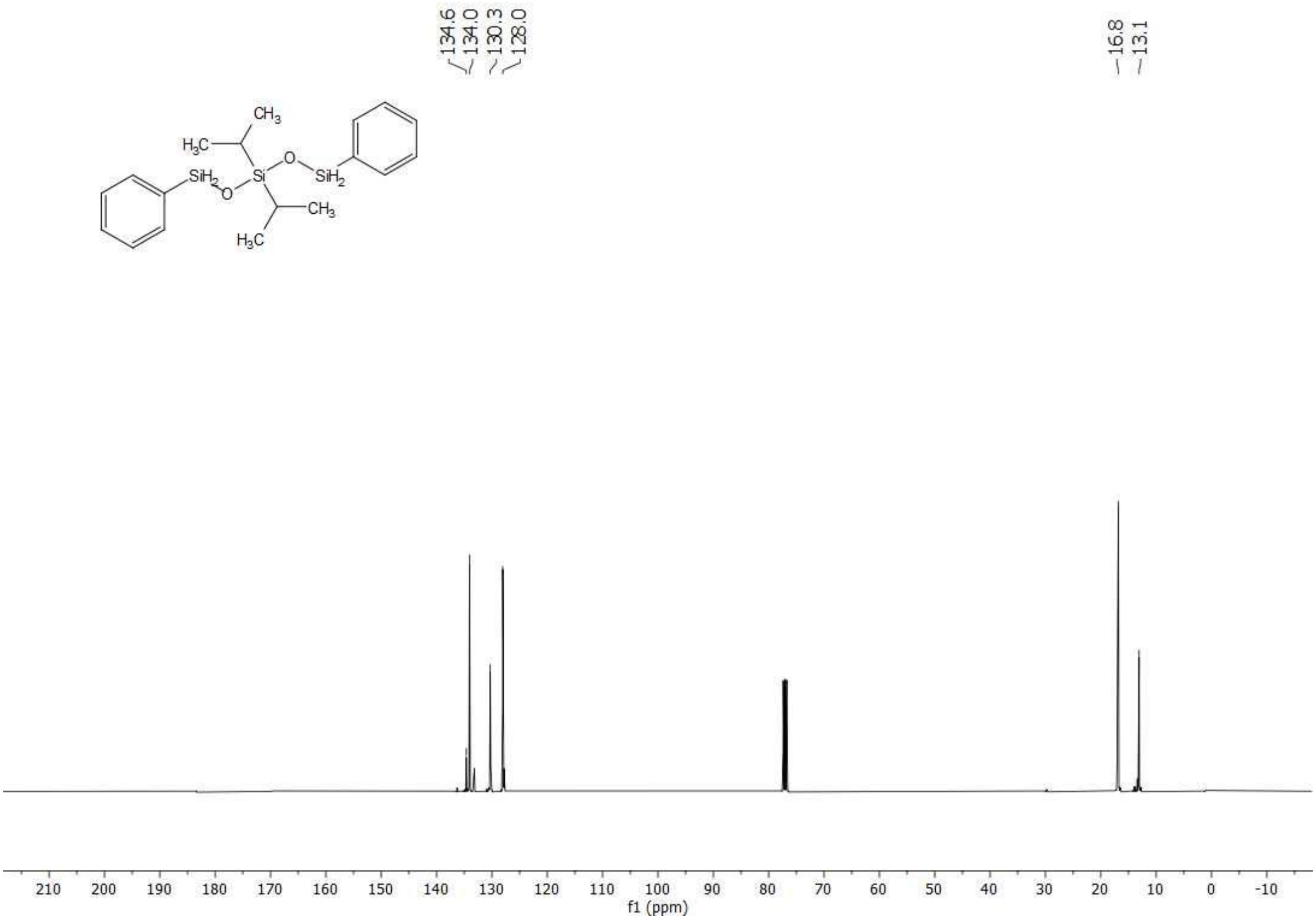

Figure SS53. ^{13}C NMR Spectrum of **20** (101 MHz, $CDCl_3$)

1,1,1,3,5,5,5-heptaphenyltrisiloxane (21)

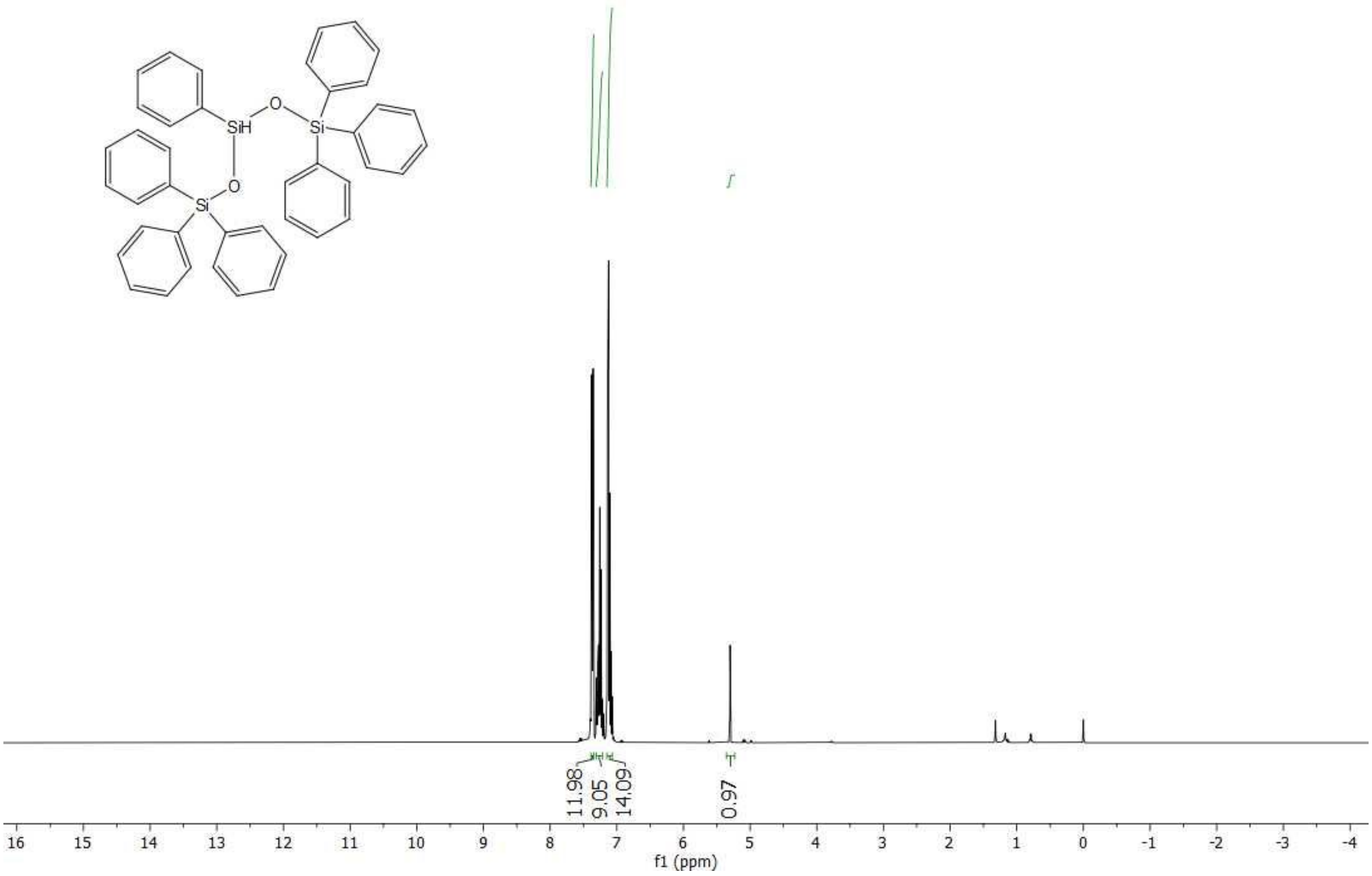

Figure SS54. ^{1}H NMR Spectrum of **21** (400 MHz, $CDCl_3$)

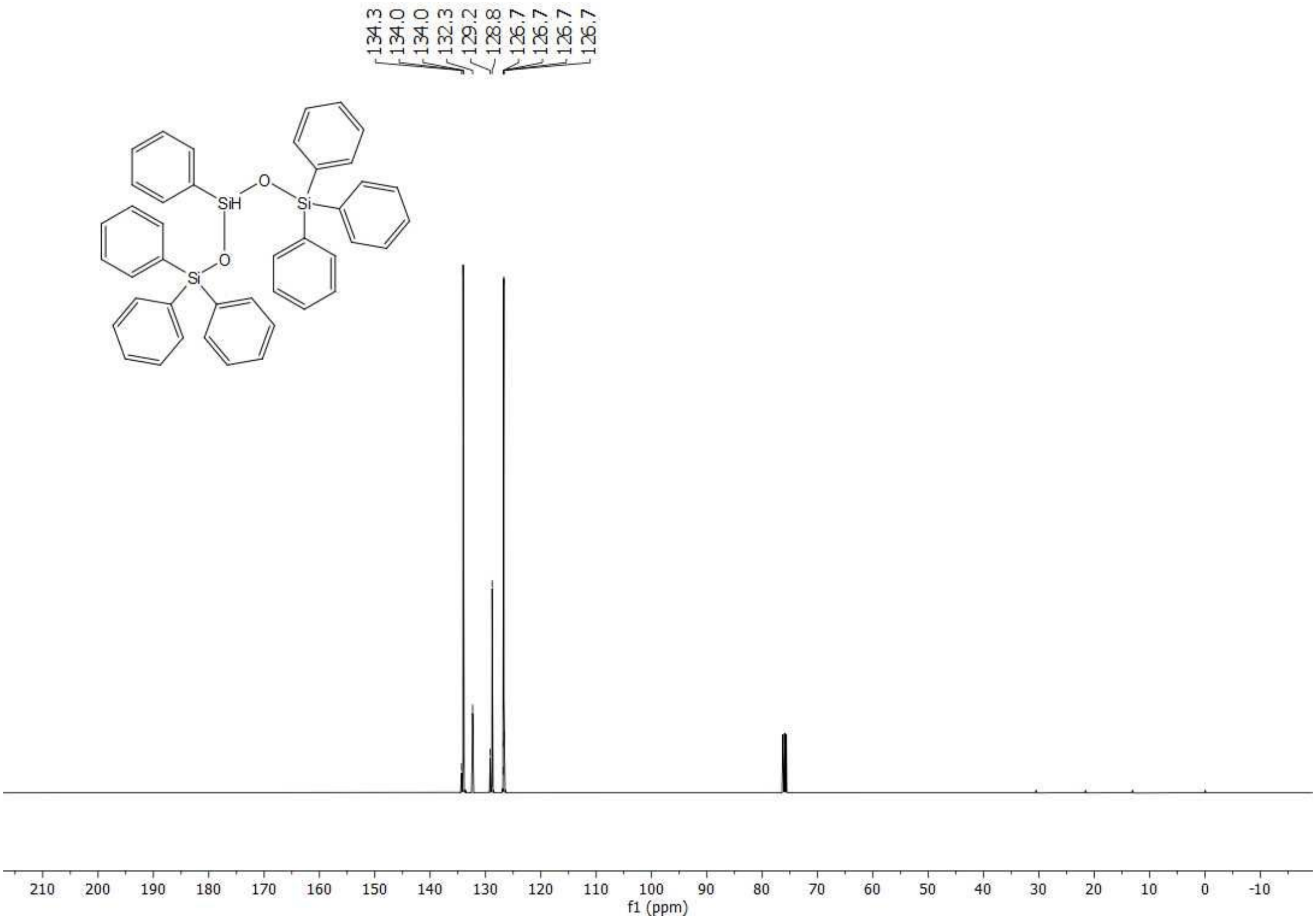

Figure SS55. ^{13}C NMR Spectrum of **21** (101 MHz, $CDCl_3$)

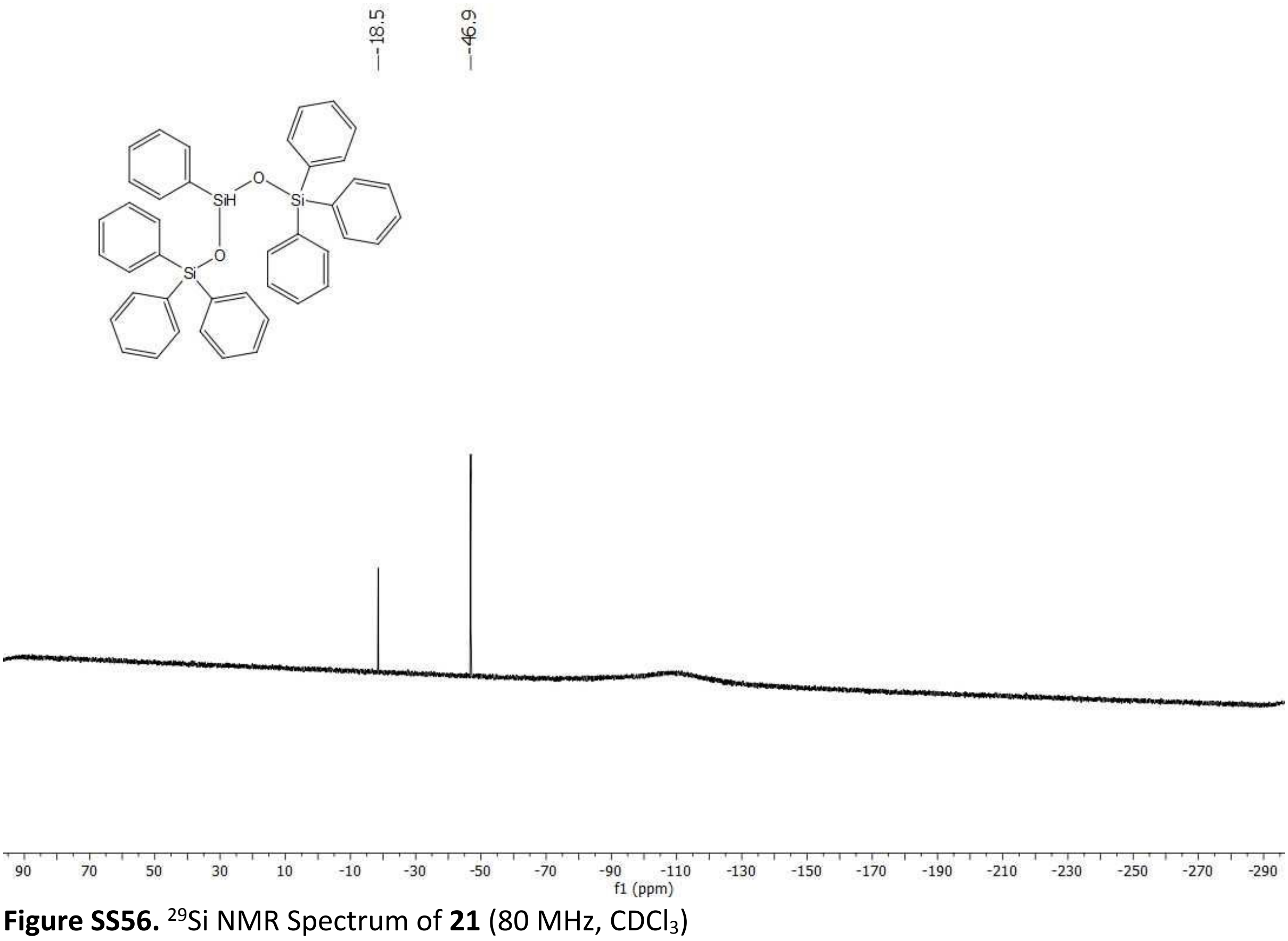

Figure SS56. ^{29}Si NMR Spectrum of **21** (80 MHz, $CDCl_3$)

1,1,1,5,5,5-hexaethyl-3-(p-tolyl)trisiloxane (22)

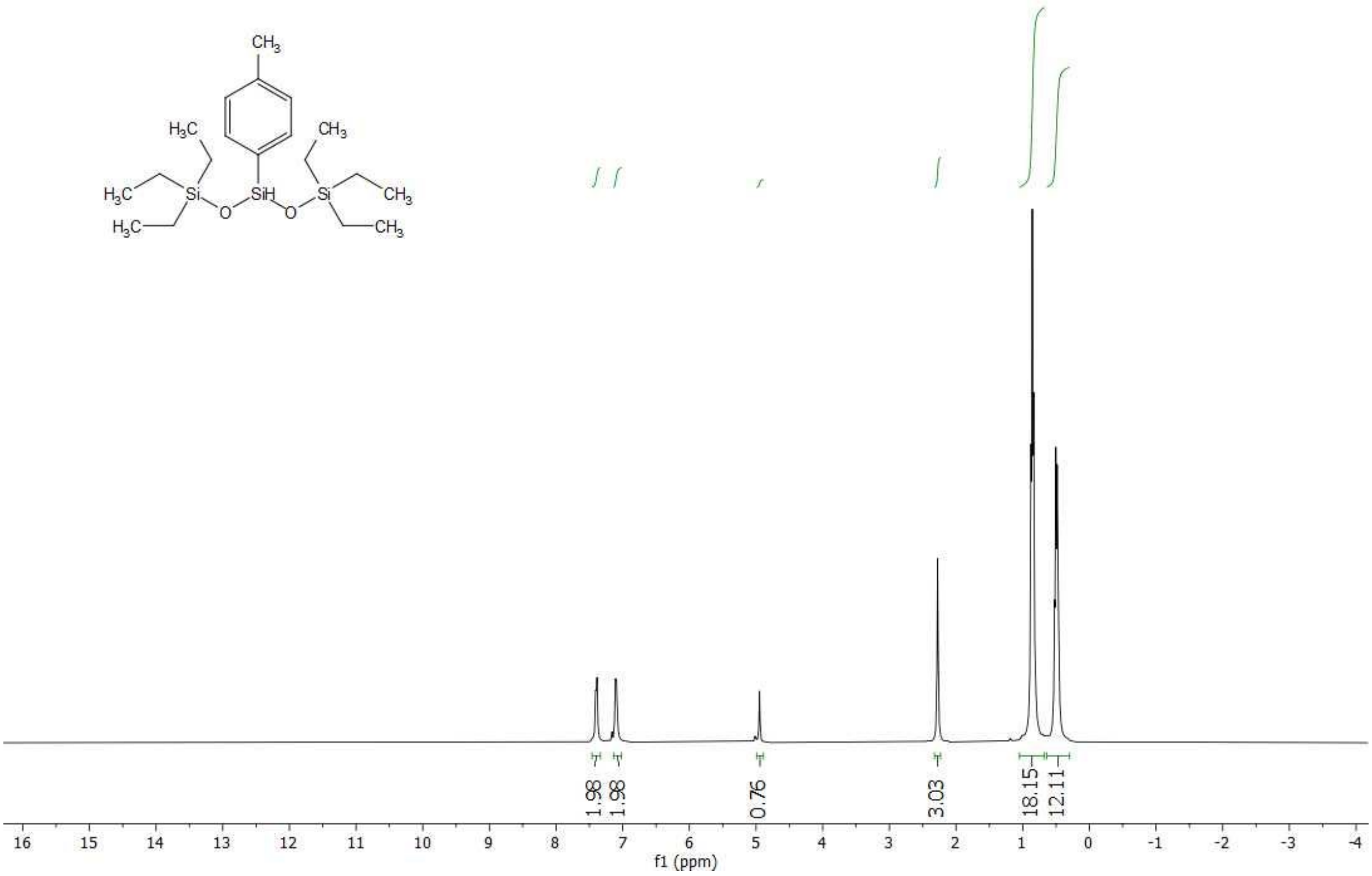

Figure SS57. ^{1}H NMR Spectrum of **22** (400 MHz, $CDCl_3$)

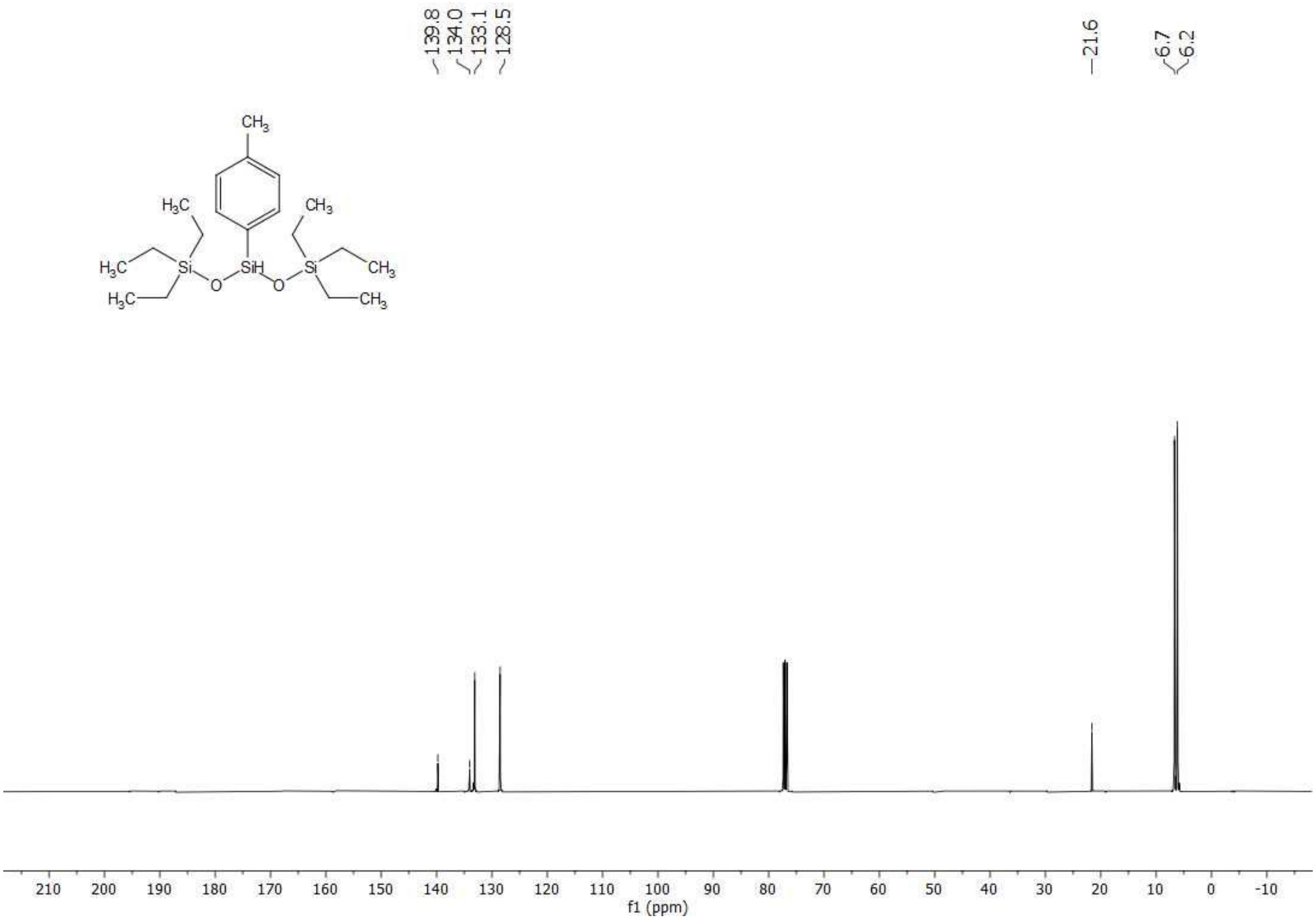

Figure SS58. ^{13}C NMR Spectrum of **22** (101 MHz, $CDCl_3$)

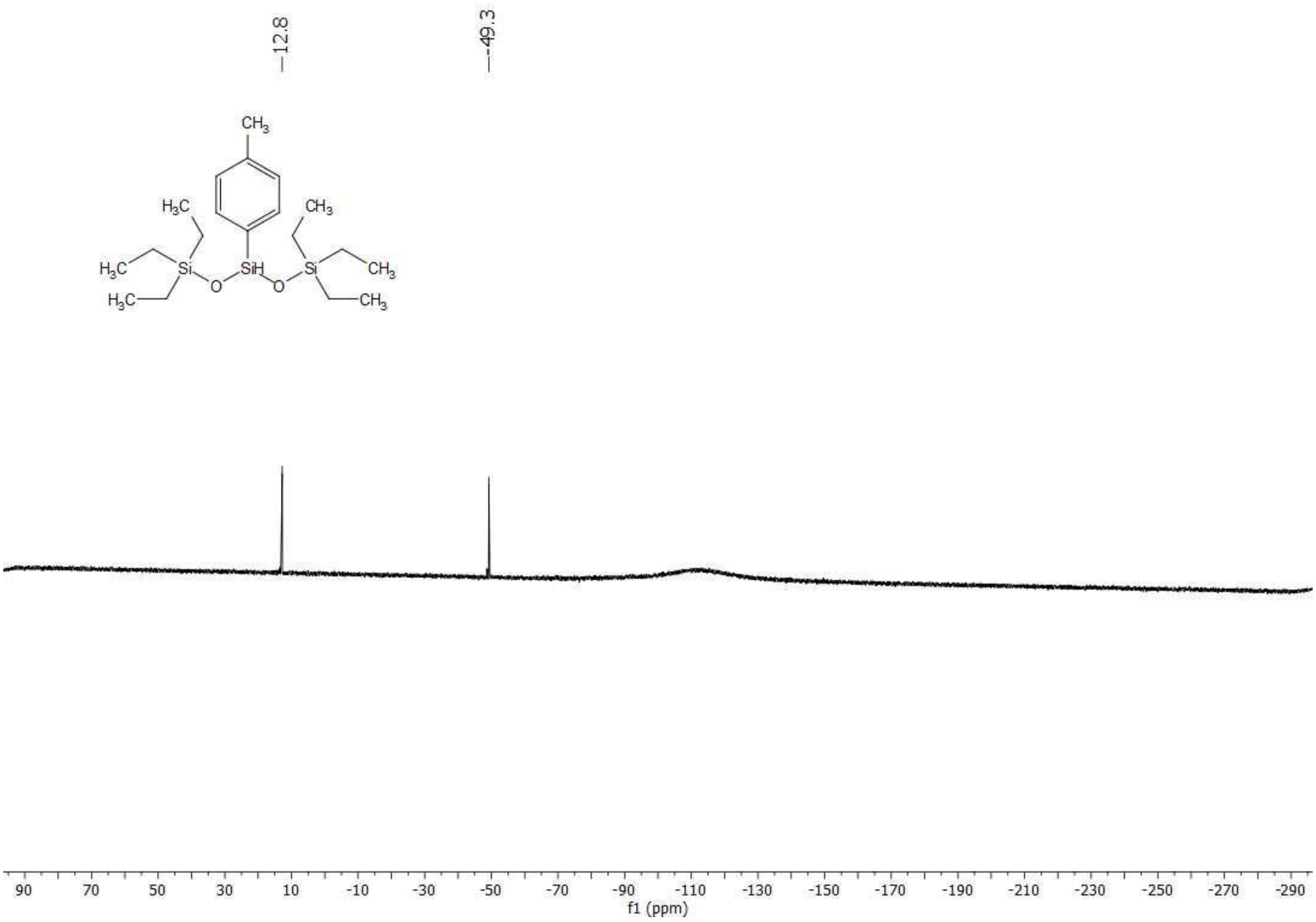

Figure SS59. ^{29}Si NMR Spectrum of **22** (80 MHz, $CDCl_3$)

1,5-di-tert-butyl-1,1,5,5-tetramethyl-3-(p-tolyl)trisiloxane (23)

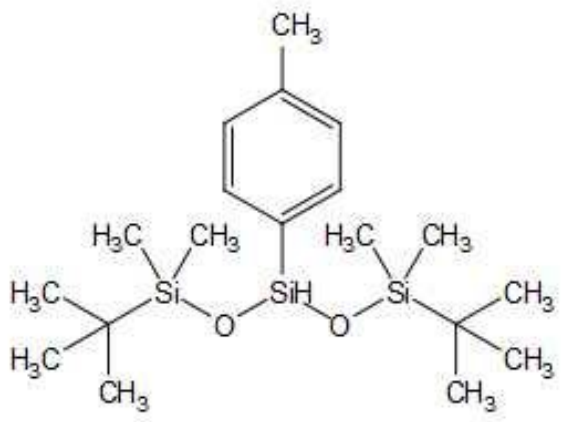

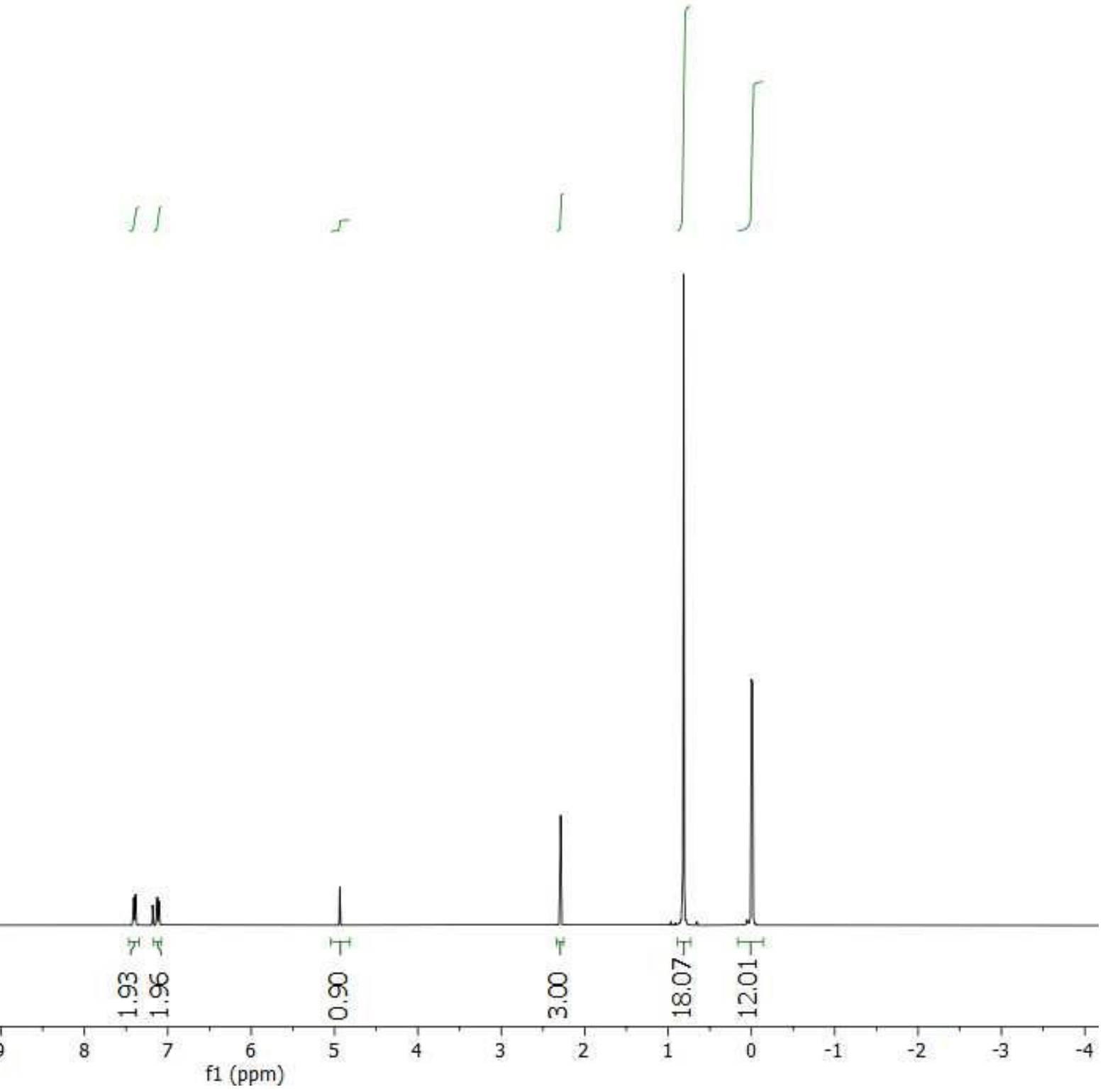

Figure SS60. ^{1}H NMR Spectrum of **23** (400 MHz, $CDCl_3$)

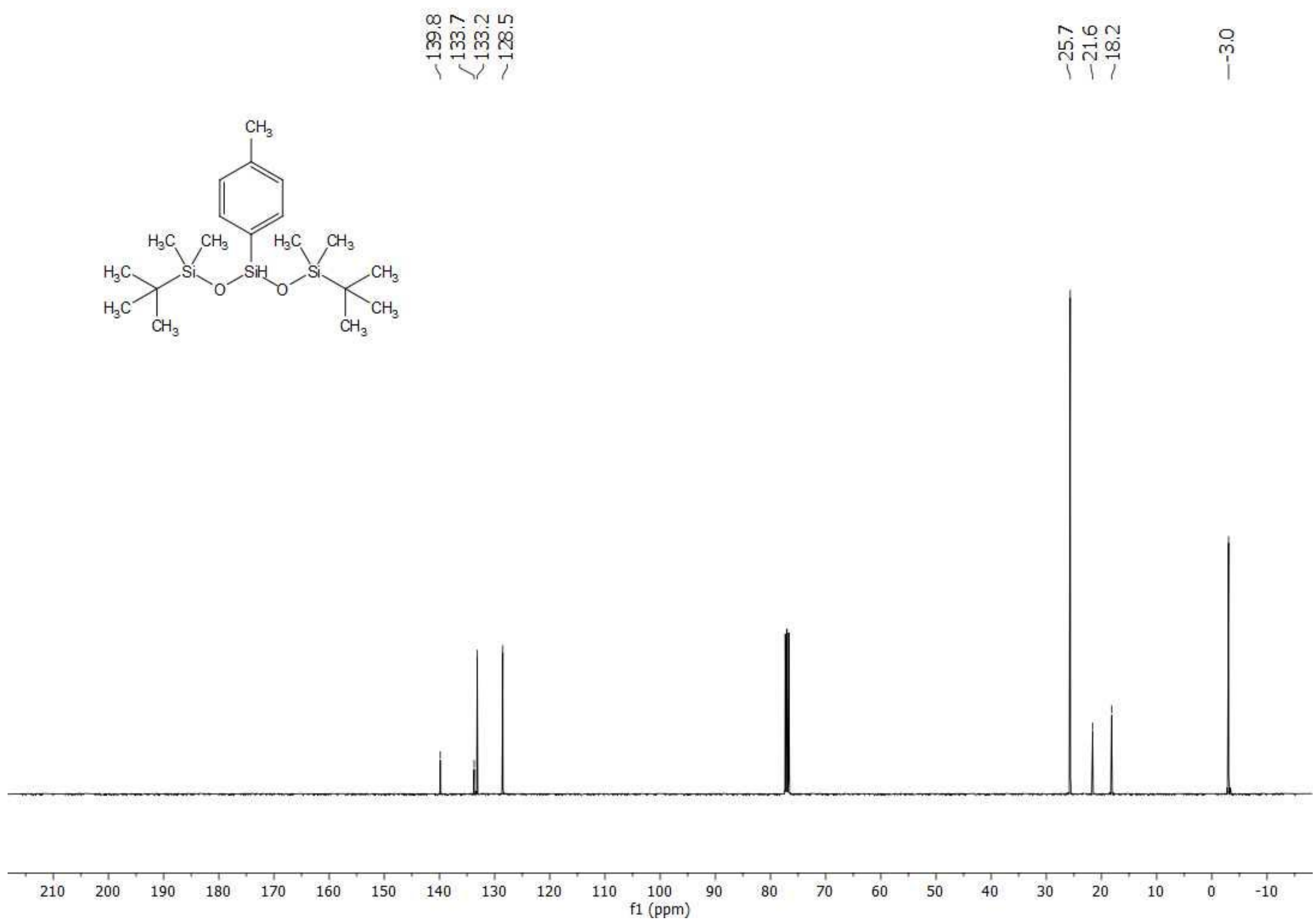

Figure SS61. ^{13}C NMR Spectrum of **23** (101 MHz, $CDCl_3$)

1,1,1,5,5,5-hexaisopropyl-3-(p-tolyl)trisiloxane (24)

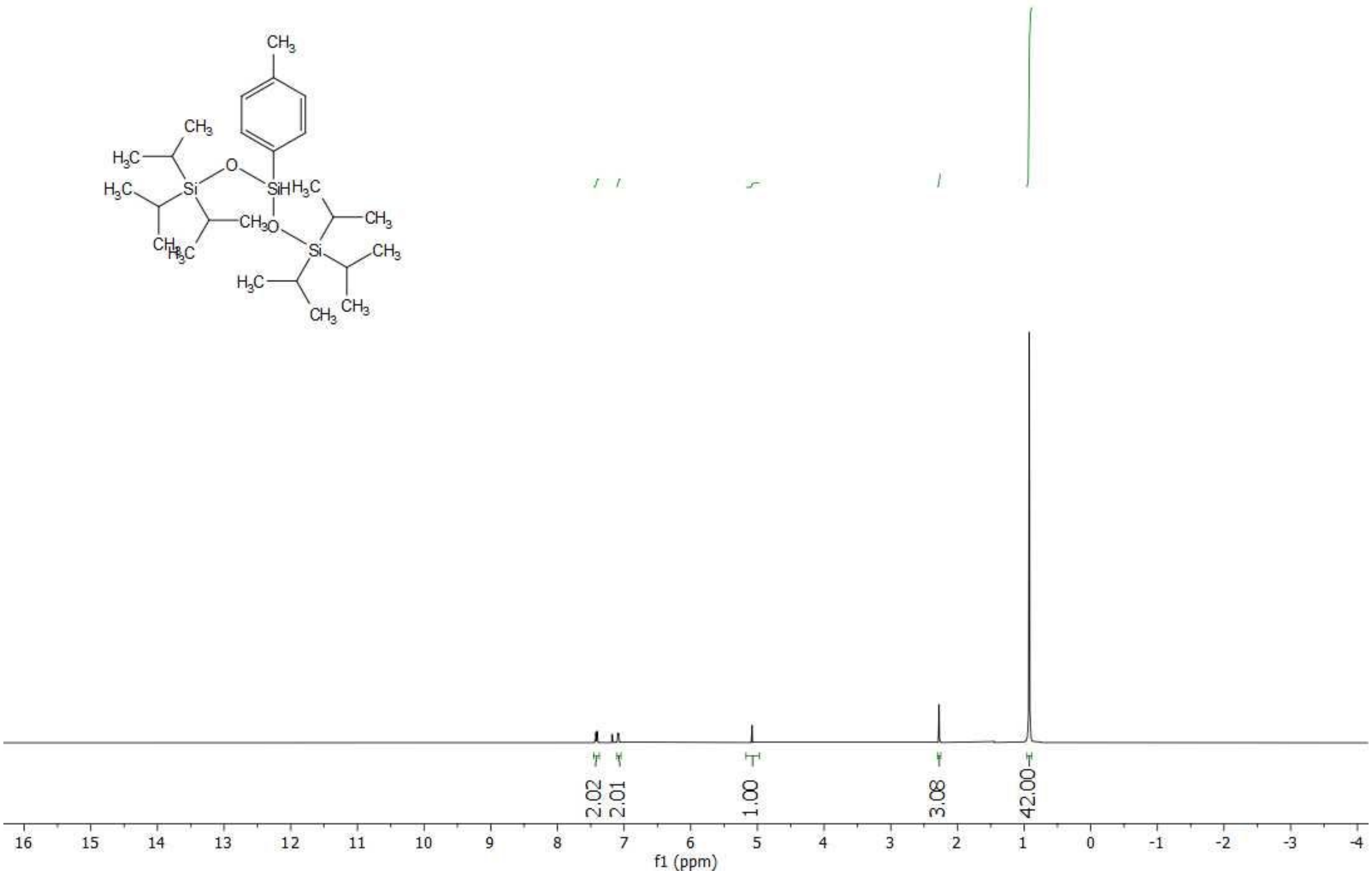

Figure SS62. ^{1}H NMR Spectrum of **24** (400 MHz, $CDCl_3$)

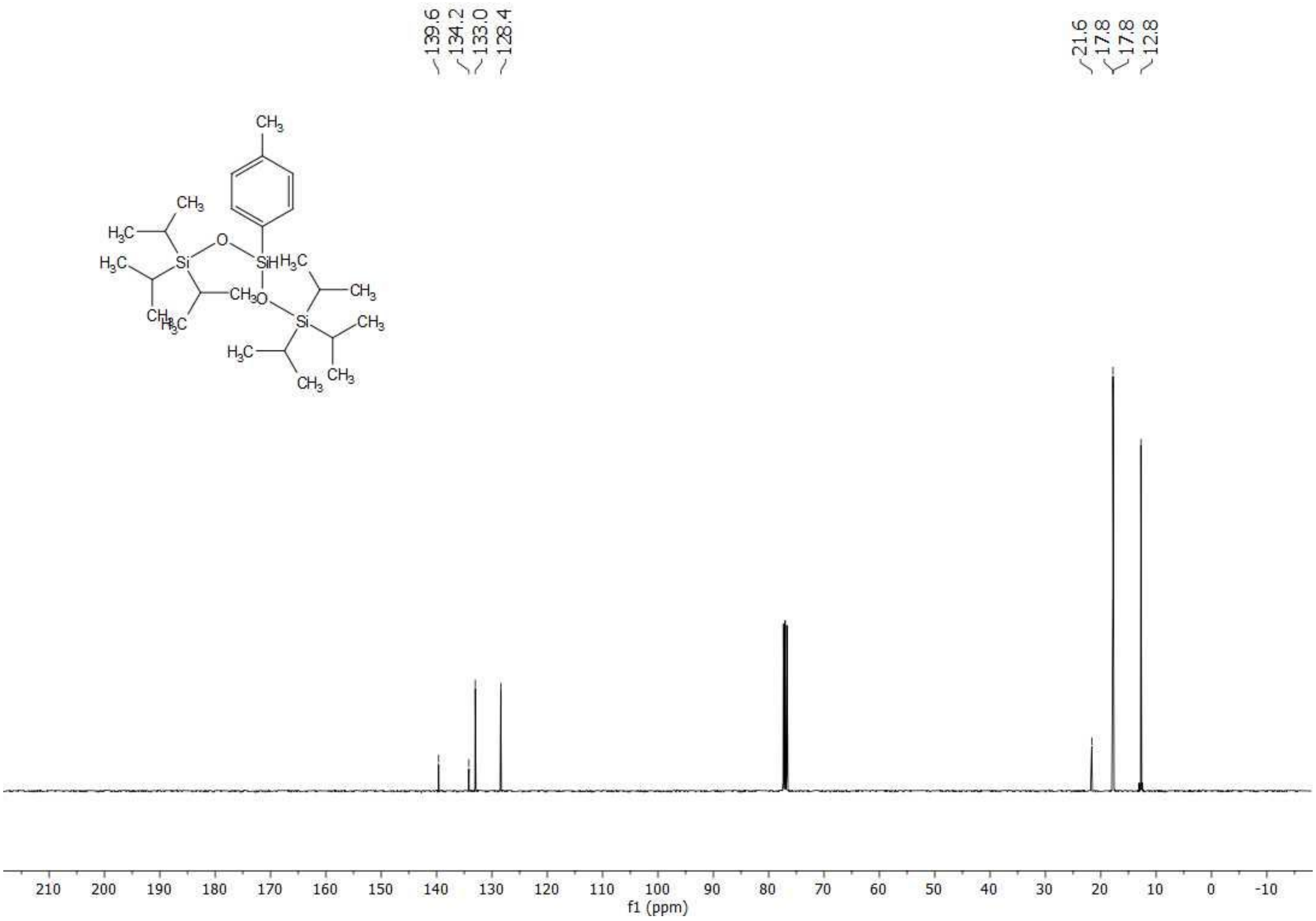

Figure SS63. ^{13}C NMR Spectrum of **24** (101 MHz, $CDCl_3$)

1,1,1,5,5,5-hexahexyl-3-(p-tolyl)trisiloxane (25)

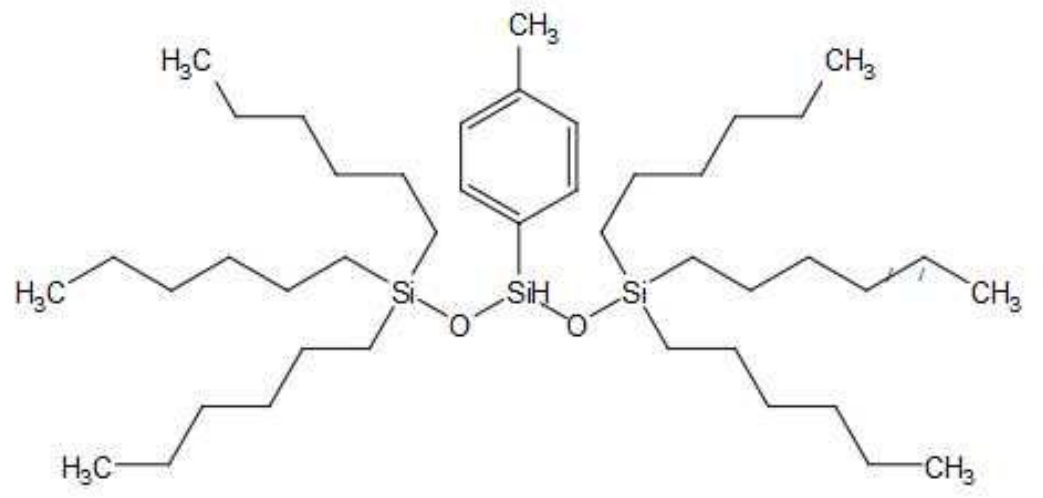

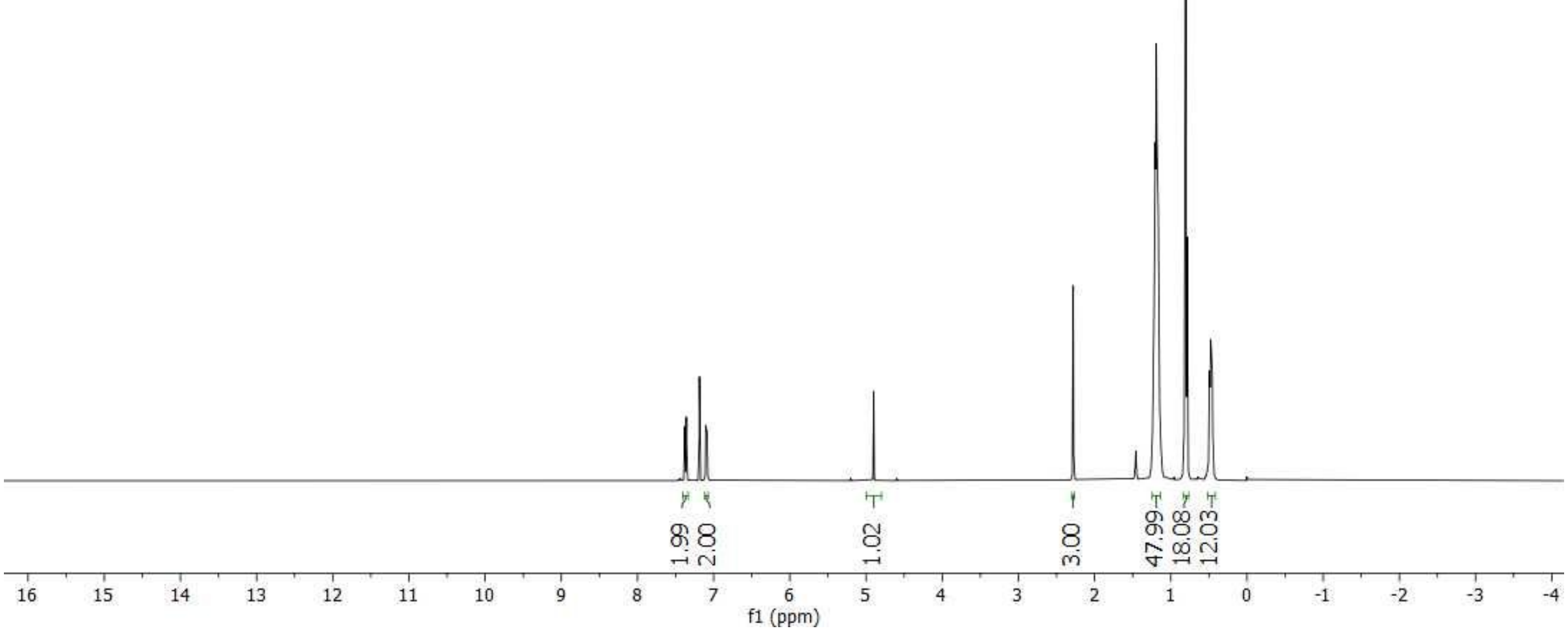

Figure SS64. ^{1}H NMR Spectrum of **25** (400 MHz, $CDCl_3$)

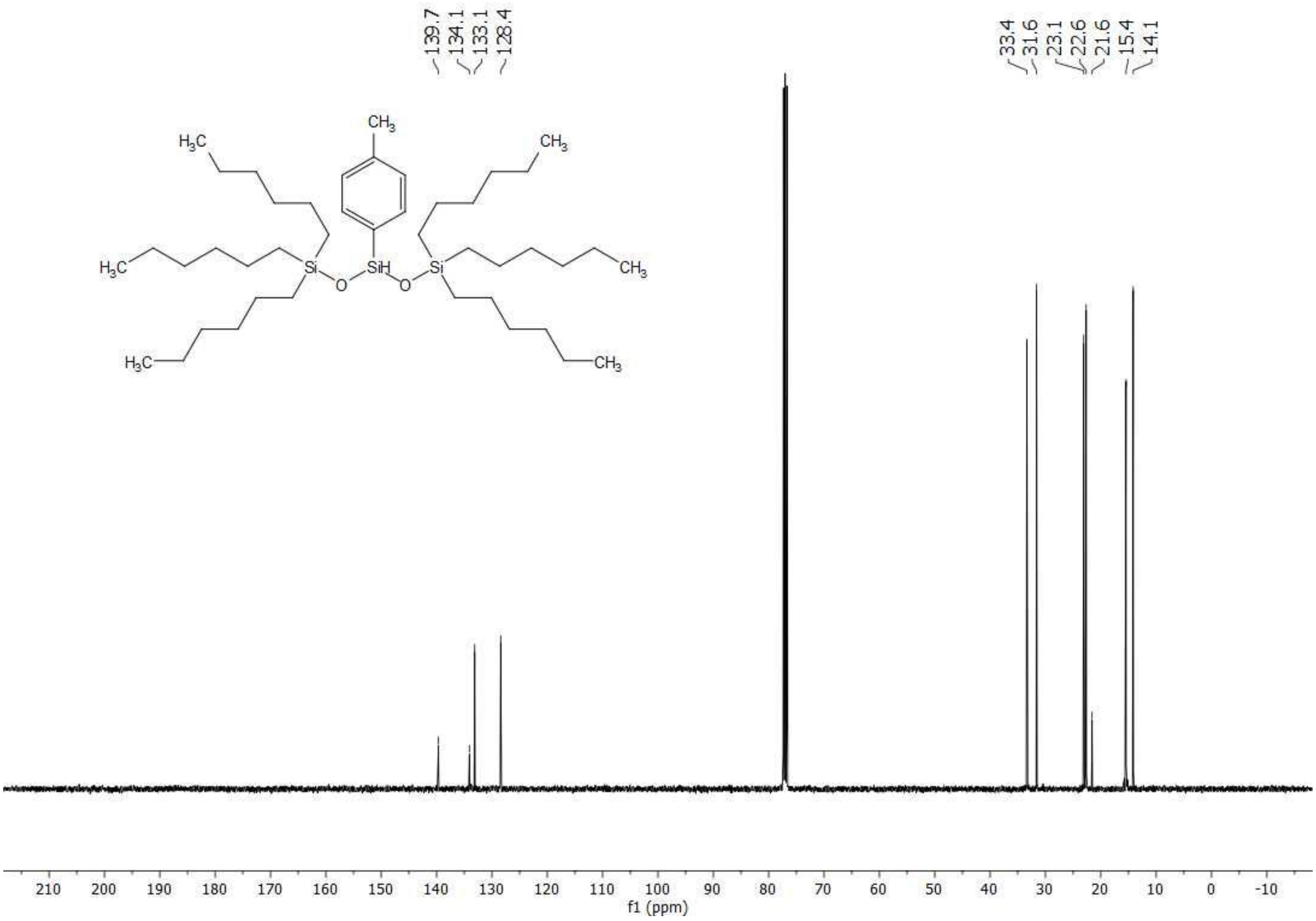

Figure SS65. ^{13}C NMR Spectrum of **25** (101 MHz, $CDCl_3$)

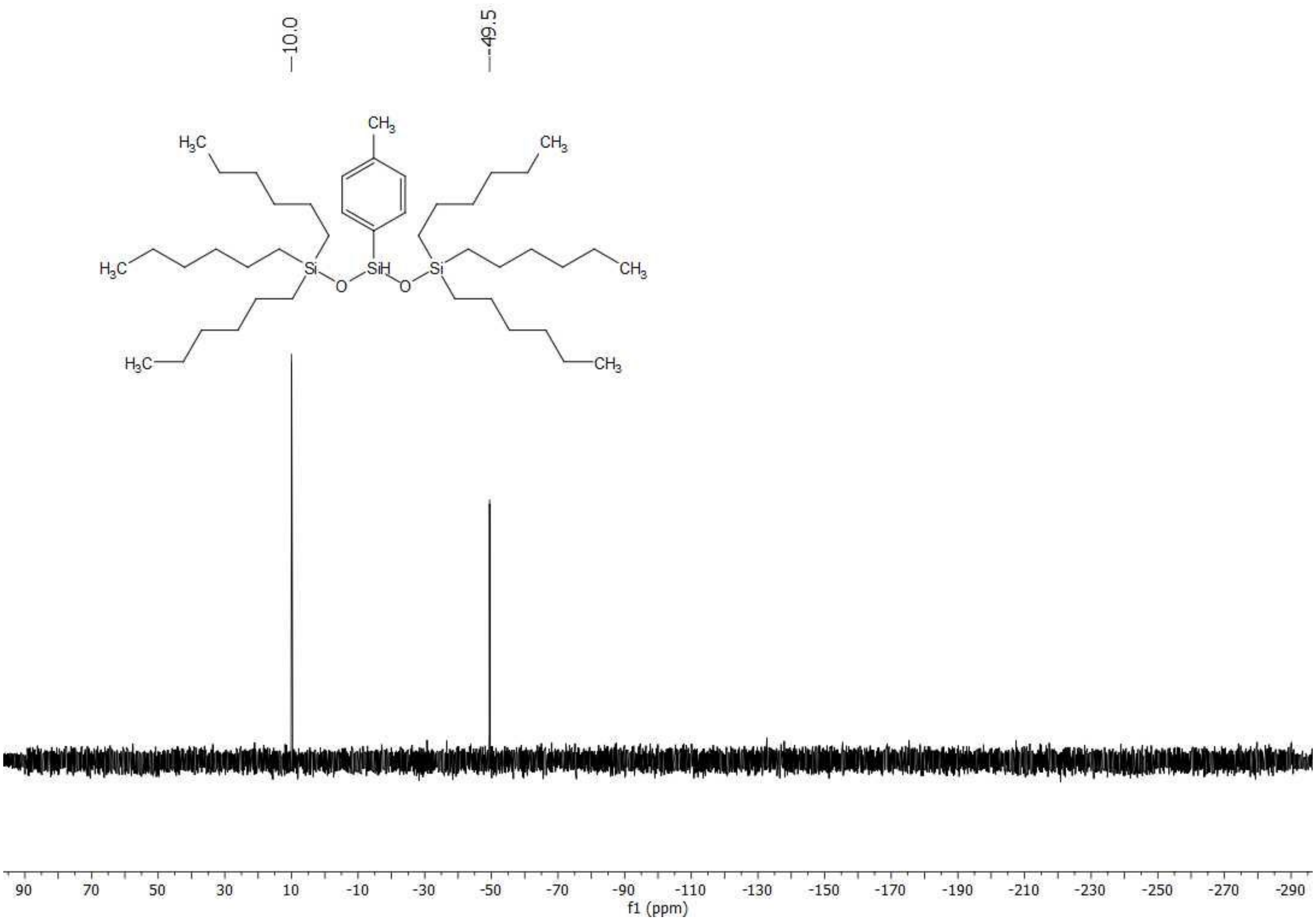

Figure SS66. ^{29}Si NMR Spectrum of **25** (80 MHz, $CDCl_3$)

1,1,1,5,5,5-hexaisobutyl-3-(p-tolyl)trisiloxane (26)

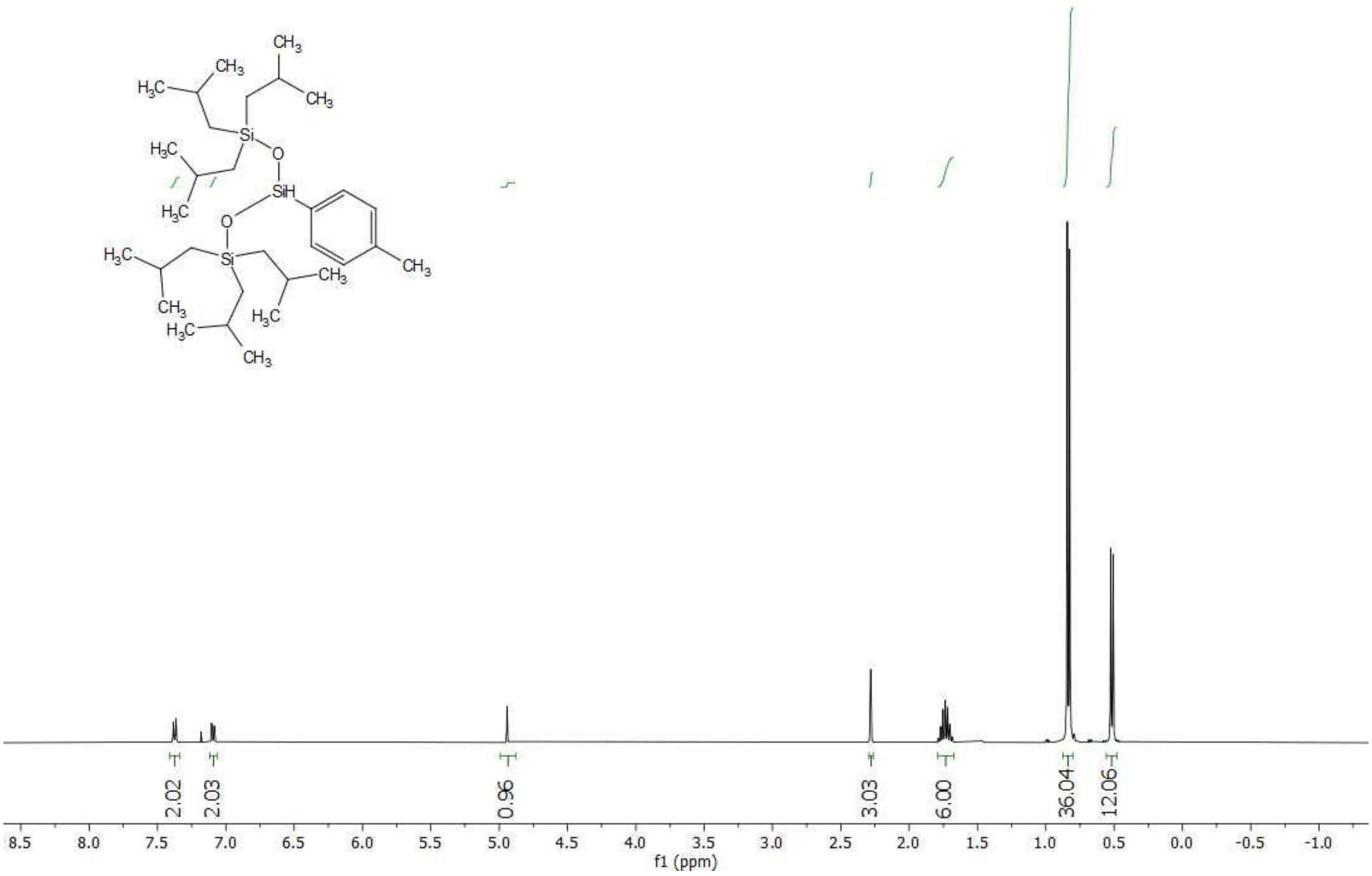

Figure SS67. ^{1}H NMR Spectrum of **26** (400 MHz, $CDCl_3$)

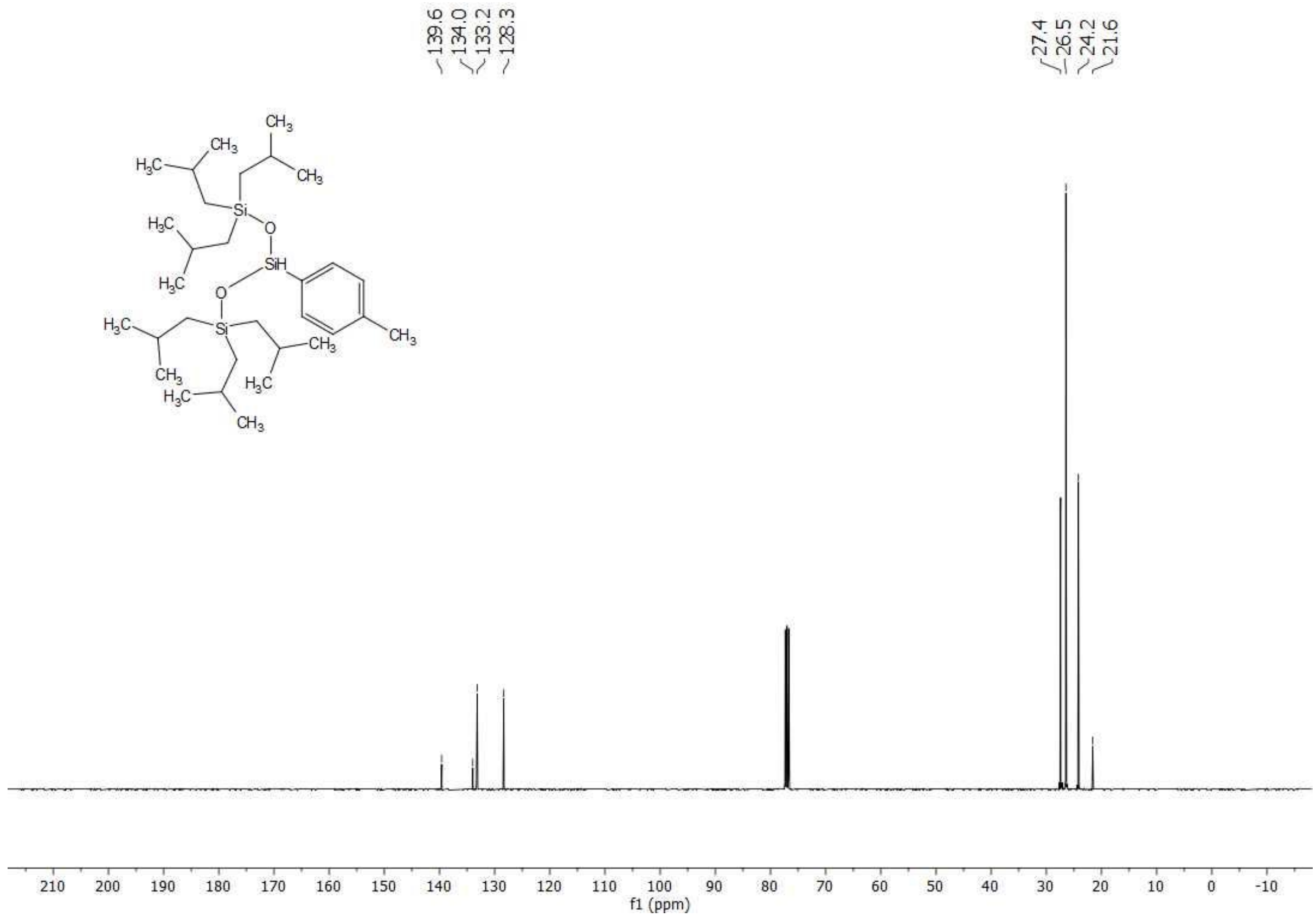

Figure SS68. ^{13}C NMR Spectrum of **26** (101 MHz, $CDCl_3$)

1,1,5,5-tetramethyl-1,5-diphenyl-3-(p-tolyl)trisiloxane (27)

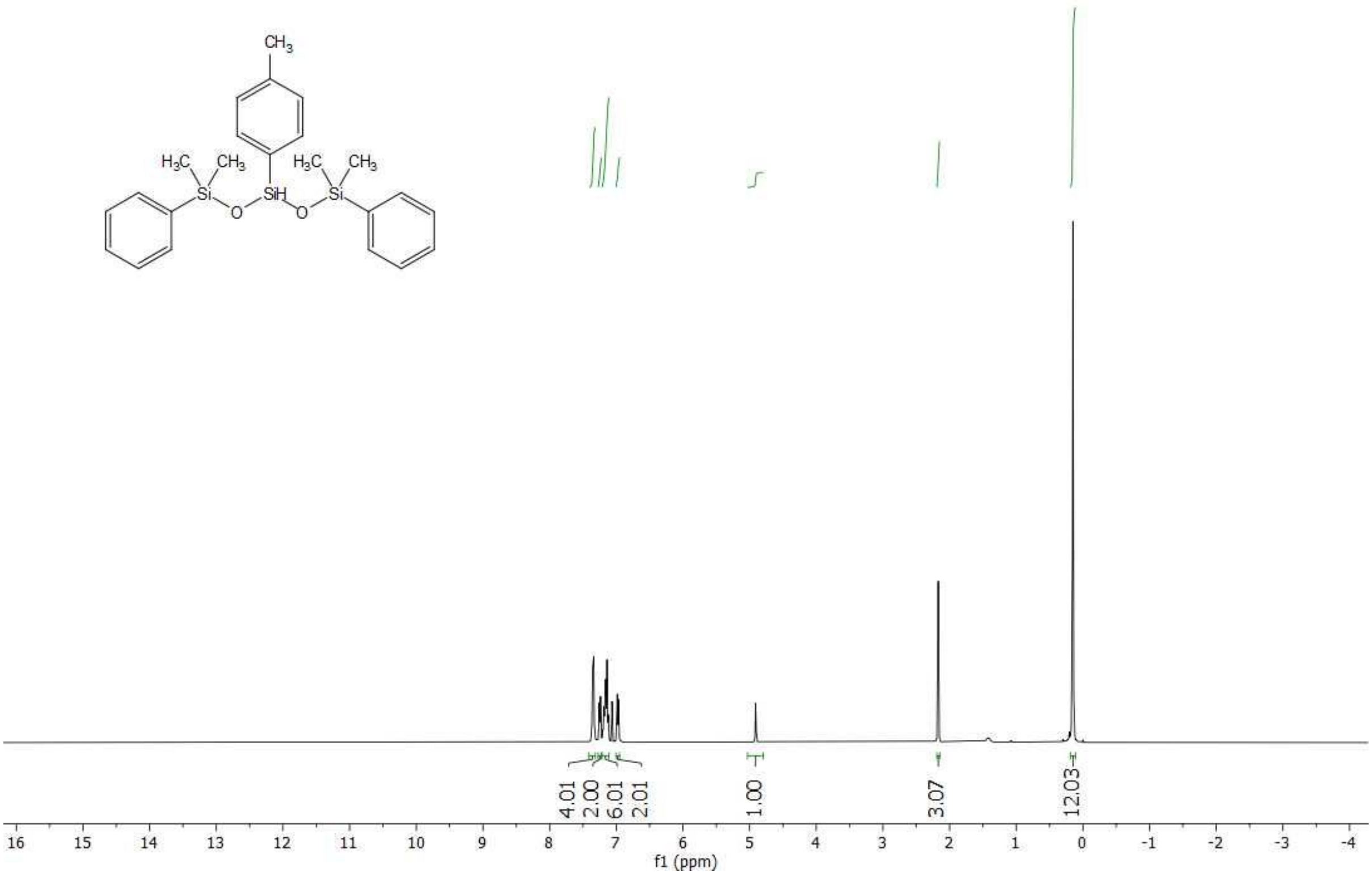

Figure SS69. ^{1}H NMR Spectrum of **27** (400 MHz, $CDCl_3$)

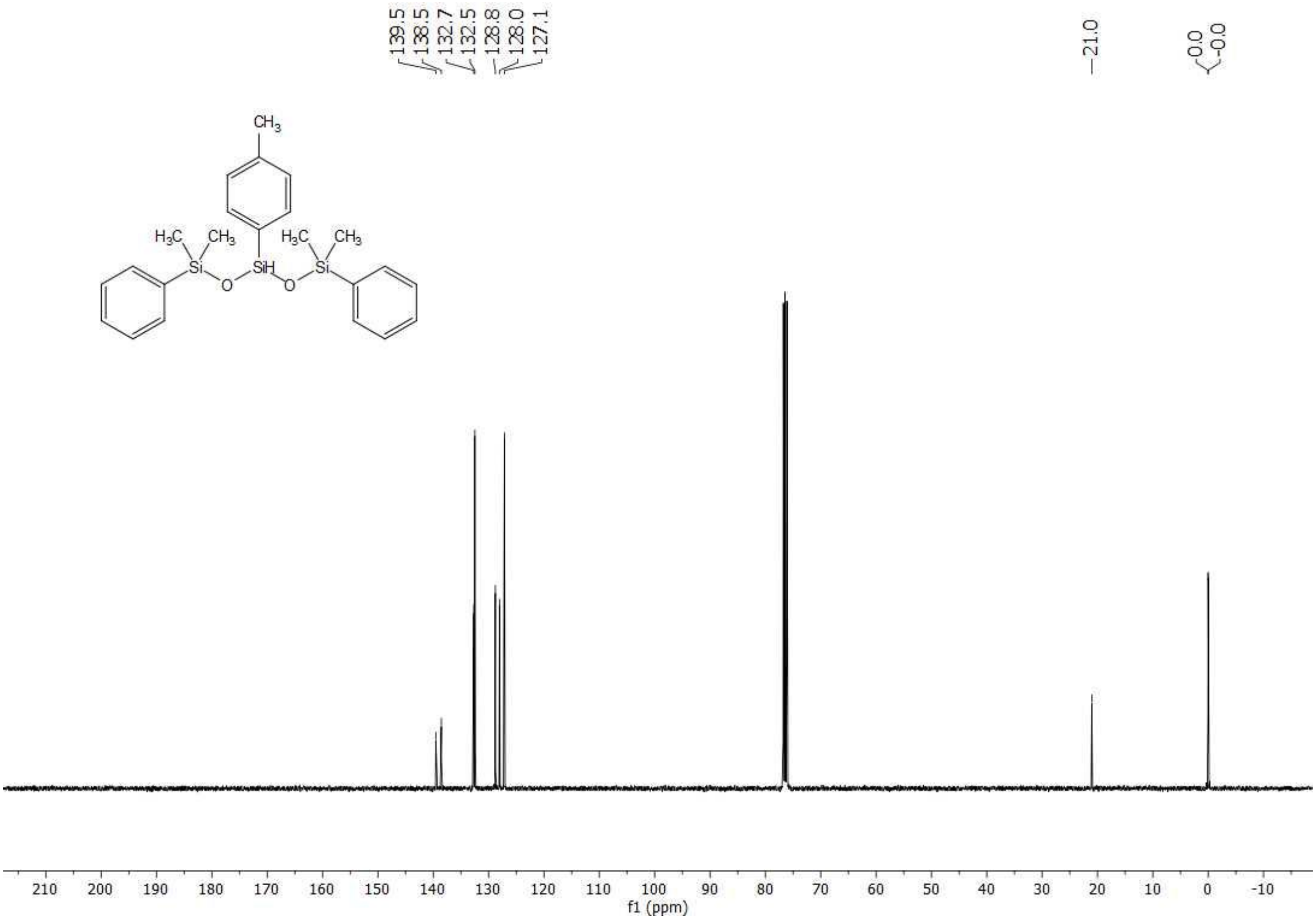

Figure SS70. ^{13}C NMR Spectrum of **27** (101 MHz, $CDCl_3$)

3,3-diisopropyl-1,5-di-p-tolyltrisiloxane (28)

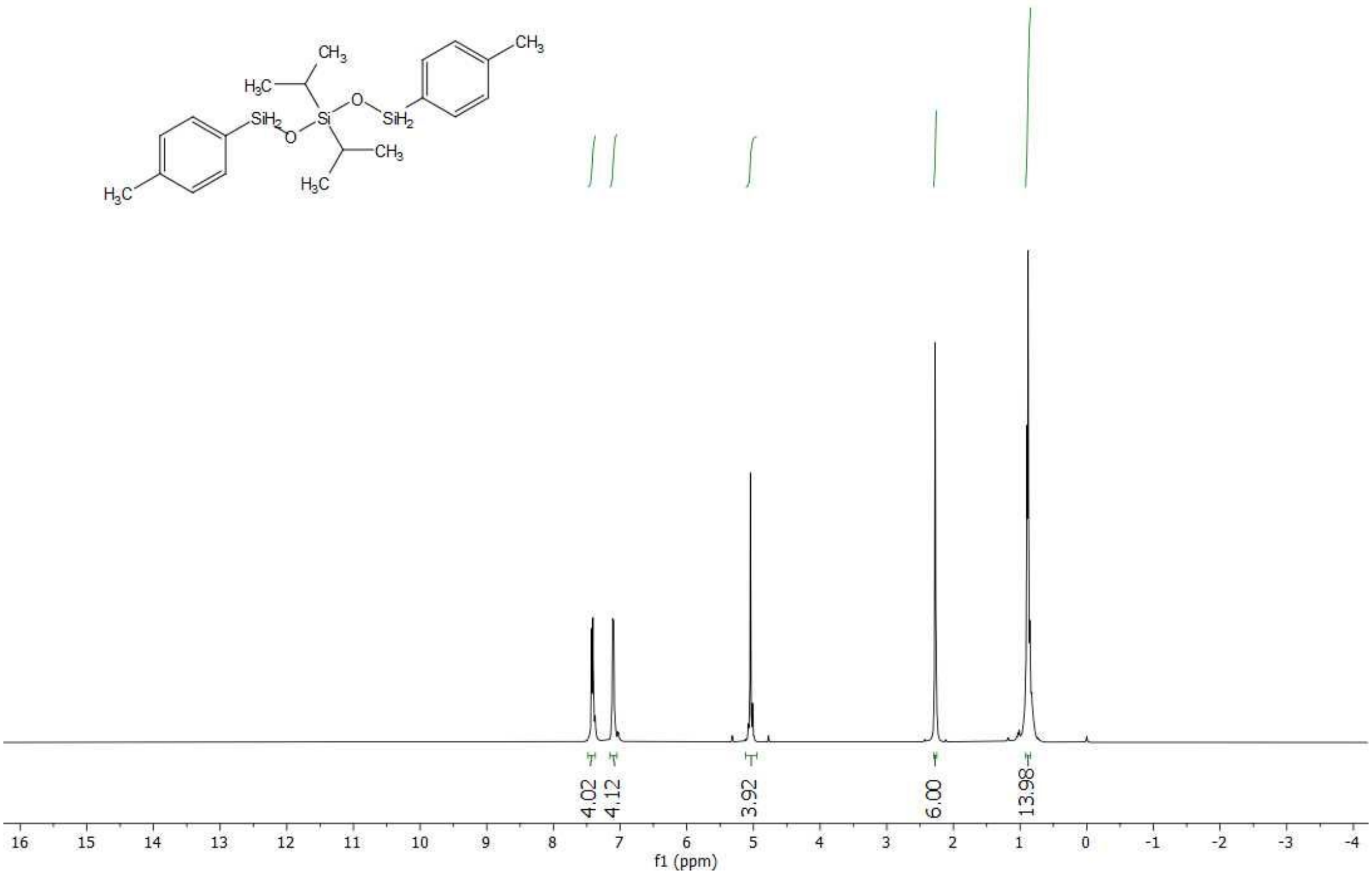

Figure SS71. ^{1}H NMR Spectrum of **28** (400 MHz, $CDCl_3$)

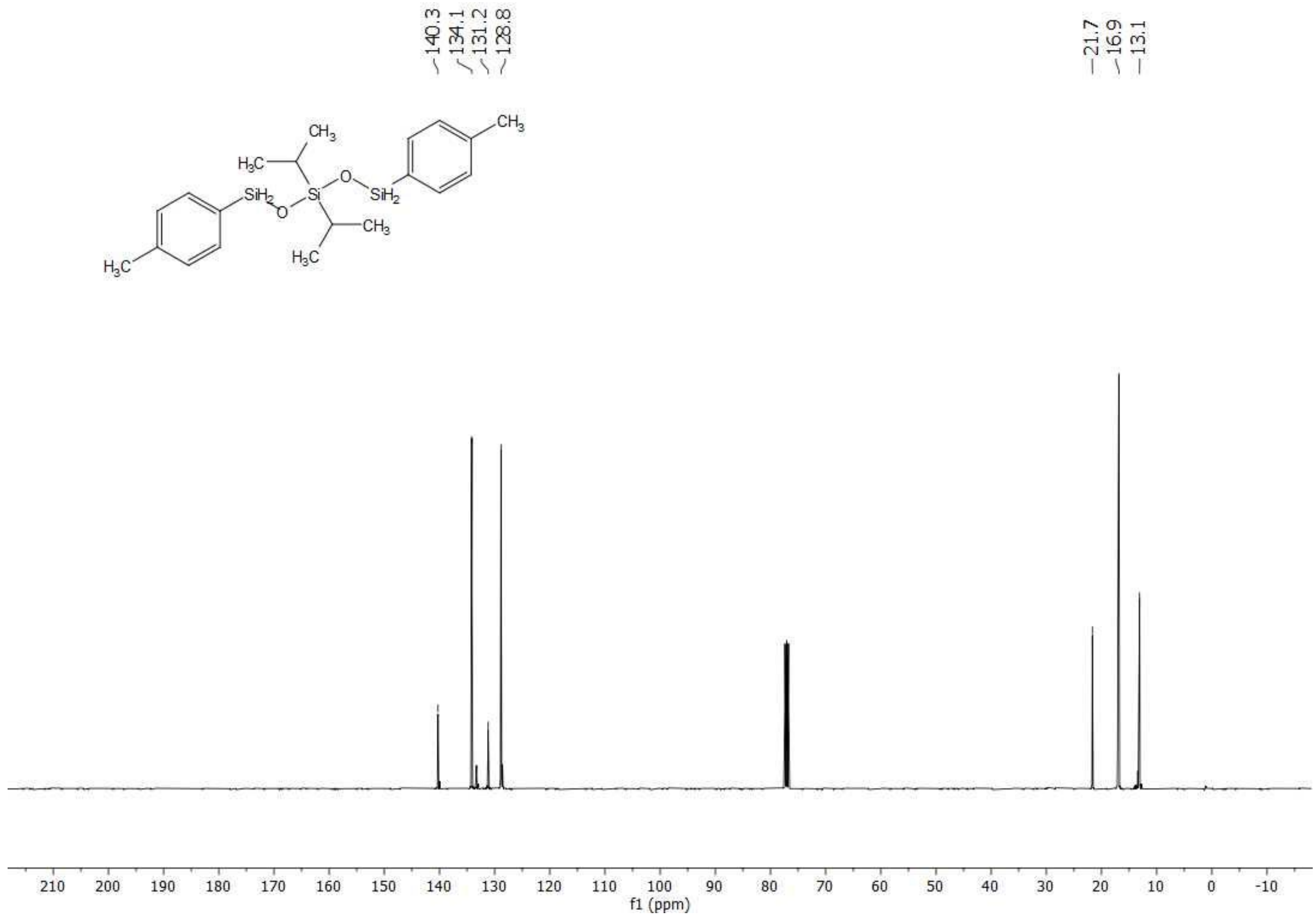

Figure SS72. ^{13}C NMR Spectrum of **28** (101 MHz, $CDCl_3$)

3-hexyl-1,1,1,5,5,5-hexamethyltrisiloxane (29)

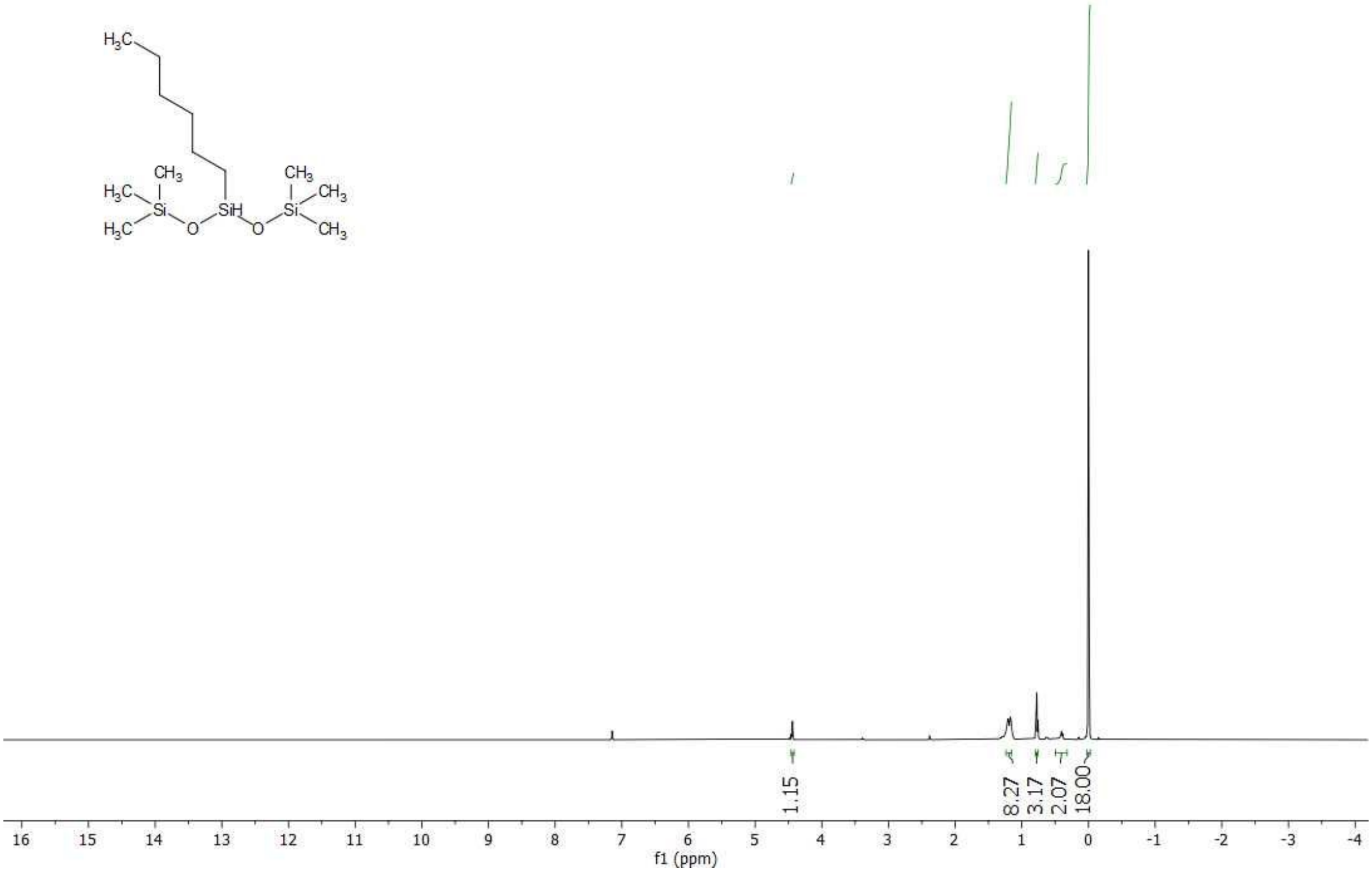

Figure SS73. ^{1}H NMR Spectrum of **29** (400 MHz, $CDCl_3$)

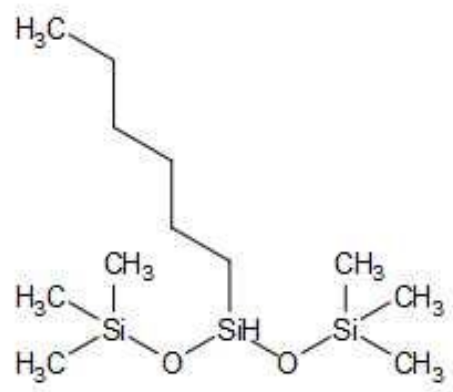

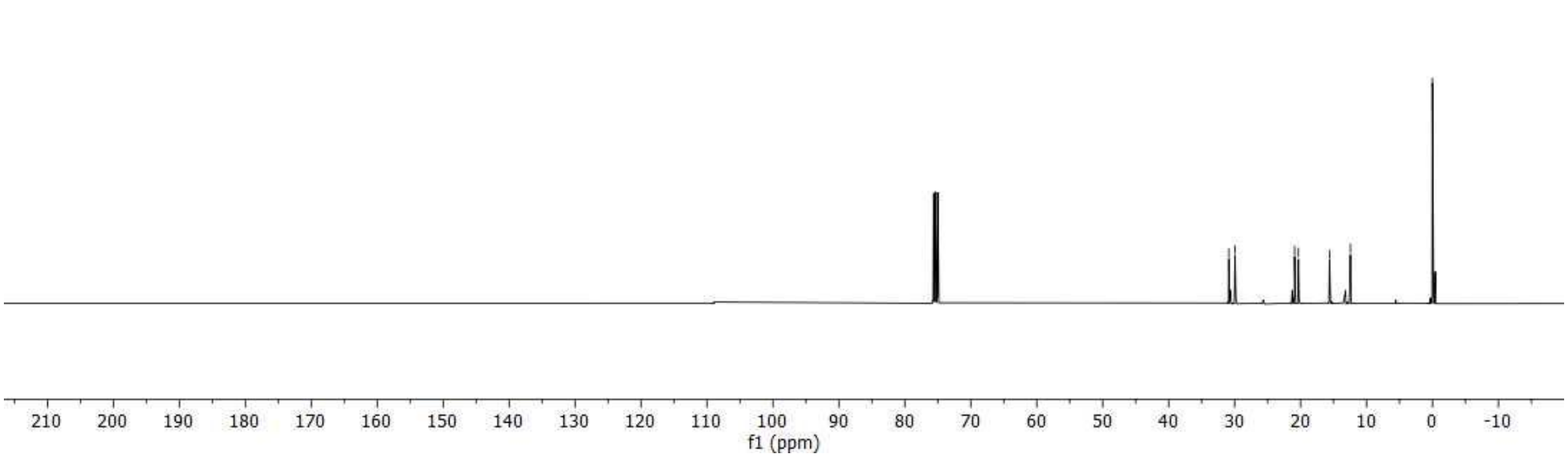

Figure SS74. ^{13}C NMR Spectrum of **29** (101 MHz, $CDCl_3$)

1,5-di-tert-butyl-3-hexyl-1,1,5,5-tetramethyltrisiloxane (30)

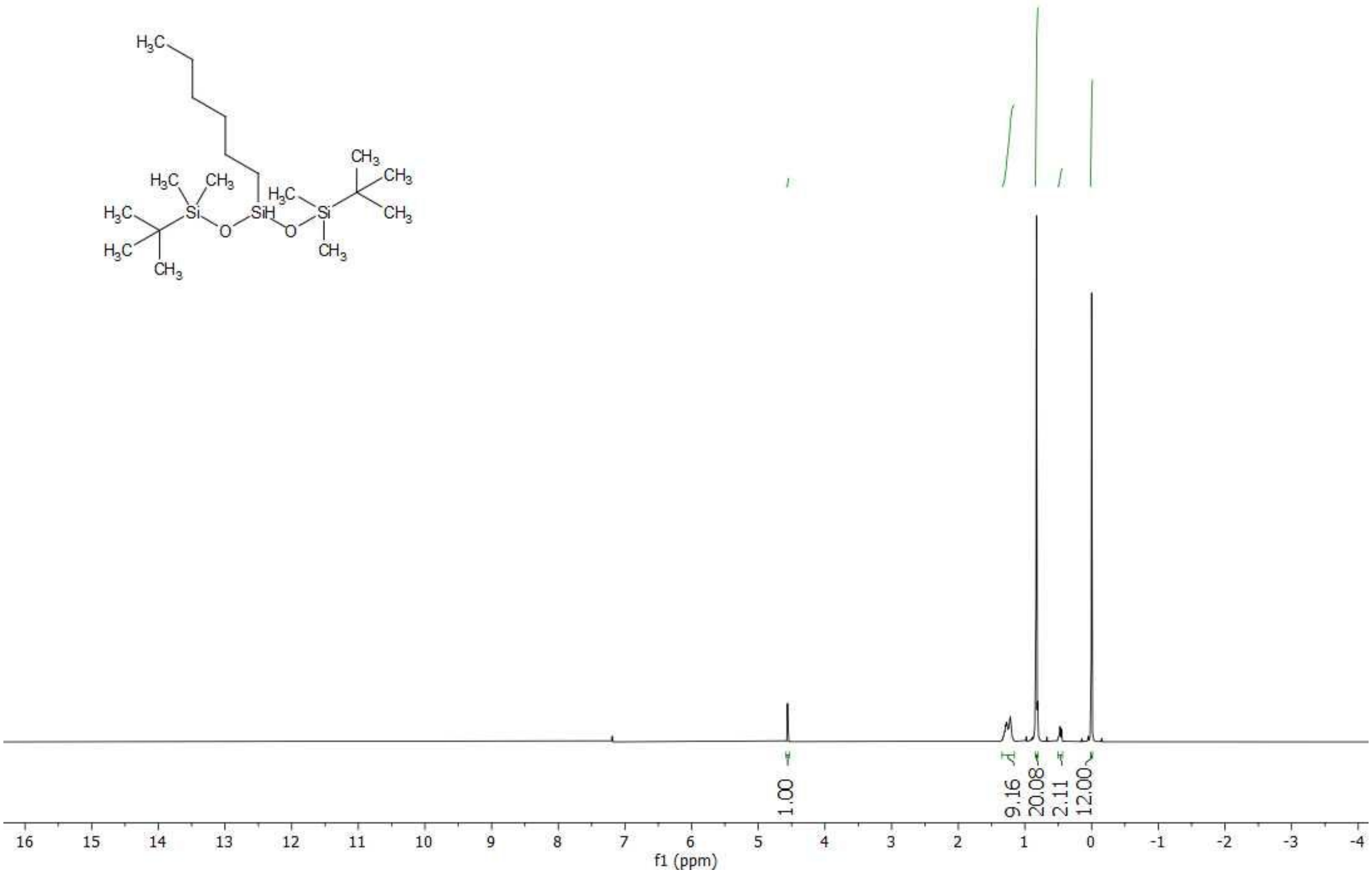

Figure SS75. ^{1}H NMR Spectrum of **30** (400 MHz, $CDCl_3$)

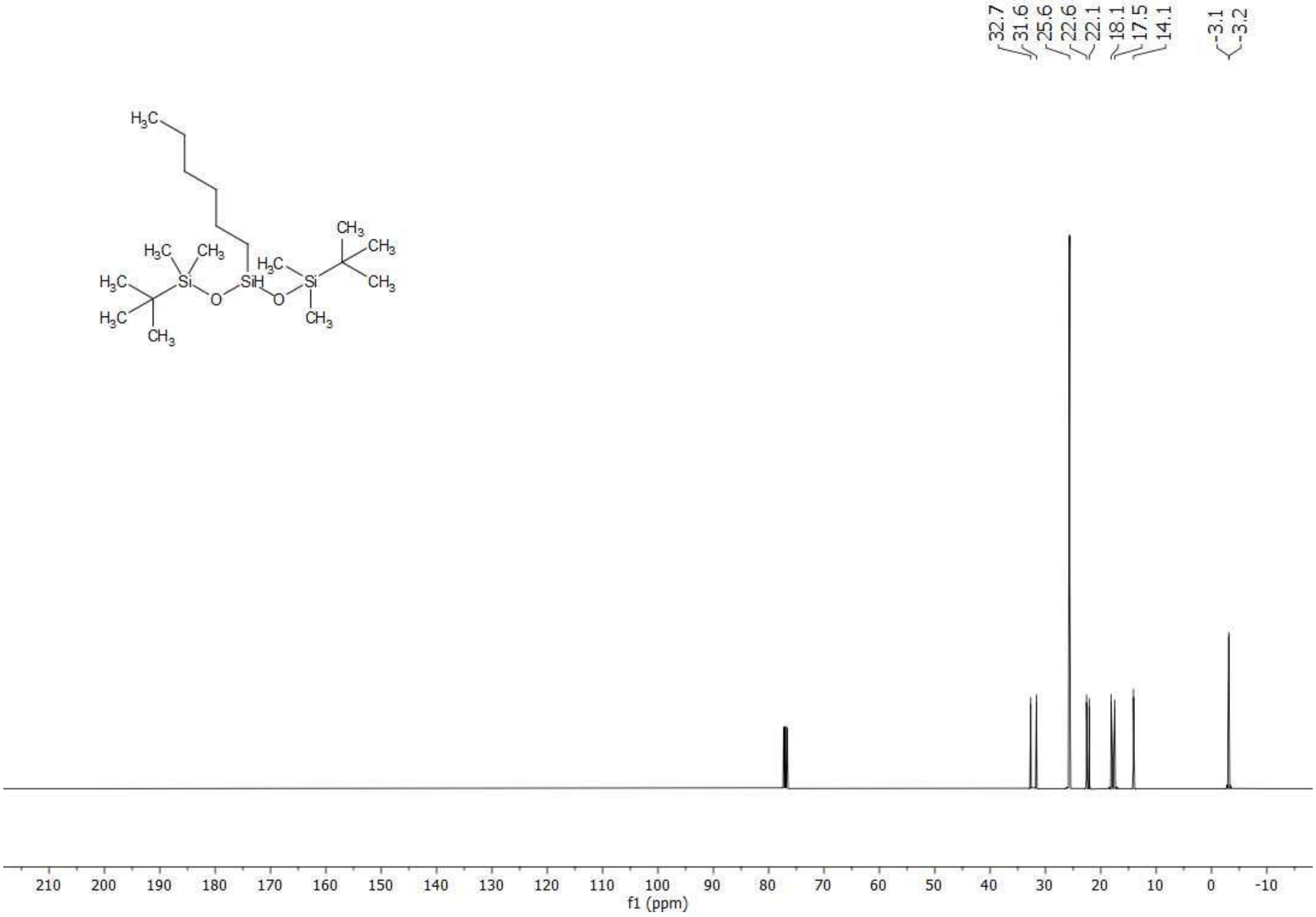

Figure SS76. ^{13}C NMR Spectrum of **30** (101 MHz, $CDCl_3$)

trimethoxy(phenyl)silane (31)

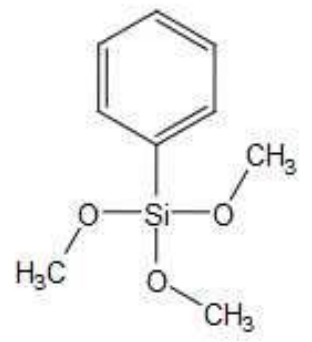

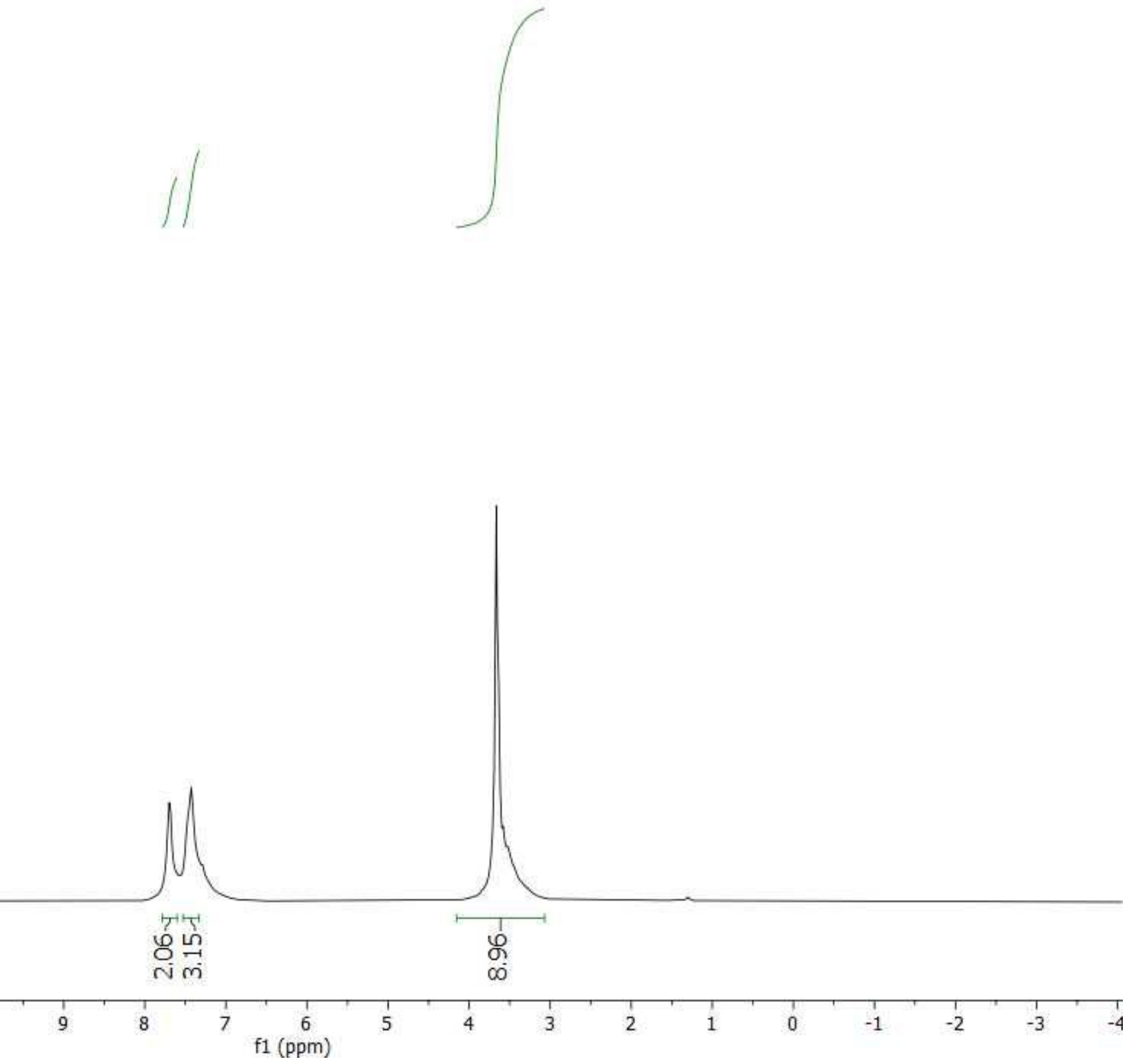

Figure SS77. ^{1}H NMR Spectrum of **31** (400 MHz, $CDCl_3$)

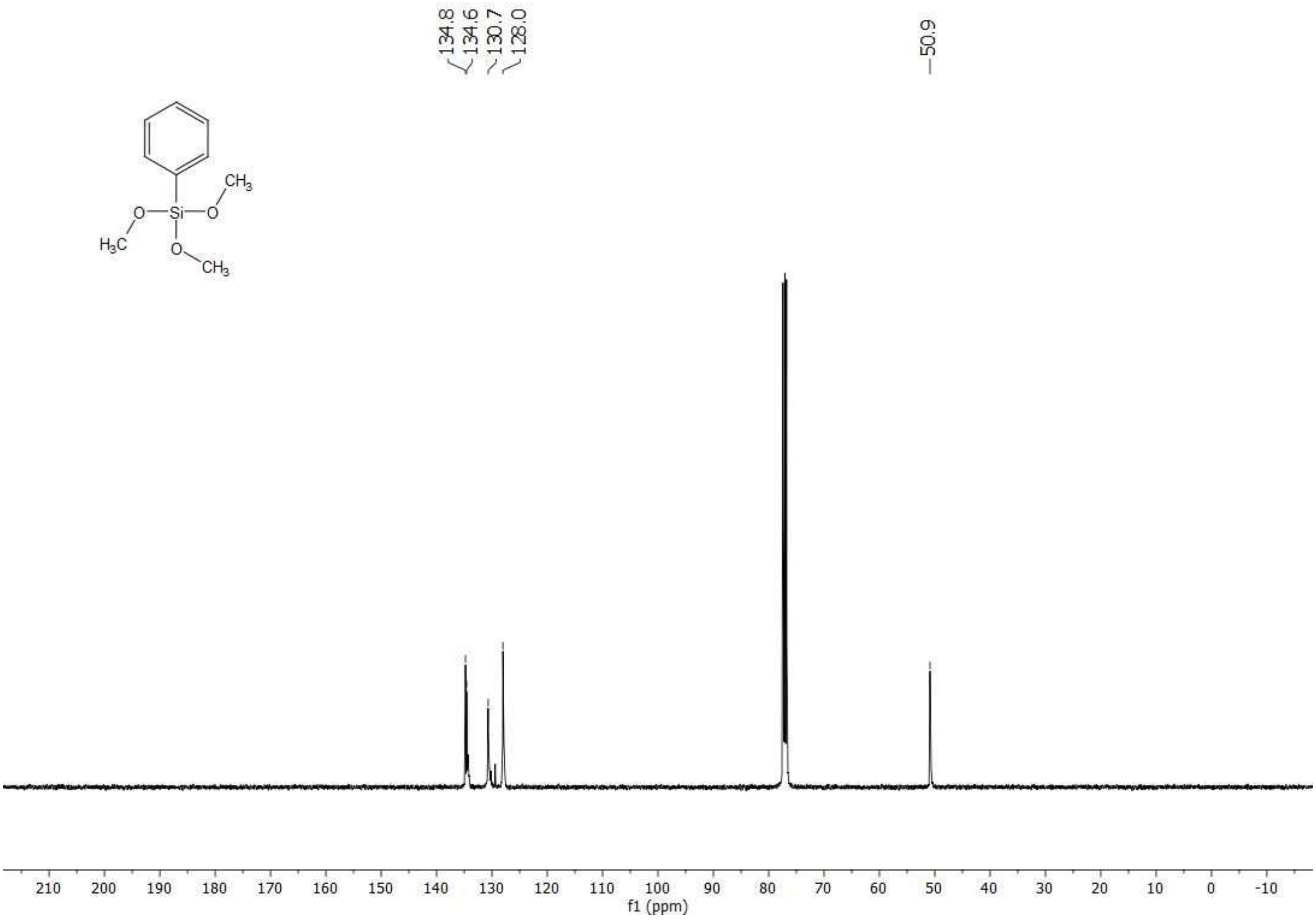

Figure SS78. ^{13}C NMR Spectrum of **31** (101 MHz, $CDCl_3$)

triethoxy(phenyl)silane (32)

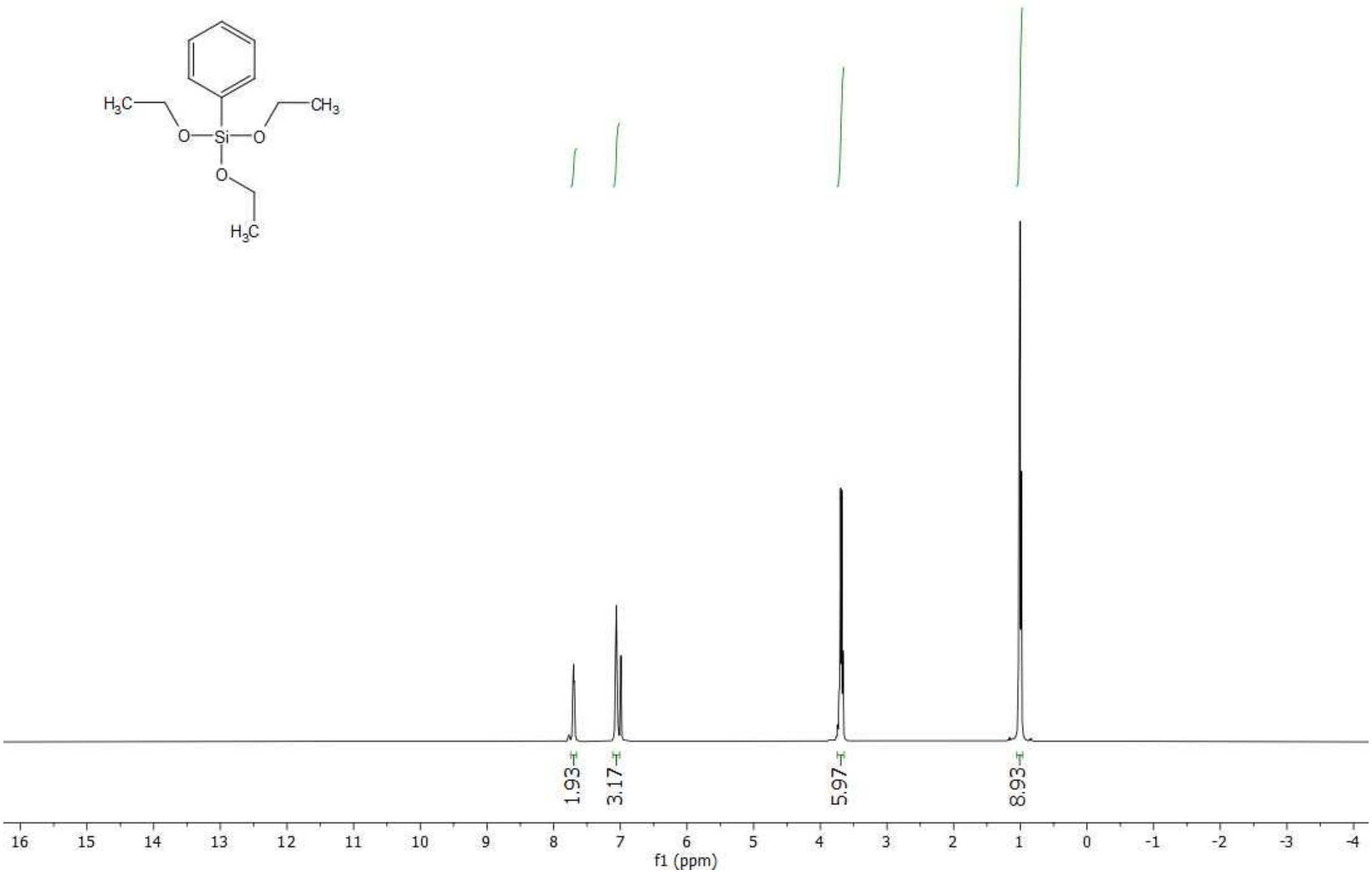

Figure SS79. ^{1}H NMR Spectrum of **32** (400 MHz, C_6D_6)

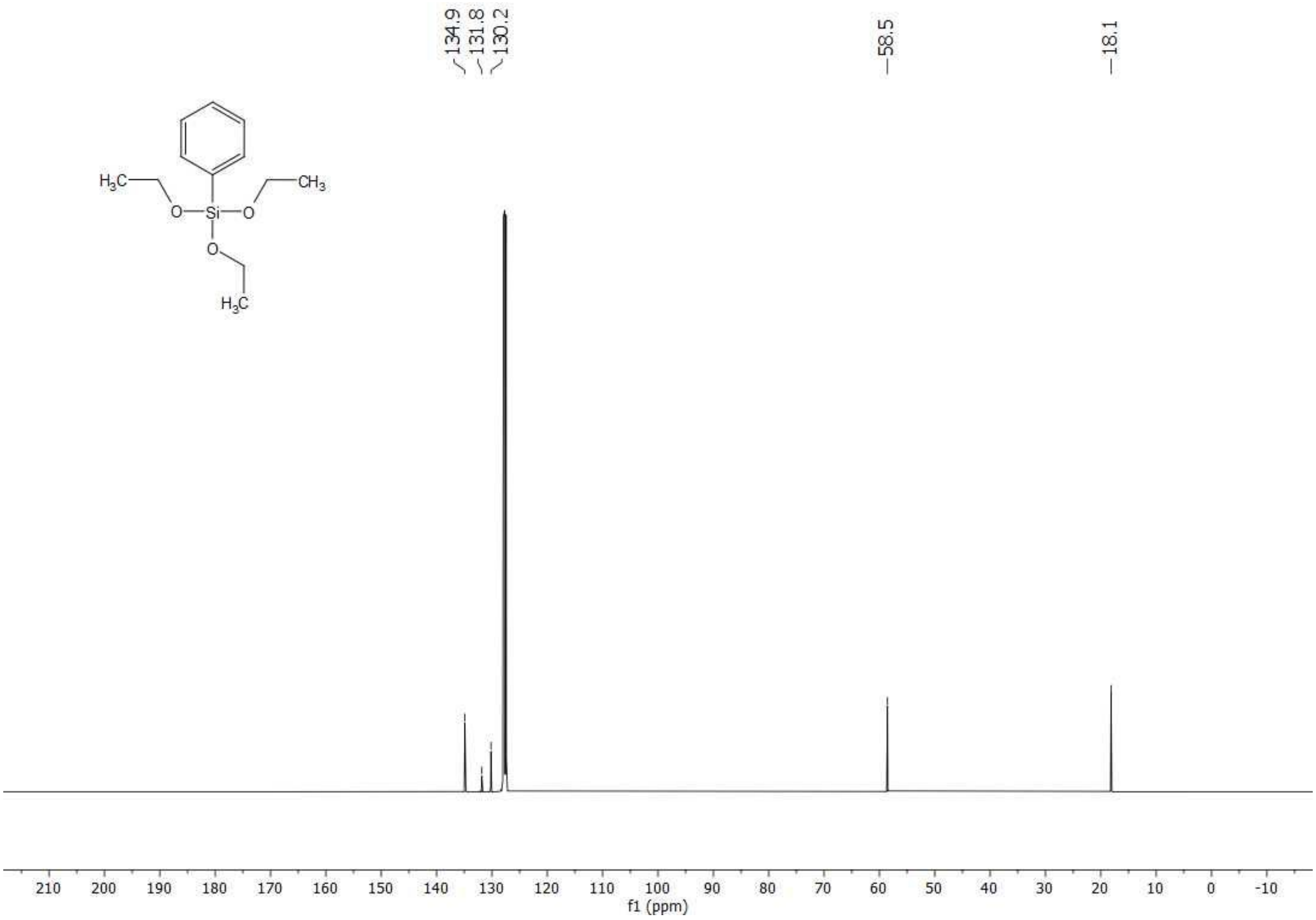

Figure SS80. ^{13}C NMR Spectrum of **32** (101 MHz, C_6D_6)

bis(pentan-3-yloxy)(phenyl)silane (33)

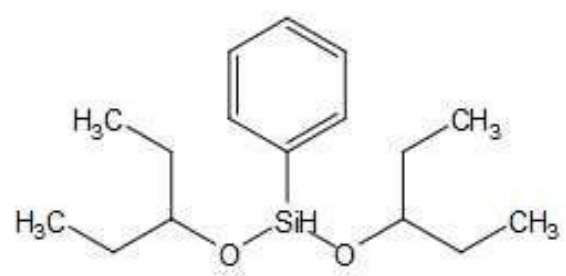

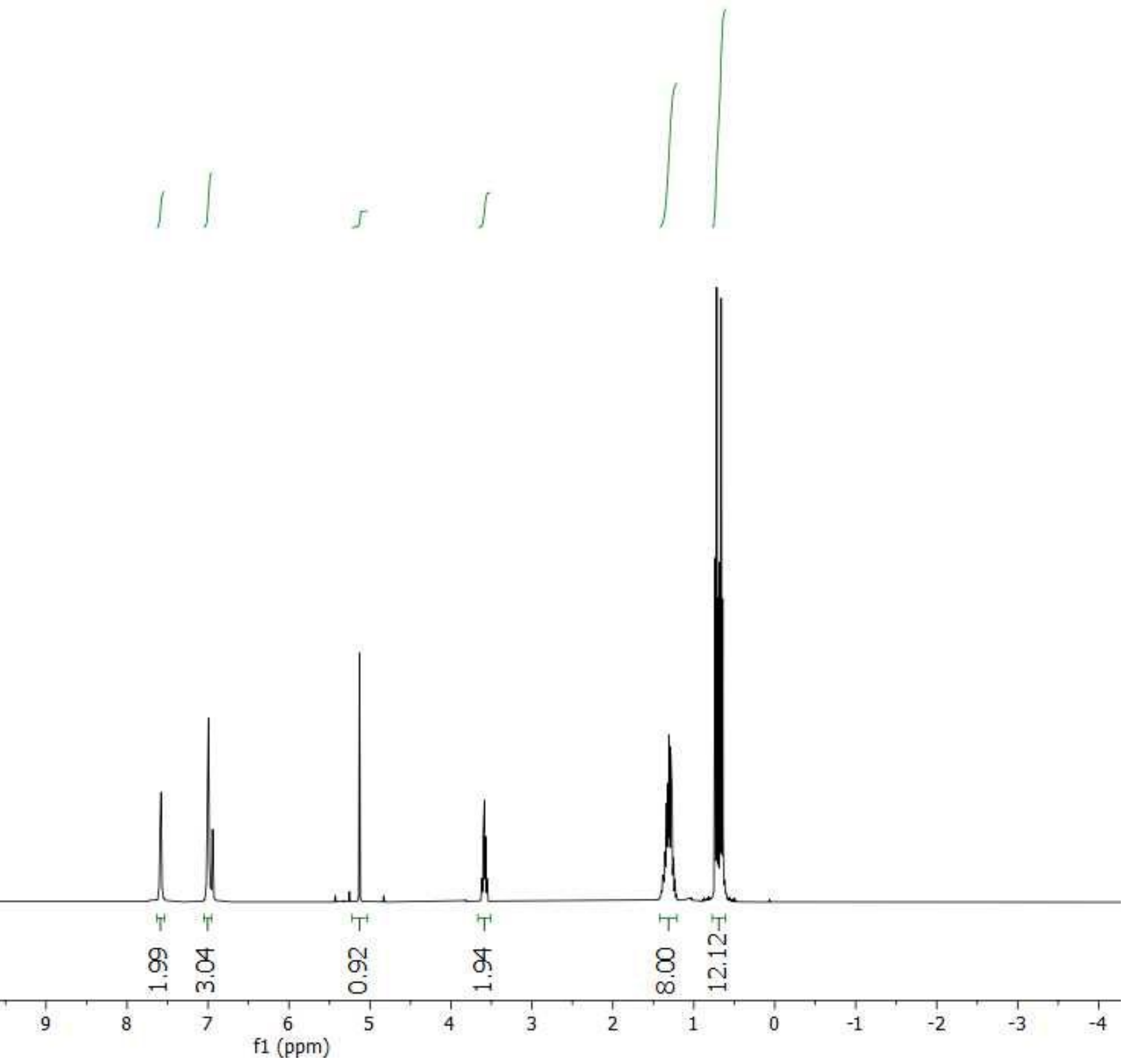

Figure SS81. ^{1}H NMR Spectrum of **33** (400 MHz, C_6D_6)

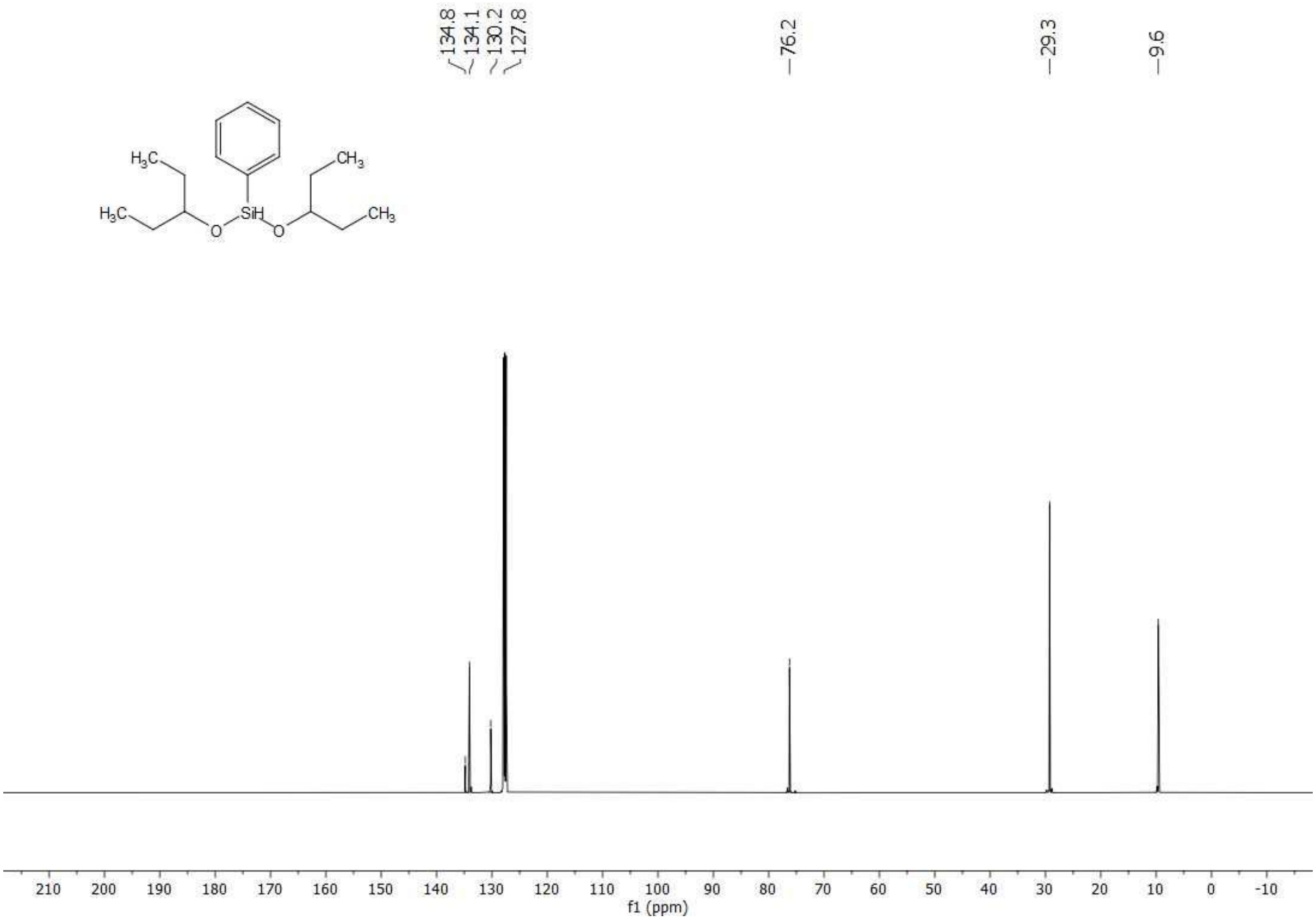

Figure SS82. ^{13}C NMR Spectrum of **33** (101 MHz, C_6D_6)

dimethoxydiphenylsilane (34)

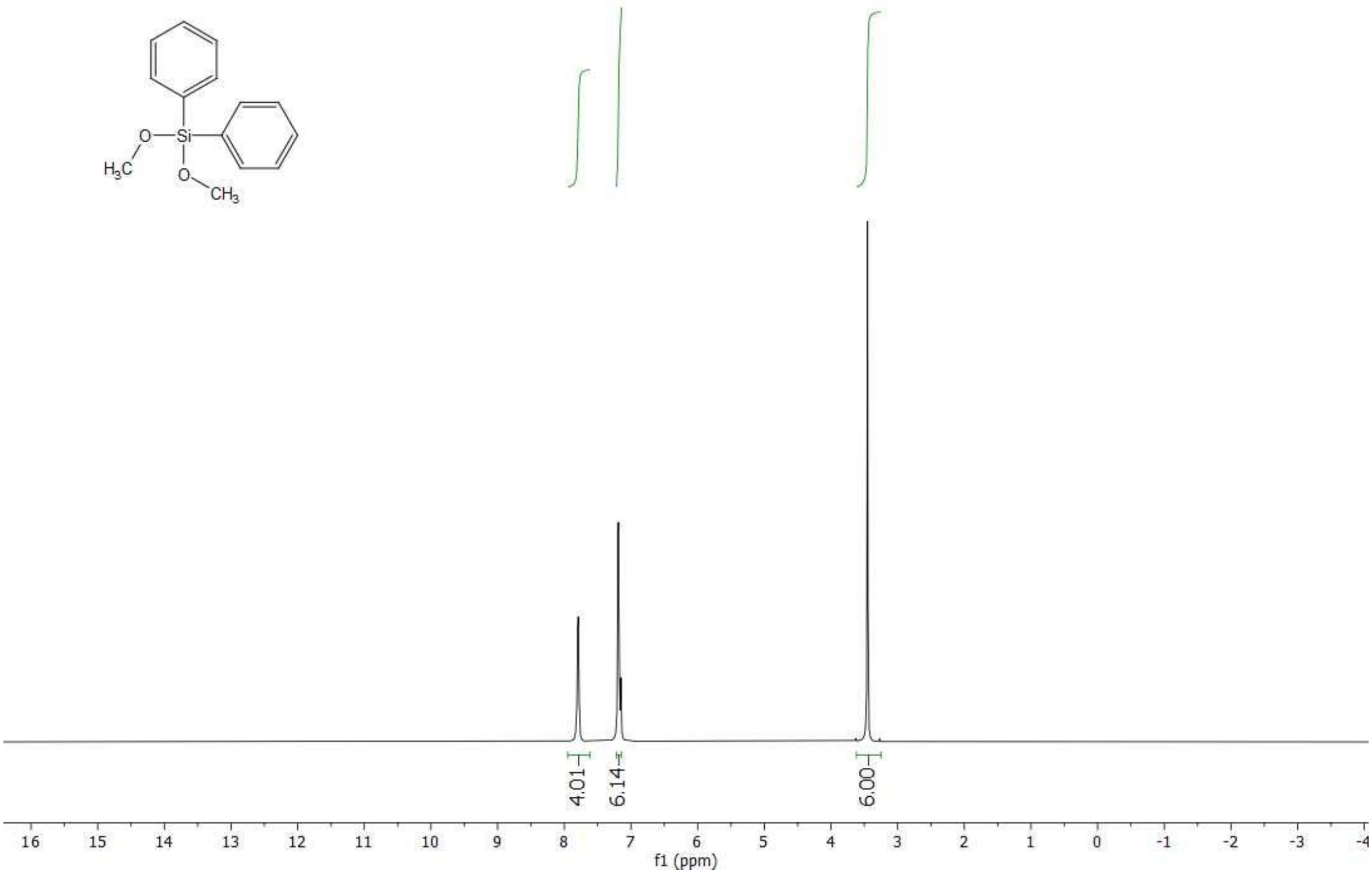

Figure SS83. ^{1}H NMR Spectrum of **34** (400 MHz, C_6D_6)

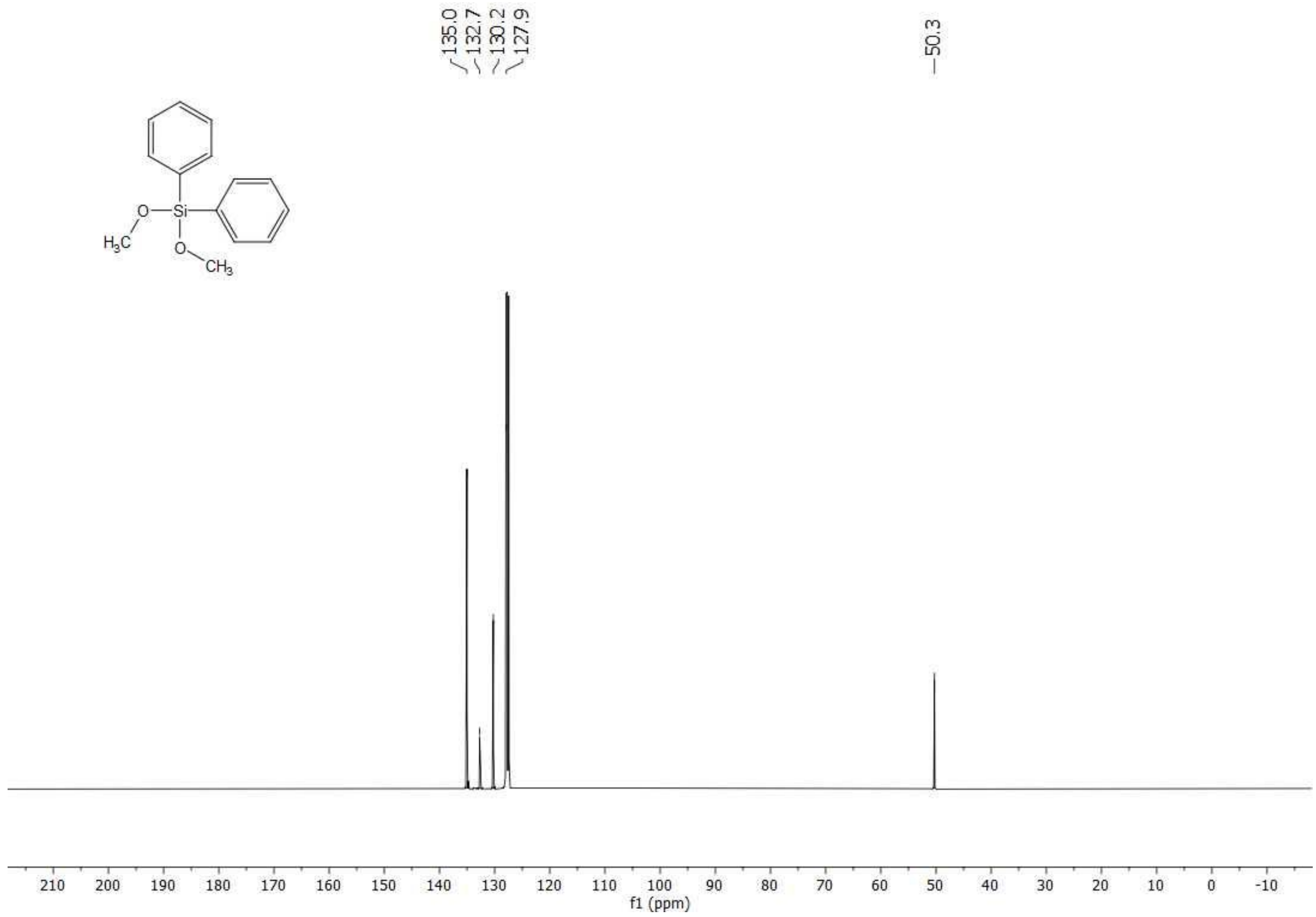

Figure SS84. ^{13}C NMR Spectrum of **34** (101 MHz, C_6D_6)

diethoxy(phenyl)silane (35)

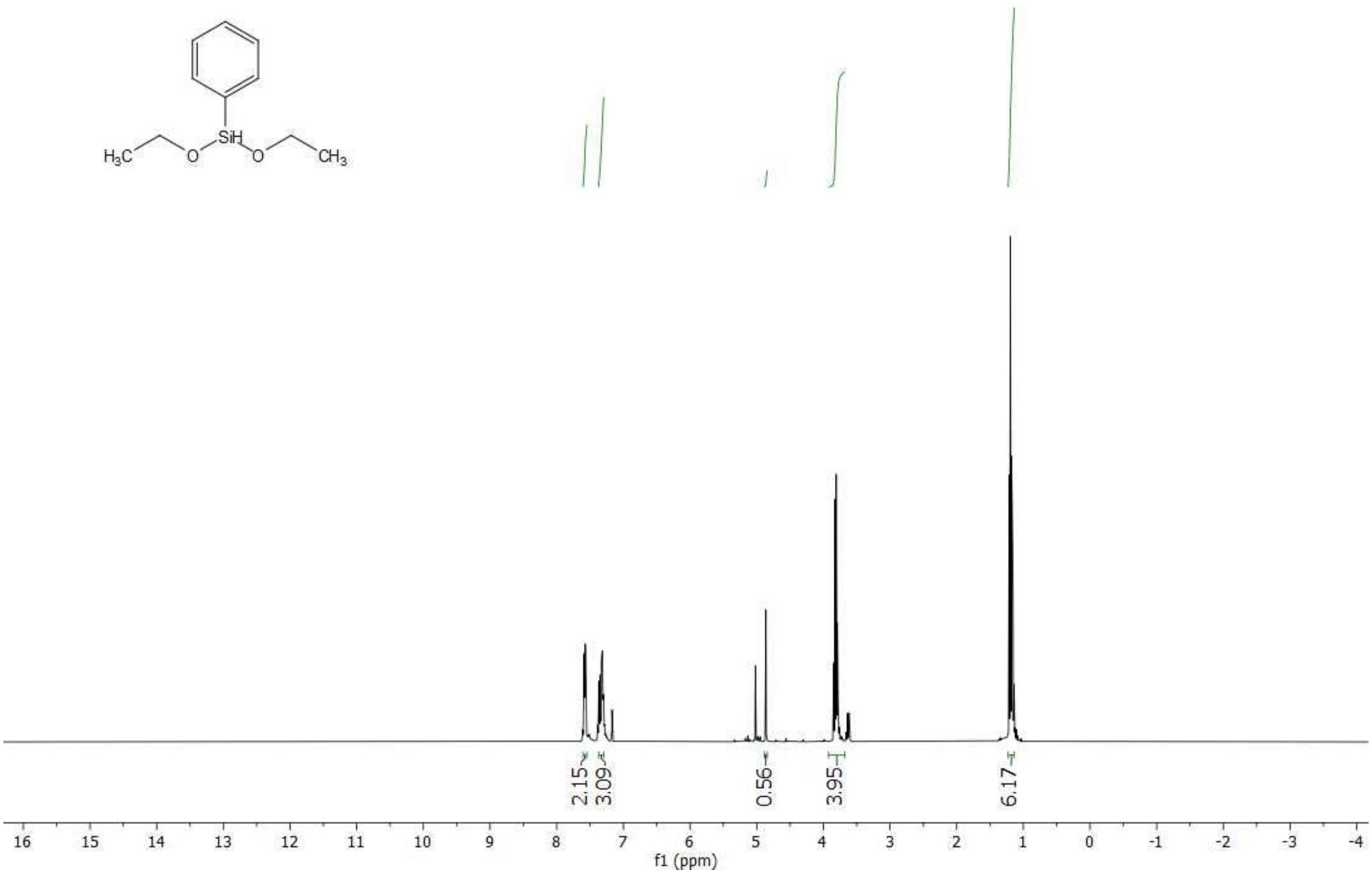

Figure SS85. ^{1}H NMR Spectrum of **35** (400 MHz, $CDCl_3$)

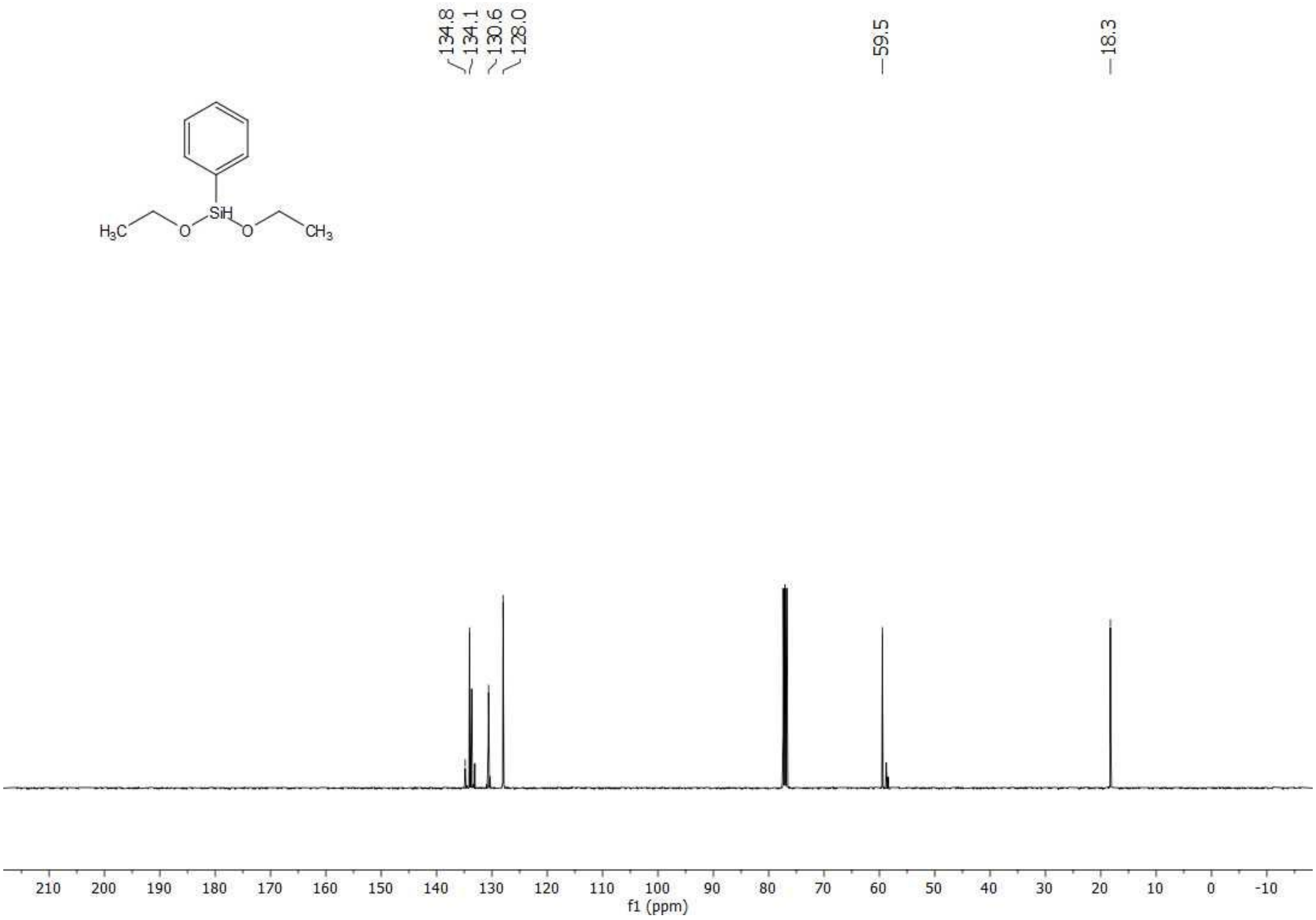

Figure SS86. ^{13}C NMR Spectrum of **35** (101 MHz, $CDCl_3$)

phenyl(prop-2-yn-1-yloxy)silane (36)

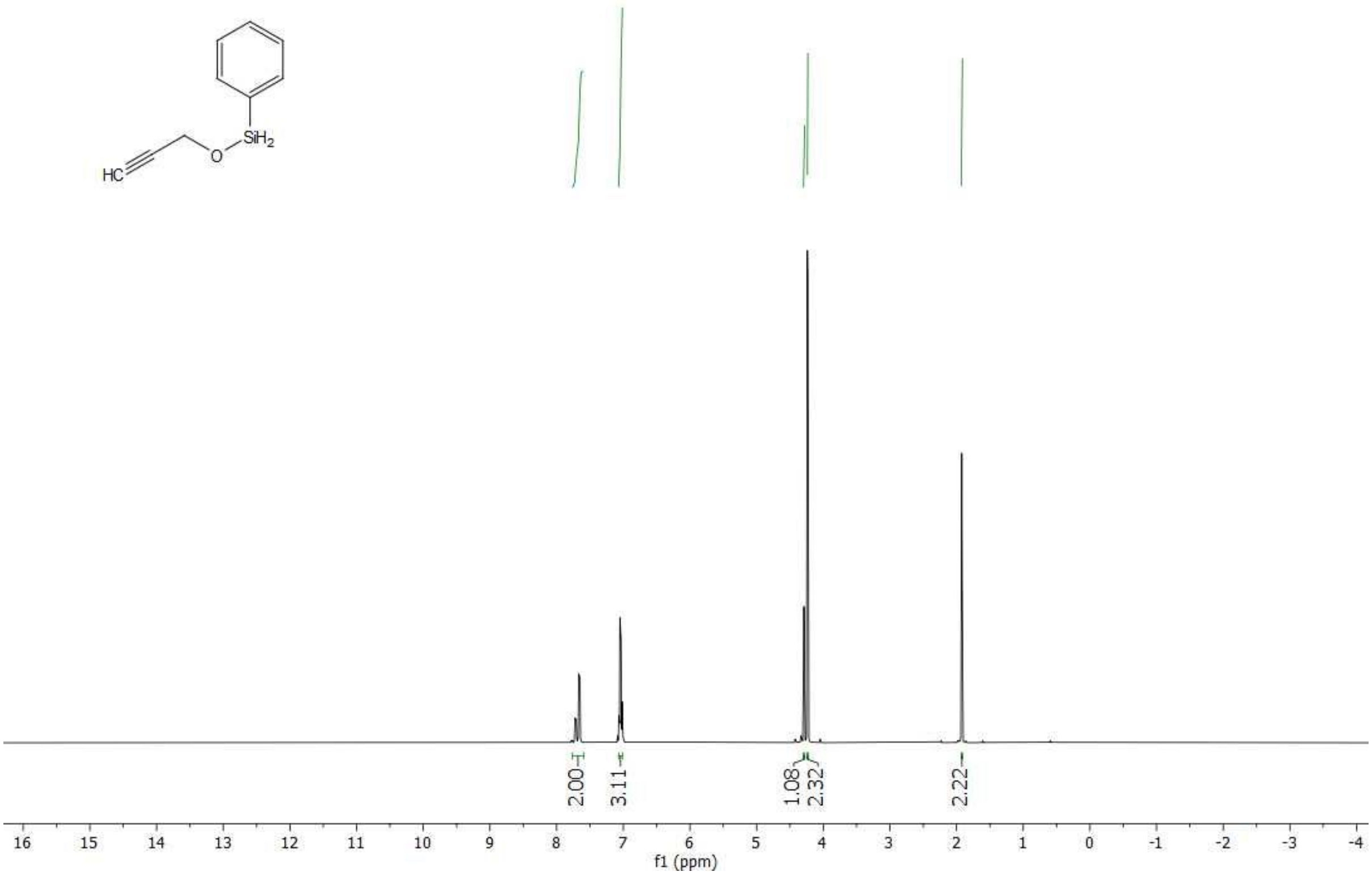

Figure SS87. ^{1}H NMR Spectrum of **36** (400 MHz, C_6D_6)

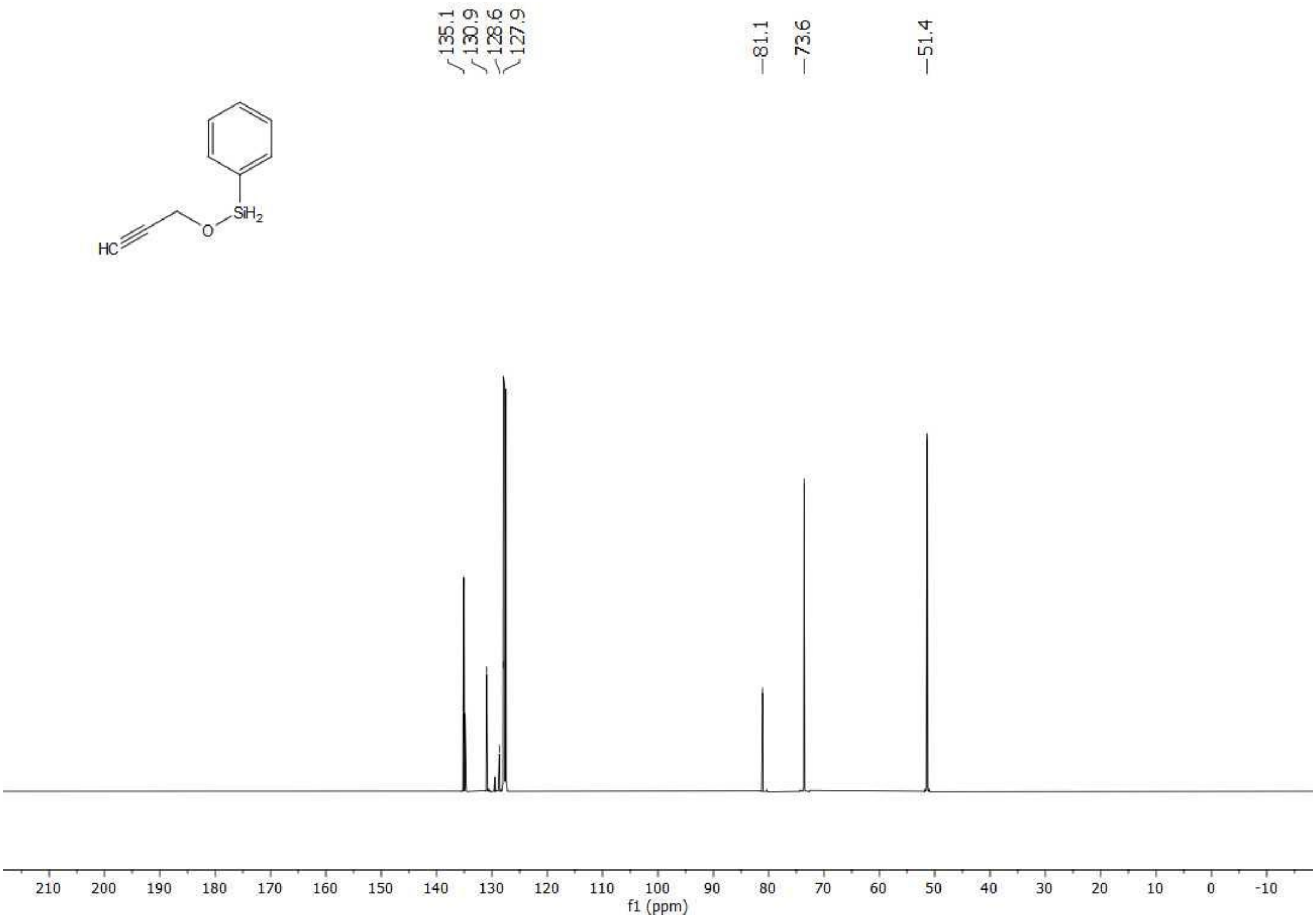

Figure SS88. ^{13}C NMR Spectrum of **36** (101 MHz, C_6D_6)

8-phenyl-4,7,9,12-tetraoxa-8-silapentadeca-1,14-diene (37)

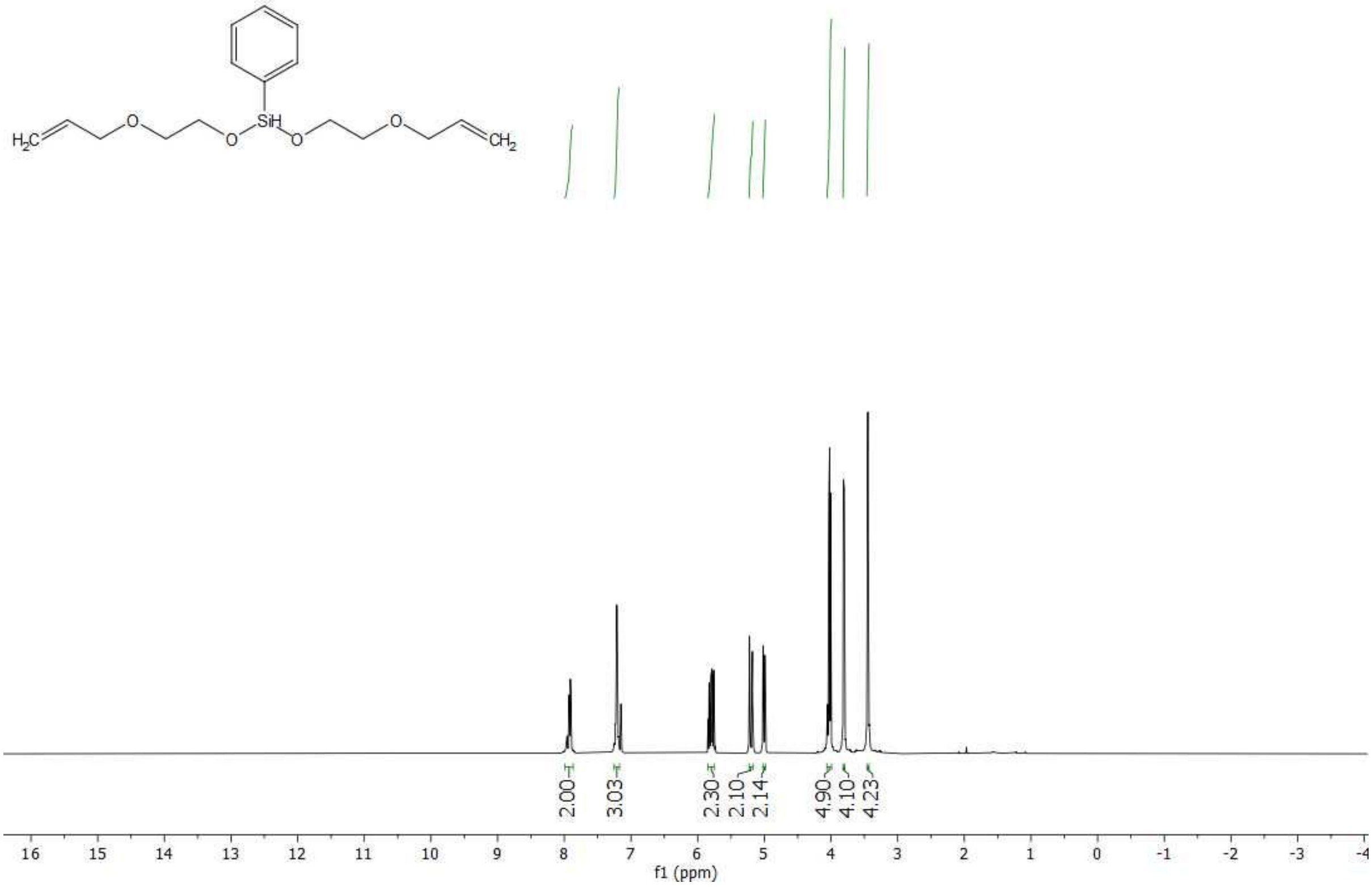

Figure SS89. ^{1}H NMR Spectrum of **37** (400 MHz, C_6D_6)

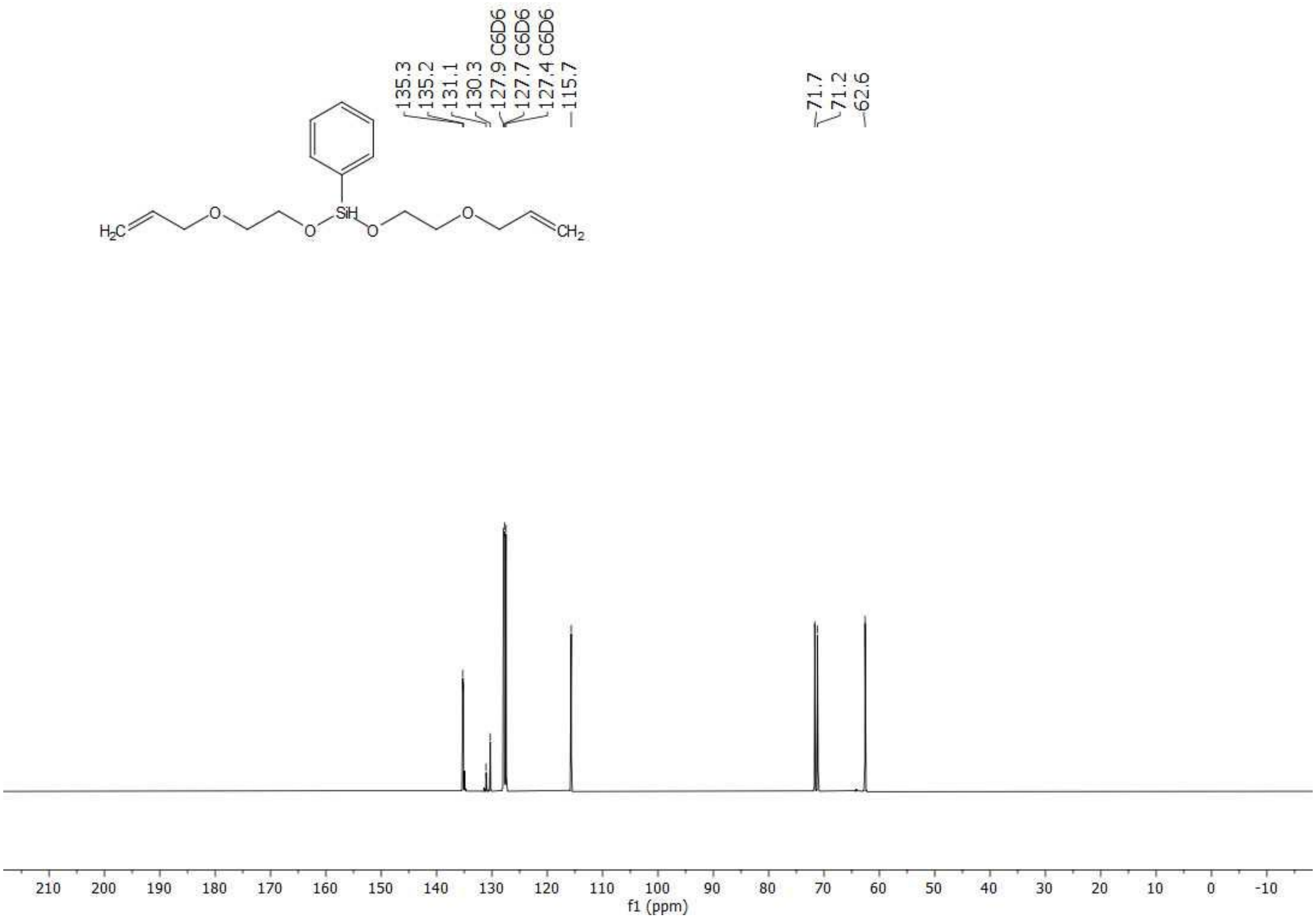

Figure SS90. ^{13}C NMR Spectrum of **37** (101 MHz, C_6D_6)

1,5-di-tert-butyl-3-decyl-1,1,5,5-tetramethyl-3-phenyltrisiloxane (38)

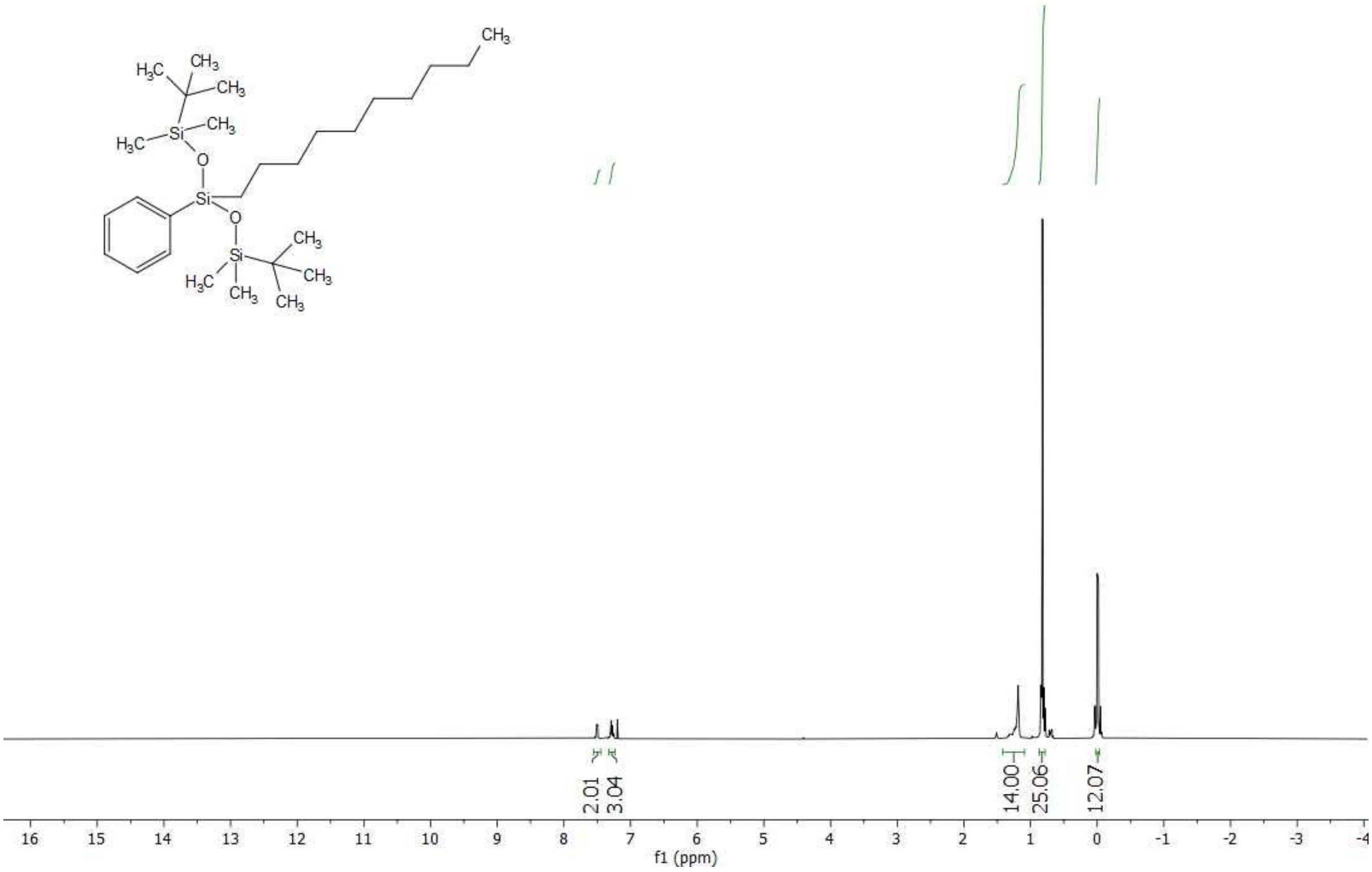

Figure SS91. ^{1}H NMR Spectrum of **38** (400 MHz, $CDCl_3$)

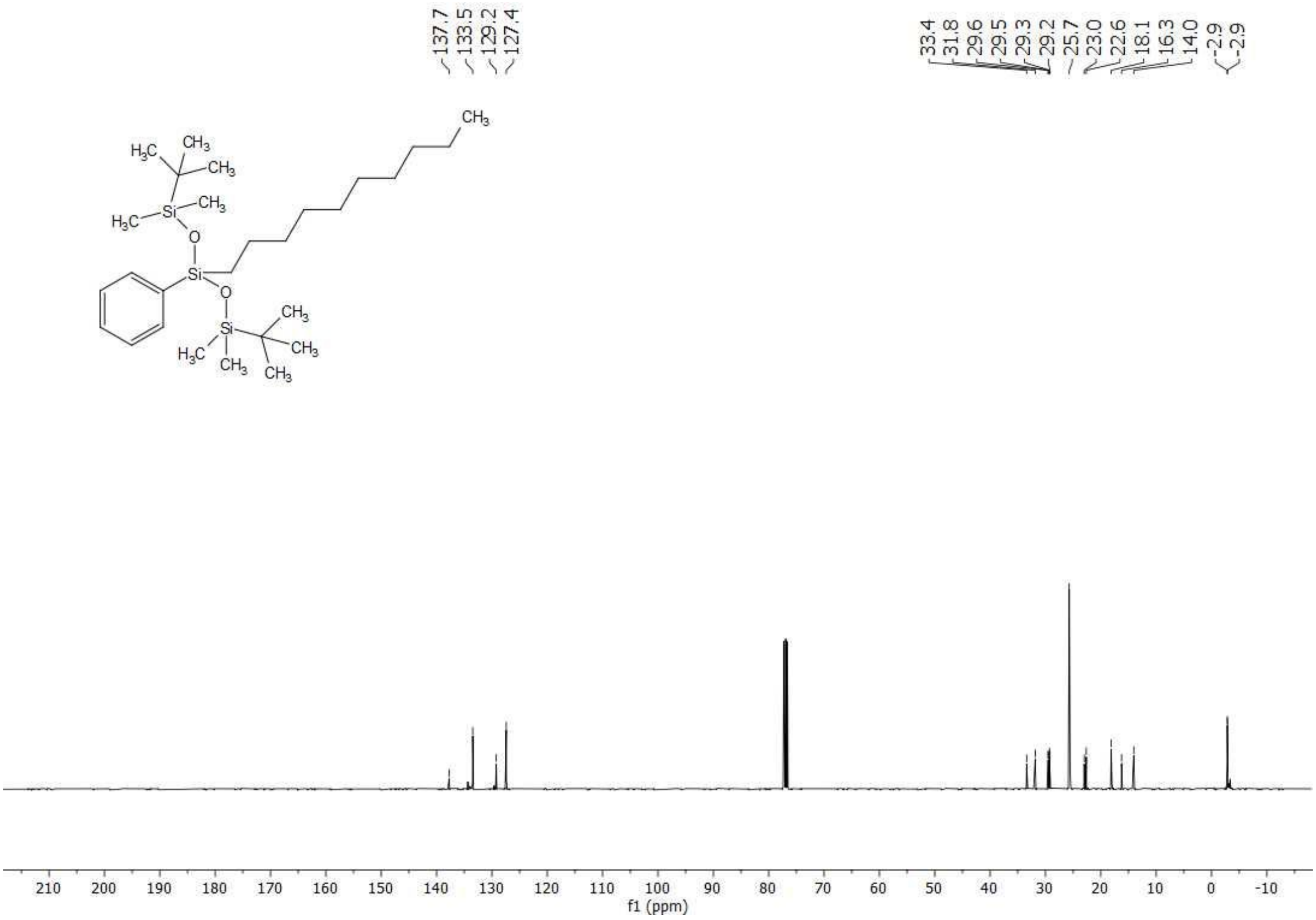

Figure SS92. ^{13}C NMR Spectrum of **38** (101 MHz, $CDCl_3$)

1-(tert-butyl)-1,1,3-trimethyl-3-phenyl-3-(2-(trimethoxysilyl)ethyl)disiloxanea (39)

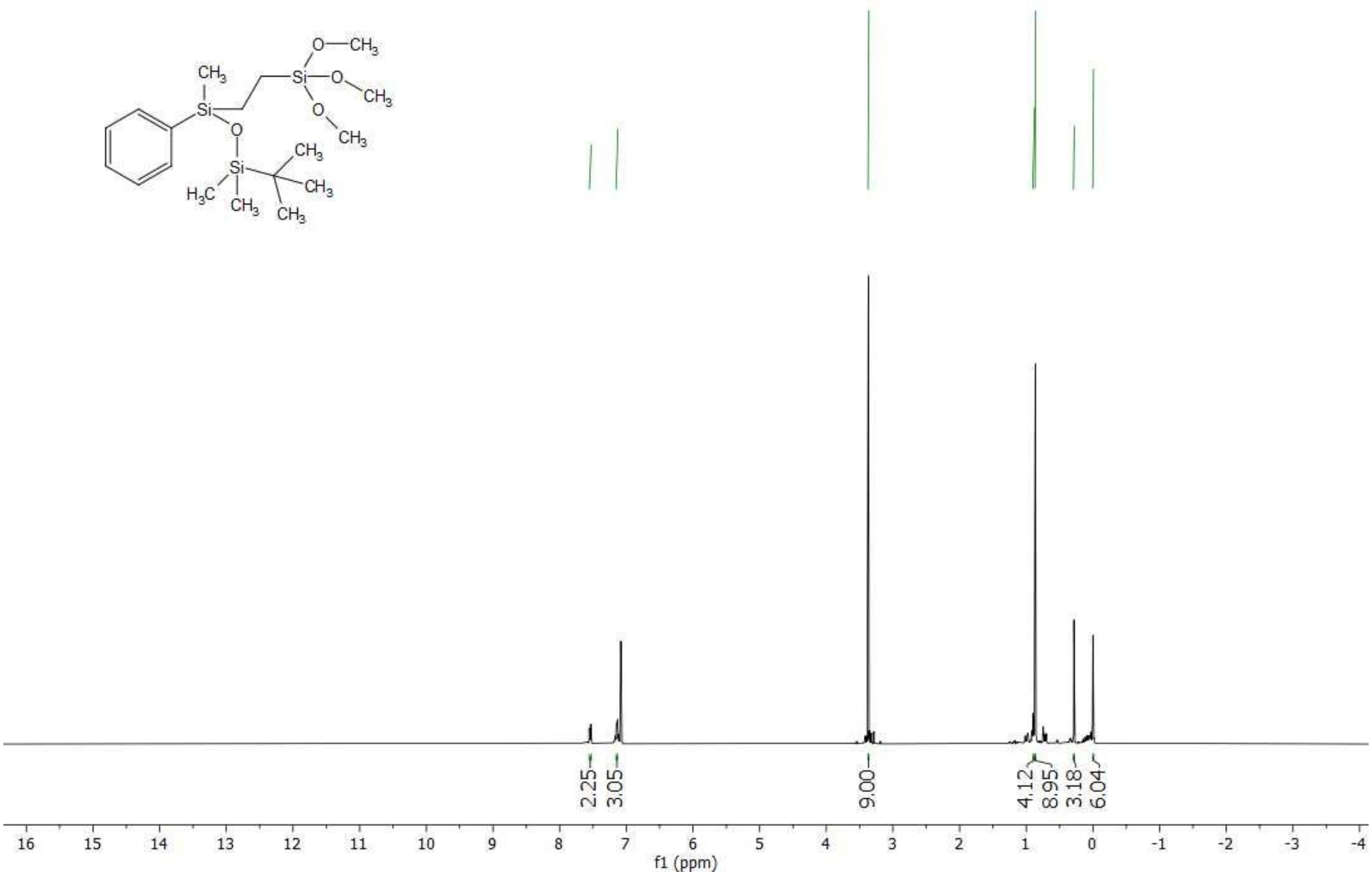

Figure SS93. ^{1}H NMR Spectrum of **39** (400 MHz, C_6D_6)

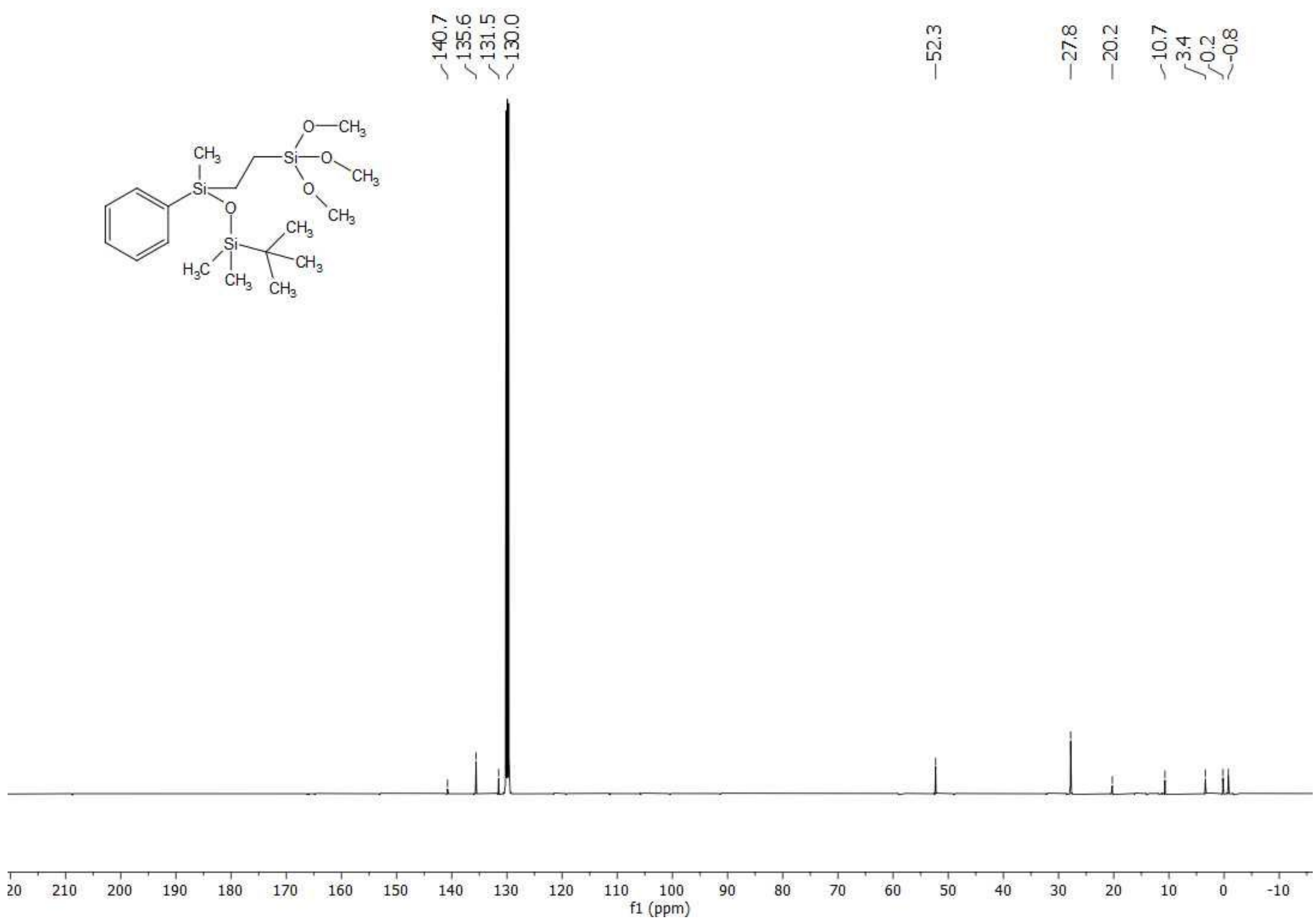

Figure SS94. ^{13}C NMR Spectrum of **39** (101 MHz, C_6D_6)

REFERENCES

(1) Stachowiak-Dłużyńska, H.; Gruszczyński, M.; Kubicki, M.; Hreczycho, G. Pt(II) Complexes Bearing P,N-Donor Ligands as Catalysts in Chemoselective Hydrosilylation, Hydrogermylation, and Hydroboration of Terminal Alkenes. *J. Catal.* **2024**, *433*, 115494.

(2) Gruszczyński, M.; Lewandowski, D.; Kuciński, K.; Kubicki, M.; Hreczycho, G. From Alkenylboronates to Enynes: Ligand and Activator-Driven Pathways in Nickel-Catalyzed Alkyne Functionalization. *J. Catal.* ***2026**, 458, 116862*

(3) Doyle, M. P.; High, K. G.; Bagheri, V.; Pieters, R. J.; Lewis, P. J.; Pearson, M. M. Rhodium(II) Perfluorobutyrate Catalyzed Silane Alcoholysis. A Highly Selective Route to Silyl Ethers. *J. Org. Chem.* **1990**, *55* (25), 6082–6086.

(4) Sudhakar, S.; Luh, T.-Y. Norbornadiene as an Efficient Hydrogen Scavenger for the Palladium-Catalyzed Conversion of Hydrosilanes to Alkoxysilanes. *J. Org. Chem.* **2002**, *67* (19), 6860–6862.

(5) Maifeld, S. V.; Miller, R. L.; Lee, D. Activation of Silanes by Grubbs' Carbene Complex Cl2(PCy3)2Ru�CHPh: Dehydrogenative Condensation of Alcohols and Hydrosilylation of Carbonyls. *Tetrahedron Lett.* **2002**, *43* (36), 6363–6366.

(6) Kaźmierczak, J.; Kuciński, K.; Lewandowski, D.; Hreczycho, G. Ru-Catalyzed Dehydrogenative Silylation of POSS-Silanols with Hydrosilanes: Its Introduction to One-Pot Synthesis. *Inorg. Chem.* **2019**, *58* (2), 1201–1207.

(7) Luo, X. L.; Crabtree, R. H. Homogeneous Catalysis of Silane Alcoholysis via Nucleophilic Attack by the Alcohol on an Ir(.Eta.2-HSiR3) Intermediate Catalyzed by [IrH2S2(PPh3)2]SbF6 (S = Solvent). *J. Am. Chem. Soc.* **1989**, *111* (7), 2527–2535.

(8) Satoh, Y.; Igarashi, M.; Sato, K.; Shimada, S. Highly Selective Synthesis of Hydrosiloxanes by Au-Catalyzed Dehydrogenative Cross-Coupling Reaction of Silanols with Hydrosilanes. *ACS Catal.* **2017**, *7* (3), 1836–1840.

(9) Ito, H.; Takagi, K.; Miyahara, T.; Sawamura, M. Gold(I)−Phosphine Catalyst for the Highly Chemoselective Dehydrogenative Silylation of Alcohols. *Org. Lett.* **2005**, *7* (14), 3001–3004.

(10) Safa, K. D.; Tofangdarzadeh, S.; Hassanpour, A. Facile Route to Synthesis of Functionalised Poly(Methylalkoxy)Siloxane under Mild and Aerobic Conditions in the Presence of Platinum Catalysts. *J. Organomet. Chem.* **2009**, *694* (25), 4107–4115.

(11) Reis, P. M.; Royo, B. Perrhenic Acid as Catalyst for Hydrosilylation of Aldehydes and Ketones and Dehydrogenative Silylation of Alcohols. *Catal. Commun.* **2007**, *8* (7), 1057–1059.

(12) Gregg, B. T.; Cutler, A. R. Manganese Carbonyl Bromide-Catalyzed Alcoholysis of the Monohydrosilane HSiMe2Ph. *Organometallics* **1994**, *13* (3), 1039–1043.

(13) Vijjamarri, S.; Chidara, V. K.; Rousova, J.; Du, G. Dehydrogenative Coupling of Alcohols and Carboxylic Acids with Hydrosilanes Catalyzed by a Salen–Mn(v) Complex. *Catal. Sci. Technol.* **2016**, *6* (11), 3886–3892.

(14) Li, T.; Zhang, H.; Chan, A. S. C.; Meng, S.-S. Manganese-Catalyzed Dehydrogenative Coupling of Silanes and Hydroxyl Compound Controlled by Phosphine Oxide. *CR-PHYS-SC* **2023**, *4* (3).

(15) Reuter, M. B.; Cibuzar, M. P.; Hammerton, J.; Waterman, R. Photoactivated Silicon–Oxygen and Silicon–Nitrogen Heterodehydrocoupling with a Commercially Available Iron Compound. *Dalton Trans.* **2020**, *49* (9), 2972–2978.

(16) Gasperini, D.; King, A. K.; Coles, N. T.; Mahon, M. F.; Webster, R. L. Seeking Heteroatom-Rich Compounds: Synthetic and Mechanistic Studies into Iron Catalyzed Dehydrocoupling of Silanes. *ACS Catal.* **2020**, *10* (11), 6102–6112.

(17) Takeshita, T.; Sato, K.; Nakajima, Y. Selective Hydrosiloxane Synthesis via Dehydrogenative Coupling of Silanols with Hydrosilanes Catalysed by Fe Complexes Bearing a Tetradentate PNNP Ligand. *Dalton Trans.* **2018**, *47* (47), 17004–17010.

(18) Pattanaik, S.; Gunanathan, C. Cobalt-Catalyzed Selective Synthesis of Disiloxanes and Hydrodisiloxanes. *ACS Catal.* **2019**, *9* (6), 5552–5561.

(19) Szafoni, E.; Kuciński, K.; Hreczycho, G. Cobalt-Catalyzed Dehydrogenative Cross-Coupling Reaction: Selective Access to Dihydrosiloxanes, Hydrosiloxanes and Functionalized Silsesquioxanes. *J. Catal.* **2023**, *423*, 1–9.
(20) Szafoni, E.; Lewandowski, D.; Gruszczyński, M.; Broniarz, K.; Stachowiak-Dłużyńska, H.; Kuciński, K.; Hreczycho, G. Streamlining Si–O Bond Formation through Cobalt-Catalyzed Dehydrocoupling. *Chem. Commun.* **2024**, *60* (78), 10886–10889.
(21) Szafoni, E.; Kuciński, K.; Hreczycho, G. Cobalt-Catalyzed Synthesis of Silyl Ethers via Cross-Dehydrogenative Coupling between Alcohols and Hydrosilanes. *Green Chem. Lett. Rev.* **2022**, *15* (3), 757–764.
(22) Ohta, H.; Miyoshi, N.; Sakata, Y.; Okamoto, Y.; Hayashi, M.; Watanabe, Y. A *N*-Heterocyclic Carbene Ni(II) Complex Bearing Bis(Cyclopentadienyl) Ligands as a Precatalyst for the Dehydrogenative Silylation of Alcohols with Hydrosilanes. *Tetrahedron Lett.* **2015**, *56* (22), 2910–2912.
(23) Kaźmierczak, J.; Hreczycho, G. Copper(II) Triflate-Mediated Synthesis of Functionalized Silsesquioxanes via Dehydrogenative Coupling of POSS Silanols with Hydrosilanes. *Dalton Trans.* **2019**, *48* (19), 6341–6346.
(24) Gunji, Y.; Yamashita, Y.; Ikeno, T.; Yamada, T. Convenient and Selective Preparation of Mono-Alkoxyphenylsilanes from Phenylsilane and Alcohols. *Chem. Lett.* **2006**, *35* (7), 714–715.
(25) Patnaik, S.; Kanbur, U.; Ellern, A.; Sadow, A. D. Hydrosilane σ-Adduct Intermediates in an Adaptive Zinc-Catalyzed Cross-Dehydrocoupling of Si−H and O−H Bonds. *Chem. Eur. J.* **2021**, *27* (40), 10428–10436.
(26) Sheldrick, G. M. SHELXT – Integrated Space-Group and Crystal-Structure Determination. *Acta Cryst. A* **2015**, *71* (1), 3–8.
(27) Sheldrick, G. M. Crystal Structure Refinement with SHELXL. *Acta Cryst. C* **2015**, *71* (1), 3–8.
(28) Stachowiak, H.; Kuciński, K.; Kallmeier, F.; Kempe, R.; Hreczycho, G. Cobalt-Catalyzed Dehydrogenative C−H Silylation of Alkynylsilanes. *Chem. Eur. J.* **2022**, *28* (1), e202103629.
(29) Morris, L.; Hales, J. J.; Trudeau, M. L.; Georgiev, P.; Embs, J. P.; Eckert, J.; Kaltsoyannis, N.; Antonelli, D. M. A Manganese Hydride Molecular Sieve for Practical Hydrogen Storage under Ambient Conditions. *Energy Environ. Sci.* **2019**, *12* (5), 1580–1591.
(30) Wang, X.; Andrews, L. Matrix Infrared Spectra and Density Functional Theory Calculations of Manganese and Rhenium Hydrides. *J. Phys. Chem. A* **2003**, *107* (20), 4081–4091.
(31) Wei, D.; Bruneau-Voisine, A.; Chauvin, T.; Dorcet, V.; Roisnel, T.; Valyaev, D. A.; Lugan, N.; Sortais, J.-B. Hydrogenation of Carbonyl Derivatives Catalysed by Manganese Complexes Bearing Bidentate Pyridinyl-Phosphine Ligands. *Adv. Synth. Catal.* **2018**, *360* (4), 676–681.
(32) Satoh, Y.; Igarashi, M.; Sato, K.; Shimada, S. Highly Selective Synthesis of Hydrosiloxanes by Au-Catalyzed Dehydrogenative Cross-Coupling Reaction of Silanols with Hydrosilanes. *ACS Catal.* **2017**, *7* (3), 1836–1840.
(33) Prieto-Pascual, U.; Rodríguez-Diéguez, A.; Freixa, Z.; Huertos, M. A. Tailor-Made Synthesis of Hydrosilanols, Hydrosiloxanes, and Silanediols Catalyzed by Di-Silyl Rhodium(III) and Iridium(III) Complexes. *Inorg. Chem.* **2023**, *62* (7), 3095–3105.
(34) Szafoni, E.; Kuciński, K.; Hreczycho, G. Cobalt-Driven Cross-Dehydrocoupling of Silanes and Amines for Silylamine Synthesis. *ChemCatChem* **2024**, *16* (15), e202400143.
(35) Takeshita, T.; Sato, K.; Nakajima, Y. Selective Hydrosiloxane Synthesis via Dehydrogenative Coupling of Silanols with Hydrosilanes Catalysed by Fe Complexes Bearing a Tetradentate PNNP Ligand. *Dalton Trans.* **2018**, *47* (47), 17004–17010.
(36) Miura, H.; Masaki, Y.; Fukuta, Y.; Shishido, T. Silylation of Aryl Chlorides by Bimetallic Catalysis of Palladium and Gold on Alloy Nanoparticles. *Adv. Synth. Catal.* **2020**, *362* (13), 2642–2650.
(37) Cruz, T. F. C.; Veiros, L. F.; Gomes, P. T. Hydrosilylation of Aldehydes and Ketones Catalyzed by a 2-Iminopyrrolyl Alkyl-Manganese(II) Complex. *Inorg. Chem.* **2022**, *61* (2), 1195–1206.

(38) Khalimon, A. Y.; Simionescu, R.; Nikonov, G. I. Catalytic and Stoichiometric Reactivity of β-Silylamido Agostic Complex of Mo: Intermediacy of a Silanimine Complex and Applications to Multicomponent Coupling. *J. Am. Chem. Soc.* **2011**, *133* (18), 7033–7053.
